# Composition and Critical Mineral Content of Major Industrial Wastewaters: Implications for Treatment and Resource Recovery Technologies

**DOI:** 10.1021/acs.est.6c04293

**Published:** 2026-08-12

**Authors:** Monong Wang, Jaebeom Park, Sui Xiong Tay, Vineet Bansal, Emily J. Rabe, Ryan S. Kingsbury

**Affiliations:** † Andlinger Center for Energy and the Environment, 6740 Princeton University, Princeton, New Jersey 08540, United States; ‡ Department of Civil and Environmental Engineering, Princeton University, Princeton, New Jersey 08540, United States; § Department of Mechanical and Aerospace Engineering, Princeton University, Princeton, New Jersey 08540, United States; ∥ Research Computing, Princeton University, Princeton, New Jersey 08540, United States; ⊥ Membrion, Inc., Seattle, Washington 98119, United States

**Keywords:** critical minerals, resource recovery, industrial wastewater, water chemistry, separation, valorization

## Abstract

Critical minerals (CMs), such as lithium for rechargeable batteries and rare-earth elements for electrical components, are the new foundation of modern economies, technologies, and infrastructure, yet their supply chains are vulnerable to disruption. The widespread presence of CMs in industrial wastewater has motivated the development of wastewater resource recovery or valorization technologies aimed at enhancing supply chain resilience and mitigating environmental impacts. However, wastewater characteristics are highly variable, and data describing them are often limited or incomplete, leading researchers to rely on synthetic substitutes that may not reflect realistic properties. To better guide this research, here we compile and analyze the pH, total dissolved solids (TDS), total organic carbon (TOC), inorganic composition, CM content, and generation rates of wastewater streams from twenty-two individual industrial activities. These activities are associated with six major industries: oil and gas production, metal and coal mining, manufacturing, fossil-fuel power generation, geothermal power generation, and gasification. We conclude our study by (1) identifying industries and waste streams with high CM recovery potential, (2) highlighting common challenges for recovery technologies, and (3) creating representative wastewater compositions for each industrial activity to support the development of realistic and effective recovery technologies.

## Graphical abstract

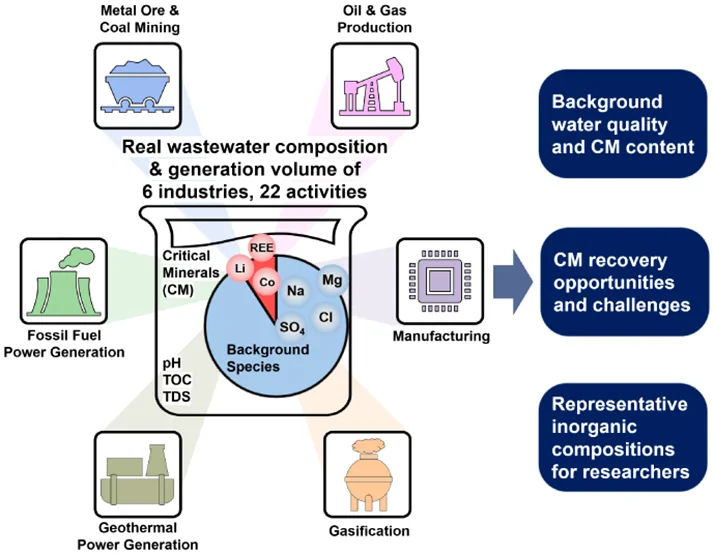

## Introduction

Global industrial activities such as oil and gas production, mining, and manufacturing generate more than 45 billion m^3^ of wastewater each year^1^—roughly 7.5 times the annual volume of oil production.^2^ This huge volume, combined with the frequent presence of toxic chemicals, has long made industrial wastewater an environmental burden and a regulatory liability, necessitating its treatment and motivating reuse. Because industrial wastewater sometimes contains dissolved critical minerals (CMs) that are essential to modern technology, it is increasingly being seen as a potential economic resource as well.^3–8^ For example, metals important for rechargeable battery applications, like lithium, are found in high concentrations in oil and gas produced water,^9^ and rare-earth elements critical for electrical components and catalysis, such as neodymium and lanthanum, have been frequently detected in mine drainage.^10–12^

The recent surge in global demand for CMs,^13,14^ together with the recognition of their supply chain vulnerabilities—often driven by changing export policies and other geopolitical factors—is motivating increased efforts to recover CMs from industrial wastewater.^15–18^ In stark contrast to geographically constrained natural resources, such as mines and CM-enriched aqueous sources like salt lakes and geothermal brines,^19^ industrial wastewater is a distributed resource that is less dependent on the location of raw material reserves. Recovering CMs by “refining” this wastewater thus promises to promote supply chain resilience and circular economy goals.^7^

In addition to addressing supply risks, recovering CMs from industrial wastewater would also provide economic incentives for improved wastewater management. Currently, around 62% of industrial wastewater worldwide receives little or no treatment,^20^ leading to public health and environmental emergencies.^21–24^ For example, in South Africa, hexavalent chromium from mining activities—known for its carcinogenic properties—has been detected in nearby crops at concentrations ranging from tens to hundreds of milligrams per kilogram.^24^ Since several CMs contribute to wastewater toxicity (e.g., chromium, arsenic, and nickel),^25,26^ recovering them for beneficial reuse would not only protect public and environmental health but also reduce legal liability for the industries involved, potentially incentivizing investments in recovery technologies that are not otherwise feasible.

The economic and environmental benefits of recovering CMs have motivated numerous research and commercial efforts to develop efficient wastewater treatment and valorization technologies.^27–30^ Testing such technologies under realistic conditions is critical to their successful development because the performance of most recovery technologies depends sensitively on multiple water characteristics, such as pH, total dissolved solids (TDS), total organic carbon (TOC), and the concentrations of the target CMs and (potentially competing) background species.^31,32^ These characteristics vary greatly among industries and even among activities within a single industry^25,33^ and are critical for evaluating the economic and technological recoverability of CMs. Unfortunately, it is often challenging to obtain samples of real industrial wastewater, and information on its composition—particularly the concentrations of background inorganic species—is often unavailable due to logistical challenges, lack of monitoring, or intellectual property concerns. As a result, many studies rely on synthetic wastewater with compositions that may or may not be representative of real conditions. This disconnect slows the progress of recovery technology development and its translation to real-world applications.

Here, to promote more realistic testing of new recovery, valorization, and treatment technologies, we compile and analyze wastewater characteristics, CM content, and wastewater generation volumes across six industries (Figure 1, panel 1, bold titles). For each industry, we conduct a detailed review of common wastewater-generating activities that involve the use or processing of CMs (Figure 1, panel 1, italicized titles) and summarize globally reported data on wastewater compositions prior to any treatment, including background species, water quality parameters, and CM content (see Table 1), as well as wastewater generation rates. We use the collected data to evaluate CM recovery opportunities across and within industries, compare CM recovery potential with demand from clean energy technologies, identify common recovery challenges posed by background species, and define “representative” inorganic wastewater compositions for each industrial activity for use in scientific or industrial technology research (Figure 1, panel 2–3). We conclude by discussing the limitations imposed by currently available data and the potential implications of our analysis for wastewater valorization economics.

**Figure 1:**
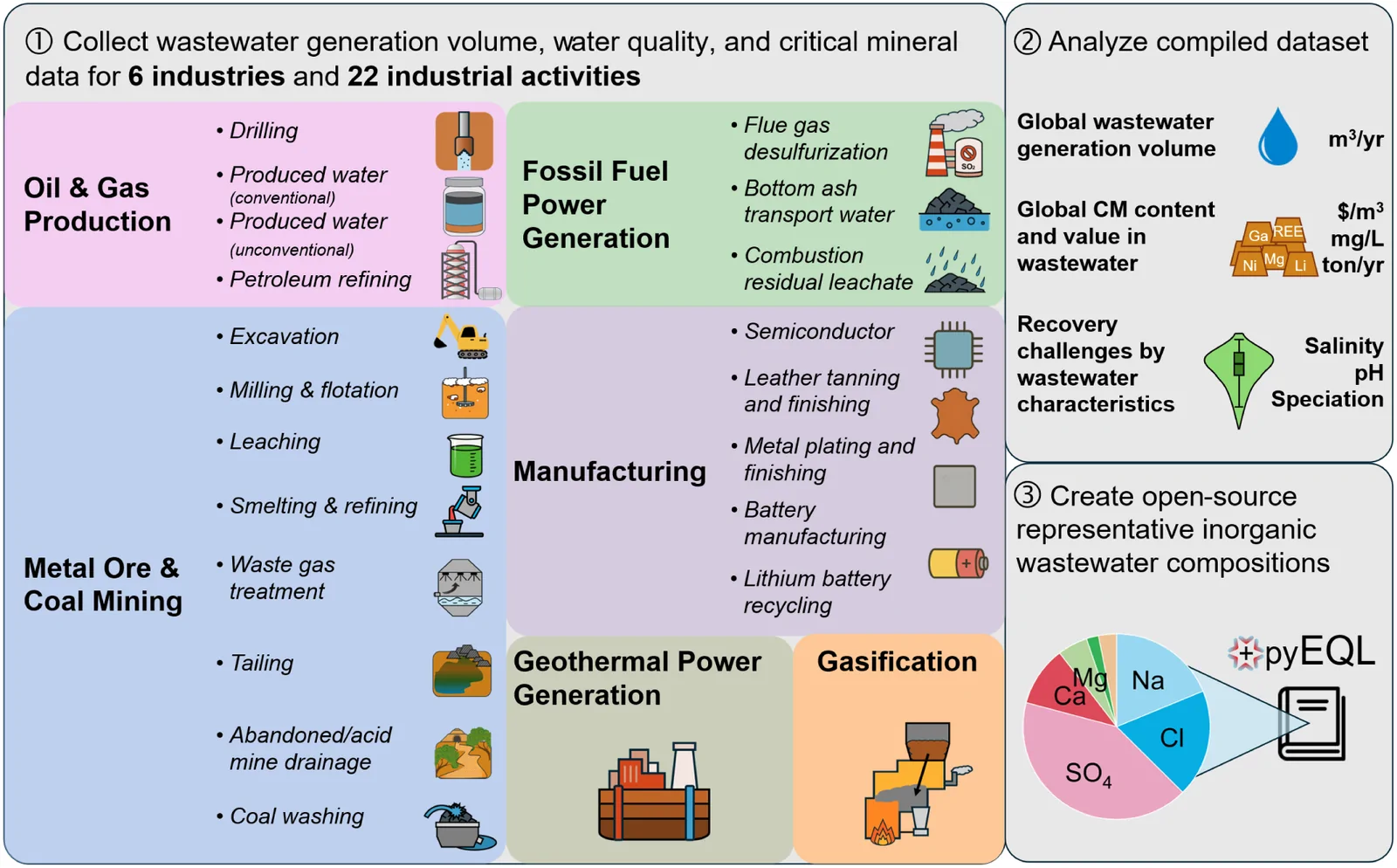
Scope of the study: Compilation and analysis of wastewater characteristics, CM content, and wastewater generation volumes for 22 major wastewater-producing activities across six industries. In panel 1 (left), bold titles within each colored block indicate the six industries studied, and specific industrial activities within each industry are labeled with italicized titles next to their corresponding illustrations. Panels 2 and 3 illustrate the analyses we performed.

**Table 1:**
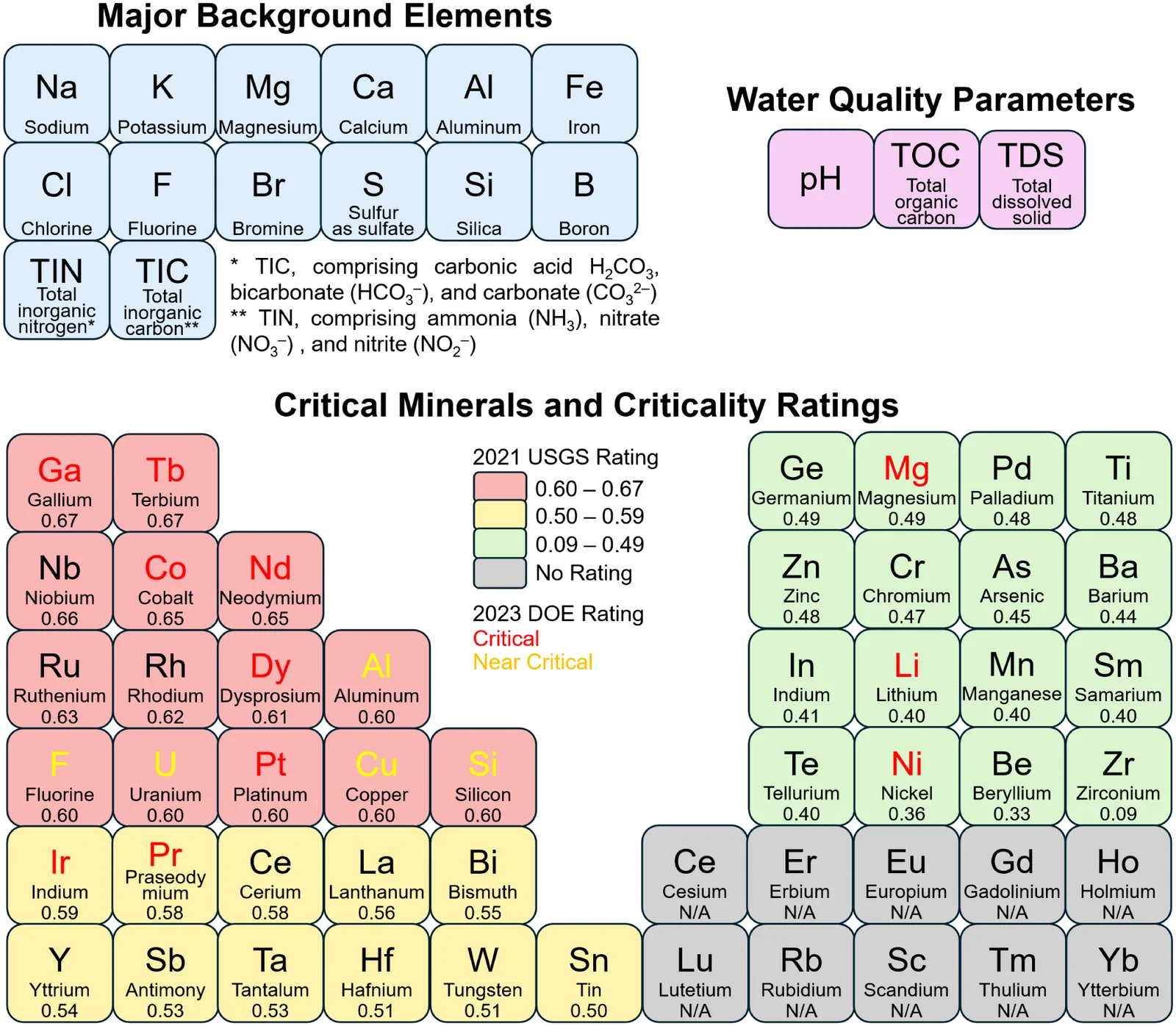
List of Critical Minerals, Background Elements, and Other Water Quality Parameters Considered in This Study^a^

a CMs marked in red and yellow are ranked as “Critical” and “Near Critical,” respectively, in the 2023 U.S. Department of Energy (DOE) 2025–2035 Medium-Term Criticality Matrix.^58^ CMs were identified by combining the DOE list with the 2021 U.S. Geological Survey (USGS) technical report.^57^ Criticality ratings were assigned based on the USGS report.^8^ For CMs present in the DOE report but not assigned a rating by USGS, we assigned ratings of 0.67 and 0.60 for CMs ranked “Critical” and “Near Critical,” respectively. CMs with a gray background were not given a criticality rating but were identified as critical by the USGS.^8^

## Methods

### Industrial Wastewater Characteristics and Generation Volume Data Sources

Our primary data sources were technical reports and open databases published by the U.S. Environmental Protection Agency (EPA),^34–38^ U.S. Geological Survey (USGS),^39–42^ International Energy Agency (IEA), U.S. Energy Information Administration (EIA),^43,44^ National Energy Technology Laboratory (NETL),^45^ Argonne National Laboratory (ANL),^46,47^ American Petroleum Institute (API),^48^ and various industrial technical reports.^49–52^ Examples of major open databases include the 2024 Discharge Monitoring Report Database (DMR),^53^ National Energy Water Treatment and Speciation (NEWTS) Database (accessed by searching “NEWTS” on NETL’s Energy Data eXchange website),^54,55^ USGS database,^40^ and EIA database.^56^ Secondary data sources included review papers, research articles, and textbooks, which often had limited descriptions of real wastewater samples. Although we attempted to obtain data sources that were global in scope, most of our sources are from the U.S. due to language constraints and data availability. To maximize the amount of data available, we included data from all time periods (1964–present).

In the Supplementary Sections S6–S11, we provide, for each industry, an appendix containing a detailed summary of the specific data sources we used and their limitations, a summary of the major wastewater-generating activities, and a discussion of its current status and potential for future development. The complete set of raw water characteristics data and their sources, comprising distinct reports from 182 sources, is provided as Supplementary Data set 1 in.xlsx format. The complete set of raw water generation volume data and their sources, comprising distinct reports from 48 sources, is provided as Supplementary Data set 2 in.xlsx format.

### Identification of Critical Minerals and Background Species

We selected critical minerals (CMs) by combining the CMs lists published by the USGS^57^ and the U.S. Department of Energy (DOE).^58^ Note that from the DOE CMs list, we only included the minerals ranked as “critical” and “near critical” in the “medium-term (2025–2035) criticality matrix”. The criticality rating of CMs was based on the 2021 USGS technical report.^8^ For CMs identified by the DOE that were absent from the USGS source, we assigned a criticality rating based on the highest ratings of minerals in their same category. Specifically, we rated Terbium 0.67 because it was classified in the “critical” category by the DOE, and the highest rating among the minerals in the USGS “critical” category was 0.67 (Gallium). Similarly, all minerals in the “near critical” category were assigned a rating of 0.6, matching that of Aluminum and Fluorine.

We included background elements by combining those frequently identified in groundwater,^59^ surface water,^60^ and seawater.^61^ The cationic species are sodium (Na), potassium (K), magnesium (Mg), calcium (Ca), aluminum (Al), and iron (Fe). Note that we report only the total elemental concentration of these species (i.e., we do not distinguish between the free cations, complexed forms, or different oxidation states). The anionic species are chlorine (Cl), bromine (Br), fluorine (F), silica (Si), boron (B), total inorganic nitrogen (TIN, comprising ammonia (NH_3_), nitrate (NO_3_^–^), and nitrite (NO_2_^–^)), and total inorganic carbon (TIC) (carbonic acid (H_2_CO_3_), bicarbonate (HCO_3_^–^), and carbonate (CO_3_^2–^)). Among these elements, only Na, K, Ca, Mg, Cl, and SO_4_ are considered major environmental species^62^ because they comprise more than 85% of the TDS in natural waters, and aqueous wastes contain most of these species at some level.

The complete list of background species, CMs, and criticality ratings is provided in Table 1 and can also be found in the Supplementary Data set 1, sheet name “Mineral List.”

### Industry Selection Criteria

To select industries for analysis, we screened the industries listed in Title 40 of the U.S. Code of Federal Regulations (CFR), Subchapter N, Parts 400–471, which is used by the U.S. Environmental Protection Agency (EPA) to classify effluent point source categories.^63^ From this list, we selected industries that either 1) involve the use or processing of CMs or 2) discharge >1% of the total wastewater volume in the U.S. These included oil and gas production (Part 435), petroleum refining (Part 419), ore mining and dressing (Part 440), coal mining (Part 434), steam electric power generating (Part 423), metal finishing (Part 433), leather tanning and finishing (Part 425), battery manufacturing (Part 461), and electrical and electronic components manufacturing (Part 469). Collectively, these industries encompass 47% of the effluent point source categories used by the EPA (see Table S37).^63^ The remaining 53% of effluent flow is dominated by drinking water treatment plants (16%), mineral mine processing (13%), and cement manufacturing (10%), with other sources each comprising <8%. We excluded drinking water treatment plants because they are not industrial processes and are not expected to contain CMs (apart from Al or Fe due to residual coagulant). For mineral-processing and cement-manufacturing industries, the total CM content in their wastewater ranked 24th and 41st among the 48 industries listed in the CFR. Therefore, we did not include them in our analysis. We also included four industries that have known CM recovery potential but were not listed in the CFR: coal-based gasification,^64^ geothermal power generation,^65^ semiconductor manufacturing,^66^ and lithium battery recycling.^67^ We grouped all the industries considered into six larger categories for our study, which are shown in Figure 1; the grouping is shown in Table S3.

Individual wastewater-generating activities within each industry were identified by reviewing literature, technical reports, and textbooks. These activities are listed in Figure 1 (panel 1, italicized text) and Table S3. Supporting Information Sections S6-S11 contain detailed descriptions of the typical activities associated with each industry and citations to the specific sources used to identify them. Note that, although several manufacturing activities, including semiconductor manufacturing, leather tanning, plating, battery manufacturing, and lithium battery recycling, could in principle be subdivided further, we did not do so because of data limitations. For example, lithium battery recycling can involve one of many processes, including acid dissolution (hydrometallurgy),^68–70^ pyrometallurgy,^68^ direct physical recycling,^71^ and flotation of black mass.^72^ These steps may generate wastewater with distinct characteristics; however, we consolidate the data from all steps and summarize the major steps and their general associated wastewater characteristics in Supporting Information Section S8.

### Estimated Global Annual Wastewater Volume

We estimated the global annual wastewater volume (*Q_ww_*) generated from each industry based on a wastewater production rate per unit of output, *R*_*ww*–*i*_ (e.g., m^3^ wastewater per m^3^ oil produced), and the annual global product output:

1 $$Q_{ww}\left[\frac{Mm^{3}}{year}\right]=\overset{m}{\underset{i}{\sum }}Q_{ww-i}=\overset{m}{\underset{i}{\sum }}R_{ww-i}\left[\frac{Mm^{3}}{unit\;product}\right]\cdot Product\;output\left[\frac{unit\;product}{year}\right]$$

where *m* is the total number of activities in the industry, *i* represents individual activities, and *Q*_*ww‑i*_ is the wastewater volume from individual activities.

The values and units of output used in *R*_*ww*–*i*_ vary by activity and are listed in Table 2. For example, wastewater generated from metal mining is reported as the volume of wastewater per weight of metal produced (e.g., m^3^/ton-metal produced); wastewater generated from drilling activities for oil and gas production is reported as the volume of wastewater per meter drilled (e.g., m^3^/m-drilled). *R*_*ww*–*i*_ was either obtained directly from relevant literature or calculated based on reported wastewater generation rates and output from representative facilities. Note that due to data limitations, most estimates of *R*_*ww*–*i*_ reflect U.S.-based industrial activities.

**Table 2:** Values and Units for *R*_*ww*–*i*_ of Different Industries^a^

Main industry	Subactivities	*R*_*ww‑i*_	Units
Oil and Gas Production	Drilling^48^	1.59	m^3^/m-drilled
	Produced Water Conventional Oil^46^	9.75	m^3^/m^3^-oil produced
	Produced Water Conventional Gas^46^	482.85	m^3^/Mm^3^-gas produced
	Produced Water Unconventional Oil^75^	0.35	m^3^/m^3^-oil produced
	Produced Water Unconventional Gas^75^	207.86	m^3^/Mm^3^-gas produced
	Petroleum Refining^42^	2.7	m^3^/m^3^-oil processed
Metal Ore and Coal Mining	Total of Excavation, Milling and Flotation, Smelting and Refining, Waste Gas Treatment^76–79^	129.15^a^	m^3^/ton-metal produced
	Spent Leachate^76–79^	0.66	m^3^/ton-metal produced
	Mine Drainage and Tailing^76–79^	1,415	m^3^/day/mine site
	Coal Washing^80^	1.25	m^3^/ton coal produced
Manufacturing	Semiconductor Manufacturing	0.028	Mm^3^/day/plant
	Leather Tanning	32.5	m^3^/ton-raw hide
	Plating	18.87	Mm^3^/year-U.S. plating market
	Battery Manufacturing	29.73	Mm^3^/year-U.S. battery production
	Lithium Battery Recycling	675	m^3^/ton-battery recycled
Fossil Fuel Power Generation	Total of Flue Gas Desulfurization, Ash Transport, and Combustion Residual Leachate^81^	0.474	Mm^3^/year/energy generating unit (EGU)^b^
Geothermal Power Generation	N/A^46,82^	15.32	kg/kwh
Gasification	N/A	5329	m^3^/Mm^3^-gas produced

a This value represents the median across all of the mineral types. However, individual *R_ww‑i_* values for iron, lead, zinc, copper, coal, and other metals were used for more accurate estimation of wastewater generation and were not listed here. These values are listed in Supporting Information Section S5.2.

b Electric generating unit (EGU): a power-generating unit of a power plant. The exact equipment included in the unit depends on the power plant design. For example, in the case of a steam electric generation unit, the EGU consists of all equipment involved in fuel delivery to the plant site, as well as individual boilers, any installed emission control equipment, and any steam turbine/generators dedicated to generating electricity. The complete definition and more examples can be found in the Washington Administrative Code, Carbon Dioxide Mitigation Program, WAC 463–85–110.^83^

The product output was obtained from relevant literature or databases. For example, the mass of globally produced zinc from mining was 12 million tons in 2024, based on the USGS 2025 Mineral Commodity Summaries report.^73^ The estimated total drilling distance in 2024 was 787,985,590 m, based on the U.S. Energy Information Administration statistics analysis.^74^

The calculated *Q_ww_* values are compared with existing references or verified through expert consultation to ensure accuracy. Detailed calculations and references for *R_ww,_* *Q_ww_*, and the global production rate of each industry are documented in Supporting Information Section S5 and Supporting Information Data Set 2.

Using the calculated wastewater volume, we estimated the global CM generation for each industry by

2 $$Estimated\;global\;CM\;mass\left[\frac{\mathrm{ton}}{year}\right]=\overset{m}{\underset{i}{\sum }}C_{i-median}\left[\frac{\mathrm{mg}}{L}\right]\cdot Q_{ww-i}\left[\frac{Mm^{3}}{\mathrm{year}}\right]$$

where *C*_*i‑median*_ is the median concentration of CMs for each individual activity within the industry.

### Criticality-Weighted Concentration

The criticality-weighted concentration (mg/L) of each wastewater sample in a data source was calculated as

3 $$Criticality-weighted\;concentration\left[\frac{\mathrm{mg}}{L}\right]=\frac{\overset{n}{\underset{i}{\sum }}C_{i}\cdot criticality\;rating_{i}}{n}$$

where *n* is the total number of CMs present in the wastewater sample, *i* represents individual CMs, and *C_i_* (mg/L) is the the concentration of the CM.

### Total Economic Value of CMs in Wastewater

The total economic value of CMs in wastewater ($/m^3^) was calculated by multiplying the CM concentration (g/m^3^) by the 2024 annual average price of the CM ($/g of element) and then summing the resulting values across all CMs in the wastewater sample. We obtained prices of all CMs from the USGS 2025 Mineral Commodity Summaries report,^73^ except for uranium, praseodymium, and samarium, for which we consulted other sources.^84–86^ In cases where the price was provided for a CM compound rather than a pure elemental form, we converted the compound price to an element-based price using molecular weight ratios. For example, the reported price for vanadium pentoxide ($5.45/lb) corresponds to a vanadium price of $0.0214/g-V. The complete list of CM prices and their corresponding grades is listed in Table S2.

### Calculation of TDS, TIN, and TIC

We calculated the median TDS value for each data source reported in Figure 6. For sources where TDS values were not available but major background species were reported, we calculated the TDS by summing all reported dissolved species. We report TIN and TIC in units of mg/L of nitrogen or carbon, respectively. For data missing TIN, we calculated it by summing the concentrations of NH_3_, NO_3_^–^, and NO_2_^–^ when data were available. The TIC was calculated by summing the concentrations of H_2_CO_3_, HCO_3_^–^, and CO_3_^2–^ when data were available.

### Proportion of Background Elements and Representative Inorganic Compositions for Research

Wastewater compositions reported in the literature are often incomplete due to limitations in detection methods and/or a lack of incentives to perform full characterization of all constituents. As a result, we observed discrepancies between the reported TDS and the TDS calculated by summing all reported solutes. Although such differences can usually be attributed to the presence of organics, in many cases, the reported compositions also did not satisfy electroneutrality, making it impractical to use the reported data directly as model wastewater compositions for research or chemical analysis. To address this issue, we defined simplified, “representative” *inorganic* compositions for each industrial activity in which we (1) calculated the charge (im)­balance; (2) added an appropriate charge-balancing species to ensure electroneutrality (see below); (3) performed chemical equilibrium calculations to obtain the final speciation; and (4) calculated the proportion of each element in the TDS. This procedure ensured that our compositions are electroneutral and represent a state of chemical equilibrium, making them ready to use in laboratory studies or geochemical analysis. Since the purpose of this analysis was to quantify major inorganic constituents, and in order to facilitate chemical equilibrium calculations, elements that constituted less than 0.1% of the original reported TDS were discarded prior to Steps (1)–(4). The detailed, step-by-step workflow we followed is illustrated in Figure S1. The final, “representative” inorganic compositions for each industrial activity are detailed in Section S2, along with example code illustrating how to automatically retrieve them using the pyEQL water chemistry library.^87^

We identified appropriate charge-balancing elements (Table S1.1) based on our understanding of the common practices within each industrial activity (e.g., the raw materials being processed, chemicals being used, and the quality of consumed water). For industrial activities that had relatively comprehensive reports of wastewater composition, such as oil and gas production and fossil fuel power generation, we simply selected background species that dominate the reported TDS. For activities in which the compositions were less complete, we selected charge-balancing species based on our understanding of the industrial process, including the raw water used, the materials processed, and the chemicals used (see Sections S6–S11). For example, gas scrubbers commonly employ lime or soda ash, which will result in sodium and/or calcium appearing in waste gas treatment wastewater, even though these ions were not reported. If multiple charge-balancing ions were equally plausible, such as sodium and calcium in the gas scrubber case, we charge-balanced the original composition with each one (separately) and reported the median composition. After electroneutrality adjustment, we summed the concentrations of all inorganic species to determine the TDS and then divided the respective total element concentrations by the TDS to obtain the proportions presented in Figure 7. All analysis was done using pyEQL^87^ version 1.4.0.

### Data Aggregation, Cleaning, and Summary Statistics

We present the distribution of wastewater quality data using a violin plot based on kernel density estimation, with the density truncated at the observed minimum and maximum values. Box plots are also presented along with the violin plots, where boxes depict the 25th to 75th percentile range and whiskers depict the 5th to 95th percentile range. Functions in the Numerical Python (NumPy) Library^88^ were used to calculate the percentiles (via the “percentile ()” function, using the linear interpolation method), as well as other statistical analyses.

## Results

### Critical Mineral Recovery Potential by Industry

We first estimate the mass of individual CMs in wastewater from each industry, then compare the recovery potential among industries, considering the total CM mass, criticality, concentration, and economic value. Figure 2a shows the estimated mass of individual CMs, calculated by multiplying the median CM concentrations by the estimated global volume of wastewater (Figure 2b; see **Methods** and Supporting Information Section S5 for calculations). We observed that Mg and Si are the most abundant CMs, exceeding 1 million tons per year and appearing in wastewater from all industries except gasification. On the other hand, some CMs were reported by only a few industries. For example, Pr and Dy were reported almost exclusively in mining and oil and gas production (∼1,000 tons/year), while Pd was mainly found in mining (∼100 tons/year). Many other observations can be made depending on specific recovery objectives and are not elaborated on here. For example, CMs relevant to manufacturing batteries, including Li, Co, and Ni,^89^ were found in high amounts in mining, manufacturing, and oil and gas production wastewater (10,000–100,000 tons/year).

**Figure 2:**
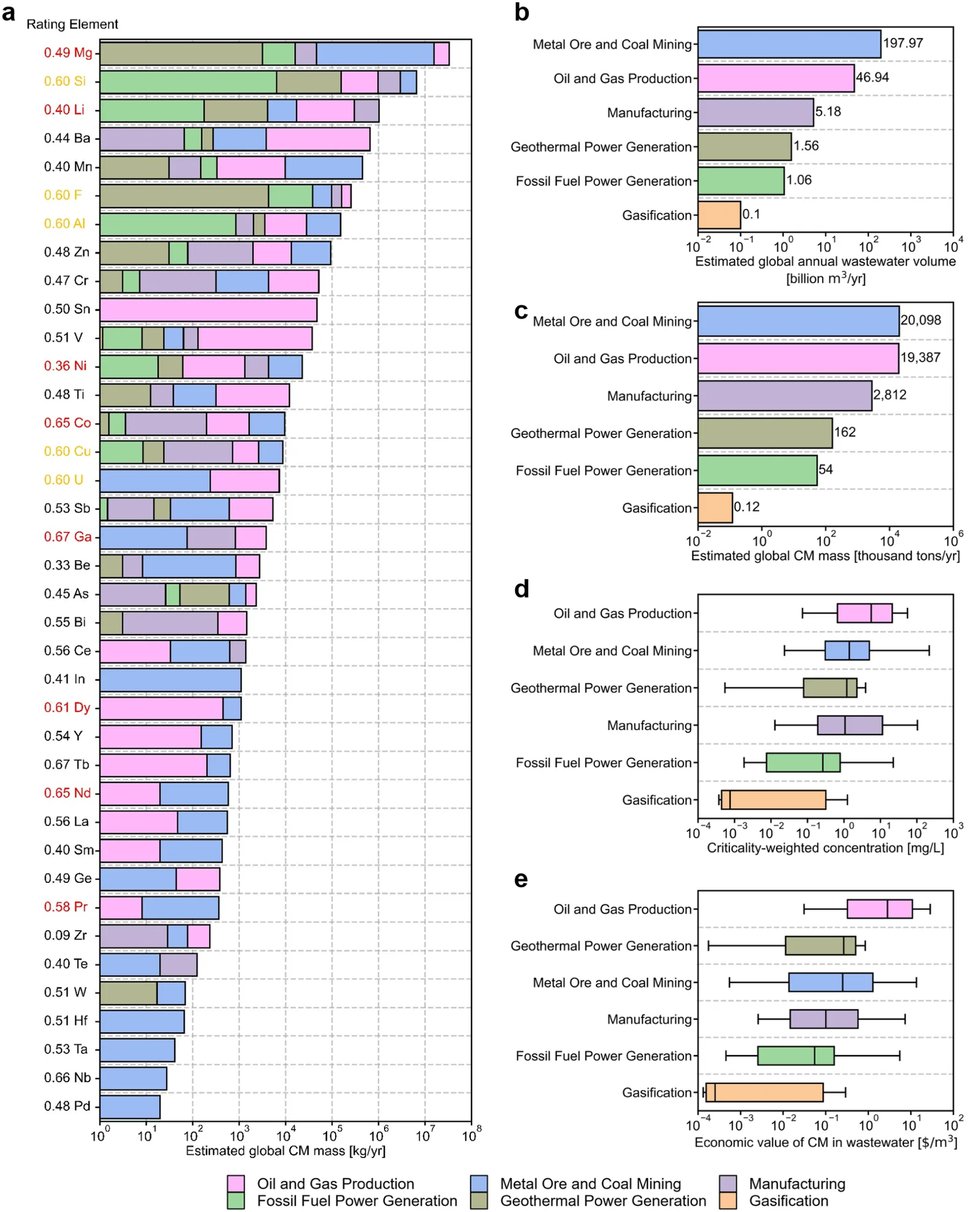
Global critical mineral content and estimated wastewater volume from major industries. (a) Estimated mass of individual CMs in wastewater globally (tons/year, log scale). Note that for gasification, only vanadium was significant among all CMs. CMs marked in red and yellow are ranked as “critical” and “near critical” in DOE’s medium-term criticality matrix, respectively. (b) Estimated global annual wastewater volume (billion m^3^/year, log scale). (c) Estimated total mass of CMs in wastewater globally (thousand tons/year, log scale). (d) Criticality-weighted concentration of CMs (mg/L, log scale), calculated as the concentration of CMs weighted by their respective criticality ratings. (e) Total CM values in wastewater ($/m^3^, log scale), calculated as the concentration of CMs multiplied by their 2024 annual average market value. Detailed calculations for each can be found in the Methods section.

To compare the total CM mass available for recovery across industries, we summed the masses of all individual CMs within each industry and present the results in Figure 2c. The mining and oil and gas production industries contained the highest estimated total CM mass (∼20 million tons/year) due to their large total volume (Figure 2b), followed by manufacturing and geothermal. Industries with lower total CM mass include fossil fuel power generation, which contained only ∼50,000 tons of CMs due to relatively lower volumes and CM concentrations, and gasification, which was the least significant CM resource with only ∼120 tons total mass. Unsurprisingly, the estimated global CM mass in wastewater bears little correlation with the minerals’ criticality ratings (Figure S3), since criticality ratings generally reflect economic rather than physical scarcity.

The CM criticality and concentration are tightly connected to the economic value and recoverability of wastewater. We therefore define a “criticality-weighted concentration” as the sum of CM concentrations weighted by their respective criticality ratings (Figure 2d, see Methods for calculations). Higher values thus indicate greater CM concentrations, greater criticality, and/or a greater mass available, all of which increase the value proposition for CM recovery from a particular industry. To further evaluate the potential economic value of fully valorizing the wastewater, we also calculated the total value of the dissolved CMs ($/m^3^) using 2024 average market prices (Figure 2e, see Methods for calculations).

As shown in Figure 2d–e, among all industries in our study, the oil and gas industry is likely to be the most valuable one for CM recovery. Its wastewater has the highest median criticality-weighted concentration (5.6 mg/L) and the highest median economic value ($2.82/m^3^). This is followed by metal ore and coal mining and geothermal power generation, which have median concentrations of 1.4 and 1.2 mg/L and economic values of $0.25 and $0.26/m^3^. The manufacturing industry has similar value as geothermal, which has a median concentration of 1.07 mg/L and CM value of $0.1/m^3^. Fossil fuel power plant and gasification are likely to be the least favorable for CM recovery, with median concentrations of 0.27 and 0.0008 mg/L and economic values of $0.06 and $0.0003/m^3^, respectively.

### Potential Contribution of Industrial Wastewater Critical Mineral Recovery to Clean Technology Demand

To contextualize the significance of the opportunity presented by “refining” industrial wastewater to obtain CMs, Figure 3 compares the estimated mass of selected CMs (i.e., the potential supply) with the amount consumed by clean energy technologies (i.e., the demand) as of 2024. Depending on the CM, clean energy technologies currently account for 30–60% of the global demand, and this share is expected to increase to 50–90% with the ongoing global energy transition.^73,90^ Note that we compare demand only from clean energy technologies because they represent one of the most significant factors driving new demand for CMs. Whereas legacy demands for CMs have largely been met by established mines, it is the growth in CM demand that is stimulating interest in wastewater valorization.

**Figure 3:**
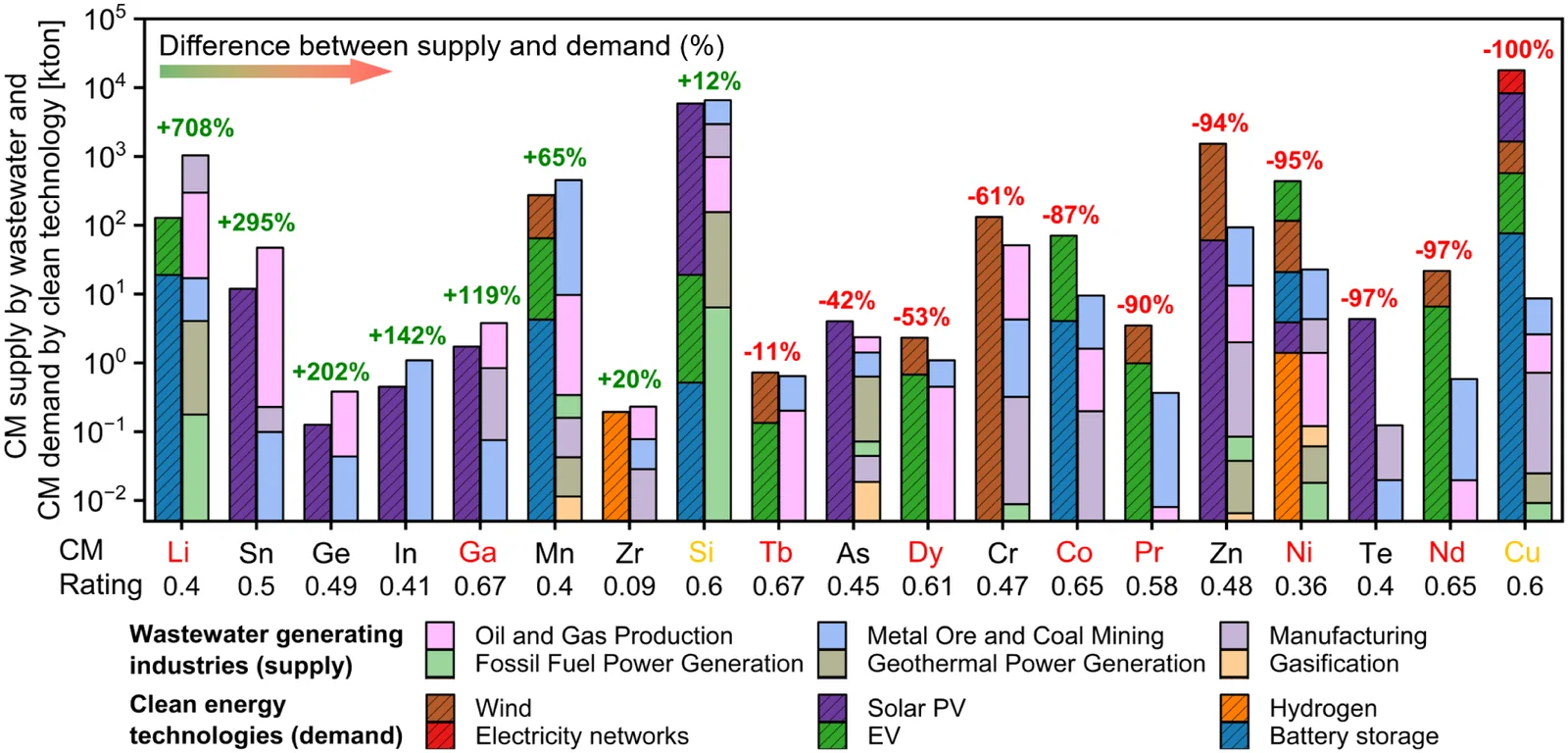
Comparison of estimated global CM mass in industrial wastewater and CM demand by clean energy technologies in 2024 ^90^ (kton, log scale). Labeled values are the percentage difference between the estimated CM mass in industrial wastewater and the demand (%). Negative values (red) indicate that the CM mass is lower than the demand; positive values (green) indicate that the CM mass exceeds the demand. CMs marked in red and yellow are ranked as “critical” and “near critical” in DOE’s medium-term criticality matrix, respectively.

Figure 3 suggests that recovering all of the CMs in industrial wastewater could fully satisfy the demand for Li, Sn, Ge, In, Ga, Mn, Zr, and Si. Although it will certainly not be economical to recover CMs from every waste stream, this analysis nonetheless highlights several especially promising opportunities, where the potential supply far exceeds the demand, including Li and Sn. The potential Li supply by wastewater was 1,035 thousand tons (kton), exceeding total demand in 2024 (128 kton) by 708%.^90^ For Sn, 47 kton can be supplied, which is equivalent to 295% of the total demand (12 kton). Notably, oil and gas wastewater alone contains 99% and 27% of the total supply of Sn and Li, which already equates to 229% and 73% of demand, respectively. By contrast, the potential contribution of industrial wastewater to CM supply was marginal for Cu, Nd, Te, Ni, Zn, and Pr, with less than 10% supply relative to demand.

Altogether, the high-level analysis shown in Figures 2–3 suggests that mining, oil and gas production, and manufacturing are the industries with the highest recovery potential due to their high concentration or large available mass. However, the total CM masses in Figures 2–3 aggregate data from all waste streams within a given industry. While this approach is useful for comparing the global recovery potential across industries, it obscures variations in CM content and water quality among specific waste streams, which constitute more realistic targets for valorization.

### Critical Mineral Recovery Potential from Individual Industrial Activities

Industrial processes typically involve multiple activities that generate waste streams with distinct CM contents and water quality. For example, oil and gas wastewater comprises drilling wastewater, produced water from conventional and unconventional production, and petroleum refining wastewater (Figure 1, Supporting Information S6), each of which has distinctive characteristics. While streams from individual activities are often combined into a single effluent for discharge or treatment, in many cases, it could be beneficial to perform CM recovery prior to mixing (e.g., if one individual activity generates wastewater with higher CM content and/or fewer interfering species than others). To assess such opportunities, we reviewed the typical wastewater-generating activities and treatment processes within each.

Figure 4 summarizes the number and median concentrations of clean energy-relevant CMs in specific industrial activities. Comprehensive descriptions of all industrial activities and detailed composition data are provided in Sections S6–S11 and Supplementary Data set 1. Some activities from the top three industries for CM recovery contain a relatively small number of CMs at relatively high concentrations (e.g., spent leachate from mining), whereas others contain many CMs but at low concentrations (e.g., mine drainage). Industrial activities with fewer CMs at higher concentrations are generally more promising recovery targets because the selectivity of state-of-the-art treatment technologies among multiple (potentially competing) CMs is limited.^62^ Leather tanning, metal plating, and lithium battery recycling from the manufacturing industry, as well as spent leachate and waste gas treatment from mining, all appear to meet these criteria. Each of these activities reported eight or fewer CMs, and those that were present (Cu, Ni, Zn, Cr, As, Si, Sn, and Li) often had concentrations of 10–100 mg/L. In contrast, activities such as unconventional produced water from oil and gas production and mine drainage from mining, which had 13 and 19 CMs, respectively—most at concentrations below 1 mg/L (except Li, Sn, and Si in produced water and Mn and Si in drainage)—are relatively challenging for valorization.

**Figure 4:**
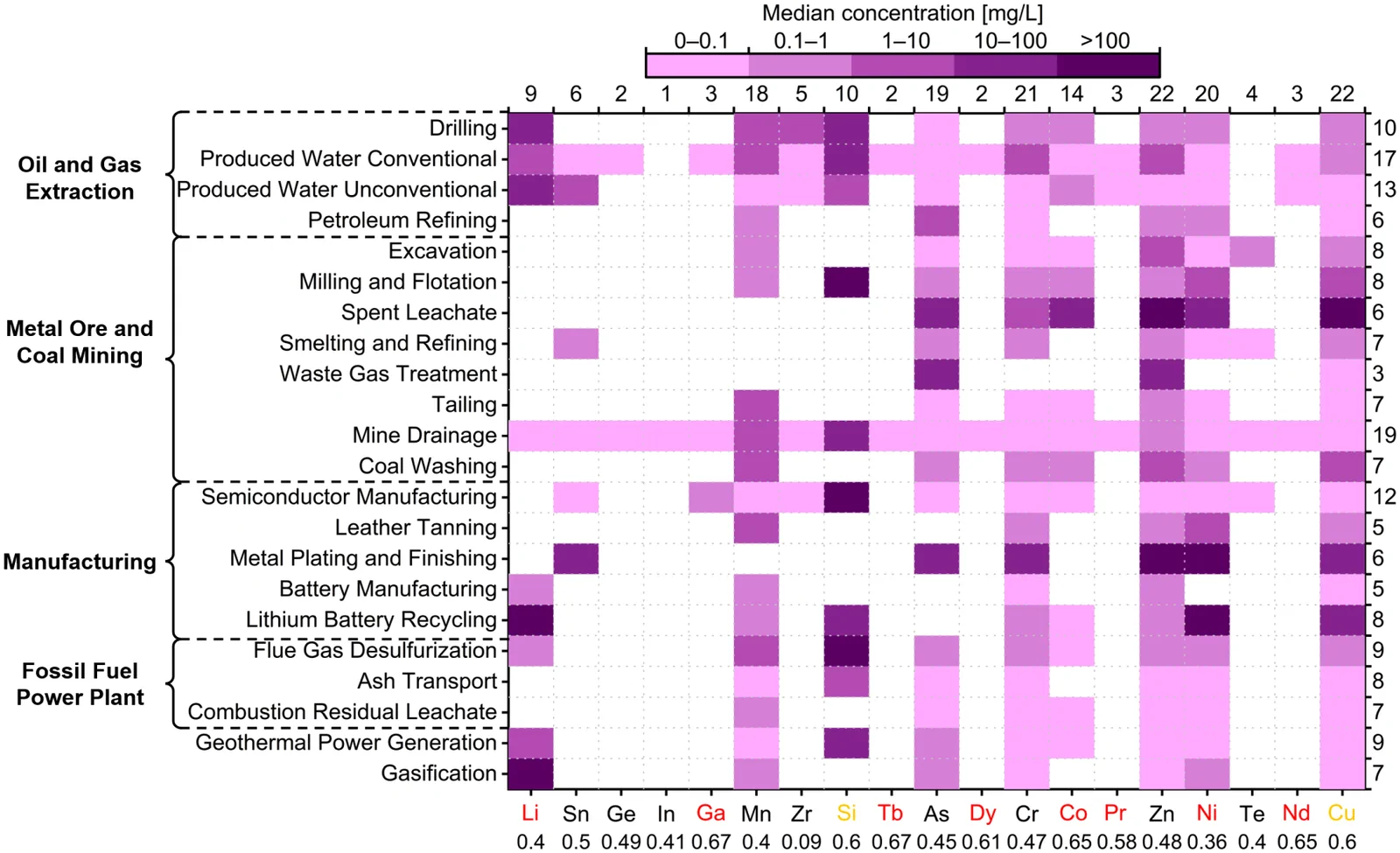
Median concentrations of energy-relevant CMs reported in wastewater from specific industrial activities. The numbers on the top axis are the number of activities reporting concentrations greater than 0 mg/L for each CM. The numbers on the right axis are the number of CMs reported in each activity.

We also observed variations in how frequently CMs appeared in the activities. CMs such as Cu, Ni, Zn, and Cr were reported in over 20 activities, with concentrations typically below 10 mg/L. On the other hand, some CMs were only reported in a few activities. For example, Zr appeared in only five activities, with the highest concentration in drilling wastewater; Sn appeared in six activities, with the highest concentration in metal plating and finishing. While this observation suggests that certain CMs are only recoverable from a select few industrial activities, it may also reflect a lack of monitoring or available data—a limitation we elaborate on in the **Discussion** section.

### Variability in Critical Mineral and Rare-Earth Element Concentrations across Industrial Activities

The analysis presented thus far has been based on median concentrations of CMs; however, we emphasize that the concentrations vary widely among individual wastewaters due to differences in raw materials, industrial activities, as well as the geographical location of the waste streams. To illustrate the magnitude of this variability, we show in Figure 5 violin plots of the reported concentrations of selected CMs that are energy-relevant (Table 1, CMs marked in red and yellow) and/or are rare-earth elements (REEs) as an example. The distribution of all CMs across all studied activities is provided in Supplementary Sections S6–S11.

**Figure 5:**
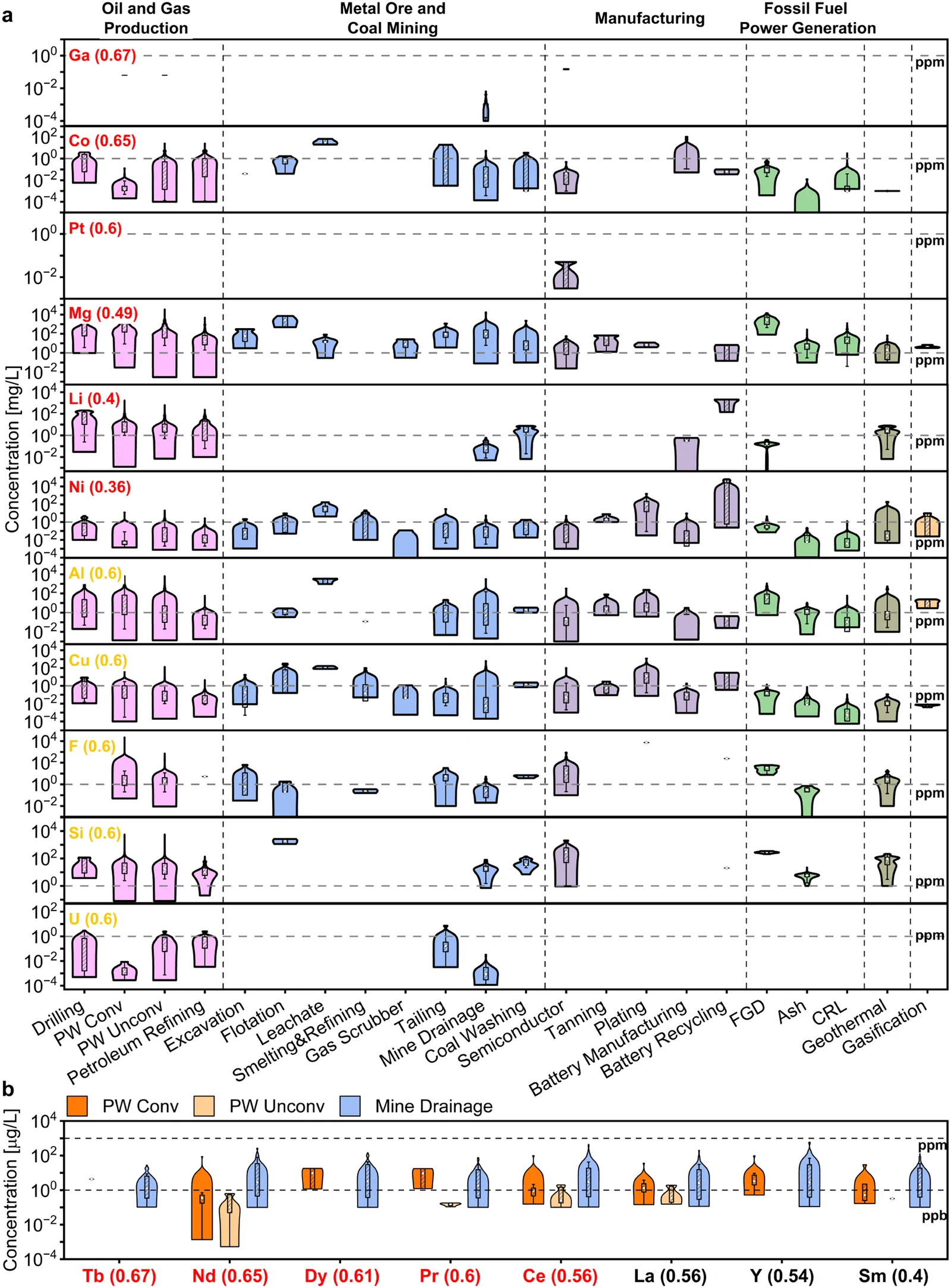
Concentration distribution of critical minerals in wastewater from individual activities. (a) All energy-related CMs except for rare-earth elements (mg/L, log scale); (b) rare-earth elements (μg/L, log scale). The violin plots are truncated by the actual maximum and minimum value of the data, showing flat boundaries at both the top and bottom of the plot. Values in parentheses are the criticality ratings of the CMs. CMs marked in red and yellow are ranked as “critical” and “near critical” by DOE, respectively. Horizontal dashed lines represent 1 ppm (ppm, equivalent to mg/L) concentration in panel (a) and 1 part per billion (ppb, equivalent to μg/L) in panel (b). Industrial activities within the same industry are shown in the same color. In the *x*-axis labels in (a), PW Conv = produced water from conventional production, PW Unconv = produced water from unconventional production, Leachate = spent leachate, Gas Scrubber = waste gas treatment, Tanning= leather tanning and finishing, Battery Recycling= lithium battery recycling, FGD = flue gas desulfurization, Ash = ash transport water, CRL = combustion residual leachate, Geothermal = geothermal power generation.

Most CMs exhibited large concentration ranges within a single industrial activity; however, the extent of the variability differed widely. In conventional produced water, for example, the median Li concentration was 5.36 mg/L, with the 5th–95th percentile range spanning 2 orders of magnitude (1–100 mg/L) and the minimum–maximum range spanning 7 orders of magnitude (0.001–1,700 mg/L; Figure 5a). By contrast, in the same wastewater, the median Co concentration was only around 0.001 mg/L, the 5th–95th percentile range spanned roughly 1 order of magnitude (0.0006–0.007 mg/L), and the difference between the minimum and maximum values spanned about 3 orders of magnitude. Critical REEs were only reported in mine drainage and produced water, with concentrations in the μg/L range (Figure 5b). Among them, mine drainage exhibited the highest REE concentrations (typically above 0.1 μg/L) and the smallest deviation (2–4 orders of magnitude).

### Recovery Challenges Posed by Water Quality

Valuable separation targets like CMs typically constitute only a small fraction of the total dissolved solids (TDS) in wastewater; the properties of wastewater are instead dominated by background species such as sodium (Na), calcium (Ca), magnesium (Mg), chlorine (Cl), sulfur (as SO_4_^2–^), and total inorganic carbon (TIC) (see Table 1 for the full list of species). These species, together with other water quality parameters (e.g., pH, TDS, and TOC), strongly influence the design and implementation of separation technologies. For example, for absorption-based technology, the presence of common ligands such as fluoride and certain pH conditions alters the chemical form of the CMs (e.g., Al, Ni, and Mg for pH-dependent complexes such as Al­(OH)_*n*_^–*n*+3^, NiCl_*n*_^–*n*+2^, and MgSO_4_)^91^ which may interact differently with absorption materials than their free-ion forms. For membrane technology, TDS, water hardness, and TOC dictate pretreatment, scaling and fouling potential, and required operating conditions, which may complicate recovery steps and increase the cost. In addition, background species with similar size and charge to CMs (e.g., Mg^2+^ and Ca^2+^ vs Ni^2+^ and Co^2+^) often compete or interfere with their separation, regardless of the technology used.^62^ For all these reasons, evaluating separation technology using representative background water quality is essential to successful translation out of the laboratory. To facilitate this, Figures 6–Figure 7 present the pH, TDS, hardness, TOC, and inorganic composition for each industrial activity, as well as examples to demonstrate the effect of water quality on CM speciation. The complete data set used in these figures is provided in Supporting Data set 1 and the figures and tables in Section S6–S11.

**Figure 6:**
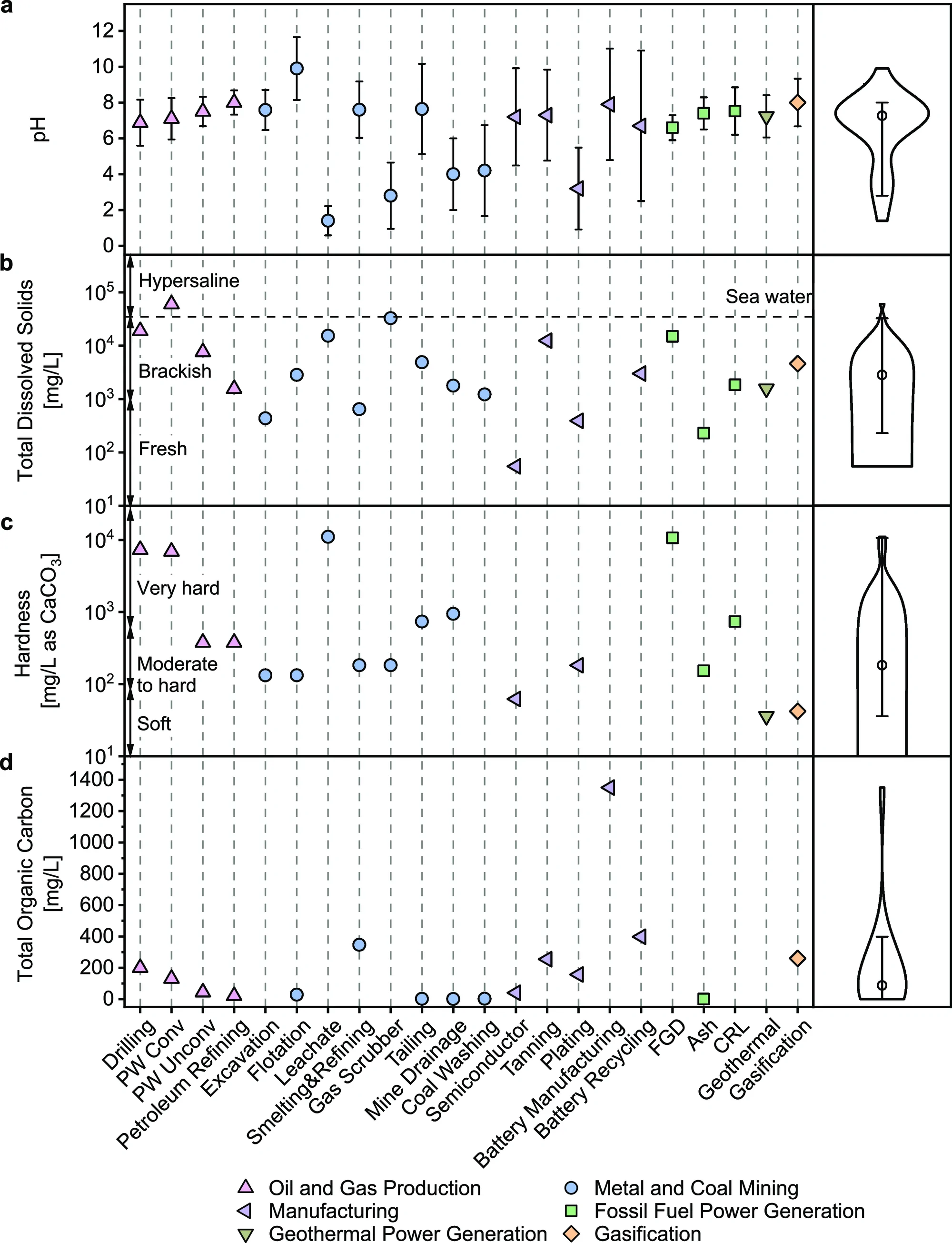
Background industrial wastewater quality. Distribution of (a) pH, (b) median TDS (mg/L, log scale), (c) median hardness (mg/L as CaCO_3_, log scale), and (d) median TOC (mg/L). Violin plots to the right of each panel show the distribution of data across all activities. Data points of the same color represent the median water quality parameters of individual activities within the same industry. Error bars for TDS, hardness, and TOC were not shown in the figures due to their extremely wide variations. Data were unavailable for activities that show no data points. TDS (mg/L) is categorized as <1,000 = freshwater; 1,000–35,000 = brackish water; >35,000 = hypersaline water.^92^ Hardness (mg/L as CaCO_3_) is categorized as <60 = soft; 60–180 = moderate to hard; >180 = very hard.^93^ In the labels of (d), PW Conv = produced water from conventional production, PW Unconv = produced water from unconventional production, Leachate = spent leachate, Gas Scrubber = waste gas treatment, Tanning= leather tanning and finishing, Battery Recycling= lithium battery recycling, FGD = flue gas desulfurization, Ash = ash transport water, CRL = combustion residual leachate, Geothermal = geothermal power generation.

**Figure 7:**
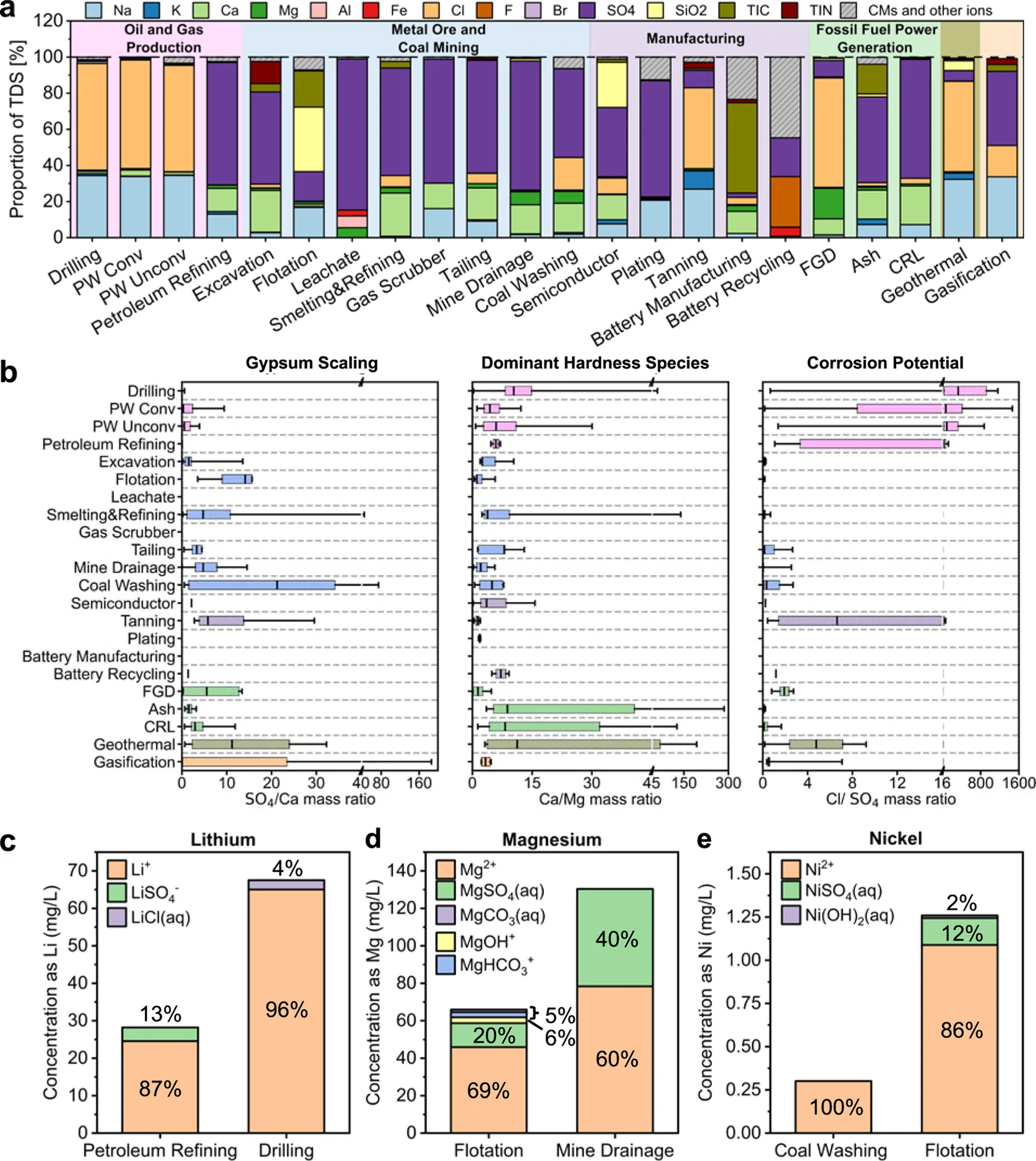
Proportion of individual background species in TDS and their effect on critical mineral speciation and recovery challenges. (a) Median proportion of background species in TDS. Proportions reflect the median composition after electroneutrality adjustment (see Methods). Hatched gray shading represents CMs and minor background species that were included in the electroneutrality calculation. Comparison of the mass ratio of (b) sulfate and calcium (left), calcium and magnesium (middle), and chlorine and sulfate (right). (c) Concentration (mg/L as Li) and proportion (%) of Li species present in petroleum refining and drilling wastewater. (d) Concentration (mg/L as Mg) and proportion (%) of Mg species present in flotation and mine drainage wastewater. (e) Concentration (mg/L as Ni) and proportion (%) of Ni species in coal washing and flotation wastewater. The labels of (c)–(e) are the proportion of each species. In the legend and *x*-axis labels of (a)–(e):PW Conv = produced water from conventional production, PW Unconv = produced water from unconventional production, Leachate = spent leachate, Gas Scrubber = waste gas treatment, Tanning= leather tanning and finishing, Battery Recycling= lithium battery recycling, FGD = flue gas desulfurization, Ash = ash transport water, CRL = combustion residual leachate, Geothermal = geothermal power generation.

Figure 6a shows that most industrial wastewaters had a median pH near neutral, typically in the range of 6–8. However, the waste streams identified previously as having high CM recovery potential, including spent leachate, metal plating, and waste gas treatment, had extremely low median pH values of 1.4, 3.2, 4.0, and 2.8, respectively. These observations indicate that low pH will be an unavoidable challenge for CM recovery efforts.

Figure 6b shows the median TDS levels. High TDS, comparable to typical brackish water levels (10^3^–10^4^ mg/L), is most common. Extreme TDS levels, with the same order of magnitude as seawater (10^4^ mg/L) or higher, were found in wastewater from tanning in manufacturing, drilling, and produced water (conventional) in oil and gas production, as well as spent leachate and waste gas treatment in mining. Lower TDS levels that close to freshwater (less than 10^3^ mg/L), were observed for semiconductor and plating in manufacturing. These observations indicate that high TDS is also a common challenge for high CM-value waste streams.

Figure 6c shows that most industrial activities generate moderately hard to very hard wastewater; the only activity whose wastewater was soft is tanning wastewater. Thus, it is likely that most CM recovery technologies will need to address the scaling challenges. As we elaborate on in the following sections, detailed analysis of the inorganic composition, coupled with chemical equilibrium modeling, can be used to inform both scaling potential and separation performance.

Finally, Figure 6d shows that industrial wastewater typically has TOC in the range of a few to hundreds of mg/L; the highest median TOC (1,400 mg/L) was reported for the leather tanning industry. In general, the organic compounds in industrial wastewaters originate from (1) the industrial process itself, such as flotation agents used in mining and drilling fluid additives; (2) raw materials, such as fossil fuels; and/or (3) natural introduction during wastewater management, such as humic acid in mine drainage. Examples and references for the possible organics associated with each industrial activity are provided in Supporting Sections S6–S11. Depending on the source and type of organic species present, recovery technologies are likely to experience challenges such as fouling and metal chelation to varying extents. However, because the complexity and variability of organics are much greater than those of inorganics, and given the significant reporting gaps in organic speciation data, we do not elaborate on this topic and instead suggest that it be investigated in the future.

In summary, low pH, brackish- to seawater-level TDS, high water hardness, and the presence of organics are common characteristics of industrial wastewater, particularly in the high-value waste streams identified in **Section 3**. We also emphasize the wide variation in water quality, as reflected by the broad TDS range from 100–10,000 mg/L. This occurs not only between industries but also among activities. For example, individual streams from semiconductor manufacturing that involve acid use (e.g., etching) had low pH values close to 1 (Supporting Section S8.1). However, these streams may be combined with higher pH streams before discharge, such as cleaning wastewater, resulting in neutral pH in many reported values. Another example is produced water from oil and gas production, whose TDS may vary by up to 10^5^ mg/L among different hydrocarbon formations (Figure S7). More information on water quality variation for each activity and the underlying causes, along with other quality parameters, is provided in Supplementary Sections S6–S11.

### Recovery Challenges Posed by Competing Background Species

To gain insights into specific background species that might complicate CM recovery or separation, we present the median proportions of TDS attributable to individual background species in Figure 7a. Other constituents, including CMs and minor background species, are shown collectively in the figure (hatched gray shading). Note that the proportions were calculated after adjusting reported compositions for electroneutrality (see Methods). The proportions calculated directly from the originally reported data, without electroneutrality adjustment, are provided in Figure S2.

As shown in Figure 7a, major background species, including Na, Cl, Ca, Mg, and SO_4_, accounted for more than 50% of the TDS in all waste streams. Notably, SO_4_ was found in wastewater from nearly every industrial activity and often comprised more than 20% of the TDS. The high proportion of SO_4_ appears to be a distinguishing feature of industrial wastewater that is distinct from seawater (where SO_4_ only comprises 7.8% of TDS).^61^ Ca and Na were also prevalent, although often with lower proportions. We note that the visually high proportions of sulfate in Figure 7a are partly an artifact of the TDS being expressed in mass-based units. The equivalent weight of sulfate (48 g/eq of charge) is larger than that of most cations (e.g., 23 g/eq for Na); therefore, even in a simple solution of sodium sulfate, where the equivalent concentrations of Na and S are equal, sulfate would contribute more than 67% of the TDS. Nevertheless, it is clear that sulfate is ubiquitous in industrial wastewater.

The relative proportions of monovalent and divalent ions varied across activities, which has implications for treatment design and scaling control (e.g., nanofiltration primarily removes divalent ions, whereas reverse osmosis removes all ions).^62,94^ Monovalent ions, dominated by Na and Cl, account for ∼80% of the TDS in drilling and PW from oil and gas production, geothermal, and tanning in manufacturing wastewater. On the other hand, in mining and fossil fuel power generation wastewaters, divalent ions comprise ∼40–60% of TDS, dominated by Ca, Mg, and SO_4_.

Species other than Na, Cl, Ca, Mg, and SO_4_ were less prevalent, although some may create recovery problems even in moderate or low proportions. For example, around 10–20% combined Al and Fe were found in spent leachate and Li-battery recycling wastewater, respectively, which may cause unwanted coprecipitation with CMs;^95^ around 28% F was found in Li-battery recycling wastewater, which can form complexes with CM ions;^96^ around 20–36% Si was found in semiconductor and flotation wastewater, which can cause colloidal fouling.^97^ High concentrations of barium (Ba) and strontium (Sr) are found in oil and gas wastewater, potentially contributing to BaSO_4_ and SrSO_4_ scaling that are notoriously hard to remove.^98^ Other CM-specific recovery problems include the coexistence of boron (B) and Li, where even low concentrations of B can interfere with direct lithium extraction and require a separate extraction step.^99^

We also analyzed pairs of background species that are known to pose challenges for separation and treatment technologies (Figure 7b).^31^ First, the mass ratio between SO_4_ and Ca informs the limiting species for gypsum (CaSO_4_) scale formation (Figure 7b-left). With the exception of oil and gas wastewater, most wastewaters had higher SO_4_ levels than Ca, indicating that Ca must be removed to effectively prevent scaling. Similarly, the ratio between Ca and Mg informs the dominating species for scale formation (e.g., gypsum/CaSO_4_ vs brucite/Mg­(OH)_2_). Most wastewaters reported more Ca than Mg, with the highest median mass ratio around 10:1 (Figure 7b-middle), indicating that scaling will be dominated by Ca salts and again suggesting that Ca removal would generally be an effective scaling control strategy. Finally, the chloride-to-sulfate mass ratio (sometimes abbreviated as CSMR) indicates the potential for galvanic corrosion of lead near dissimilar metals such as copper.^100^ A high Cl-to-SO_4_ mass ratio (more than 5:1) was observed in drilling and produced water in oil and gas production, tanning in manufacturing, and geothermal (Figure 7b-right), indicating a high corrosion potential if lead–metal junctions are present in the treatment system.^101^

Our analysis of background species highlights that SO_4_ commonly accounts for a large proportion of TDS and, when present alongside Ca, is usually present in excess. This last observation implies that removing Ca (or softening treatment in general) is likely to be an important step in many CM recovery processes. Our analysis of CM speciation suggests a strong dependence on wastewater chemistry and that Cl, SO_4_, and pH are likely the major factors contributing to metal complexation.

Combining the water quality parameters listed in Figure 6 and the composition of major background species in Figure 7a enables numerous, more detailed assessments of water quality and its effect on CM speciation for specific wastewater. To illustrate such an analysis, we calculated the speciation of Li, Mg, and Ni as representative monovalent, divalent, and transition metal CM ions in multiple wastewaters. We used the composition data shown in Figures 6 and 7 to define and model the chemical equilibrium of each water using pyEQL via its interface to PHREEQC.^87,102^

Figure 7c shows the Li speciation in petroleum refining and drilling wastewater. These two have similar pH (7.82 and 6.7) but different Cl concentrations (0.06 vs 59.32 mg/L) and SO_4_ concentrations (68.55 vs 0.30 mg/L). As a result, LiSO_4_^–^ is the predominant Li complex in petroleum refining wastewater, accounting for 13% of the total Li, whereas LiCl is the predominant complex in drilling wastewater, accounting for 4% of the total Li. The remaining Li is present as free Li^+^ ion.

Figure 7d shows the Mg speciation in flotation and mine drainage wastewater. These two have similar median Cl and SO_4_ levels (0.35 and 0.72 mg/L, 27.31 and 71.30 mg/L, respectively) but different pH (9.9 vs 4.0). Mg–CO_3_ complexes are observed in flotation wastewater due to its high pH and the presence of inorganic carbon, accounting for 5% of the total Mg. In both wastewaters, MgSO_4_ accounts for ∼20–40% of total Mg, indicating the stronger contribution of SO_4_ to Mg complexation compared to pH-induced carbonate complexation.

Figure 7e compares the Ni speciation in coal washing and flotation wastewaters, which have similar median SO_4_ levels (50.57 and 27.31 mg/L) but different pH values (4.2 vs 9.90). As a result of the high pH and SO_4_, Ni­(OH)_2_ and NiSO_4_ complexes were observed in flotation wastewater and account for 2% and 12% of total Ni, respectively. Both wastewaters contain a significant fraction (86–100%) of Ni as free Ni^2+^.

Altogether, these examples illustrate the importance of detailed water quality information and chemical equilibrium (speciation) modeling to inform separation process development. In the lithium case, the predominant form between two oil and gas wastewaters has an opposite charge: LiSO_4_^–^ in one case and Li^+^ in the other (Figure 7c). In the cases of Mg and Ni (Figure 7d-e), a significant fraction of the elements (roughly 1/3–1/5) is present as neutral complexes rather than free cations. Such effects could have significant implications for the expected performance of sorbents, membranes, or precipitation-based recovery technologies.

### Representative Inorganic Compositions for Research

To facilitate such detailed analysis and provide further, specific guidance to technology developers, we defined a “representative” inorganic background water quality for each of the 22 industrial activities presented, using values in Figures 6 and 7a. The resulting representative wastewater compositions are documented in Supplementary Section S2 and have also been added as “presets” to the pyEQL water chemistry library^87^ to facilitate easy access for researchers. Representative conditions of pH, salinity, and competing inorganic species are particularly important in the context of CM recovery because separation performance, such as membrane selectivity in complex, mixed-salt solutions, can be completely different from that predicted from simpler, single salt solutions.^103–106^ We emphasize (and any potential users of these representative compositions must remember) that there is tremendous variability in water quality even within a single industry, as detailed above (e.g., Figure 5 and Sections S6–S11), and that our compositions are based on the limited data that is publicly available. In addition, our compositions do not incorporate dissolved organics due to a general lack of data regarding specific constituents. Nevertheless, although the analysis we have performed here is clearly no substitute for detailed characterization of specific wastewater samples, we submit that using even an approximate synthetic wastewater (or simplified binary/ternary solutions derived from it) in the early stages of research can substantially accelerate new technology development. We hope that these compositions serve as a reference point that can speed the translation of new resource recovery technologies from the laboratory into practice.

## Discussion

In summary, we compiled and analyzed publicly available data on wastewater composition, critical mineral content, and generation volume for 22 industrial activities as a first step toward evaluating their potential as targets for valuable CM recovery. We estimate that more than 30 million tons of dissolved CMs are present in industrial wastewater, generated mainly by the oil and gas, mining, and manufacturing industries. We identified individual industrial activities that are particularly promising for wastewater valorization due to their low number and high concentrations (10–100 mg/L) of reported CMs, which include leather tanning, metal plating, lithium battery recycling, and spent leachate and waste gas treatment from mining. We also identified ubiquitous recovery challenges from a water chemistry point of view, which include low pH, brackish- to seawater-level TDS, high hardness, and the presence of problematic background species, particularly SO_4_. Overall, we show that our data set is highly valuable for identifying the value and challenges of recovering CMs from industrial wastewater.

Our analysis enabled us to estimate the total economic value ($/m^3^) of CMs dissolved in industrial wastewater based on recent market prices (Figure 2e). Wastewater from the oil and gas industry had an order of magnitude higher value ($2.82/m^3^) than the next two industries (mining and geothermal power generation; ∼$0.26/m^3^). These are upper-bound estimates of the value that could be extracted, since not all wastewater and not every CM would be economical to recover due to, factors such as, the geographic distribution of sources, competition among many similar CMs as seen in mining wastewater (Figure 5), the specific nature of organic compounds present (due to their high potency for fouling and complex interactions with CMs), practical considerations such as the need to remove naturally occurring radioactive materials (NORM) prior to valorization, the availability of power and water at a given site, and more. While analysis of these factors was beyond the scope of this study, we can nevertheless take our economic values as rough estimates of the highest feasible levelized cost of water treated that a viable recovery technology can have, as suggested by Du Chanois et al.^107^ Levelized costs of water treatment for existing and emerging ion removal technologies range from $0.20–0.40/m^3^ for brackish water capacitive deionization or reverse osmosis,^108^ $0.60/m^3^ for seawater reverse osmosis,^109^ and $5–6/m^3^ for zero liquid discharge.^110,111^ This suggests that the economic case for dedicated industrial wastewater valorization processes may be challenging unless other factors (such as the value of clean water recovered, increased resilience, reduced liability, or hazard reduction) are considered in addition to the marketable CM value. It also motivates the importance of integrating recovery technology into existing treatment or water recycling processes, which should minimize additional capital needs and carry a much lower marginal levelized cost. We believe the analysis presented here highlights the importance of detailed water quality information as a critical foundation for techno-economic analysis, which is a necessary next step for further evaluating the prospects of valorizing any of the industrial wastewaters analyzed here.

Although we believe that this analysis is informative, we must also qualify it with several important caveats. First, we considered only existing aqueous waste and focused primarily on dissolved inorganic species. Some types of solid waste, including solid mine tailings^112^ and coal combustion residuals,^113^ may contain more CMs than associated wastewater. Although recovery technologies differ from those for liquid waste, these opportunities may prove more economical than recovery from wastewater and should not be overlooked.^114^

Second, our analysis was obviously constrained by the availability and quality of detailed wastewater composition data. We want to first address the incompleteness of reported CMs. Although at least five CMs were reported for every industrial activity, we suspect that the available data do not always reflect all the CMs actually present due to factors such as a lack of reporting incentives and limitations in the detection methods used for routine or regulatory monitoring. In some cases, one can infer the likely presence of missing CMs—and therefore identify additional recovery opportunities—by considering the raw materials processed. Taking the mining industry as an example, many CMs, especially REEs, occur only in trace amounts in their host ores and are therefore rarely recovered, remaining instead in the waste.^115^ By analyzing the elemental composition of the raw ore, one could identify the CMs that are likely to be present even if they are not directly monitored. For example, certain copper sulfide ores in Nigeria contain around 0.01 wt % unrecovered Ti and Nd,^116^ which appeared only in mine drainage in our data set (Table S19–S26, Figure 5b) but should also be present in other upstream activities (e.g., flotation wastewater, leachate). Similar cases include Ge, associated with coal deposits,^117^ and In, associated with Pb–Zn ores,^118^ all of which were rarely reported in the data we analyzed.

In addition to the incompleteness of reported data, uncertainties also exist due to the age of the data. Although we included the most up-to-date data available to the best of our knowledge, some of the sources—particularly for the mining industry—date back several decades, as most measurements were originally conducted for regulatory or permitting purposes. Given advances in industrial practices (e.g., water-management strategies and technological advancements), current and future wastewater characteristics and generation rates are likely to differ from those reported historically. Using the mining industry as an example, modern water-management practices have increased the water-reuse rate from around 22% to 80% between 1973 ^36^ and 2024.^119^ Therefore, one should expect a lower wastewater generation rate for newly opened mines with advanced water management. Meanwhile, CMs present in trace amounts and not recovered during processing are likely to accumulate in the recycled water, leading to a gradual increase in their concentrations. On the other hand, advances in hydrometallurgy have improved metal-recovery efficiency in mining. For example, the recent wide adoption of electrolytic refining for Cu, high-pressure leaching for Ni,^120^ and high-temperature leaching for Pt^121^ have increased recovery efficiencies from 80% to over 99%.^122^ Therefore, for CMs that are primary recovery targets, lower concentrations can be expected in wastewater.^123^ Similar evaluations of the future CM recovery potential for each industry are documented in the **Data Limitation** sections of Supplementary Sections S6–S11.

Despite these limitations, we believe that our (necessarily approximate) analysis of industrial wastewater composition provides valuable new insights into the opportunities and challenges for CM recovery. The rapid and ongoing growth of industrial activities, represented by the hundreds of planned and newly opened metal^124–126^ and coal^127^ mines worldwide, highlights the need to expedite our efforts toward more circular wastewater management. As researchers continue to develop technologies to capture the large opportunity represented by industrial wastewater refining, we hope that this analysis will encourage the use of realistic testing conditions at the early stages of recovery technology development, thereby accelerating their optimization and implementation.

## Supplementary Material









## Data Availability

All data analyzed during this study are included in this published article and its Supporting Information files.

## Notes

Supplementary Table S15–S36­(ZIP)
Supplementary Information: Abbreviations and unit conversions (Section S1); representative inorganic wastewater composition for research (Section S2, Table S1.1–S1.4, Figure S1); list of critical mineral prices and corresponding grade, list of industries, wastewater-generating activities, and their abbreviations, and list of reported CMs elements in each subactivity (Section S3, Table S3–S4); proportion of major background species calculated from the original data source (Section S4, Figure S2); criticality rating vs dissolved mass (Figure S3); calculations of wastewater production rates and volumes (Section S5, Table S5–S14), overviews of industrial activities, data sources, and data limitations (Section S6–S11); wastewater generation schematics and concentration distributions of wastewater constituents (Figure S4–S29); maximum, minimum, and median values of wastewater generation volumes, wastewater quality parameters, and 5th–95th percentile ranges and median concentrations of wastewater constituents (Table S15–S36); annual wastewater discharge volume in the U.S. (Section S12, Table S37–S46) (PDF)
Dataset 1 Wastewater constituents: All concentration data used in our analysis and their data sources (XLSX)
Dataset 2 Wastewater production rate and volumes: All wastewater generation volume data used and calculated in our analysis and their data sources (XLSX)
