# Supplementary material for: Composition and Critical Mineral Content of Major Industrial Wastewaters: Implications for Treatment and Resource Recovery Technologies
Source: Environ Sci Technol. 2026 Aug 12;60(33):23035–53. doi: 10.1021/acs.est.6c04293 (PMC13523687; doi:10.1021/acs.est.6c04293)
Supplement: Supplementary file 1 [file es6c04293_si_002.pdf]

Supplementary Information

for

Composition and Critical Mineral Content of Major Industrial Wastewaters: Implications for Treatment and Resource Recovery Technologies

Monong Wang[a], Jaebeom Park[a,b], Sui Xiong Tay[a,c], Vineet Bansal[d], Emily J. Rabe [e], Ryan S. Kingsbury [a,b]*

[a] Andlinger Center for Energy and the Environment, Princeton University, Princeton, New Jersey 08540, United States

[b] Department of Civil and Environmental Engineering, Princeton University, Princeton, New Jersey 08540, United States

[c] Department of Mechanical and Aerospace Engineering, Princeton University, Princeton, New Jersey 08540, United States

[d] Research Computing, Princeton University, New Jersey 08540 USA

[e] Membrion, Inc., Seattle, Washington, 98119, United States

This Supplementary Information contains the following items from page S1 to S122

- Section S1 to Section S12
- Figure S1 to Figure S31
- Table S1 (S1.1–S1.4) to Table S46
- References

[*] The author to whom correspondence should be addressed. E-mail: kingsbury@princeton.edu.

Summary of Contents

Section S1. Abbreviations and unit conversions 4

S1.1 Abbreviations 4

S1.2 Unit conversions 4

S1.3 Conversion of COD and BOD to Total Organic Carbon (TOC) 4

Section S2. Representative Inorganic Compositions for Research 5

S2.1 Step-by-step procedure for deriving representative inorganic composition 5

S2.2 Example code illustrating how to retrieve representative inorganic compositions using pyEQL 9

S2.3 Representative inorganic composition retried from pyEQL in their salt form 10

Section S3. Supplementary Tables 16

Section S4. Supplementary Figures 20

Section S5. Calculation of Annual Wastewater Volumes 21

S5.1 Oil and gas production annual wastewater volume 21

S5.2 Metal and coal mining annual wastewater volume 23

S5.3 Manufacturing annual wastewater volume 25

S5.4 Fossil fuel power generation annual wastewater volume 26

S5.5 Geothermal produced wastewater volume 27

S5.6 Gasification annual wastewater volume 28

Section S6. Oil and Gas Production 29

S6.1 Industry description 29

S6.2 Overview of oil and gas production wastewater streams 29

S6.3 Drilling wastewater 30

S6.4 Produced water 32

S6.5 Petroleum refining 39

S6.6 Data sources 40

S6.7 Data limitations 41

Section S7. Metal Ore and Coal Mining 42

S7.1 Industry description 42

S7.2 Overview of metal mining wastewater streams 43

S7.3 Excavation wastewater 43

S7.4 Metal concentrating wastewater–milling and flotation 45

S7.5 Metal concentrating wastewater–spent leachate 48

S7.6 Smelting and refining wastewater 50
S7.7 Waste gas treatment wastewater 52
S7.8 Overflow wastewater from tailings ponds 54
S7.9 Abandoned / acid mine drainage (AMD) 56
S7.10 Coal washing 58
S7.11 Data sources 59
S7.12 Data limitations 60
Section S8. Manufacturing 61
S8.1 Semiconductor manufacturing wastewater 61
S8.2 Leather tanning wastewater 66
S8.3 Metal plating and finishing wastewater 68
S8.4 Battery manufacturing wastewater 69
S8.5 Lithium battery (LIB) recycling wastewater 72
S8.6 Data sources 74
S8.7 Data limitations 75
Section S9. Fossil Fuel Power Generation 76
S9.1 Industry description 76
S9.2 Overview of fossil fuel wastewater streams 76
S9.3 Flue gas desulfurization (FGD) wastewater 76
S9.4 Bottom ash transport water 79
S9.5 Combustion residual leachate 81
S9.6 Data sources 82
S9.7 Data limitations 83
Section S10. Geothermal Power Generation 84
S10.1 Industry description 84
S10.2 Overview of geothermal wastewater streams 84
S10.3 Data sources 87
S10.4 Data limitations 87
Section S11. Gasification 88
S11.1 Industry description 88
S11.2 Overview of gasification wastewater streams 88
S11.3 Data sources 90
S11.4 Data limitations 90

Section S12. Treated Wastewater volume and CM content 92
S12.1 Oil and gas extraction wastewater discharge volume and CM content 93
S12.2 Petroleum refining wastewater discharge volume and CM content 94
S12.3 Ore mining and dressing wastewater discharge volume and CM content 95
S12.4 Coal mining wastewater discharge volume and CM content 96
S12.5 Semiconductor manufacturing wastewater discharge volume and CM content 97
S12.6 Leather tanning and finishing wastewater discharge volume and CM content 98
S12.7 Metal plating wastewater discharge volume and CM content 99
S12.8 Battery manufacturing wastewater discharge volume and CM content 100
S12.9 Steam electric power generation wastewater discharge volume and CM content 101
References 102

Section S1. Abbreviations and unit conversions

S1.1 Abbreviations

40 CFR – title 40 of the U.S. code of federal regulations
ANL – Argonne National Laboratory
API – American Petroleum Institute
CM – Critical mineral
CPinfo – Combustion product information
DMR – Discharge Monitoring Report Database (2024)
DOE – U.S. Department of Energy
EIA – U.S. Energy Information Administration
EPA – U.S. Environmental Protection Agency
EPRI – Electrical Power Research Institute
IEA – International Energy Agency
NETL – National Energy Technology Laboratory
NEWTS – National Energy Water Treatment and Speciation
NREL – National Renewable Energy Laboratory
SIC – Standard Industrial Classification
SS – Suspended solids
TDS – Total dissolved solids
TIC – Total inorganic carbon
TIN – Total inorganic nitrogen
TOC – Total organic carbon
USGS – U.S. Geological Survey

S1.2 Unit conversions

Metric tonne (ton) = 1,000 kilograms (kg)
Million tons (Mt) = 1,000,000 metric tons
Parts per thousand (ppth) = 1,000 parts per million (ppm) =1,000,000 parts per trillion (ppt)
Million cubic meters (Mm^3) = 1,000,000 m^3
Million gallons (Mgal) = 0.003785 Mm^3

S1.3 Conversion of COD and BOD to Total Organic Carbon (TOC)

We adapted the conversion from COD and BOD to TOC using the relationship described by Dubber et al.[1] Note that this equation was developed based on domestic and municipal wastewater data, therefore may not accurately reflect the relationship for certain industrial wastewater.

$$TOC = \frac{COD - 49.2}{3}$$

$$TOC = \frac{BOD_5 - 23.7}{1.68}$$

Where TOC (mg/L) is the total organic carbon, COD (mg/L) is the chemical oxygen demand, and BOD5 (mg/L) is the 5-day biochemical oxygen demand.

Section S2. Representative Inorganic Compositions for Research

S2.1 Step-by-step procedure for deriving representative inorganic composition

The “representative inorganic composition” for wastewater is an electroneutral composition derived from dissolved inorganic compounds reported in our data sources. For the wastewater from each industrial activity, the composition was derived according to the following procedure:

1. Calculate the median concentrations of inorganic solutes and the median pH for each industrial activity.
2. Exclude any solute whose proportion of the TDS was lower than 10^{-3}. This step eliminates trace species and is necessary to facilitate the chemical equilibrium calculations.
3. Calculate the charge (im)balance
4. Balance the charge with one of the cations or anions listed in **Table S1.1**.
5. Use the pyEQL wrapper to the USGS PHREEQC software (pyEQL-PHREEQC)[2,3] with the llnl.dat thermodynamic database to chemically equilibrate the composition at the median pH (see an example for Waste Gas Treatment wastewater before and after equilibration in **Table S1.3**).
6. In cases where multiple balancing ions were used (e.g., in Waste Gas Treatment wastewater, both Na^+ and Ca^{2+} can be the charge balancing cation), steps 4-5 were repeated for each ion. A median composition was then calculated and re-equilibrated using the dominant charge-balancing ion following step 7 (see the Waste Gas Treatment wastewater example in **Table S1.4**)
7. Calculate the TDS by summing all solutes after equilibration
8. Re-equlibrate and ensure that the final equilibrated pH matches the median pH of the industry.
9. Calculate the proportion of each element in total TDS by dividing the total element concentration in the equilibrated composition from Step 7 by the calculated TDS. These are the values reported in Figure 7 of the main text.

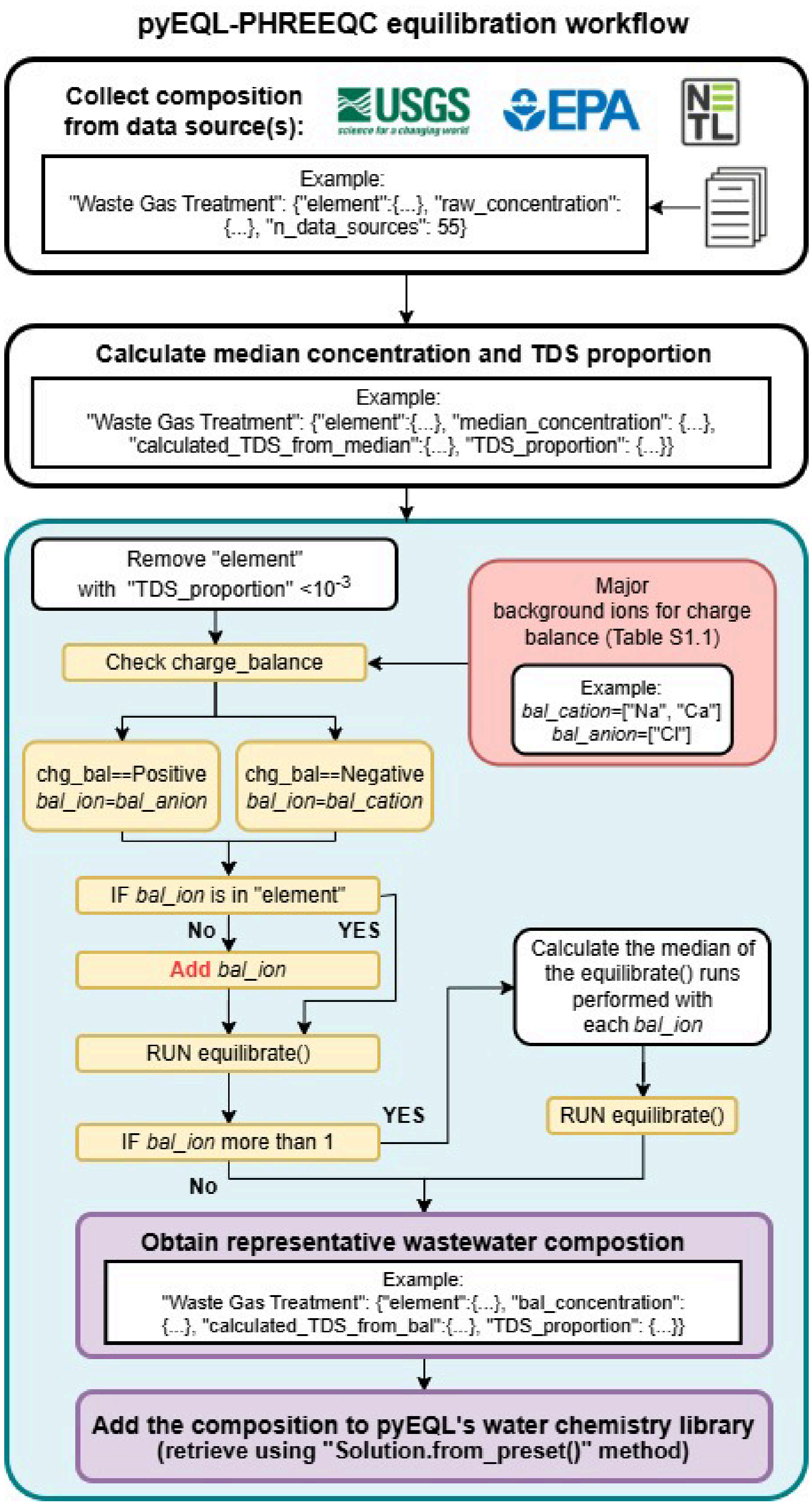

Figure S1: Steps to develop representative inorganic compositions included in the pyEQL library.

Table S1.1. Charge-balancing ions for wastewater from major industrial activities.

Main Industry	Sub Activities	Major Background Cations	Major Background Anions
Oil and Gas Production	Drilling	Na^+	Cl^-
	Produced Water from Conventional Production	Na^+	Cl^-
	Produced Water from Unconventional Production	Na^+	Cl^-
	Petroleum Refining	Na^+	SO_4^{2-}
Metal Ore and Coal Mining	Excavation	Ca^{2+}	SO_4^{2-}
	Milling and Flotation	Na^+	SO_4^{2-}
	Smelting and Refining	Ca^{2+}	SO_4^{2-}
	Waste Gas Treatment*	Na^+ or Ca^{2+}	SO_4^{2-}
	Spent Leachate	Ca^{2+} or Al^{3+} or Fe^{3+}	SO_4^{2-}
	Mine Drainage	Ca^{2+}	SO_4^{2-}
	Tailing	Ca^{2+}	SO_4^{2-}
	Coal Washing	Ca^{2+} or Mg^{2+}	SO_4^{2-}
Manufacturing	Semiconductor Manufacturing	Ca^{2+}	F^- or SO_4^{2-}
	Leather Tanning	Na^+	Cl
	Plating	Na^+ or Al^{3+}	SO_4^{2-}
	Battery Manufacturing**	Na^+ or K^+	SO_4^{2-}
	Lithium Battery Recycling	Na^+	F^- or SO_4^{2-}
Fossil Fuel Power Generation	Flue Gas Desulfurization	Ca^{2+} or Mg^{2+}	Cl^-
	Ash Transport	Ca^{2+}	SO_4^{2-}
	Combustion Residual Leachate	Ca^{2+}	SO_4^{2-}
Geothermal	N/A	Na^+	Cl^-
Gasification	N/A	Na^+	SO_4^{2-}

* Na^+ and Ca^{2+} were not found in reported data. However, lime, soda lime, and lime stone are commonly used as gas scrubber materials, therefore contributing to Na^+ or Ca^{2+} in wastewater.[4–7]
** Na^+ and K^+ were not found in reported data. However, electrolytes for alkaline batteries often contain sodium and potassium hydroxide.[8–10]

Table S1.2. Keywords for representative inorganic compositions in pyEQL.

Main Industry	Sub Activities	pyEQL 'preset' keyword
Oil and Gas Production	Drilling	`drilling`
	Produced Water from Conventional Production	`pw_conv`
	Produced Water from Unconventional Production	`pw_unconv`
	Petroleum Refining	`refining`
Metal Ore and Coal Mining	Excavation	`excavation`
	Milling and Flotation	`flotation`
	Smelting and Refining,	`smelting`
	Waste Gas Treatment	`waste_gas`
	Spent Leachate	`leachate`
	Mine Drainage	`mine_drainage`
	Tailing	`mine_tailings`
	Coal Washing	`coal_washing`
Manufacturing	Semiconductor Manufacturing	`semiconductor`
	Leather Tanning	`tanning`
	Plating	`plating`
	Battery Manufacturing	`batt_mfg`
	Lithium Battery Recycling	`batt_recycling`
Fossil Fuel Power Generation	Flue Gas Desulfurization	`FGD`
	Ash Transport	`ash`
	Combustion Residual Leachate	`CRL`
Geothermal	N/A	`geothermal`
Gasification	N/A	`gasification`

Table S1.3. Example: Waste Gas Treatment wastewater constituent and quality before and after pyEQL equilibration. (A) Median concentration and proportion of raw Gas Scrubber data. (B)–(C) pyEQL-PHREEQC equilibrated concentration and proportion using representative charge-balancing species, (B) Ca^{2+} and (C) Na^+.

**(A) Composition from a raw dataset[5]**			**(B) Composition after equilibration with Ca^{+2}**			**(C) Composition after equilibration with Na^+**		
**Species**	**Concentration (mg/L)**	**Proportion in TDS (%)**	**Species**	**Concentration (mg/L)**	**Proportion in TDS (%)**	**Species**	**Concentration (mg/L)**	**Proportion in TDS (%)**
SO_4^{2-}	32428.04	98.81	$CaSO_4$(aq)	22426.7	44.44	SO_4^{2-}	20038.69	41.60
As	17.40	0.49	SO_4^{2-}	15885.6	34.36	Na^+	12458.99	25.86
Zn	149.55	0.46	Ca^{2+}	6710.4	14.51	$NaSO_4^-$	14156.76	29.39
Pb	79.22	0.24	HSO_4^-	690.2	1.49	HSO_4^-	837.56	1.74
			$As(OH_2)_2^-$	252.7	0.55	$As(OH_2)_2^-$	252.92	0.53
			$ZnSO_4$ (aq)	215.7	0.47	$ZnSO_4$ (aq)	230.21	0.48
			Zn^{2+}	62.1	0.13	Pb^{+2}	79.22	0.16
			Pb^{2+}	79.2	0.17	Zn^{+2}	56.25	0.12
TDS	32818.43	100	TDS	46250.79	96.12	TDS	48171.93	99.88
pH	2.8		pH	2.76		pH	2.76	
Charge balance (Eq/L)	-6.62×10^{-1}		Charge balance (Eq/L)	-1.23×10^{-09}		Charge balance (Eq/L)	-1.19×10^{-09}	

Table S1.4. Example: representative inorganic composition for Waste Gas Treatment wastewater. (A) Median of pyEQL-PHREEQC equilibrated concentration and proportion with Na^+ and Ca^{2+} (values in Table S1.3(B)–(C)). (B) Final reported representative inorganic composition calculated by equilibrating the median in (A) using pyEQL-PHREEQC.

**(A) Median of equilibrated proportions**			**(B) Representative proportion after equilibration**		
**Species**	**Median concentration (mg/L)**	**Median proportion TDS (%)**	**Species**	**Median concentration (mg/L)**	**Median proportion TDS (%)**
SO_4^{2-}	32453.81	68.71	SO_4^{2-}	32453.81	68.71
As^{3+}	0.0474	1×10^{-4}	As^{3+}	0.0475	1×10^{-4}
Ca^{2+}	6640.58	14.06	Ca^{2+}	6640.58	14.06
Na^+	7601.05	16.09	Na^+	7601.05	16.09
Zn^{2+}	149.64	0.32	Zn^{2+}	149.64	0.32
As^{5+}	161.66	0.34	As^{5+}	161.66	0.34
Pb^{2+}	79.27	0.17	Pb^{2+}	79.272	0.17
TDS (mg/L)	47223.26	99.69	TDS (mg/L)	47233.23	99.69
pH	2.76		pH	2.67	
Charge balance (Eq/L)	6.78×10^{-3}		Charge balance (Eq/L)	-1.22×10^{-09}	

S2.2 Example code illustrating how to retrieve representative inorganic compositions using pyEQL

`pyEQL`[11] is a python library for water chemistry calculations, offering a suite of engineering models and utilities designed to facilitate environmental engineering research. It provides features such as an intuitive interface for creating and manipulating solutions, a built-in database containing physicochemical properties for hundreds of dissolved species, methods for estimating properties of complex, multicomponent electrolytes such as osmotic pressure, transference numbers, and electrical conductivity, automatic unit conversion, and the ability to perform chemical equilibrium (speciation) calculations via a wrapper to PHREEQC.

First, install `pyEQL`[11] according to the documentation at https://pyeql.readthedocs.io/.

Next, execute the following code in a python interpreter. Specify the desired industrial activity as an argument to the from_preset() method. The arguments associated with each activity are listed in **Table S1.2.**

```
>>> from pyEQL import Solution
>>> s1 = Solution.from_preset('pw_unconv')
```

Various methods are available to access properties of the resulting Solution object stored in variable s1, such as concentrations, density, pH, hardness, etc. Refer to the online documentation for further details.

To view an example "recipe: for how to prepare a solution from salts, call the `get_salt_dict()` method as shown below. This will print a list of the molar concentrations of the dominant salts in the composition.

```
>>> from pprint import pprint
>>> pprint({k: v['mol'] for k,v in s1.get_salt_dict().items()})

{'NaCl': 0.10888,

'CaCl2': 0.0279,

 'SrCl2': 0.0223,

 'LiCl': 0.00147 }
```

S2.3 Representative inorganic composition retried from pyEQL in their salt form

Oil and Gas Production

1. drilling: pH=6.99 TDS=23570.20 mg/L

```
==========================================
 NaCl      :  0.35213 mol/L
 CaCl2     :  0.00726 mol/L
 MgCl2     :  0.00703 mol/L
 KCl       :  0.00665 mol/L
 LiBr      :  0.00273 mol/L
 SrCl2     :  0.00262 mol/L
 LiNO3     :  0.00218 mol/L
 Li2SO4    :  0.00043 mol/L
 SrBr2     :  0.00031 mol/L
 BaSO4     :  0.00031 mol/L
 Ba(HCO3)2 :  0.00006 mol/L
```

2. pw_conv: pH=7.17 TDS=59816.83 mg/L

```
=========================================
 NaCl      :  0.88041 mol/L
 CaCl2     :  0.04967 mol/L
 MgCl2     :  0.01706 mol/L
 MgSO4     :  0.00318 mol/L
 K2SO4     :  0.00224 mol/L
 SrBr2     :  0.00160 mol/L
 Sr(HCO3)2 :  0.00011 mol/L
 SrSO4     :  0.00015 mol/L
```

3. pw_unconv: pH=7.57 TDS=7259.10 mg/L

```
==========================================
 NaCl      :  0.10868 mol/L
 CaCl2     :  0.00278 mol/L
 SrCl2     :  0.00222 mol/L
 LiCl      :  0.00147 mol/L
 MgSO4     :  0.00047 mol/L
 KHCO3     :  0.00027 mol/L
 MgCl2     :  0.00021 mol/L
```

KIO3 : 0.00018 mol/L
KBr : 0.00015 mol/L
Mg(HCO3)2 : 0.00004 mol/L

4. refining: pH=7.65 TDS=4959.77 mg/L

==

CaSO4 : 0.01588 mol/L
Na2SO4 : 0.01408 mol/L
K2SO4 : 0.00090 mol/L
BaSO4 : 0.00078 mol/L
SrSO4 : 0.00005 mol/L

Metal Ore and Coal Mining

5. excavation: pH=7.67 TDS=451.82 mg/L

==

CaSO4 : 0.00240 mol/L
NaNO3 : 0.00048 mol/L
Ca(NO3)2 : 0.00020 mol/L
Mg(HCO3)2 : 0.00014 mol/L
MgCl2 : 0.00008 mol/L
KCl : 0.00006 mol/L
NaHCO3 : 0.00004 mol/L
ZnCl2 : 0.00002 mol/L
MnCl2 : 0.00001 mol/L

6. flotation: pH=9.11 TDS=3529.57 mg/L

==

NaHCO3 : 0.01158 mol/L
Na2SO4 : 0.00600 mol/L
NaNO3 : 0.00033 mol/L
NaCl : 0.00031 mol/L
Na2HPO4 : 0.00012 mol/L
Na2Cu(CO3)2: 0.00005 mol/L
Na2MoO4 : 0.00003 mol/L
Na2WO4 : 0.00003 mol/L
NaOH : 0.00002 mol/L

7. leachate: pH=1.45 TDS=36808.38 mg/L

==

H2SO4 : 0.05089 mol/L
Fe(HSO4)3 : 0.02150 mol/L
Zn(HSO4)2 : 0.00170 mol/L
Cu(HSO4)2 : 0.00134 mol/L
Ni(HSO4)2 : 0.00039 mol/L
Co(HSO4)2 : 0.00039 mol/L

8. smelting: pH=7.68 TDS=616.99 mg/L

====================================

CaSO4 : 0.00369 mol/L
MgCl2 : 0.00055 mol/L
NaHCO3 : 0.00020 mol/L
MgSO4 : 0.00013 mol/L
Mg(HCO3)2 : 0.00007 mol/L
Zn(HCO3)2 : 0.00001 mol/L

9. waste_gas: pH=2.67 TDS=47233.23 mg/L

=======================================

Na2SO4 : 0.16531 mol/L
H2SO4 : 0.00106 mol/L
PbSO4 : 0.00038 mol/L

10. mine_drainage: pH=4.02 TDS=1796.58 mg/L

==

CaSO4 : 0.00724 mol/L
MgSO4 : 0.00536 mol/L
Na2SO4 : 0.00068 mol/L
KCl : 0.00015 mol/L
HCl : 0.00011 mol/L
MnSO4 : 0.00010 mol/L
Fe(OH)2Cl : 0.00003 mol/L
MnCl2 : 0.00001 mol/L

11. mine_tailings: pH=7.54 TDS=4866.39 mg/L

==

CaSO4 : 0.02144 mol/L
Na2SO4 : 0.00971 mol/L
MgCl2 : 0.00391 mol/L
MgSO4 : 0.00050 mol/L
KHCO3 : 0.00032 mol/L
Mg(NO3)2 : 0.00035 mol/L
KF : 0.00033 mol/L
KNO3 : 0.00024 mol/L

12. coal_washing: pH=4.12 TDS=1314.55 mg/L

===

CaSO4 : 0.00531 mol/L
MgCl2 : 0.00214 mol/L
MgSO4 : 0.00141 mol/L
NaCl : 0.00110 mol/L
KCl : 0.00032 mol/L
PbCl2 : 0.00018 mol/L
FeHPO4Cl : 0.00012 mol/L

AlHPO4Cl : 0.00010 mol/L
HCl : 0.00008 mol/L
CdCl2 : 0.00007 mol/L
ZnCl2 : 0.00006 mol/L
CuCl2 : 0.00003 mol/L
MnCl2 : 0.00002 mol/L

Manufacturing

13. semiconductor: pH=7.37 TDS=671.10 mg/L

==

CaSO4 : 0.00233 mol/L
NaCl : 0.00153 mol/L
Na2SO4 : 0.00035 mol/L
KF : 0.00025 mol/L
KCl : 0.00014 mol/L
Mg(HCO3)2 : 0.00009 mol/L

14. plating: pH=3.16 TDS=663.30 mg/L

==================================

Na2SO4 : 0.00299 mol/L
NiSO4 : 0.00041 mol/L
ZnSO4 : 0.00039 mol/L
H2SO4 : 0.00039 mol/L
CaSO4 : 0.00008 mol/L
MgSO4 : 0.00007 mol/L
CuSO4 : 0.00005 mol/L
K2SO4 : 0.00005 mol/L
AlF3 : 0.00002 mol/L
CdSO4 : 0.00001 mol/L

CdF2 : 0.00001 mol/L
PbF2 : 0.00001 mol/L

15. tanning: pH=7.88 TDS=5793.92 mg/L

==================================

NaCl : 0.06776 mol/L
KCl : 0.00538 mol/L
K2SO4 : 0.00477 mol/L
Mg(NO3)2 : 0.00126 mol/L
MgSO4 : 0.00103 mol/L
Ca(NO3)2 : 0.00036 mol/L
Ca(HCO3)2 : 0.00001 mol/L

16. batt_mfg: pH=7.66 TDS=37.62 mg/L

==================================

Ca(HCO3)2 : 0.00012 mol/L
Mg(HCO3)2 : 0.00004 mol/L
NaCl : 0.00002 mol/L
MgCl2 : 0.00001 mol/L
Na2SO4 : 0.00001 mol/L

17. batt_recycling: pH=6.73 TDS=165486.97 mg/L
==
LiF : 1.34851 mol/L
CoF2 : 0.17137 mol/L
Cr3(OH)4F5: 0.14796 mol/L
CoSO4 : 0.12892 mol/L
(FeF2)2SO4: 0.07373 mol/L
NiSO4 : 0.06646 mol/L
Na2SO4 : 0.00429 mol/L
(CaF)2SO4 : 0.00152 mol/L
(CuF)2SO4 : 0.00110 mol/L

Fossil Fuel Power Generation

18. ash: pH=7.99 TDS=256.36 mg/L
==============================
CaSO4 : 0.00103 mol/L
NaHCO3 : 0.00033 mol/L
Na2SO4 : 0.00024 mol/L
Mg(HCO3)2 : 0.00017 mol/L
KCl : 0.00014 mol/L
KAlO2 : 0.00003 mol/L
KHCO3 : 0.00002 mol/L

19. CRL: pH=7.45 TDS=2006.91 mg/L
===============================
CaSO4 : 0.01073 mol/L
Na2SO4 : 0.00304 mol/L
MgCl2 : 0.00077 mol/L
NaCl : 0.00023 mol/L
Mg(HCO3)2 : 0.00011 mol/L

20. FGD: pH=6.11 TDS=17507.38 mg/L
================================
MgCl2 : 0.12195 mol/L
CaCl2 : 0.02768 mol/L
CaBr2 : 0.00045 mol/L
Ca(HCO3)2 : 0.00002 mol/L

Geothermal

21. geothermal: pH=7.26 TDS=1844.64 mg/L

==

NaCl	: 0.02589 mol/L
K2SO4	: 0.00075 mol/L
CaSO4	: 0.00029 mol/L
LiHCO3	: 0.00015 mol/L
KCl	: 0.00015 mol/L
Li2SO4	: 0.00008 mol/L
LiF	: 0.00006 mol/L
Mg(NO3)2	: 0.00004 mol/L
MgF2	: 0.00004 mol/L

Gasification

22. gasification: pH=8.74 TDS=8313.81 mg/L

==

NaCl	: 0.04064 mol/L
Na2SO4	: 0.03552 mol/L
NaHCO3	: 0.00522 mol/L
NaNO3	: 0.00433 mol/L
NaOH	: 0.00001 mol/L

Section S3. Supplementary Tables

Table S2. List of critical mineral prices and corresponding grade. Prices for elements except Uranium, Praseodymium, and Samarium, were obtained from USGS 2025 Mineral Commodity Summaries report.[12] The price for Uranium, Praseodymium, and Samarium were not found in the report, and online resources were used to obtain the price.[13–15]

Element	Price ($/g-element)	Commercial Form
Al	0.00287	metal (primary aluminum)
Cu	0.00937	metal (refined cathode copper)
Pb	0.00243	metal (refined lead)
Ni	0.017	metal (primary nickel)
Zn	0.00317	metal (slab zinc)
Sn	0.0309	metal (refined tin)
Au	77.2	metal (refined bullion)
Ag	0.89	metal (refined)
Pt	30.5	metal (refined)
Pd	31.5	metal (refined)
Rh	148	metal (refined)
Ir	154	metal (refined)
Ru	14.1	metal (refined)
Co	0.0375	metal (refined cobalt)
Li	0.014	compound (lithium carbonate equivalent)
Mn	0.0058	ore/ferroalloy (Mn content basis)
V	0.0214	compound (vanadium pentoxide, V_2O_5)
Mg	0.00772	metal (primary magnesium)
Sb	0.0209	metal (antimony ingot)
As	0.00441	metal (arsenic)
Bi	0.0117	metal (bismuth)
Cd	0.0041	metal (cadmium)
Cr	0.00397	alloy (ferrochrome / charge chrome)
Ga	0.5	metal (refined gallium)
Ge	2.1	metal (refined germanium)
In	0.34	metal (refined indium)
Mo	0.047	compound (molybdenum oxide, MoO_3)
Nb	0.026	alloy (ferroniobium)
Ta	0.17	compound (tantalum pentoxide, Ta_2O_5 concentrate)
Te	0.075	metal (refined tellurium)
Tl	9.5	metal (refined thallium)
Ti	0.013	metal (titanium sponge)
W	0.025	compound (tungsten oxide, WO_3)
Y	0.033	metal (yttrium)
Zr	0.025	metal (zirconium)
Hf	4.6	metal (hafnium)

Ce	0.001	oxide (cerium oxide, CeO_2)
La	0.001	oxide (lanthanum oxide, La_2O_3)
Nd	0.056	oxide (neodymium oxide, Nd_2O_3)
Dy	0.26	oxide (dysprosium oxide, Dy_2O_3)
Tb	0.81	oxide (terbium oxide, Tb_4O_7)
F	0.00047	mineral (fluorspar, acid-grade CaF_2)
Si	0.00397	metal (silicon metal) / alloy (ferrosilicon)
U	0.1234	compound (U_3O_8 equivalent)
Pr	0.10734	oxide (praseodymium oxide, Pr_2O_3)
Ba	0.00022	mineral (barite, $BaSO_4$)
Sm	0.0125	oxide (samarium oxide, Sm_2O_3)
Be	1.5	alloy (beryllium-copper master alloy, contained Be basis)

Table S3. List of industries, wastewater-generating activities, and their abbreviations.

Industry	CFR Parts Referenced	Sub Activities	Abbreviation
Oil and Gas Production	Oil and Gas Production (435)	Drilling	N/A
		Produced Water from Conventional Production	PW Conv
		Produced Water from Unconventional Production	PW Unconv
	Petroleum Refining (419)	Petroleum Refining	N/A
Metal Ore and Coal Mining	Ore Mining and Dressing (440), Coal Mining (434)	Excavation	N/A
		Milling and Flotation	Flotation
		Spent Leachate	Leachate
		Smelting and Refining	Smelting & Refining
		Waste Gas Treatment	Gas Scrubber
		Tailing	N/A
		Mine Drainage	N/A
		Coal Washing	N/A
Manufacturing	Electrical and Electronic Components (469)	Semiconductor Manufacturing	Semiconductor
	Leather Training and Finishing (425)	Leather Tanning	Tanning
	Metal finishing (433)	Plating	N/A
	Battery Manufacturing(461)	Battery Manufacturing	N/A
		Lithium Battery Recycling	Battery Recycling
Fossil Fuel Power Generation	Steam Electric Power Generating (423)	Flue Gas Desulfurization	FGD
		Ash Transport	Ash
		Combustion Residual Leachate	CRL
Geothermal Power Generation	N/A	N/A	Geothermal
Gasification	N/A	N/A	N/A

Table S4. CMs reported in wastewater from different activities.

Industry	Sub Activities	CMs reported in data	CMs not reported in data	Number of CMs in data
Oil and Gas Production	Drilling	Mg, Al, F, Si, Co, U, Cu, Sb, V, Ti, Zn, Cr, As, Ba, Li, Mn, Ni, Be, Zr	Ge, Y, Pd, La, Tb, In, Sn, Ru, Dy, Rh, Ir, Hf, Nb, Ta, Sm, Te, Pt, Ga, Bi, Nd, Pr, W, Ce	19
	Produced Water (Conventional)	Mg, Al, F, Si, Ga, Tb, Co, Nd, Rh, Dy, U, Cu, Pr, Ce, La, Y, Sb, Hf, V, Sn, Ge, Zn, Cr, As, Ba, Li, Mn, Sm, Ni, Be, Zr	Pd, In, Ru, Ir, Ti, Nb, Ta, Te, Pt, Bi, W	31
	Produced Water (Unconventional)	Mg, Al, F, Si, Tb, Co, Nd, Dy, U, Cu, Pr, Ce, La, Bi, Sb, V, Sn, Ti, Zn, Cr, As, Ba, Li, Mn, Sm, Ni, Be, Zr	Ge, Y, Pd, In, Ru, Rh, Ir, Hf, Nb, Ta, Te, Pt, Ga, W	28
	Petroleum Refinery	F, Cu, Zn, Cr, As	Ge, Y, Pd, La, Si, Tb, Ni, Ba, In, Sn, Ru, Dy, Rh, Ir, Mg, Hf, Ti, Zr, Nb, Ta, Sm, Te, U, Pt, Ga, Co, Sb, Bi, Nd, Li, Pr, Mn, V, W, Al, Be, Ce	5
Metal Ore and Coal Mining	Excavation	Mg, F, Co, Cu, Sb, Zn, Cr, As, Mn, Te, Ni, Be	Ge, Y, Pd, La, Si, Tb, Ba, In, Sn, Ru, Dy, Rh, Ir, Hf, Ti, Zr, Nb, Ta, Sm, U, Pt, Ga, Bi, Nd, Li, Pr, V, W, Al, Ce	12
	Milling and Flotation	Mg, Al, F, Si, Co, Cu, Sb, W, Zn, Cr, As, Mn, Ni, Be	Ge, Y, Pd, La, Tb, Ba, In, Sn, Ru, Dy, Rh, Ir, Hf, Ti, Zr, Nb, Ta, Sm, Te, U, Pt, Ga, Bi, Nd, Li, Pr, V, Ce	14
	Spent Leachate	Mg, Al, Co, Cu, Zn, Cr, As, Ni, Be	F, Ge, Y, Pd, La, Si, Tb, Ba, In, Sn, Ru, Dy, Rh, Ir, Hf, Ti, Zr, Nb, Ta, Sm, Te, U, Pt, Ga, Sb, Bi, Nd, Li, Pr, Mn, V, W, Ce	9
	Smelting and Refining	Mg, Al, F, Cu, Sb, Sn, Zn, Cr, As, Te, Ni, Be	Ge, Y, Pd, La, Si, Tb, Ba, In, Ru, Dy, Rh, Ir, Hf, Ti, Zr, Nb, Ta, Sm, U, Pt, Ga, Co, Bi, Nd, Li, Pr, Mn, V, W, Ce	12
	Waste Gas Treatment	Cu, Zn, As, Te, Ni	F, Ge, Y, Pd, La, Si, Tb, Ba, In, Sn, Ru, Dy, Rh, Ir, Mg, Hf, Ti, Zr, Nb, Ta, Sm, U, Pt, Cr, Ga, Co, Sb, Bi, Nd, Li, Pr, Mn, V, W, Al, Be, Ce	5
	Tailing	Mg, Al, F, Co, U, Cu, Sb, V, Zn, Cr, As, Ba, Mn, Ni, Be	Ge, Y, Pd, La, Si, Tb, In, Sn, Ru, Dy, Rh, Ir, Hf, Ti, Zr, Nb, Ta, Sm, Te, Pt, Ga, Bi, Nd, Li, Pr, W, Ce	15
	Mine Drainage	Mg, Al, F, Si, Ga, Tb, Nb, Co, Nd, Dy, U, Cu, Pr, Ce, La, Y, Sb, Ta, Hf, W, V, Sn, Ge, Pd, Ti, Zn, Cr, As, Ba, In, Li, Mn, Sm, Te, Ni, Be, Zr	Bi, Ru, Rh, Ir, Pt	37
Geothermal Power Generation	N/A	Mg, Al, F, Si, Co, Cu, Bi, Sb, W, V, Ti, Zn, Cr, As, Ba, Li, Mn, Ni, Be	Ge, Y, Pd, La, Tb, In, Sn, Ru, Dy, Rh, Ir, Hf, Zr, Nb, Ta, Sm, Te, U, Pt, Ga, Nd, Pr, Ce	19
Fossil Fuel Power Generation	Fuel Gas Desulfurization	Mg, Al, F, Si, Co, Cu, Sb, V, Ti, Zn, Cr, As, Ba, Li, Mn, Ni, Be	Ge, Y, Pd, La, Tb, In, Sn, Ru, Dy, Rh, Ir, Hf, Zr, Nb, Ta, Sm, Te, U, Pt, Ga, Bi, Nd, Pr, W, Ce	17
	Ash Transport	Mg, Al, F, Si, Co, Cu, Sb, V, Ti, Zn, Cr, As, Ba, Mn, Ni, Be	Ge, Y, Pd, La, Tb, In, Sn, Ru, Dy, Rh, Ir, Hf, Zr, Nb, Ta, Sm, Te, U, Pt, Ga, Bi, Nd, Li, Pr, W, Ce	16
	Combustion Residual Leachate	Mg, Al, Co, Cu, Sb, V, Sn, Ti, Zn, Cr, As, Ba, Mn, Ni, Be	F, Ge, Y, Pd, La, Si, Tb, In, Ru, Dy, Rh, Ir, Hf, Zr, Nb, Ta, Sm, Te, U, Pt, Ga, Bi, Nd, Li, Pr, W, Ce	15
Gasification	N/A	Mg, Al, Cu, Sb, V, Ti, Zn, Cr, As, Ba, Mn, Ni, Be	F, Ge, Y, Pd, La, Si, Tb, In, Sn, Ru, Dy, Rh, Ir, Hf, Zr, Nb, Ta, Sm, Te, U, Pt, Ga, Co, Bi, Nd, Li, Pr, W, Ce	13
Manufacturing	Semiconductor Manufacturing	Mg, Al, F, Co, Pt, Cu, Sb, V, Sn, Ti, Zn, Cr, As, Ba, Mn, Te, Ni, Be	Ge, Y, Pd, La, Si, Tb, In, Ru, Dy, Rh, Ir, Hf, Zr, Nb, Ta, Sm, U, Ga, Bi, Nd, Li, Pr, W, Ce	18
	Leather Tanning	Mg, Al, Cu, Zn, Cr, Mn, Ni	F, Ge, Y, Pd, As, La, Si, Tb, Ba, In, Sn, Ru, Dy, Rh, Ir, Hf, Ti, Zr, Nb, Ta, Sm, Te, U, Pt, Ga, Co, Sb, Bi, Nd, Li, Pr, V, W, Be, Ce	7
	Plating	Al, Cu, Zn, Cr, Ni	F, Ge, Y, Pd, As, La, Si, Tb, Ba, In, Sn, Ru, Dy, Rh, Ir, Mg, Hf, Ti, Zr, Nb, Ta, Sm, Te, U, Pt, Ga, Co, Sb, Bi, Nd, Li, Pr, Mn, V, W, Be, Ce	5
	Battery Manufacturing	Al, Co, Cu, Sb, Zn, Cr, As, Li, Mn, Ni	F, Ge, Y, Pd, La, Si, Tb, Ba, In, Sn, Ru, Dy, Rh, Ir, Mg, Hf, Ti, Zr, Nb, Ta, Sm, Te, U, Pt, Ga, Bi, Nd, Pr, V, W, Be, Ce	10
	Lithium Battery Recycling	Mg, Al, F, Si, Co, Cu, V, Pd, Ti, Zn, Cr, Ba, Li, Mn, Ni	Ge, Y, As, La, Tb, In, Sn, Ru, Dy, Rh, Ir, Hf, Zr, Nb, Ta, Sm, Te, U, Pt, Ga, Sb, Bi, Nd, Pr, W, Be, Ce	15

Section S4. Supplementary Figures

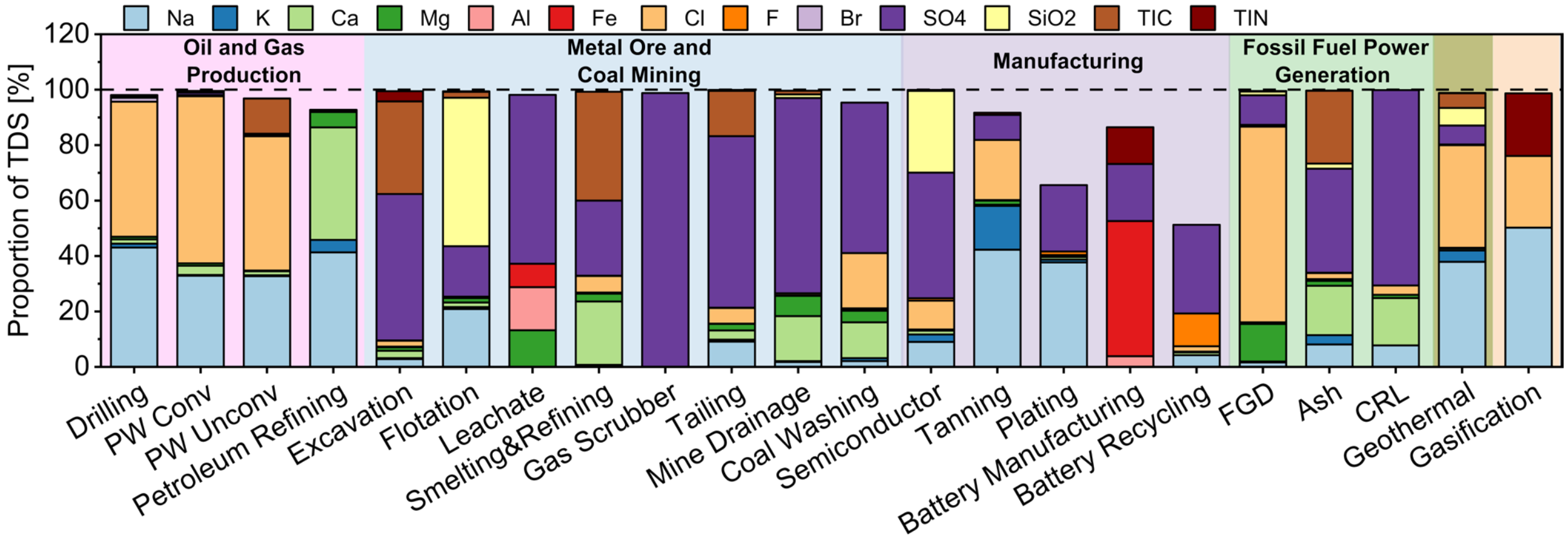

Figure S2. Proportion of individual background species in total dissolved solids without electroneutrality adjustment. Median proportion of background species in total dissolved solids that calculated from the sum of all reported inorganic species. PW Conv = produced water from conventional production, PW Unconv = produced water from unconventional production, Leachate = spent leachate, Tanning= leather tanning and finishing, Battery Recycling= lithium battery recycling, FGD = flue gas desulfurization, Ash = ash transport water, CRL = combustion residual leachate, Geothermal = geothermal power generation. Proportions higher than 100% are a result of taking median concentrations directly to proportions. Proportions lower than 100% reflect the species not belonging to the identified background species and are critical minerals.

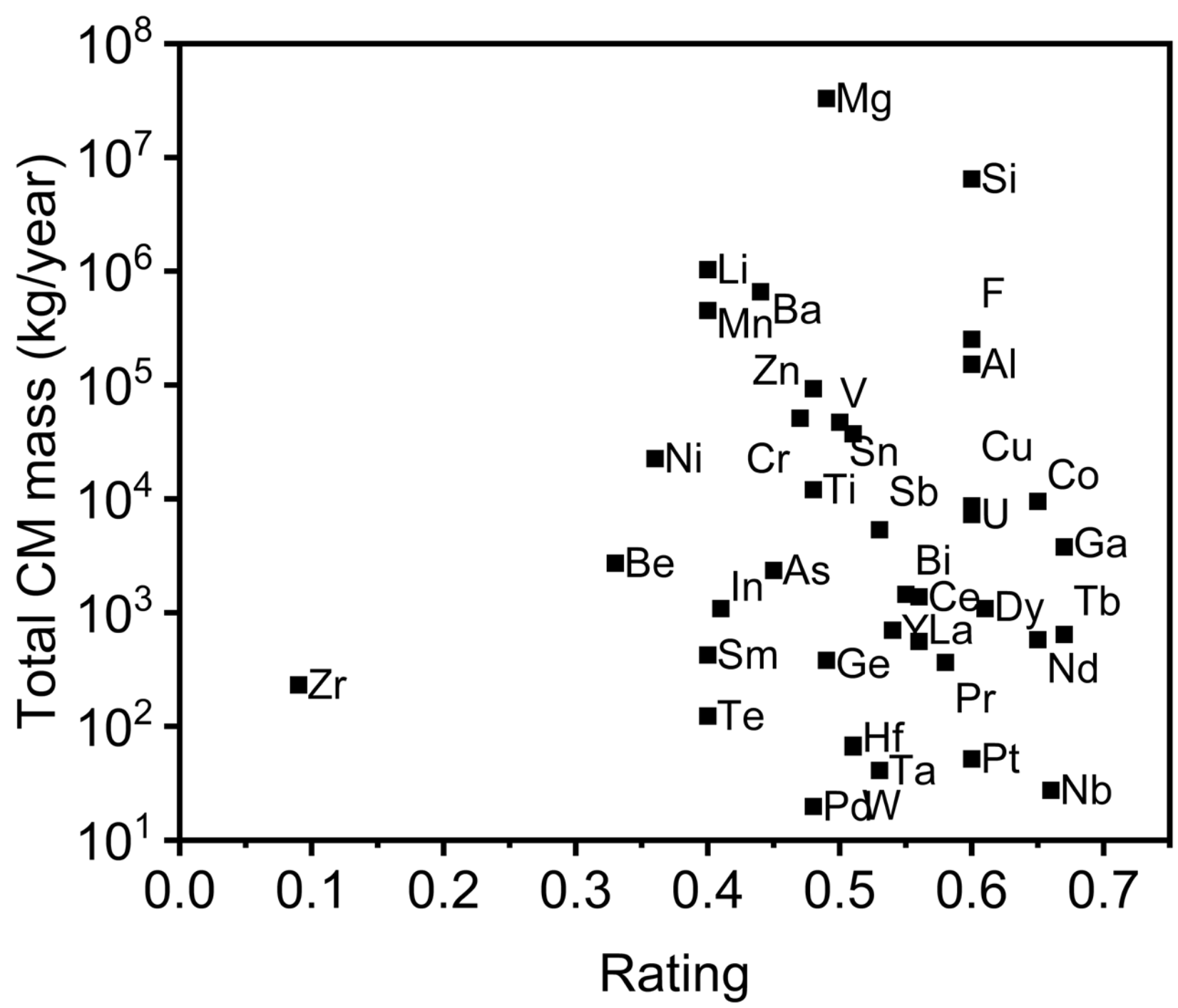

Figure S3. Criticality rating vs. dissolved mass. Estimated annual mass flow (kg/year) of critical minerals listed in Table 1 vs. their respective criticality ratings.

Section S5. Calculation of Annual Wastewater Volumes

This section details the methodology for estimating wastewater generation volumes across industries and industrial activities. Relevant data and data sources are presented below and further documented in **Supplementary Dataset 2**.

S5.1 Oil and gas production annual wastewater volume

The estimated annual wastewater volume from oil and gas production, $Q_{ww\text{-}O\&G}$ is calculated as:

$$Q_{ww-O\&G} = Dilling\ distance\ [m] \cdot R_{ww-dilling} + \sum_i Produced\ hydrocarbon_i \cdot R_{ww-i}$$

Where i represents an individual waste stream from the activity, and R_{WW} is the rate of wastewater generation per unit of production, described in the Main Text. Note that we assume all produced oil will go through petroleum refining step. R_{WW} values for these activities were obtained from technical reports by the USGS, American Petroleum Institute (API), and other international publications and are listed in **Table S5**.

Table S5. Volume of waste streams generated from oil and gas production

Waste Streams	R_{ww}	Unit
Drilling wastewater[16]	1.59	m^3/m-drilled
Produced water conventional-oil ($PW_{cov\text{-}oil}$)[17]	9.75	m^3/m^3-oil produced
Produced water conventional-gas ($PW_{cov\text{-}gas}$)[17]	483	m^3/Mm^3-gas produced
Produced water unconventional-oil ($PW_{uncov\text{-}oil}$)[18]	0.35	m^3/m^3-oil produced
Produced water unconventional-gas ($PW_{uncov\text{-}gas}$)[18]	208	m^3/Mm^3-gas produced
Petroleum refining wastewater[19]	2.7	m^3/m^3-oil processed

The amount of produced hydrocarbon of all types (conventional and unconventional), and the drilling distance are documented in **Table S6**. For countries that were missing information on unconventional production proportion, we assumed all hydrocarbon production was conventional. For countries that were missing drilling information, wastewater from drilling was not considered.

Based on these assumptions, the estimated $Q_{ww\text{-}O\&G}$ in the U.S. in 2024 is 7,703 Mm^3/year. If only produced water is considered, the amount is 3,835 Mm^3/year, which is similar to a previous, separate estimate by Argonne National Laboratory (ANL) in 2009 (3,420 Mm^3/year).[17] This consistency suggests that our assumptions and input data are reasonable for this level of analysis. The estimated wastewater generated globally is 46,941 Mm^3/year.

Table S6. Global oil and gas production, drilling distance, and estimated wastewater generation in 2024.

Country	Oil Production[20] [m^3]	Proportion of Unconventional Oil	Gas Production[20] [Mm^3]	Proportion of Unconventional Gas	Drilling Distance [m/year]	$Q_{ww\text{-}O\&G}$ [Mm^3/year]
United States	966,204,342	0.64[21]	840,579	0.78[21]	787,985,590[22]	7,703.14
China	640,099,881	0.02[23]	650,020	0.12[24]	33,044,810*[25,26]	8,201.35
Russia	254,579,058	Unknown	594,995	Unknown	29,400,000[27]	3,504.59
India	208,229643	Unknown	314,995	Unknown	Unknown	2,745.30
Iran	194,437881	Unknown	245,640	Unknown	Unknown	2,540.05

Saudi Arabia	170,620965	Unknown	223,217	Unknown	Unknown	2,232.62
Japan	148,905189	Unknown	220,925	Unknown	Unknown	1,961.08
Canada	123,431904	Unknown	175,154	Unknown	Unknown	1,621.74
Germany	977,79933	Unknown	167,844	Unknown	Unknown	1,298.75
South Korea	97,511,823	Unknown	165,097	Unknown	Unknown	1,294.09
Other countries**	1,061,325,126	Unknown	1,286,881	Unknown	Unknown	7,703.14
**Global annual wastewater volume [Mm^3/year]**						**46,941.38**
* The total drilling distance in China is unavailable, however, we made an estimation using the average drilling distance per well (2634.46 m/well)[1] and the number of new wells in 2024 reported from the top three hydrocarbon producing companies in China.<br>**Other countries account for 26.8% of the global hydrocarbon production.						

S5.2 Metal and coal mining annual wastewater volume

The estimated global annual wastewater volume from mining ($Q_{ww\text{-}mine}$) is calculated by:

$$Q_{ww-mine} = \sum_i (Metal\ and\ coal\ production\ from\ raw\ ore_i[ton] \cdot R_{ww-i}\left[\frac{m^3}{ton}\right] + Tailing\ overflow\ and\ mine\ drainage\ \left[\frac{m^3}{day \cdot mine\ site}\right] \cdot number\ of\ mine\ site_i)$$

where i is type of mines, such as Cu and Pb. Note that because spent leachate is only generated when leaching-electrorefining process was used for metal ore beneficiation, this value is only considered for a portion of the total production for a specific metal. The proportion of the global metal production that uses electrorefining is documented in **Table S7.**

The R_{ww} of waste streams from metal and coal mining were obtained from technical reports by the EPA and various international publications and are tabulated in **Table S7**. We analyzed the R_{ww} separately for top-producing mines including Fe, Pb and Zn, Cu, and Coal. For other metal mine types, due to the lack of data we used a single median value across all mine types. In cases where the reports did not provide R_{ww} directly, but did report the volume of wastewater and mass of processed ores, we calculated the R_{ww} as:

$$R_{ww} = \frac{Q_{waste\ stream}[m^3]}{M_{ore}[ton] \cdot \pi_{metal} \cdot \eta_{recovery}}$$

where M_{ore} is the mass of ore excavated. π_{metal} is the proportion of metal in ore, and $\eta_{recovery}$ is the recovery efficiency during ore beneficiation.

Table S7. Median R_{ww} values associated with mining activities.

Mine type / Waste streams	R_{ww}					Unit
	Iron (Fe)[4]	Lead (Pb) & Zinc (Zn)[4,6]	Copper (Cu)[4,5]	Other metal [4,6,7]	Coal [28]	
Excavation	39.44	176.55	18.19	39.44		m^3/ton-metal produced
Flotation	26.49	115.66	43.12	43.12		m^3/ton-metal produced
Spent Leachate*				0.66		m^3/ton-metal produced
Smelting & Refining		47.59	10.17	27.07		m^3/ton-metal produced
Waste Gas Treatment			199.52	19.52		m^3/ton-metal produced
Coal Washing					1.25	m^3/ton coal processed
Tailing Overflow[29] and Mine Drainage[30,31]				1,415		m^3/day/mine site

* This value is only applicable when the leaching-electrorefining process was used. Approximately, electrorefining are used for 0% of Fe and Pb beneficiation, 10.26% of nickel production[32], 20.4% of Cu production[33], 90% of Zn production[34], 90% of Au production[35], 100% of Al production[36].

Table S8 and **Table S9** summarize the total metal and coal production in the U.S. and around the world, respectively, along with our estimated $Q_{ww\text{-}mine}$ values. In the U.S., our estimated volume (4841 Mm^3/year) is significantly higher than one previously reported estimate (~140 Mm^3/year),[37] as well as the total wastewater discharge reported by the EPA (~1,076 Mm^3/year).[29]

The deviation from earlier estimates is because they only included mine process water from Cu mines, without considering other types of mines. The deviation from EPA reported discharge is because they only included for wastewater discharged to the local watershed, while our estimate also account those directed to the tailings pond for storage, not including the recycled volume. Globally, we estimate $Q_{ww\text{-}mine}$ as 197,969 Mm^3/year.

Table S8. U.S. mine production and estimated wastewater volume

Product	Number of Mine Sites in the U.S.	U.S. Metal and Coal Production 2024 [tons/year][12]	Estimated Wastewater Production in the U.S. [Mm^3/year]	Note
Iron (Fe)	12[38]	30,000,000[12]	3,380.77	No electrorefining
Gold (Au)	72[39]	160[12]	0.11	Assume 90% produced Au use electrorefining.[35]
Lead (Pb)	13[40]	300,000[12]	102.35	No electrorefining
Zinc (Zn)	15[41]	750,000[12]	276.61	Assume 90% produced Zn use electrorefining.[34]
Copper (Cu)	17[12]	1,100,000[12]	107.83	Assume 20.4% produced Cu use electrorefining.[33]
Nickel (Ni)	1[42]	8,000[12]	0.92	Assume 10.26% produced Ni use electrorefining.[32]
Aluminum (Al)	0[12]	670,000[12]	68.85	Assume 100% produced Al use electrorefining.[36]
Coal	560[43]	524,262,000[44]	904.28	
**U.S. annual wastewater volume [Mm^3/year]**			**4,841.71**	

Table S9. Global mine production and estimated wastewater volume

Product	Global Metal and Coal Production 2024 [tons/year][45]	Global Mine Sites[46]	Estimated Wastewater Production [Mm^3/year]
Iron (Fe)	1,600,000,000	474	180,233.89
Gold (Au)	3,300	1,500	2.26
Lead (Pb)	4,300,000	450	1,584.20
Zinc (Zn)	12,000,000	524	4,552.05
Copper (Cu)	23,000,000	837	2,468.78
Nickel (Ni)	3,700,000	197	305.98
Aluminum (Al)	72,000,000	85	7,436.17
Coal	8,900,000,000[44]	3119	12,511.57
**Global annual wastewater volume [Mm^3/year]**			**197,969.9**

S5.3 Manufacturing annual wastewater volume

The estimated annual wastewater volume from manufacturing ($Q_{ww\text{-}manufacturing}$) is calculated by:

$$Q_{ww-manufacturing} = R_{ww}\left[\frac{m^3}{product}\right] \cdot production\ rate\ \left[\frac{product}{year}\right]$$

The R_{ww} values for manufacturing wastewaters were obtained from multiple international publications and are listed in **Table S10.**

Table S10. Estimated global wastewater generation from manufacturing.

Manufacturing Industries	R_{ww}	Unit	Production Rate	Unit	$Q_{ww\text{-}manufacturing}$
Semiconductor Manufacturing	0.028[47,48]	Mm^3/day/plant	438	Number of plants global	4,478
Leather Tanning and Finishing	32.5[49]	m^3/ton-raw hide	13.4	million ton global raw hide production/year	435
Metal Plating and Finishing	18.87[50]	Mm^3/year-U.S.	3.23	Market size global/U.S.	60.87
Battery Manufacturing	29.73[8]	Mm^3/year-U.S.	6.99	Market size global/U.S.	3.50
Lithium Battery Recycling	675[51]	m^3/ton-recycled	1.6	million ton/year	691
**Global annual wastewater volume [Mm^3/year]**					**5,183.3**

S5.4 Fossil fuel power generation annual wastewater volume

The estimated annual wastewater volume from fossil fuel power generation ($Q_{ww\text{-}power}$) is calculated as:

$$Q_{ww-power} = R_{ww}\left[\frac{Mgal}{year \cdot EGU}\right] \cdot Number\ of EGU$$

The R_{ww} of waste streams from fossil fuel power plants in the U.S. were directly obtained from the "Technical Development Document for Effluent Limitations Guidelines…" report by EPA and are listed in **Table S11**. [52]

Table S11. Volume of waste streams generated per unit of electric generating unit (EGU)

Waste Streams	R_{ww} [Mgal/year/EGU*]	R_{ww} [Mm^3/year/EGU*]
Flue gas Desulfurization (FGD)	76.15[52]	0.32
Bottom Ash Transport (BA)	23.57[52]	0.1
Combustion Residual Leachate (CRL)	11.56[52]	0.05
**Total**	**111.29**	**0.47**
* Electric generating unit (EGU): a power-generating unit of a power plant. Equipment included in the unit depends on specific power plant plan. For example, in the case of a steam electric generation unit, the EGU consists of all equipment involved in fuel delivery to the plant site, as well as individual boilers, any installed emission control equipment, and any steam turbine/generators dedicated to generating electricity. More detailed definition and examples can be found in Washington Administrative Code, Carbon dioxide mitigation program, WAC 463-85-110. [53]		

We first estimated the $Q_{ww\text{-}power}$ in the U.S. In 2020, there are 235 EGU from coal-fired power plants.[52] Therefore, $Q_{ww\text{-}power}$ is estimated to be 111.3 Mm^3/year. This estimated value is comparable to previously reported value (76 Mm^3/year).[37]

To estimate the global wastewater volume, we obtained the total power generation capacity by coal-fired power plants around the world[54], and projected the number of EGU using the power generation for each country:

$$EGU_{country} = EGU_{U.S} \cdot \frac{Power\ Generation\ Capacity_{country}}{Power\ Generation\ Capacity_{U.S.}}$$

As a result, we estimate the global annual wastewater volume from coal-fired power plants to be 1,063 Mm^3/year. The power generation capacity and estimated EGU for each country are tabulated in **Table S12**.

Table S12. Estimated global wastewater generation from coal-fired power plants.

Country	2024 Power Generation Capacity [gigawatts] [54]	Estimated Number of EGU[52]	Q_{ww} [Mm^3/year]
U.S.	196.22	235	111.3
China	1,147.23	1,374	650.6
India	239.65	287	135.9
Japan	54.75	66	31.1
Indonesia	52.32	63	29.7
South Africa	44.22	53	25.1
South Korea	41.18	49	23.4
Russia	37.81	45	21.4
Germany	32.41	39	18.4
Poland	28.46	34	16.1
**Global annual wastewater volume [Mm^3/year]**			**1,063.0**

S5.5 Geothermal produced wastewater volume

The estimated geothermal produced water volume ($Q_{ww\text{-}geothermal}$) is calculated by:

$$Q_{ww-geothermal} = R_{ww}\left[\frac{kg}{kWh}\right]\cdot\frac{geothermal\ power\ capacity[MW]}{geothermal\ utilization\ rate}\cdot 8760\frac{h}{year}$$

The geothermal utilization rate describes the efficiency with which geothermal water is converted to energy, and was over 75% as of 2023.[55] The R_{ww} of waste streams from geothermal produced water was calculated by:

$$R_{ww}[\frac{\text{kg}}{\text{kwh}}] = \frac{1}{\eta_{power}[\%]\cdot h[\frac{kJ}{kg}]}\cdot\frac{Q_{injection}\ [\frac{Mgal}{day}]}{Q_{production}\ [\frac{Mgal}{day}]}$$

where R_{ww} (kg/kwh) is the mass of produced water for every kWh energy generated, η_{power} (%) is the heat-to-energy conversion efficiency of power plant, h (kJ/kg) is the heat capacity of geothermal fluid, $Q_{injection}$ (Mgal/day) is the flow rate of produced water injected back to the geothermal production well and $Q_{production}$ (Mgal/day) is the flow rate of geothermal fluid extracted from the well. The values of η_{power} and h were obtained from Zarrouk et al.,[56] and the injection and production flow rate are obtained from Argonne National Laboratory.[17] The values used in our calculations are tabulated in **Table S13**.

Table S14 summarize our estimated wastewater volumes in the U.S. and globally. The estimated $Q_{ww\text{-}geothermal}$ in the U.S. is 347 Mm^3/year, similar to a previously reported value (381 Mm^3/year).[57] The estimated global $Q_{ww\text{-}geothermal}$ is 1,556 Mm^3/year

Table S13. Parameters for R_{ww} calculation

Parameters	Highest Value	Lowest Value
η_{power}	21% (Single/multi-stage flash)	10% (Binary)
h	2,850 kJ/kg	306 kJ/kg
**Technology**	**$Q_{production}$ [Mgal/day]**	**$Q_{injection}$ [Mgal/day]**
Binary	14.317	13.98
Binary	46.091	44.198
Binary	35.386	33.126
Multi-stage flash	25.195	13.113
Multi-stage flash	53.381	43.664

Table S14. Global geothermal power capacity and estimated wastewater generation

Country	Geothermal Power Capacity[58] [MW]	$Q_{ww\text{-}geothermal}$ [Mm^3/year]
U.S.	3,397	346.83
Indonesia	2,653	270.87
Philippines	1,984	202.56
Türkiye	1,734	177.02
New Zealand	1,207	123.23
Kenya	985	100.5
Mexico	976	99.65
Italy	916	93.52
Iceland	786	80.25
Japan	601	61.36
Total	**15239**	
**Global annual wastewater volume [Mm^3/year]**		**1,555.91**

S5.6 Gasification annual wastewater volume

The estimated annual wastewater volume from coal gasification ($Q_{ww\text{-}gasification}$) is calculated by:

$$Q_{ww-gasification} = R_{ww}\left[\frac{m^3}{Mm^3 gas}\right] \cdot production\ rate\ [\frac{Mm^3 gas}{year}]$$

The R_{ww} values for coal gasification wastewaters were directly obtained from multiple international publications[59,60] and the median is 5329 m^3/ Mm^3-gas.

The syngas production from coal gasification in the U.S. was not found. However, the production in China was found to be 4298 Mm^3 gas/year in 2022[61]. Because China held around 24.07% of the global syngas market[62], the global syngas production was estimated to be 17,852 Mm^3 gas/year. Therefore, the global wastewater generation from gasification is calculated to be:

$$Q_{ww-gasification} = 5329\left[\frac{m^3}{Mm^3 gas}\right] \cdot 17{,}852\ \left[\frac{Mm^3 gas}{year}\right] = 95.13\left[\frac{Mm^3}{year}\right]$$

Section S6. Oil and Gas Production

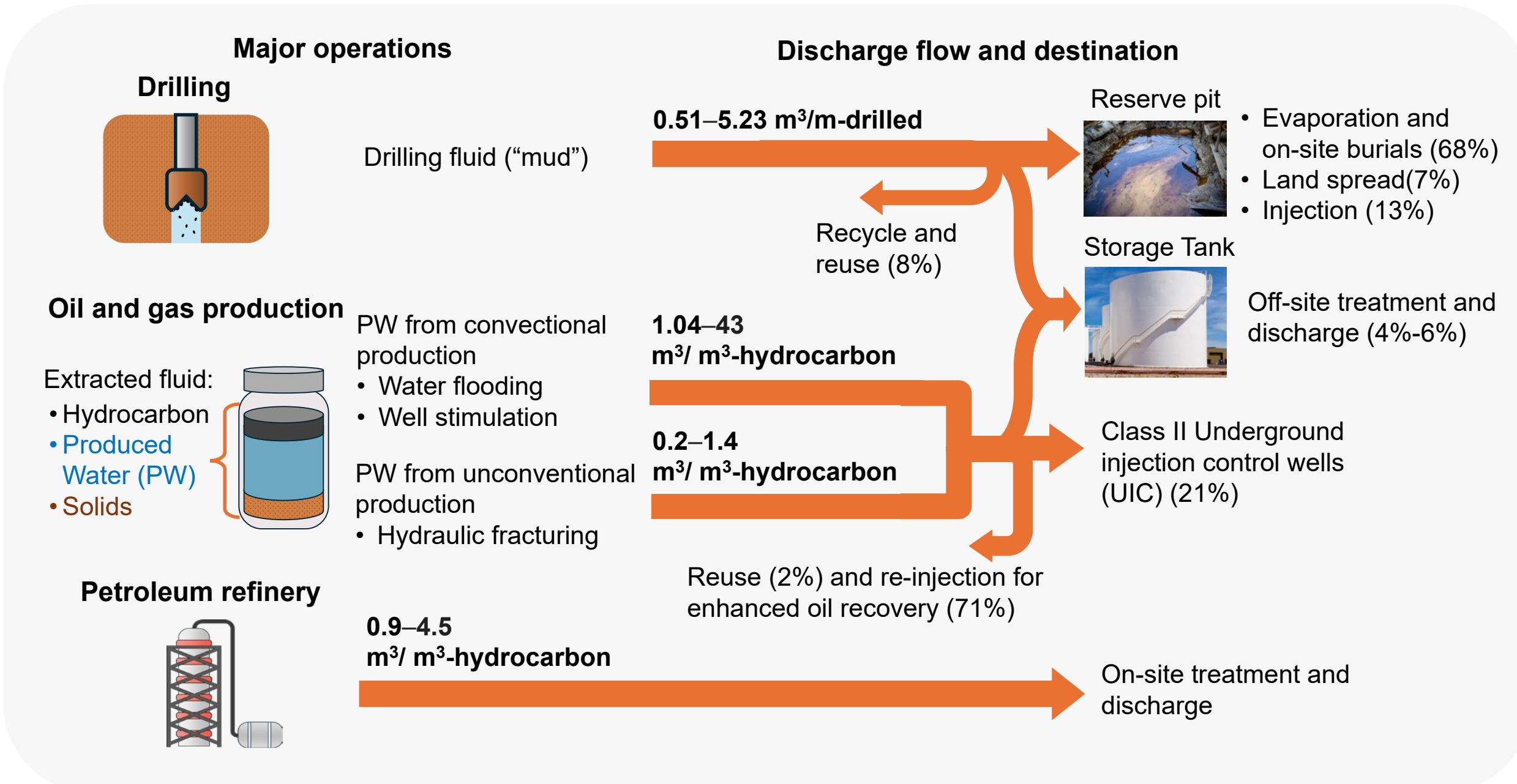

Figure S4. Schematic of wastewater generation from oil and gas production.

S6.1 Industry description

Oil and gas production involves 1) drilling exploration and production wells to locate and produce hydrocarbons (petroleum and natural gas), 2) extracting hydrocarbon from these wells, and 3) refining produced hydrocarbon into marketable products.[63] Hydrocarbon reserves are located underground at depths ranging from the land surface to as deep as 12 km, and are trapped in porous sedimentary rocks (reservoir rocks).[64] Hydrocarbons are categorized into various types based on the geological characteristics of the reserve (e.g., reservoir rock porosity and permeability); examples include conventional, shale gas, and deep off-shore. Hydrocarbon production is classified as "conventional" or "unconventional" depending on the type of reserve and the extraction technologies being used.[63] As of 2023, there were 405,071 oil wells and 512,997 natural gas wells producing hydrocarbons in the U.S., accounting for around 20% of total global oil and gas production.[65,66] Approximately 15,000 new wells are established every year, and existing wells are also being continuously exploited.[67]

S6.2 Overview of oil and gas production wastewater streams

Collectively, oil and gas production generates around 9.4 Mm^3 of wastewater every day in the U.S.[16,17] This wastewater originates from all three activities described above—drilling, hydrocarbon production (as produced water, PW), and refining processes, with PW being the primary source.[16] **Figure S4** shows the major operations, flow rates, and discharge destinations of the oil and gas production industry. Wastewater is usually directed into on-site reserve pits or storage tanks for subsequent disposal or reuse. Drilling waste is usually disposed of through evaporation and on-site burial (68% of total waste); whereas PW is mostly managed through deep well injection, where 71% is injected for enhanced oil recovery, and 21% is injected into underground injection control (UIC) wells for disposal.[16,68] Only 4-6% of liquid waste from both drilling waste and PW are treated for subsequent disposal.[16,69]

S6.3 Drilling wastewater

Drilling activities are performed during both exploration and development of hydrocarbon wells. Typically during drilling, drilling fluids (muds) are injected into the borehole through the drill bits, and drill cuttings are carried with the drilling fluids to the surface. Used drilling fluid and drill cuttings constitute drilling waste. Drilling waste consists of both solid waste and liquid wastewater, with the wastewater accounting for around 73.6% of the total volume.[16] Drilling fluids are formulated using complex mixtures of natural and synthetic chemical compounds, designed to achieve specific fluid and chemical properties that meet the required design criteria (e.g., maintaining borehole stability and cooling).[16] Water-based drilling fluid is currently mostly used over other types, which include oil-based fluid and emulsions.[70] The amount of drilling fluid used per well is proportional to the drill length. In the U.S., the average drill length of exploration and production wells was 18,000 feet (5.5 km) in 2021, increased by approximately 8,000 feet (2.4 km) since 2010, and is expected to continue increasing for both existing and new wells.[71,72]

Figure S5 and **Table S15** show the typical volume and characteristics of drilling wastewater. For every meter drilled, around 0.51 to 5.23 m^3 of wastewater is generated.[16] The pH ranges from 5.55 to 9.90, while the TDS ranges from 284 to 79,586 mg/L.[73–75] The TOC ranges from 7 to 3,950 mg/L. Within all major background species presented, Cl, Na, K, Ca, Mg, and Br are the most prominent, reaching a few thousand mg/L. The major CMs are Al, Mg, Ba, and Li, with concentrations reaching a few hundred mg/L.

The wastewater is typically alkaline, as high pH drilling fluid provide optimal mud rheological and filtration properties.[76] The wide range of reported TDS for drilling wastewater is due to the use of both fresh water-based and sea water-based drilling fluids[16] The TOC of the wastewater comes from organic compounds used in drilling fluid (e.g., carboxymethyl cellulose).[16] CMs in wastewater can originate from the dissolution of drill cuttings, mixing with groundwater, and the addition of drilling fluid chemicals. Examples of chemical additives include Zn oxide, manganese tetroxide, and ilmenite (Ti-Fe oxide).

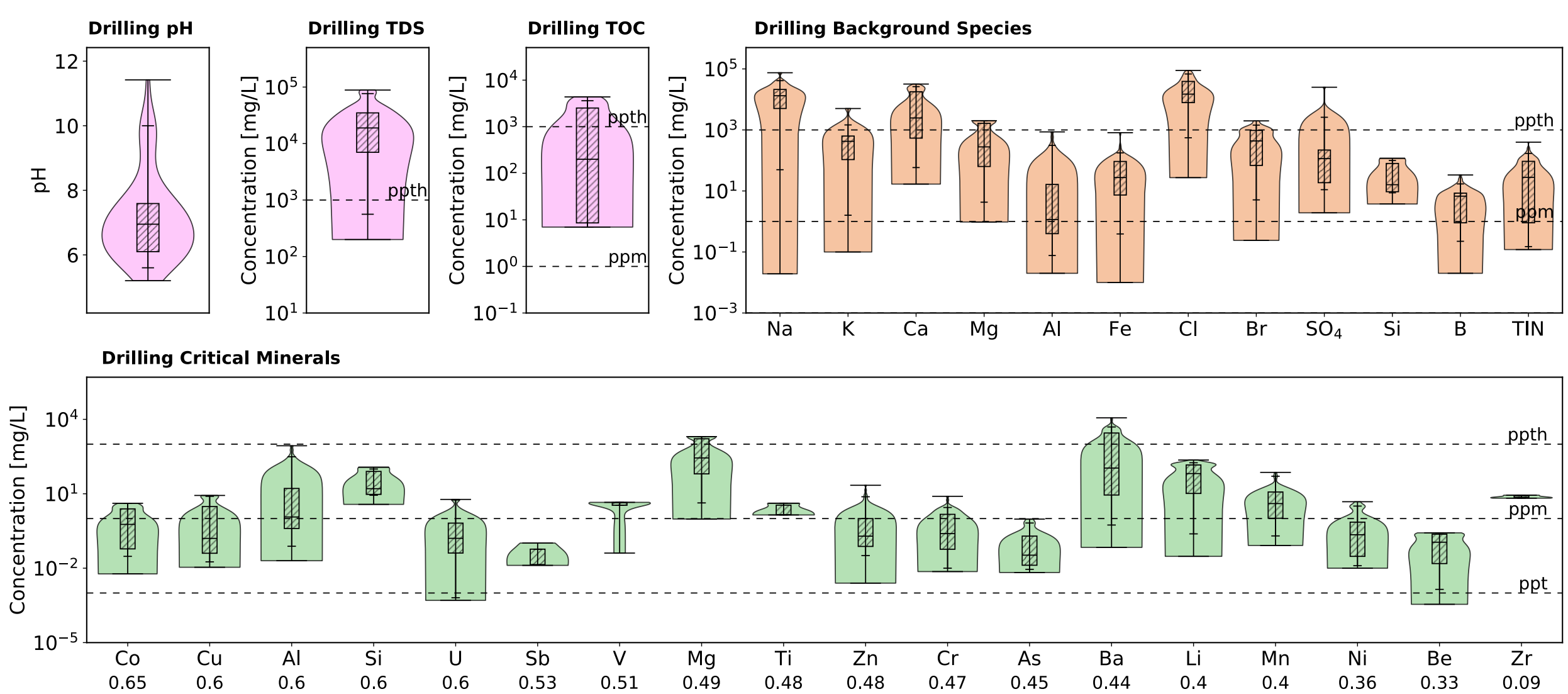

Figure S5. Drilling wastewater pH, TDS, TOC, background species, and critical minerals concentration distribution.

Table S15. Drilling wastewater volume and characteristics. Values in parentheses represent the median concentrations; ranges show the 5th to the 95th percentile.

Drilling			
**Flow [m^3/m-drilled]**	**pH**	**TDS [mg/L]**	**TOC [mg/L]**
0.51–5.23 (1.59)	5.56–10 (6.95)	488–79,594 (18,884)	7.00–3,950 (200)

Wastewater Constituents					
**Background Species**	**Concentration [mg/L]**	**Median Mineral Proportion in TDS [%]**	**Critical Minerals**	**Concentration [mg/L]**	**Criticality Rating**
Cl	190–74,760 (14,935)	49	Co	0.03–4.14 (0.58)	0.65
Na	49–41,837 (13,200)	43	U	6.29×10^{-4}–5.63 (0.16)	0.6
Ca	29–25,445 (474)	1.55	Al	0.06–488 (1.16)	0.6
Br	5.10–1,533 (436)	1.42	Cu	0.02–8.36 (0.16)	0.6
K	0.93–3,066 (424)	1.38	Sb	0.01–0.09 (0.01)	0.53
Mg	4.10–1,985 (278)	0.91	V	1.06–4.51 (4.40)	0.51
SO_4	9.60–2,600 (115)	0.38	Mg	4.10–1,985 (278)	0.49
TIC	43–761 (108)	0.35	Ti	1.40–4.16 (1.44)	0.48
TIN	3.49–211 (64)	0.21	Zn	0.03–8.39 (0.20)	0.48
Si	4.98–113 (16)	0.05	Cr	0.01–3.06 (0.25)	0.47
Fe	0.12–134 (15)	0.05	As	8.43×10^{-3}–0.74 (0.03)	0.45
B	0.18–20 (6.75)	0.02	Ba	0.55–5,245 (82)	0.44
Al	0.06–488 (1.16)	3.78×10^{-3}	Mn	0.19–51 (4.02)	0.4
			Li	0.24–179 (65)	0.4
			Ni	0.01–3.83 (0.22)	0.36
			Be	9.28×10^{-4}–0.27 (0.11)	0.33
			Zr	6.64–8.76 (7.16)	0.09

S6.4 Produced water

Hydrocarbon production is always associated with water production, as most hydrocarbon-bearing rocks contain water in their pore spaces (formation water).[77] In addition to formation water, water used in activities such as waterflooding for enhanced recovery, hydraulic fracturing for hydrocarbon reservoir stimulation, and residuals from drilling also contributes to PW.

Water use in conventional and unconventional hydrocarbon production differs as different extraction methods are used. In conventional production, minimal water is used for primary recovery, as hydrocarbons are in highly permeable rocks and can flow naturally to the well under reservoir pressure. As more hydrocarbons are drawn from the well, reservoir pressure declines, and the remaining hydrocarbons are often trapped in less permeable rocks. Methods such as water flooding and steam stimulation are then used for secondary and enhanced recovery.[78] In unconventional production, hydrocarbons are usually found in very low-permeability rocks that need to be fractured for extraction. Therefore, hydraulic fracturing, which primarily uses pressurized water-based fluid, is commonly performed.[77] In the U.S., 64% of oil and 89% of natural gas come from unconventional production.[21,79]

When hydrocarbon production begins, production fluid that contains a mixture of oil, gas, and water flows to the surface. Water is then separated from hydrocarbons through a series of separation and recovery treatments, and all water is combined as waste PW. The amount of wastewater generated per volume of hydrocarbons produced varies significantly depending on the geological characteristics of the reservoir, the technology used for production, and the elapsed production time.[77]

Figure S6 and **Table S16** show the typical PW volume and characteristics from conventional hydrocarbon production.[29,80,81] For oil production, the annual wastewater volume ranges from 1.0 to 43 m^3/m^3-oil-produced. For gas production, the volume ranges from 24.7 to 6,782 m^3/Mm^3-gas-produced. The pH ranges from 5.20 to 8.70; the TDS ranges from 2,624-308,000 mg/L, and the TOC ranges from 24 to 1,054 mg/L. Within all background species presented, Na, K, Ca, Mg, Al, Br, SO_4, and TIC are the most prominent, reaching a few thousand mg/L. The major CMs are Al, F, Si, Mg, Cr, Ba, Li, and Mn, with concentrations in the tens of mg/L; other CMs are mostly found at less than 0.1 mg/L.

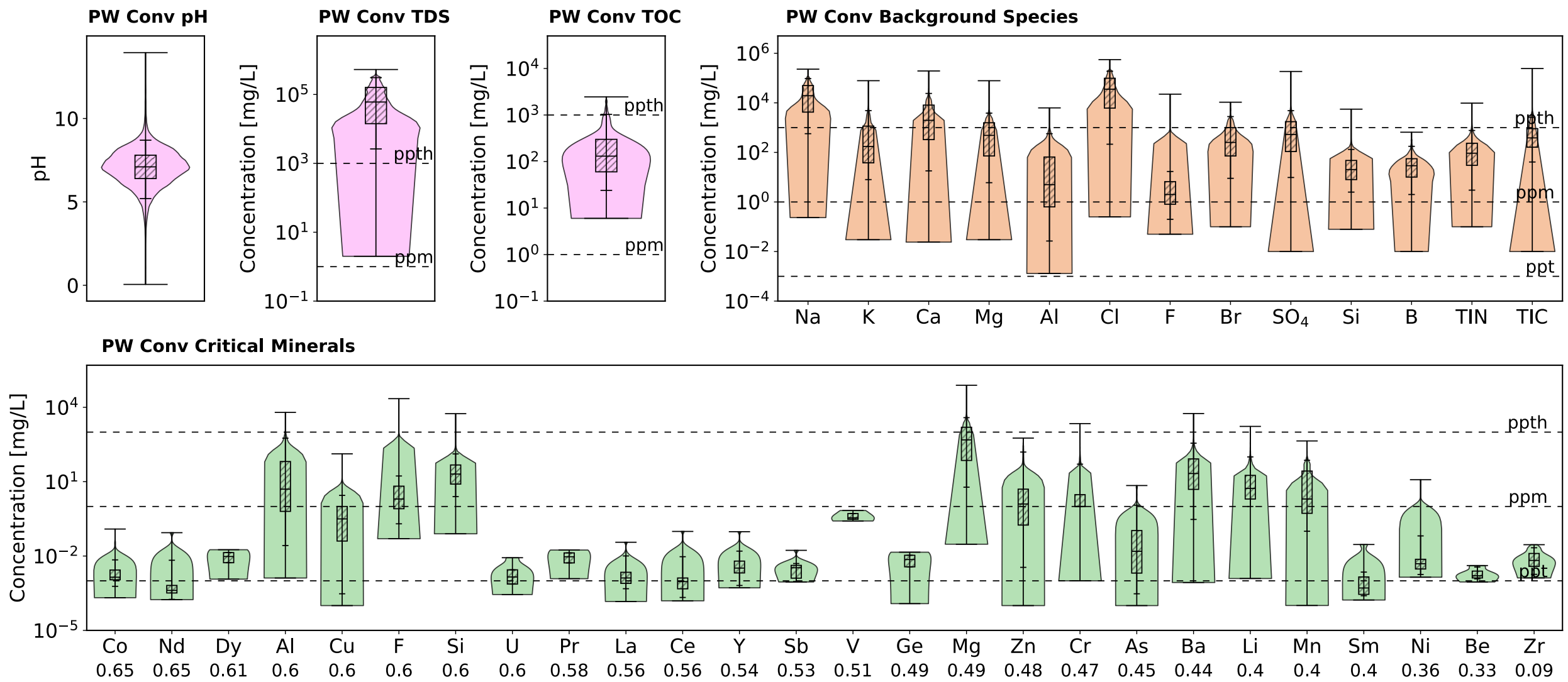

Figure S6. Produced water (conventional hydrocarbon production) pH, TDS, background species, and critical minerals concentration distribution.

Table S16. Conventional produced water volume and characteristics. Values in parentheses represent the median concentrations; ranges show the 5th to the 95th percentile.

Produced Water from Conventional Production					
Flow [m^3/m^3-oil produced]		pH		TDS [mg/L]	TOC [mg/L]
1.04–43 (9.75)		5.20–8.70 (7.10)		2,624–308,000 (60,366)	24–1,054 (130)
**Wastewater Constituents**					
**Background Species**	**Concentration [mg/L]**	**Median Mineral Proportion in TDS [%]**	**Critical Minerals**	**Concentration [mg/L]**	**Criticality Rating**
Cl	214–187,900 (35,519)	60	Co	5.89×10^{-4}–7.15×10^{-3} (1.40×10^{-3})	0.65
Na	569–95,100 (19,374)	33	Nd	6.55×10^{-6}–6.48×10^{-3} (3.21×10^{-4})	0.65
Ca	18–23,971 (1,967)	3.34	F	0.20–17 (2.00)	0.6
SO_4	9.71–4,832 (528)	0.90	Al	0.02–578 (5.00)	0.6
Mg	6.00–3,840 (486)	0.82	Cu	3.00×10^{-4}–2.83 (0.32)	0.6
TIC	30–2,458 (305)	0.52	U	3.72×10^{-4}–7.40×10^{-3} (1.43×10^{-3})	0.6
Br	9.00–2,790 (253)	0.43	La	7.46×10^{-6}–8.51×10^{-3} (8.07×10^{-4})	0.56
K	8.00–4,850 (173)	0.29	Ce	1.24×10^{-5}–8.68×10^{-3} (4.92×10^{-4})	0.56
TIN	2.79–559 (47)	0.08	Y	1.62×10^{-5}–0.03 (2.96×10^{-3})	0.54
B	2.00–176 (29)	0.05	Sb	6.00×10^{-5}–7.99×10^{-3} (2.45×10^{-3})	0.53
Si	2.50–132 (20)	0.03	V	0.27–0.65 (0.35)	0.51
Al	0.02–578 (5.00)	8.48×10^{-3}	Mg	6.00–3,840 (486)	0.49
F	0.20–17 (2.00)	3.39×10^{-3}	Zn	3.37×10^{-3}–163 (1.26)	0.48
			Cr	1.00–52 (1.00)	0.47
			As	2.97×10^{-4}–1.40 (0.02)	0.45
			Ba	0.29–359 (21)	0.44
			Li	1.00–100 (5.36)	0.4
			Mn	0.10–73 (2.00)	0.4
			Ni	1.68×10^{-3}–0.07 (4.90×10^{-3})	0.36
			Be	1.01×10^{-3}–3.94×10^{-3} (1.60×10^{-3})	0.33
			Zr	1.06×10^{-3}–0.02 (6.22×10^{-3})	0.09

Figure S7 and **Table S17** show the water characteristics from unconventional hydrocarbon production.[29] The annual wastewater volume ranges from 0.2 to 1.4 m^3/m^3-hydrocarbon-produced. The pH ranges from 6.00 to 8.60, the TDS ranges from 704-122,000 mg/L, and the TOC ranges from 0.74 to 648 mg/L. Within all background species presented, Cl, Na, Ca, and TIC are the most prominent, reaching a few thousand mg/L. The major CMs are Al, F, Si, Mg, Cr, Ba, Li, and Mn, with concentrations in the tens of mg/L; other CMs are mostly found at less than 0.1 mg/L.

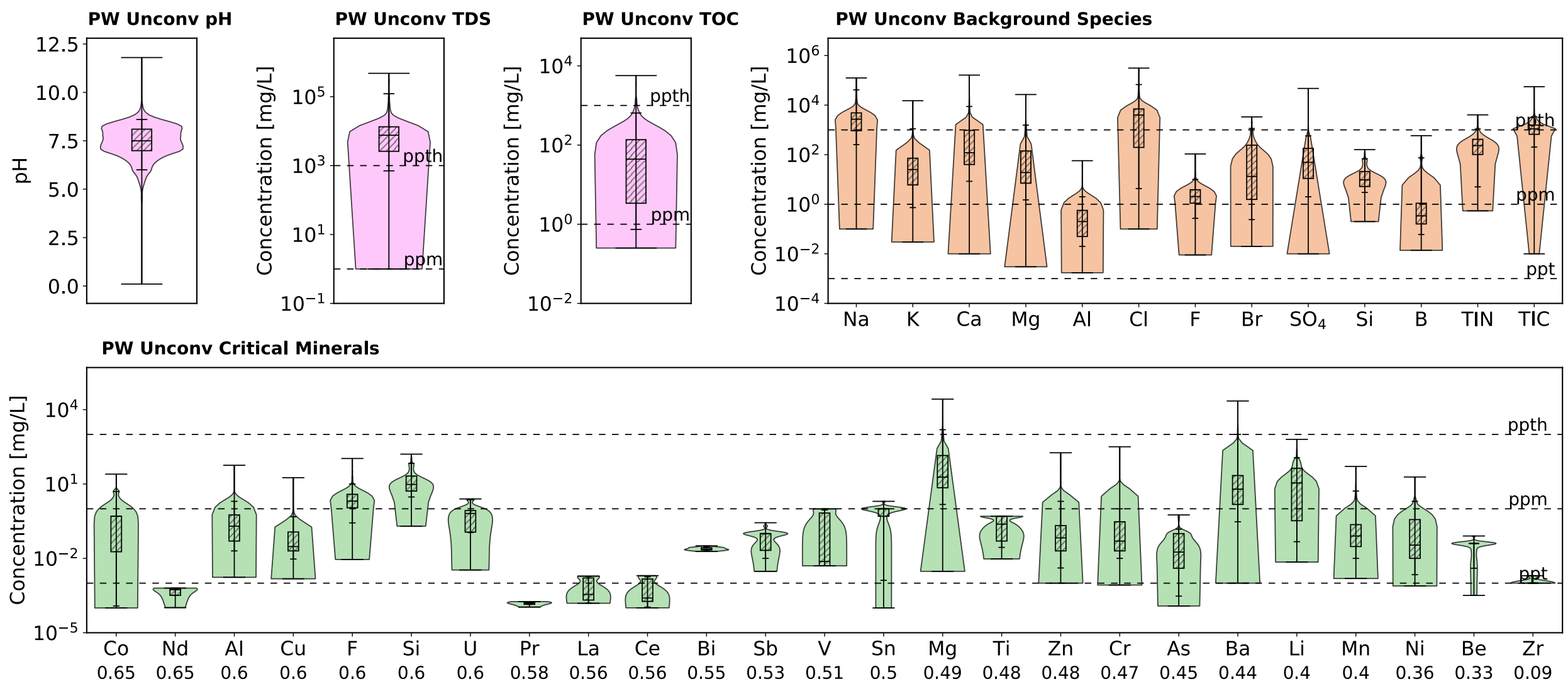

Figure S7. Produced water (unconventional hydrocarbon production) pH, TDS, background species and critical minerals concentration distribution.

Table S17. Unconventional produced water volume and characteristics. Values in parentheses represent the median concentrations; ranges show the 5th to the 95th percentile.

Produced Water from Unconventional Production					
Flow [m^3/m^3-oil produced]		pH		TDS [mg/L]	TOC [mg/L]
0.20–1.40 (0.35)		6.00–8.60 (7.50)		704–122,000 (7,626)	0.74–648 (44)
**Wastewater Constituents**					
**Background Species**	**Concentration [mg/L]**	**Median Mineral Proportion in TDS [%]**	**Critical Minerals**	**Concentration [mg/L]**	**Criticality Rating**
Cl	4.30–67,296 (3,983)	48	Co	1.20×10^{-4}–5.00 (0.50)	0.65
Na	255–41,233 (2,690)	33	Nd	1.60×10^{-5}–6.26×10^{-4} (8.13×10^{-5})	0.65
TIC	60–3,319 (1,040)	13	U	0.10–2.32 (0.65)	0.6
Ca	8.40–8,786 (120)	1.46	Cu	9.31×10^{-3}–0.48 (0.03)	0.6
SO_4	2.00–570 (49)	0.60	Al	0.02–2.00 (0.20)	0.6
K	0.73–1,104 (25)	0.31	F	0.27–10 (2.05)	0.6
Mg	1.50–1,550 (19)	0.23	Ce	1.07×10^{-6}–1.83×10^{-3} (1.88×10^{-4})	0.56
Br	0.24–1,139 (13)	0.16	La	1.07×10^{-6}–1.88×10^{-3} (2.20×10^{-4})	0.56
TIN	0.14–633 (7.03)	0.09	Bi	0.02–0.03 (0.02)	0.55
F	0.27–10 (2.05)	0.02	Sb	0.01–0.16 (0.10)	0.53
B	0.06–74 (0.34)	4.14×10^{-3}	V	5.00×10^{-3}–0.97 (7.50×10^{-3})	0.51
Al	0.02–2.00 (0.20)	2.43×10^{-3}	Sn	1.23×10^{-3}–1.00 (1.00)	0.5
			Mg	1.50–1,550 (19)	0.49
			Zn	4.01×10^{-3}–2.00 (0.07)	0.48
			Ti	0.03–0.50 (0.24)	0.48
			Cr	9.94×10^{-3}–1.00 (0.05)	0.47
			As	2.83×10^{-4}–0.17 (0.02)	0.45
			Ba	0.30–1,029 (6.20)	0.44
			Mn	0.01–5.24 (0.08)	0.4
			Li	0.05–115 (11)	0.4
			Ni	2.18×10^{-3}–2.00 (0.03)	0.36
			Be	4.00×10^{-3}–0.04 (0.04)	0.33
			Zr	1.00×10^{-3}–2.00×10^{-3} (1.00×10^{-3})	0.09

The characteristics of conventional and unconventional PW share some similarities. First, in both cases, the pH can be acidic or basic. Acidic pH is mostly seen in PW from deep formations, possibly due to high concentrations of dissolved CO_2.[82] Extremely low pH conditions may result from the oxidation of sulfur-bearing rocks after produced fluids are stored in reserve pits, or from the addition of acid used to control water chemistry during flooding and fracking.[83] Basic pH may be a result of the equilibration of carbonate species from the reservoir rocks, however, the reason for extremely high pH is unclear. Second, both PW types are high in TDS, TOC, and CM. This is because the formation water originate from ancient seawater that contains high.

There are also differences between conventional and unconventional PW. The annual wastewater volume per unit of hydrocarbon produced is lower for unconventional production, possibly because hydraulic fracturing extracts hydrocarbon more efficiently.[18] Moreover, certain unconventional hydrocarbon reserves (e.g., coalbed methane) do not require hydraulic fracturing. In these cases, the PW is primarily naturally occurring reservoir water and may be lower in volume compared to that from hydraulic fracturing.[84] Unconventional PW also generally has less TDS, possibly because hydraulic fracturing generates more water per well than conventional production, resulting in more dilution.[85] Its TOC is also higher than conventional PW, possible due to the organic additives added to the hydrofracking water.[86]

Despite differences in water quality between production types, we also observed significant variations in PW across different geological formations. To illustrate these differences, we selected six basins from the top six states that together contribute to 80% of PW production in the U.S. as representative examples (**Figure S8**). These states and their contribution to total PW productions are Texas (31%), Illinois (18%), California (12%), Oklahoma (7%), New Mexico (6%), and Wyoming (6%).[81,87] The selected basins, as identified by the USGS,[88] are the Permian (Texas and New Mexico), Gulf Coast (Texas), Illinois (Illinois), San Joaquin (California), Anadarko (Oklahoma), and Greater Green River (Wyoming).

Figure S8 shows the water quality data from these six basins. We observed that the San Joaquin and Greater Green River basins had lower TDS concentration compared to other basins. Specifically, the median TDS of PW from the Greater Green River Basin was 9 times lower than that of the Permian Basin. On the other hand, although the Permian and Gulf Coast Basins had similar TDS ranges, PW from the Gulf Coast contained 1.5 times less sulfate and 3.6 times less magnesium (based on median values). The pH also varied between different basins. PW from the Greater Green River Basin had a higher pH than that from other basins, ranging from 6.0 to 8.8, whereas PW from the Permian Basin had a pH range of 5.4 to 8.3.

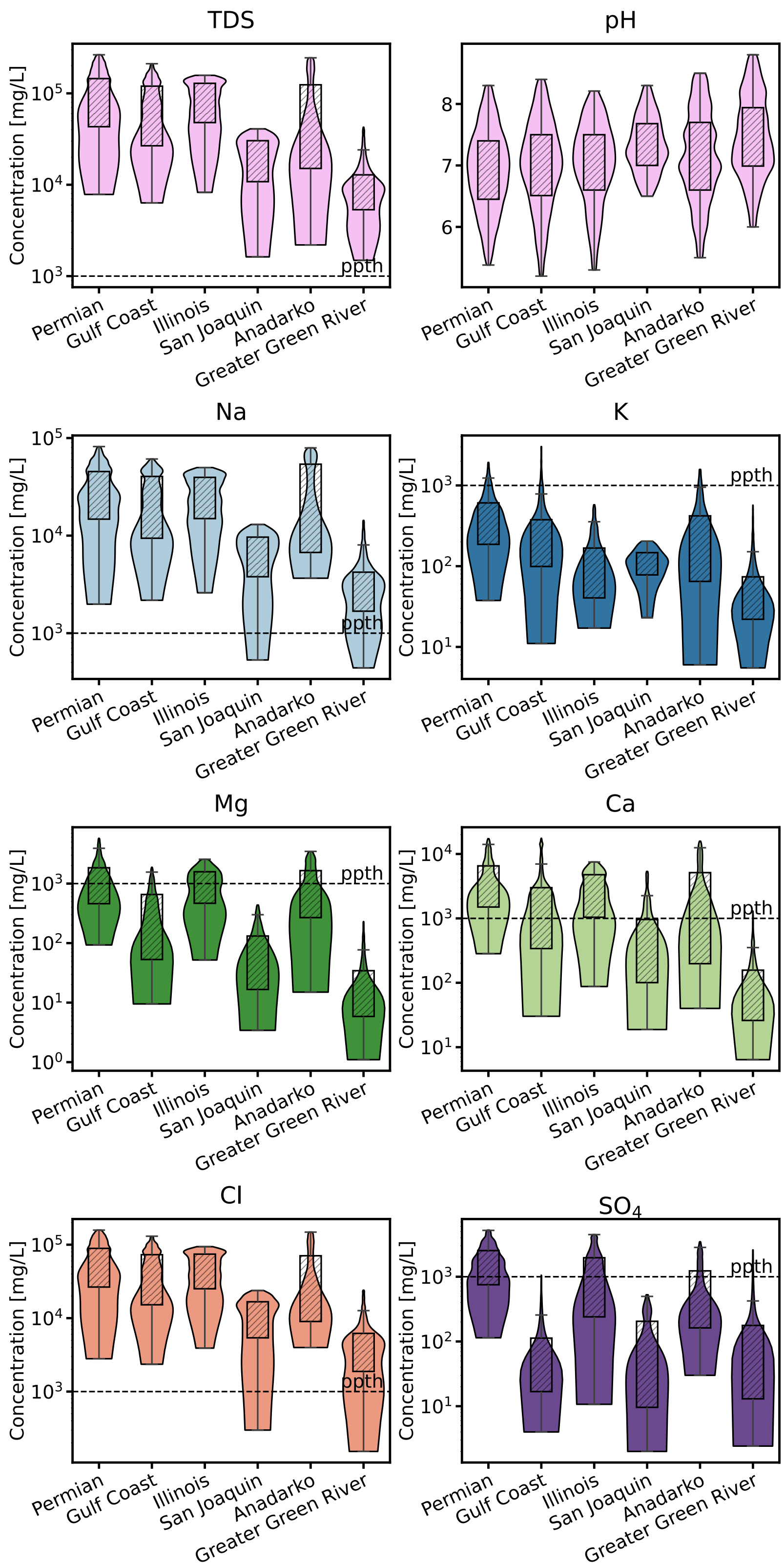

Figure S8. pH, total dissolved solids, and background ion content of produced water from six basins in the top six produced water generating states.

S6.5 Petroleum refining

Raw crude oil contains impurities including water, inorganics, and organics that need to be separated and processed to produce valuable products.[89] The petroleum refining process typically involves crude oil desalting, distillation for component separation, conversion into high-value products, and treatment to remove residual impurities.[89,90] Wastewater is often treated on-site, then discharged to local watersheds.[91] As of 2024, there were 825 refineries in operation around the world,[92] and 132 refineries were in the U.S.[93]

Wastewater from refining originates from the water impurity in crude oil and process water. Depending on the refining process, wastewater can be further categorized into desalter effluent, sour water, tank bottom draws, and spent caustic. Due to data limitations, we are not able differentiate among these wastewater types.[19] However, we note that each type of wastewater may have different characteristics. For example, desalter effluents have neutral pH and TDS; sour water has acidic pH; spent caustic may have high ammonia, sulfide, and cyanide concentration.[19]

Figure S9 and **Table S18** show the typical volume and characteristics for petroleum refinery wastewater that combines all waste streams from refining processes.[19,94] Approximately 0.9 to 4.5 m^3 wastewater is produced per m^3 of oil processed.[19] The pH ranges from 7.06 to 8.98. The TDS ranges from 4.79 to 26,997 mg/L. The TOC ranges from 0.24 to 4,551 mg/L. Limited data were found for background species. A few sources reported Fe and TIN level on the order of tens of mg/L, and only one dataset reported SO_4, Cl, and F, with F at a few mg/L and Cl and SO_4 reaching hundreds to around one thousand mg/L. However, given its similarity to PW, other background species such as Na, Ca, and TIC are also likely to be present. The concentration of CMs is typically below mg/L levels.

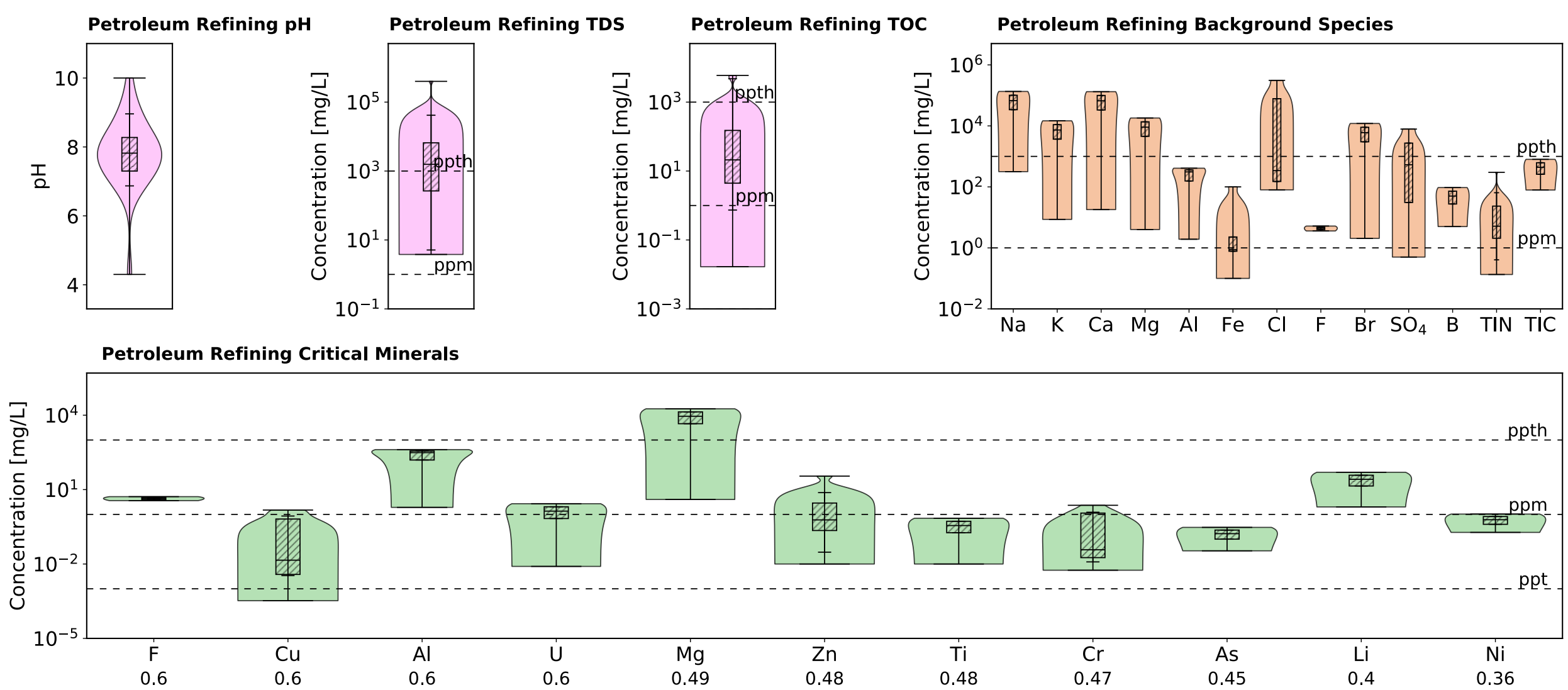

Figure S9. Petroleum refining pH, TDS, TOC, background species, and critical minerals concentration distribution. Only one concentration data was found for SO_4, Cl, and F, therefore these species are not presented in the background species figure.

Table S18. Petroleum refining wastewater characteristics.

Petroleum Refining					
Flow [m^3/m^3-oil produced]		pH		TDS [mg/L]	TOC [mg/L]
0.90–4.51 (2.70)		6.74–9.14 (7.82)		5.05–59,520 (1,565)	0.35–5,307 (21)
**Wastewater Constituents**					
**Background Species**	**Concentration [mg/L]**	**Median Mineral Proportion in TDS [%]**	**Critical Minerals**	**Concentration [mg/L]**	**Criticality Rating**
Na	7,033–127,935 (67,484)	41	Cu	0–1.31 (5.00×10^{-3})	0.6
Ca	6,651–126,054 (66,352)	41	F	3.67–5.12 (4.39)	0.6
Mg	911–17,238 (9,074)	5.55	Al	33–400 (310)	0.6
K	741–13,917 (7,329)	4.49	U	0.14–2.57 (1.35)	0.6
SO_4	6.58–6,826 (530)	0.32	Mg	911–17,238 (9,074)	0.49
Cl	94–264,053 (341)	0.21	Zn	0–24 (0.45)	0.48
Al	33–400 (310)	0.19	Ti	0.04–0.67 (0.35)	0.48
TIC	7.90–728 (79)	0.05	Cr	0–1.94 (0.04)	0.47
B	9.50–90 (50)	0.03	As	3.40×10^{-3}–0.27 (0.03)	0.45
F	3.67–5.12 (4.39)	2.69×10^{-3}	Ba	1,120–21,280 (11,200)	0.44
TIN	0.05–92 (3.86)	2.36×10^{-3}	Li	4.40–48 (26)	0.4
Br	0.20–10,800 (2.05)	1.25×10^{-3}	Mn	8.75–166 (88)	0.4
Fe	0–71 (0.78)	4.74×10^{-4}	Ni	0.23–0.99 (0.61)	0.36

S6.6 Data sources

Most of our data sources were from the U.S., with limited data from other countries including Nigeria, Romania, United Kingdom (U.K.), and China. We report characteristics of wastewater measured directly at the source (i.e., prior to any treatment or control), and we include all background species and CMs that were reported in the original data. Data reported as below detection limit are considered as zero.

Data on wastewater characteristics from drilling activities presented in **S6.3** were obtained from peer-reviewed literature[74,75,94,95] published between 2012 and 2015. Data on drilling wastewater generation rates come from a technical report by the American Petroleum Institute (API) [16], dated 2000.

Data on wastewater characteristics from produced water **(S6.4**) were obtained from the USGS National Produced Waters Geochemical Database (Ver. 3.0, December 2023)[88]. Data on produced water generation were obtained from technical reports from USGS[96], API[16], and ANL[17], and journal article[18], dated from 2000 to 2023.

Data on wastewater characteristics and generation from petroleum refineries was obtained from technical reports from USGS[19,97], U.S. Environmental Protection Agency (EPA)[90], and peer-reviewed literature[91,98–100], dated from 1964 to 2025.

S6.7 Data limitations

Variability of the data. The composition of PW is highly variable and depends strongly on the geological characteristics of the hydrocarbon reservoirs, as shown in **Figure S8**. Drilling wastewater also exhibits substantial variability due to differences in drilling-fluid formulations. [16] For example, depending on requirements on fluid chemistry and water availability, either freshwater or seawater may be used as the base fluid. Additional chemical additives are used to achieve desired fluid properties, such as Zn oxide to increase density and ammonium salts to stabilize the clay layers of the reservoir. Because most of the PW and drilling-wastewater data used in this study were obtained from U.S. sources, the reported compositions may not fully represent global wastewater characteristics.

Incomplete reporting of dissolved species. The databases used for PW and drilling wastewater are relatively comprehensive, containing 78 and 43 dissolved species, respectively. In contrast, fewer data were available for petroleum refining wastewater. Species commonly present at high concentrations in PW—such as sodium and calcium—are also expected to occur in refinery wastewater because these streams originate from processing the same hydrocarbon. However, among the seven refinery wastewater data sources included, none reported these species, likely because they are considered to be less relevant for toxicity studies.

Minerals from suspended solids. Our analysis focuses on dissolved species; however, suspended solids (SS) present in PW and drilling fluids may also contain recoverable CMs.[88,101] Particularly, drilling fluids typically contain substantial amounts of solids, comprising approximately 26.4% of total volume. PW generally contains much lower solids content, with total solids ranging from 0.09–6.06% and a median of 0.65%. Depending on wastewater management practices (e.g., storage in reserve pits), SS may dissolve into the aqueous phase over time, potentially increasing TDS and contributing to elevated CM concentrations.

Section S7. Metal Ore and Coal Mining

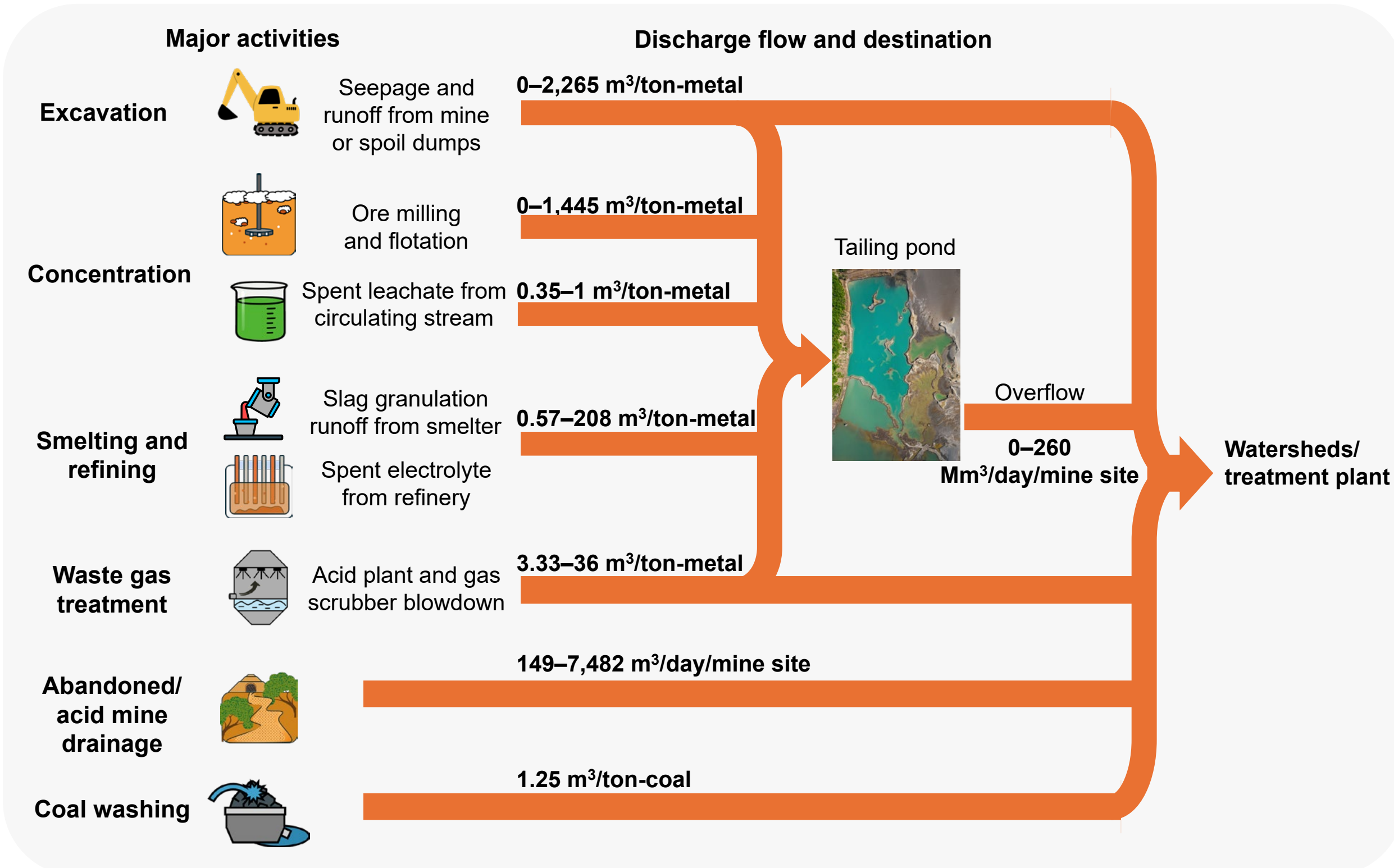

Figure S10. Flow diagrams from major mining activities and wastewater stream flow rate.

S7.1 Industry description

Mining is the process of excavating valuable minerals such as metals and coal from the earth. Wastewater is generated from the water use during mining activities,[102] as well as naturally occurring water such as precipitation and groundwater seepage that come into contact with mining sites.[103] These wastewaters contain metals that are not recovered during the mining activities, which include background ions and CMs.

Metal ore mining involves (1) extracting valuable metal-bearing ores from the Earth's crust, (2) concentrating the metal from the main ore body (often referred as "beneficiation" or "dressing"), and (3) refining them to achieve high purity. In 2023, more than 493 million tons (Mt) of CMs were mined around the world.[104] In the U.S., the most produced minerals were Fe, Cu, Zn, and Pb, each with an annual production exceeding 100,000 tons.[105] Other minerals, including Au, Ag, Ni, Co, and rare-earth metals are produced in smaller quantities, often as byproducts of the mining operations of the most produced metals. From 2000 to 2017, more than 6,500 metal mines were active around the world.[46] In the U.S., approximately 208 ore mines were active as of 2022.[106] The number of active mines is likely to continue growing due to the increasing demand of CMs. For example, in 2024, three uranium mines opened in Arizona and Utah, and one lithium mine in Nevada received approval.[107–109]

Coal mining involves similar activities as metal mining, although with fewer processing steps. After excavation, coal is transported to coal preparation plants (CPP) for cleaning, which mainly involves crushing and washing.[110] As of 2024, global coal production reached 9,150 million tons, with major contributions from China (51.0%), India (11.8%) and Indonesia (9.1%).[44] In the

U.S., 465 million tons coal were produced in 2024. As of 2023, approximately 4,300 coal mines were active around the world,[111] and 560 coal mines were active in the U.S.[112] Despite growing efforts to transition to cleaner energy, coal mining remains significant in many regions as it remains one of the major sources of electric power.[113] In 2024, 70 new coal mines were established, and more than 500 additional coal mine projects are planned worldwide. [111]

In the following sections, we summarize the wastewater characteristics and annual wastewater volume for commonly practiced mining activities in metal mining. Due to data limitations, only mine drainage (e.g., acid mine drainage) is discussed for coal mining. Additionally, because the cause and compositions of coal mine and metal mine drainage share similarities, we refer to them collectively as "abandoned mine drainage." The presented data for metal mining aims to encompass most metal ore types that share similar mining activities and wastewater characteristics; therefore, they do not highlight the differences between various ore types. Specific types of metal ores that deviate from the common characteristics are discussed separately if information is available.

S7.2 Overview of metal mining wastewater streams

The wastewater generated from metal mining industries originates from ore processing, mine drainage, and stormwater run-off.[114] Specifically, ore processing comprises four major operations that generate distinct waste streams: (1) excavation, (2) concentration (milling and flotation or leaching), (3) smelting and refining, and (4) treatment of waste gases. **Figure S10** shows the various wastewater streams from mining activities. In many cases, these waste streams are combined and discharged into a "tailings pond," which may or may not subsequently release to surface waters. Depending on the site plan, wastewater from one or more of these activities may also be discharged directly into surface waters.

S7.3 Excavation wastewater

Excavation operations in mining typically involve removing the overburden (waste rock) to expose the valuable ore, then transporting the ore from the deposit to a processing plant for further concentration. Wastewater generated from excavation mainly comes from groundwater seepage or precipitation runoff, as well as the water sent to the mine for utility use (e.g., equipment cooling, dust control).

Table S19 and **Figure S11** show the characteristics of excavation wastewater.[5,6,115,116] The estimated wastewater discharge amount ranges from 0 to 2,265 m^3/ton-metal-produced. The pH ranges from 5.46 to 8.36. The TDS ranges from 64 mg/L to 16,710 mg/L. Major background species include Na, Ca, Mg, Fe, Cl, and SO_4, with SO_4 having the highest concentration, exceeding hundreds of mg/L. Major CMs include Mg, Zn, and Mn, with concentration in a few mg/L; other CMs are found at levels below 1 mg/L.

The annual wastewater volume depends on the climate and geology of the mine site, the mining method (e.g., underground, open-pit, etc.), and the waste management method on the mine site.[5] For mines in arid area, almost no runoff from excavation is observed, whereas for mines in high-precipitation areas, higher runoff rate is anticipated. The low pH in wastewater is caused by the oxidation of sulfide-bearing ores, especially the ones containing Fe sulfide minerals (e.g., pyrite).[116] However, because the oxidation process is often slow and neutralizing minerals (e.g., calcite) are present,[15] wastewater from active mines and those lacking oxidizing agents mostly have a neutral to slightly alkaline pH. The wastewater is high in sulfate because most of the

valuable ores are sulfur-bearing minerals. The water hardness is also high due to the dissolution of calcium and magnesium from minerals such as calcite and dolomite that are abundant in ores and in Earth's crust.[117,118]

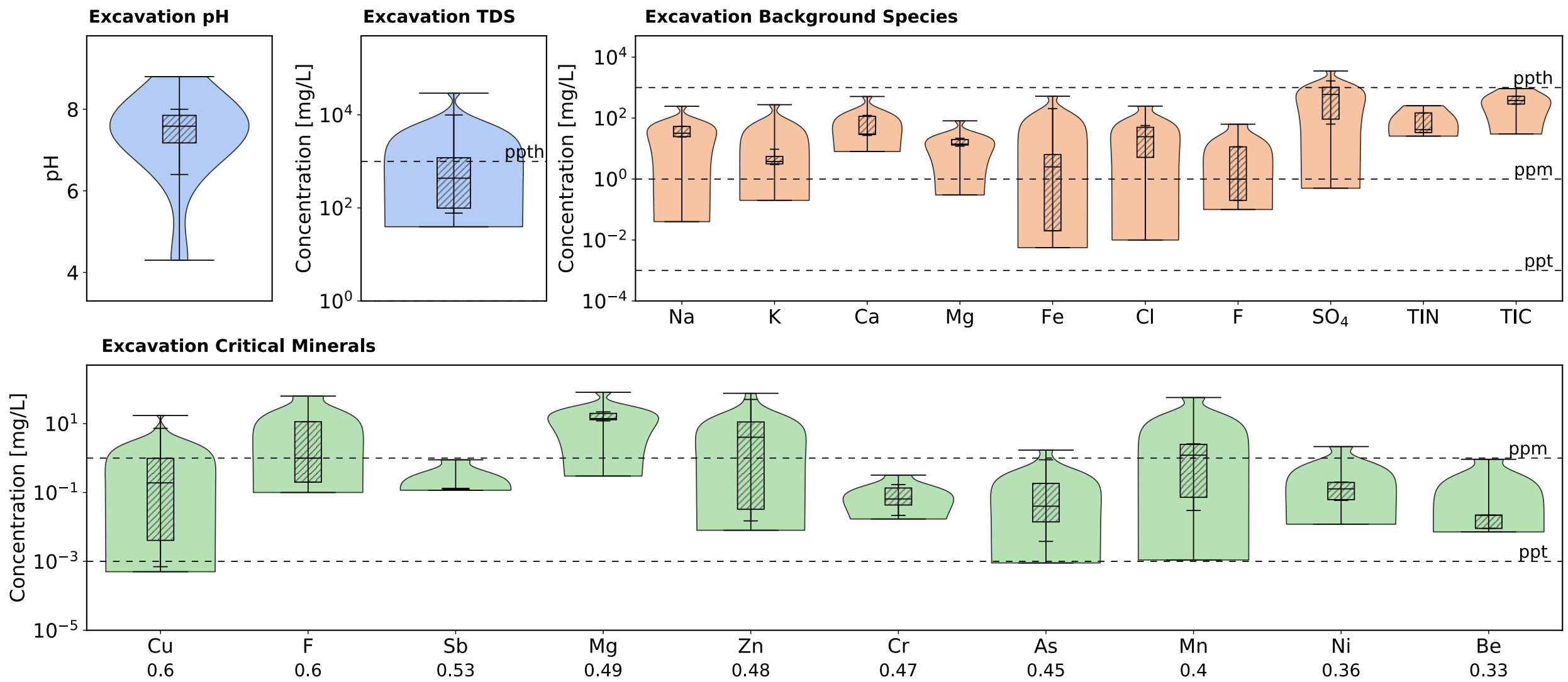

Figure S11. Excavation wastewater pH, TDS, background species, and critical minerals concentration distribution.

Table S19. Excavation wastewater characteristics. Values in parentheses represent the median concentrations; ranges show the 5th to 95th percentile.

Excavation					
Flow [m^3/ton-metal produced]		pH		TDS [mg/L]	TOC [mg/L]
0–2,265 (39)		5.46–8.36 (7.58)		64–16,710 (436)	N/A
**Wastewater Constituents**					
**Background Species**	**Concentration [mg/L]**	**Median Mineral Proportion in TDS [%]**	**Critical Minerals**	**Concentration [mg/L]**	**Criticality Rating**
SO_4	32–2,584 (597)	53	Cu	5.00×10^{-4}–7.30 (0.18)	0.6
TIC	82–847 (378)	33	F	0.12–53 (1.00)	0.6
TIN	27–233 (42)	3.68	Sb	0.12–0.74 (0.13)	0.53
Na	9.62–168 (32)	2.83	Mg	4.98–57 (14)	0.49
Ca	16–355 (30)	2.66	Zn	0.01–57 (4.03)	0.48
Cl	0.01–200 (24)	2.16	Cr	0.02–0.27 (0.07)	0.47
Mg	4.98–57 (14)	1.24	As	2.35×10^{-3}–1.29 (0.04)	0.45
K	1.18–182 (3.75)	0.33	Mn	8.32×10^{-3}–44 (1.21)	0.4
Fe	0.01–395 (2.50)	0.22	Ni	0.02–1.66 (0.13)	0.36
F	0.12–53 (1.00)	0.09	Be	7.54×10^{-3}–0.74 (0.02)	0.33

S7.4 Metal concentrating wastewater–milling and flotation

The proportion of valuable minerals in ore is often low (e.g., 0.6% to 2% for Cu and 2% to 10% for Pb and Zn). Therefore, a concentration operation is almost always employed to obtain a metal concentrate that is economical for subsequent purification steps (i.e., smelting and refining). Milling and flotation (hereafter referred to as flotation) is one of the most used methods for extracting valuable ores from the main ore body, and it generates the highest volume of wastewater.[119] Excavated ores are first milled and crushed to a fine size, then mixed with water containing a flotation agent (e.g., pH modifiers) and bubbled with compressed air to create froth. Milled ores that contain valuable minerals are attached to the froth and separated from the flotation water.

Figure S12 and **Table S20** show characteristics of the flotation wastewater.[5,7,120] The estimated wastewater volume from 0 to 1,445 m^3/ton-metal-produced. The pH ranges from 6.74 to 12. The TDS ranges from 243 to 5,497 mg/L. The TOC ranges from 9.77–889 mg/L. Within presented background species, Na, SO_4, and Si are the predominant ones exceeding hundreds of

mg/L. Major CMs are Cu, Si, Mg, and Zn, with concentrations reaching few hundred mg/L; other CMs are found at levels of a few mg/L.

Out of 103 mines reporting flotation discharge data, 71 mines (69%) perform full recycling, resulting in zero discharge flow rate as shown in **Table S20**. When flotation water becomes excessively saline or when its chemistry cannot be adjusted to achieve desired flotation performance, the water is discharged to the surface. The neutral to alkaline pH condition is from the addition of pH modifiers (e.g., lime, sodium carbonate) for enhancing flotation efficiency.[121,122] Alkaline conditions are generally preferred because they give minerals better stability and stronger electrostatic interactions with the froth.[123,124] The high TDS is mainly caused by the dissolution of minerals from finely milled ores and the addition of pH modifiers. Compared to excavation wastewater, flotation wastewater has elevated concentrations for all minerals, due to the exposure of minerals on the surfaces of finely milled ores. The presence of TOC in excavation wastewater is due to the addition of organic flotation agents (e.g., saturated hydrocarbons, polypropylene glycols, etc.). Note that most flotation agents are organic and do not introduce additional minerals to the processing water; however, certain chemicals such as $CuSO_4$ (mainly used in Pb-Zn ores for sphalerite activation)[125] may be used. In such cases an elevated concentration of the added mineral is expected.

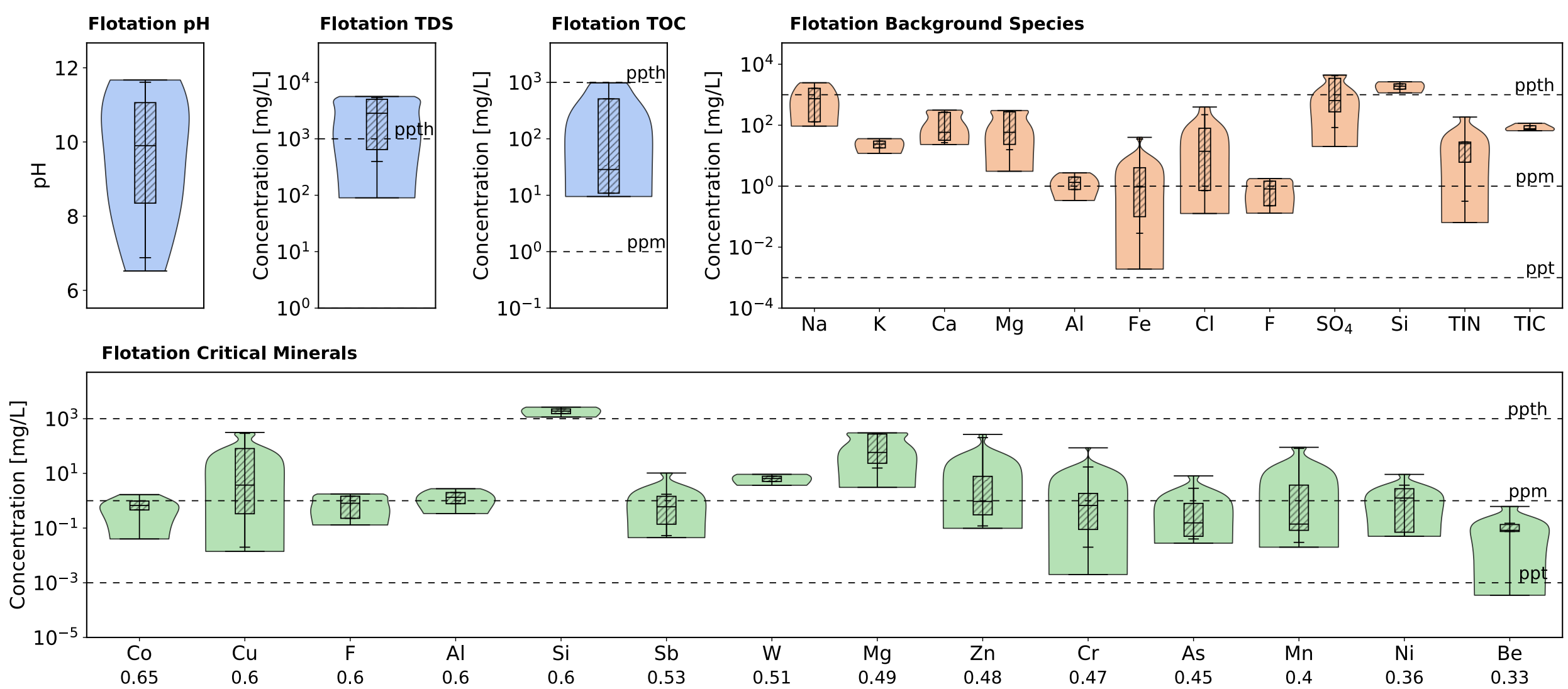

Figure S12. Milling and flotation (flotation) wastewater pH, TDS, TOC, background species, and critical minerals concentration distribution.

Table S20. Milling and flotation wastewater characteristics. Values in parentheses represent the median concentrations. The range represents the 5th to the 95th percentile.

Milling and Flotation					
Flow [m^3/ton-metal produced]		pH		TDS [mg/L]	TOC [mg/L]
0–1,445 (43)		6.74–12 (9.90)		243–5,497 (2,834)	9.77–889 (29)
**Wastewater Constituents**					
**Background Species**	**Concentration [mg/L]**	**Median Mineral Proportion in TDS [%]**	**Critical Minerals**	**Concentration [mg/L]**	**Criticality Rating**
Si	1,225–2,575 (1,900)	54	Co	0.12–1.54 (0.67)	0.65
Na	100–2,300 (741)	21	Cu	0.02–294 (3.73)	0.6
SO_4	62–4,270 (645)	18	Al	0.43–2.60 (1.33)	0.6
TIC	26–315 (76)	2.15	F	0.15–1.71 (0.81)	0.6
Mg	6.87–300 (58)	1.65	Sb	0.05–7.31 (0.60)	0.53
Ca	16–295 (58)	1.65	W	3.96–8.98 (6.47)	0.51
K	13–35 (24)	0.68	Mg	6.87–300 (58)	0.49
Cl	0.33–334 (14)	0.39	Zn	0.12–209 (0.94)	0.48
TIN	0.06–115 (7.42)	0.21	Cr	0.02–31 (0.67)	0.47
Al	0.43–2.60 (1.33)	0.04	As	0.03–6.00 (0.15)	0.45
Fe	0.03–35 (0.95)	0.03	Mn	0.03–85 (0.14)	0.4
F	0.15–1.71 (0.81)	0.02	Ni	0.05–6.73 (1.25)	0.36
			Be	0.02–0.50 (0.08)	0.33

S7.5 Metal concentrating wastewater–spent leachate

Leaching is a hydrometallurgical process that extracts and concentrates minerals from ore with chemicals such as acids and bases.[126] Leaching is usually done when flotation is economically or technically unfavorable. For example, low grade Cu ores (less than ~ 0.5% Cu), Cu oxides, Al, Au, and U are commonly extracted and concentrated through leaching. Additional concentrating steps such as electrowinning, cementation, and solvent extraction are usually performed in sequence with leaching. Although leaching solution is almost 100% recycled after concentrating and removing the minerals by the refinery, it eventually becomes so high in impurities that it must be discarded or regenerated, therefore contributing to overall waste discharge.

Figure S13 and **Table S21** show the characteristics of the leaching wastewater.[5,116,120,127,128] Wastewater discharged from leaching (often called "raffinate") ranges from 0.35 to 1.00 m^3/ton-metal-produced. Note that this wastewater accounts for the recycled portion with concentrating and refining steps (a.k.a., the wastewater is from the leach-concentrate-refine cycle). The pH of the leaching wastewater depends on the leaching chemical being used. For minerals like Cu and Zn, sulfuric acid is usually used for leaching, resulting in a pH range of 0.99 to 2.73; for Au and Al leaching, due to the use of sodium cyanide and sodium hydroxide, the pH can range from 9 to 10.[129,130] The TDS ranges from 10,487 to 51,615 mg/L. Major background species are Mg, Al, Fe and SO_4, with concentrations exceeding thousands of mg/L. CMs are found in concentrations ranging from a few to thousands of mg/L.

The high TDS and mineral concentrations can be attributed primarily to: 1) the enhanced mineral dissolution caused by the leaching chemicals, and 2) the accumulation of impurities from multiple cycles of recirculation.[128] The concentration of CMs is also high because the leaching-concentration-refining cycle dissolves most minerals but selectively removes specific ones. Minerals that have not historically been economically viable for extraction remain in wastewater.

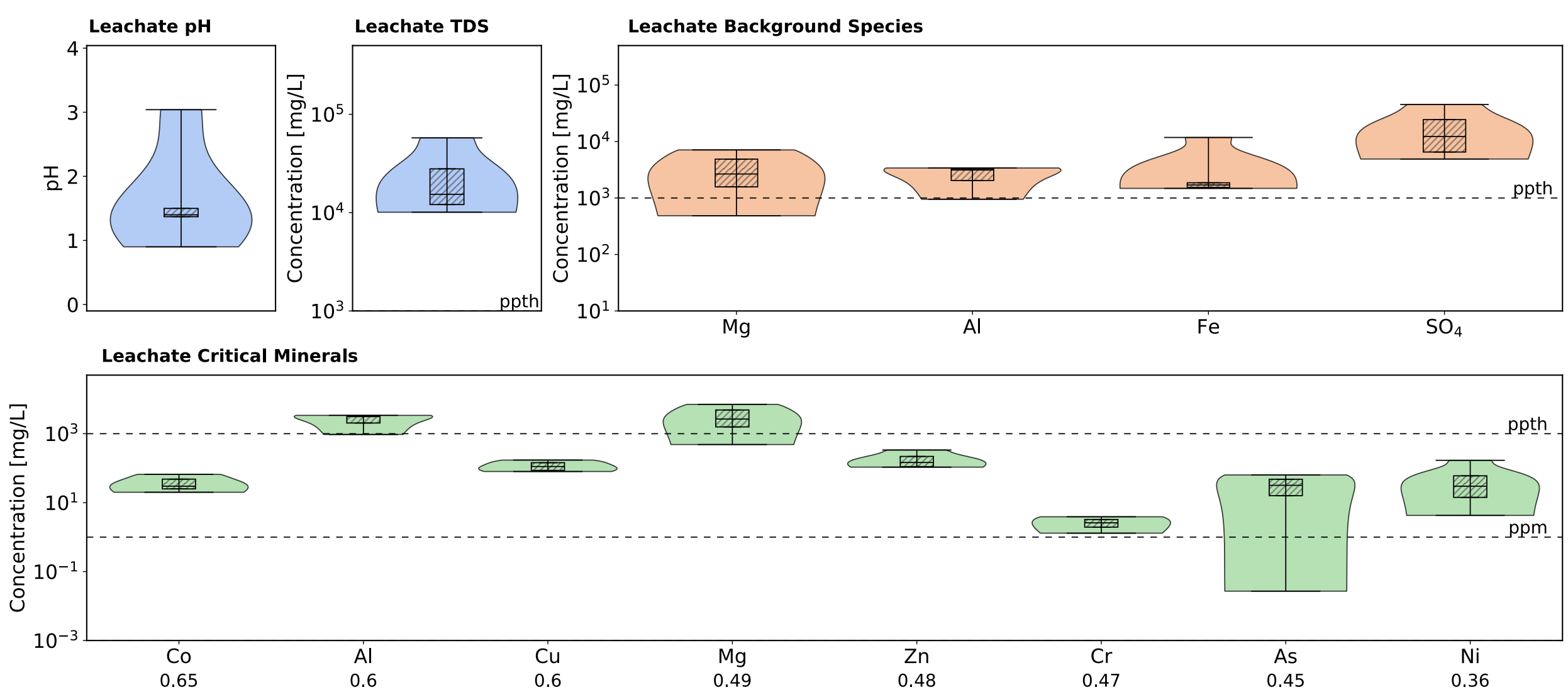

Figure S13. Spent leachate wastewater pH, TDS, background species, and critical minerals concentration distribution.

Table S21. Spent leachate characteristics. Values in parentheses represent the median concentrations. The data range represents the 5th to the 95th percentile.

Spent Leachate					
Flow [m^3/ton-metal produced]		pH		TDS [mg/L]	TOC [mg/L]
0.35–1.00 (0.66)		0.99–2.73 (1.40)		10,487–51,615 (15,357)	N/A
**Wastewater Constituents**					
**Background Species**	**Concentration [mg/L]**	**Median Mineral Proportion in TDS [%]**	**Critical Minerals**	**Concentration [mg/L]**	**Criticality Rating**
SO_4	5,223–41,045 (12,278)	61	Co	21–62 (30)	0.65
Al	1,168–3,373 (3,130)	16	Cu	81–166 (112)	0.6
Mg	703–6,656 (2,662)	13	Al	1,168–3,373 (3,130)	0.6
Fe	1,492–9,812 (1,700)	8.44	Mg	703–6,656 (2,662)	0.49
			Zn	108–313 (146)	0.48
			Cr	0.13–3.63 (1.30)	0.47
			As	3.21–60 (32)	0.45
			Ni	6.25–147 (30)	0.36
			Be	0.01–0.28 (0.15)	0.33

S7.6 Smelting and refining wastewater

Smelting and refining are the final steps of ore processing that bring valuable minerals to marketable purity. Smelting is a pyrometallurgical process that removes impurities from concentrated ore by applying high temperature. The only wastewater generated from smelting is from the overflow of slag granulation, a process where cold water is used to quench the molten slag separated from the metal, and the wash water for the fire-refined metals.[5] Refining operations are applied to concentrated leachate or to ore after smelting. A few different technologies including fire refining and electrolytic refining are used. The wastewater generated is mainly from the spent electrolyte from electrolytic refining.

Figure S14 and **Table S22** show the characteristics of the smelting and refining wastewater.[5,116] Wastewater discharge rates ranged from 0.57 to 208 m^3/ton-metal-produced wastewater. Major background species were Ca and SO_4, with concentrations from tens to hundreds of mg/L. The major CMs were Mg, Zn, Cu, and Ni, with concentration reaching tens of mg/L; other CMs were typically below mg/L level. The pH ranged from 4.85 to 8.52. The TDS ranged from 108 to 27,600 mg/L. Only one study reported a TOC of 560 mg/L.

Because electrolytic refining highly resembles electrowinning in after a leaching operation, the wastewater shares similar characteristics as spent leachate, resulting in low pH and high TDS wastewater. However, the CM content is low because of the efficient recovery of metals.

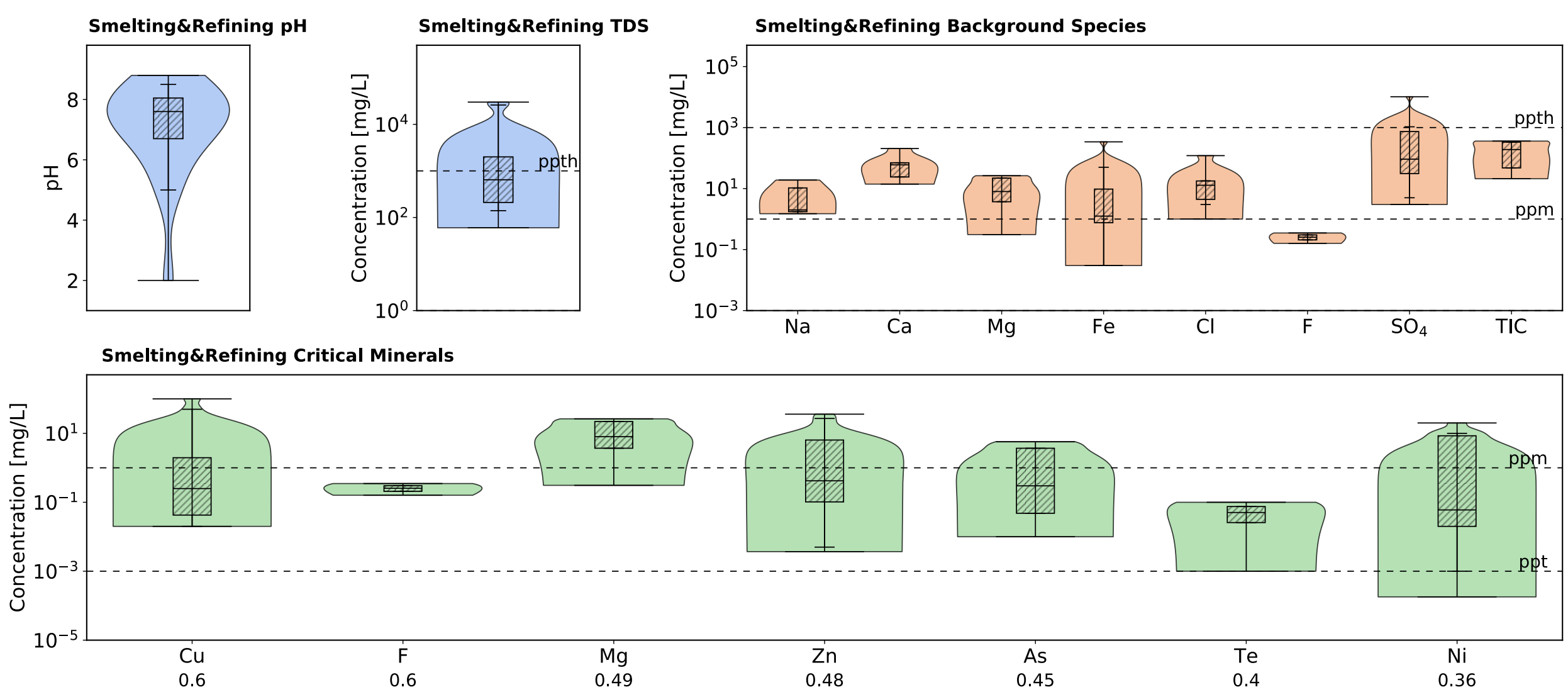

Figure S14. Smelting and refining wastewater pH, TDS, background species, and critical minerals concentration distribution. The TOC distribution is not shown because there are only two data points.

Table S22. Smelting and refining wastewater characteristics. Values in parentheses represent the median concentrations. The range represents the 5th to the 95th percentile.

Smelting and Refining					
**Flow [m^3/ton-metal produced]**		**pH**		**TDS [mg/L]**	**TOC [mg/L]**
0.57–208 (27)		4.85–8.52 (7.60)		108–27,600 (645)	155–539 (347)
**Wastewater Constituents**					
**Background Species**	**Concentration [mg/L]**	**Median Mineral Proportion in TDS [%]**	**Critical Minerals**	**Concentration [mg/L]**	**Criticality Rating**
TIC	13–349 (112)	39	F	0.17–0.34 (0.26)	0.6
SO_4	4.10–5,020 (77)	27	Cu	0–67 (0.14)	0.6
Ca	9.32–162 (65)	23	Mg	0.99–26 (8.00)	0.49
Cl	1.40–100 (17)	5.98	Zn	4.61×10^{-3}–30 (0.44)	0.48
Mg	0.99–26 (8.00)	2.82	As	0.02–5.30 (0.30)	0.45
Na	1.55–17 (2.00)	0.70	Te	5.95×10^{-3}–0.10 (0.05)	0.4
Fe	0.03–195 (1.20)	0.42	Ni	6.72×10^{-4}–14 (0.06)	0.36
F	0.17–0.34 (0.26)	0.09			

S7.7 Waste gas treatment wastewater

Waste gases are generated during pyrometallurgical operations such as smelting and fire refining, and other high temperature hydrometallurgical operations. The waste gas is typically high in particulates and SO_2 since most ores are sulfur-bearing. For waste gas with a SO_2 proportion above 4%, catalyst beds are used to capture the SO_2 and produce marketable sulfuric acid on-site, and minimal wastewater is generated.[5] Waste gases with no marketable value are treated with gas scrubbers for air pollution control. Wet scrubbers that use water or water-alkaline chemicals (e.g., limestone) to capture the waste gas are the major sources of wastewater.

Figure S15 and **Table S23** shows characteristics of waste gas treatment wastewater.[5,6] Wastewater was generated at rates ranging from 3.33 to 36 m^3/ton-metal-produced. Only Fe and SO_4 were reported as background species; however, SO_3 should also be present due to the dissolution of SO_2 in water. Moreover, Na, Ca, and TIC are also likely to be present at high concentrations if alkaline chemicals such as limestone and soda are used in gas scrubbers. Note that in Al processing, high fluoride concentrations (13–150 mg/L) were found in scrubber wastewater due to the use of cryolite (Na_3AlF_6) during the refining process[131]; however, because fluoride was not reported for any other mines types, it was excluded from the presented data. CMs had a wide concentration range, and some minerals such as Zn and As had high concentration reaching tens to hundreds of mg/L. The pH ranged from 1.83 to 5.47. The TDS was very high, ranging from 17,640–47,997 mg/L.

The dissolution of waste SO_2 in water produces sulfurous acid that leads to low pH, and the use of alkaline chemicals leads to high TDS. The presence of CMs can be attributed to the dissolution of particulates in the waste gases that contain unrecovered metals from prior refining steps.

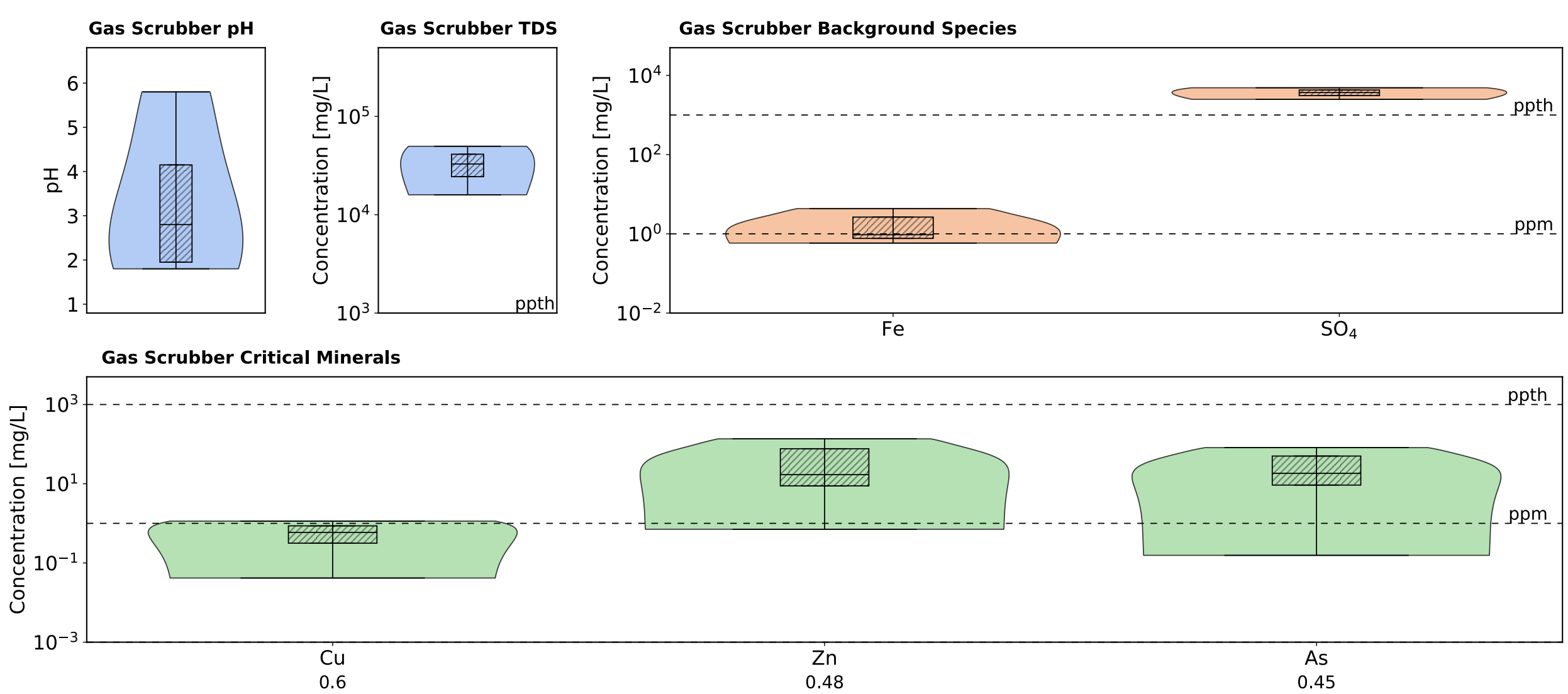

Figure S15. Gas scrubber wastewater pH, TDS, background species, and critical minerals concentration distribution.

Table S23. Waste gas treatment wastewater characteristics. Values in parentheses represent the median concentrations. The presented data range is from the 5th to the 95th percentile.

Waste Gas Treatment					
Flow [m^3/ton-metal produced]		pH		TDS [mg/L]	TOC [mg/L]
3.33–36 (20)		1.83–5.47 (2.80)		17,640–47,997 (32,818)	N/A
**Wastewater Constituents**					
**Background Species**	**Concentration [mg/L]**	**Median Mineral Proportion in TDS [%]**	**Critical Minerals**	**Concentration [mg/L]**	**Criticality Rating**
SO_4	2,609–4,748 (3,679)	99	Cu	4.17×10^{-3}–1.03 (0.04)	0.6
Fe	0.62–4.00 (0.95)	0.03	Zn	2.33–124 (17)	0.48
			As	1.97–75 (18)	0.45

S7.8 Overflow wastewater from tailings ponds

Around 70% of wastewater generated from the previously discussed operations is combined and discharged into a tailings pond, where it is held for recycling and evaporation, and any overflows are discharged to local wastewater treatment plants or surface watersheds.

Figure S16 and **Table S24** show the characteristics of tailings pond wastewater. The pH ranged from 3.2 to 11, while the TDS had a wide distribution that ranged from 101 to 120,584 mg/L.[29] The TOC ranged from 1.67 to 44 mg/L. Similar to excavation wastewater, the major reported background species were Na, Mg, Ca, Cl, and SO_4 with concentrations reaching few hundreds of mg/L. The major CMs were Mg and Zn, with concentrations reaching tens of mg/L; other CMs were reported at the mg/L level.

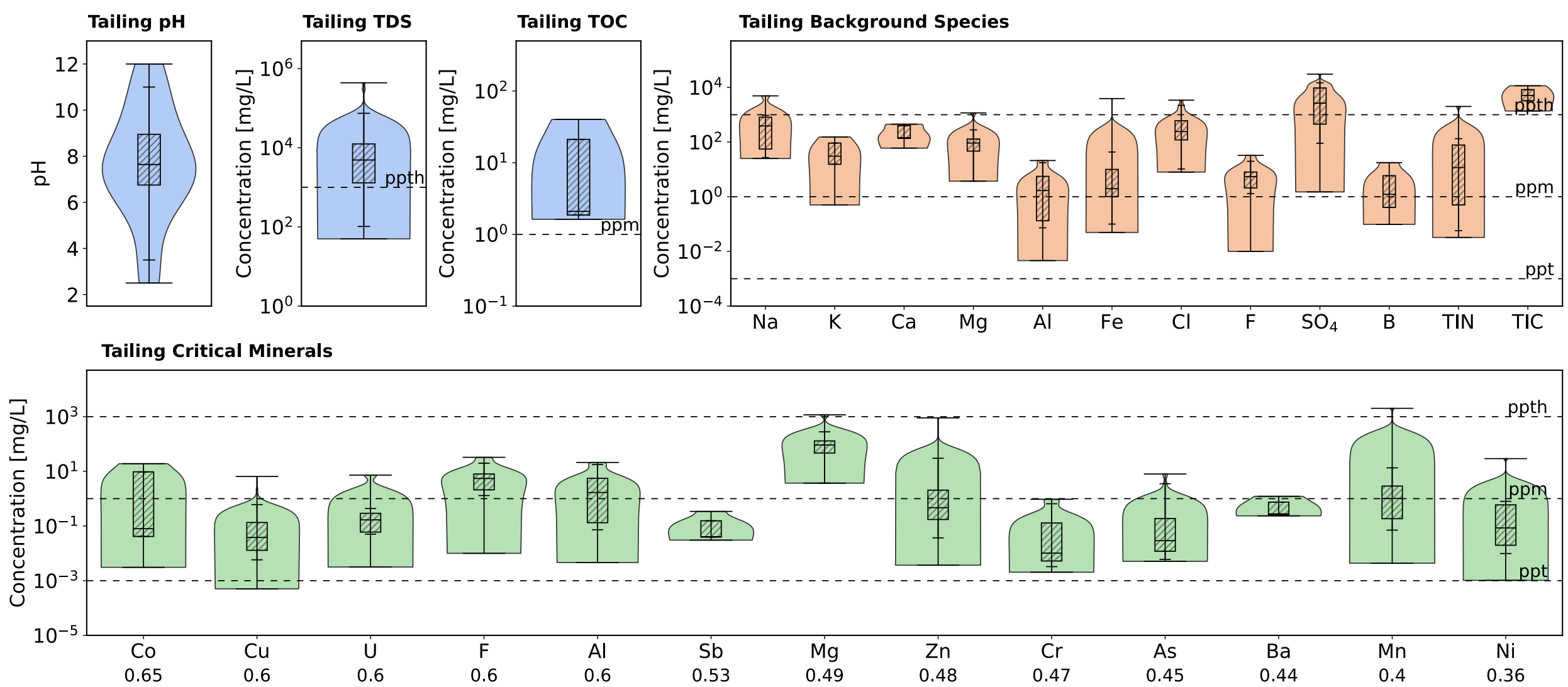

Figure S16. Tailing wastewater pH, TDS, TOC, background species, and critical minerals concentration distribution.

Table S24. Tailings pond wastewater characteristics. Values in parentheses represent the median concentrations. The range represents the 5th to the 95th percentile.

<table>
<tr><th colspan="6">Tailing</th></tr>
<tr><th colspan="2">Flow [m^3/day/site]</th><th colspan="2">pH</th><th>TDS [mg/L]</th><th>TOC [mg/L]</th></tr>
<tr><td colspan="2">0–260,247
(224)</td><td colspan="2">3.20–11
(7.64)</td><td>101–120,584
(4,906)</td><td>1.67–36
(2.10)</td></tr>
<tr><th colspan="6">Wastewater Constituents</th></tr>
<tr><th>Background Species</th><th>Concentration [mg/L]</th><th>Median Mineral Proportion in TDS [%]</th><th>Critical Minerals</th><th>Concentration [mg/L]</th><th>Criticality Rating</th></tr>
<tr><td>SO_4</td><td>70–15,007
(2,652)</td><td>62</td><td>Co</td><td>0.01–17
(0.08)</td><td>0.65</td></tr>
<tr><td>TIC</td><td>279–9,585
(700)</td><td>16</td><td>Cu</td><td>4.87×10^{-3}–0.76
(0.04)</td><td>0.6</td></tr>
<tr><td>Na</td><td>26–3,513
(390)</td><td>9.09</td><td>Al</td><td>0.05–19
(1.68)</td><td>0.6</td></tr>
<tr><td>Cl</td><td>10–2,201
(242)</td><td>5.64</td><td>U</td><td>0.02–4.89
(0.17)</td><td>0.6</td></tr>
<tr><td>Ca</td><td>75–440
(144)</td><td>3.36</td><td>F</td><td>0.53–27
(5.47)</td><td>0.6</td></tr>
<tr><td>Mg</td><td>19–767
(101)</td><td>2.36</td><td>Sb</td><td>0.03–0.30
(0.04)</td><td>0.53</td></tr>
<tr><td>K</td><td>3.48–141
(30)</td><td>0.71</td><td>Mg</td><td>19–767
(101)</td><td>0.49</td></tr>
<tr><td>TIN</td><td>0.07–131
(15)</td><td>0.35</td><td>Zn</td><td>0.03–33
(0.47)</td><td>0.48</td></tr>
<tr><td>F</td><td>0.53–27
(5.47)</td><td>0.13</td><td>Cr</td><td>2.65×10^{-3}–0.80
(0.01)</td><td>0.47</td></tr>
<tr><td>Fe</td><td>0.10–49
(1.98)</td><td>0.05</td><td>As</td><td>6.00×10^{-3}–4.81
(0.03)</td><td>0.45</td></tr>
<tr><td>Al</td><td>0.05–19
(1.68)</td><td>0.04</td><td>Ba</td><td>0.24–1.12
(0.28)</td><td>0.44</td></tr>
<tr><td>B</td><td>0.16–15
(1.23)</td><td>0.03</td><td>Mn</td><td>0.01–34
(1.02)</td><td>0.4</td></tr>
<tr><td></td><td></td><td></td><td>Ni</td><td>4.88×10^{-3}–0.80
(0.09)</td><td>0.36</td></tr>
</table>

S7.9 Abandoned / acid mine drainage (AMD)

Wastewater from abandoned coal and metal mines is primarily generated by the infiltration of groundwater and precipitation, which leaches through mine sites and eventually makes its way into surrounding surface water bodies.[132,133] Acid mine drainage (AMD) is the most environmentally significant mine wastewater with high metal concentrations and acidic pH due to the geochemical and microbial reactions of oxygen and water with pyrite (i.e., Fe disulfide).[134]

Figure S17 and **Table S25** show the characteristics of abandoned mine drainage.[135–138] The flow rate from a single drainage site ranged from 149 to as high as 7,482 m^3/day, depending heavily on the site's size, meteorological, and geological characteristics.[30,31] The pH ranged from 2.57 to 7.71. The TDS ranged from 309 to 21,165 mg/L. The TOC ranged from 1.10 to 160 mg/L. The major background species were Na, Mg, Cl, and SO_4, with concentrations reaching thousands of mg/L. The major CMs included Cu, Al, Si, Mg, Zn, and Mn with concentrations of tens to thousands of mg/L; other CMs were found below mg/L level.

The pH of AMD is influenced by numerous environmental factors, ranging from mine characteristics to surrounding water and soil chemistry.[139] For example, newly abandoned mines with less sulfur content in the mined ores are likely to have pH close to neutral;[140] the presence of Fe, oxygen, and chemotrophic microbials can accelerate the formation of AMD, resulting in low pH.[141] This low-pH condition increases metal solubility, increasing the TDS.[137] Both metal and coal mine waste contribute to the CMs in mine drainage. Coal typically contains mineral components comprising up to 20% of its weight, including background species such as Fe, S, Ca, and Si, as well as CMs such as As, Co, Li, and Mn.[142] Note that the composition of these minerals in coals varies significantly depending on coal types (e.g., anthracite, bituminous, subbituminous, and lignite) and geographical origin.[137]

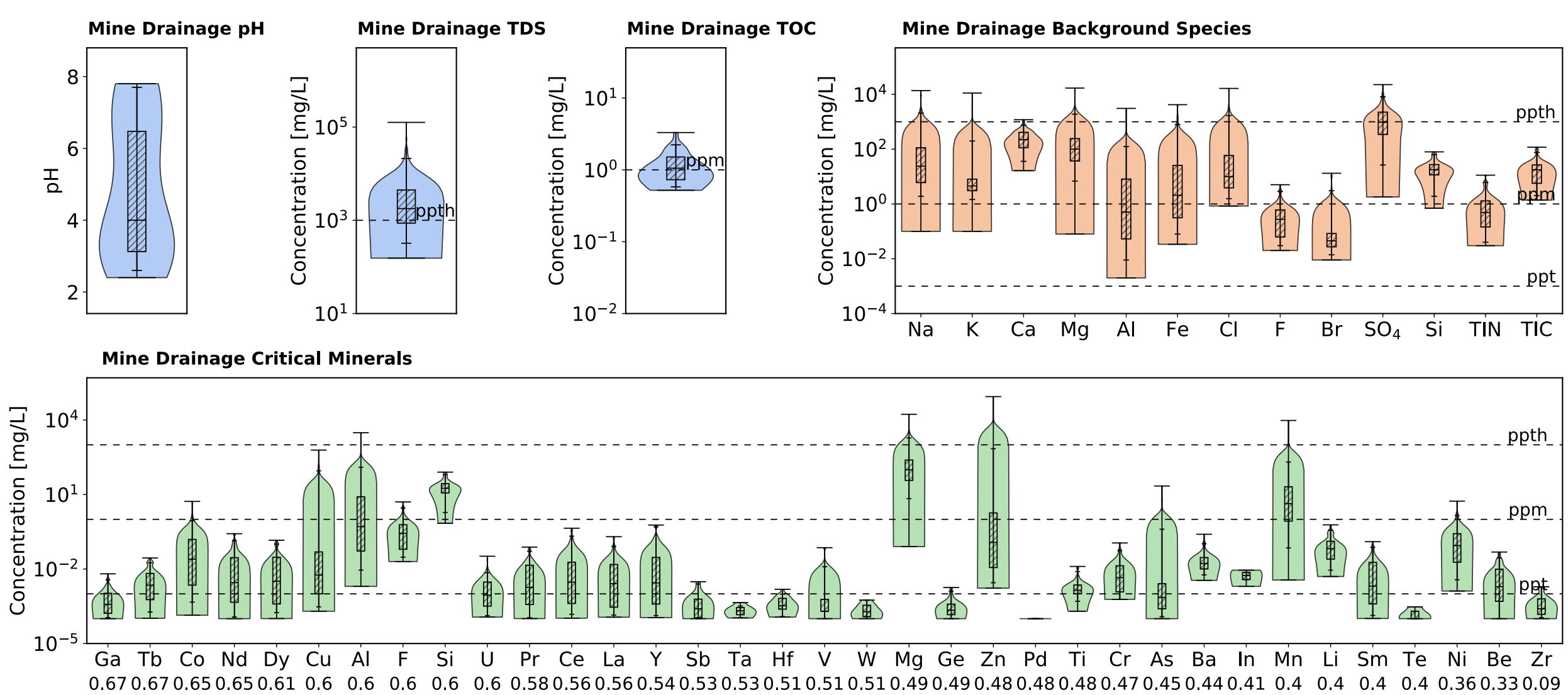

Figure S17. Mine drainage wastewater pH, TDS, TOC, background species, and critical minerals concentration distribution.

Table S25. Abandoned / acid mine drainage wastewater characteristics. Values in parentheses represent the median concentrations. The range represents the 5th to the 95th percentile.

Mine Drainage					
Flow [m^3/day/mine site]		pH		TDS [mg/L]	TOC [mg/L]
149–7,482 (1,191)		2.57–7.71 (4.00)		309–21,165 (1,782)	0.55–2.29 (1.04)
Wastewater Constituents					
Background Species	Concentration [mg/L]	Median Mineral Proportion in TDS [%]	Critical Minerals	Concentration [mg/L]	Criticality Rating
SO_4	25–8,148 (962)	70	Ga	1.00×10^{-5}–2.25×10^{-3} (1.45×10^{-4})	0.67
Ca	36–752 (222)	16	Nd	5.60×10^{-6}–0.13 (5.07×10^{-4})	0.65
Mg	6.66–2,000 (100)	7.30	Co	4.14×10^{-4}–0.89 (0.03)	0.65
Na	1.90–2,112 (24)	1.75	Al	8.70×10^{-3}–129 (0.51)	0.6
Si	1.76–68 (18)	1.30	Cu	2.80×10^{-4}–91 (5.70×10^{-3})	0.6
TIC	1.92–84 (18)	1.30	U	1.53×10^{-5}–7.02×10^{-3} (4.60×10^{-4})	0.6
Cl	1.48–1,761 (9.97)	0.73	F	0.03–2.86 (0.28)	0.6
K	1.43–202 (4.54)	0.33	La	8.80×10^{-6}–0.07 (3.75×10^{-4})	0.56
Fe	0.08–987 (2.08)	0.15	Ce	1.26×10^{-5}–0.21 (7.68×10^{-4})	0.56
Al	8.70×10^{-3}–129 (0.51)	0.04	Y	1.90×10^{-5}–0.48 (1.28×10^{-3})	0.54
F	0.03–2.86 (0.28)	0.02	Sb	1.00×10^{-5}–8.18×10^{-4} (2.00×10^{-5})	0.53
TIN	0.03–6.05 (0.26)	0.02	V	1.00×10^{-4}–0.02 (2.00×10^{-4})	0.51
Br	0.01–3.19 (0.04)	3.30×10^{-3}	W	2.00×10^{-5}–4.98×10^{-4} (5.00×10^{-5})	0.51
			Mg	6.66–2,000 (100)	0.49
			Ti	5.00×10^{-4}–8.70×10^{-3} (1.40×10^{-3})	0.48
			Zn	2.69×10^{-3}–797 (0.12)	0.48
			Pd	1.00×10^{-5}–1.00×10^{-4} (2.00×10^{-5})	0.48
			Cr	6.00×10^{-4}–0.06 (4.50×10^{-3})	0.47
			As	1.00×10^{-4}–0.40 (6.80×10^{-4})	0.45
			Ba	5.59×10^{-3}–0.11 (0.02)	0.44
			In	1.00×10^{-6}–8.57×10^{-4} (1.00×10^{-5})	0.41
			Li	8.60×10^{-3}–0.36 (0.07)	0.4
			Mn	0.06–215 (4.28)	0.4
			Te	1.00×10^{-4}–2.80×10^{-4} (1.00×10^{-4})	0.4
			Ni	3.58×10^{-3}–1.46 (0.09)	0.36
			Be	1.00×10^{-4}–0.03 (1.95×10^{-3})	0.33
			Zr	1.00×10^{-5}–5.91×10^{-4} (3.00×10^{-5})	0.09

S7.10 Coal washing

Coal washing wastewater (also called coal washery effluent) is a byproduct of the coal cleaning process, mostly generated in coal preparation or coal washing plants.[143,144] The coal washing process is essential to removing impurities like sulfur, thereby improving combustion efficiency and reducing potential air pollution.[145] Typically, 1.5-2.5 m^3 of water is required to process each ton of the raw coal, generating approximately 1.0-1.5 m^3 of wastewater.[28] Since coal washing process extracts impurities from the coal and utilizes washing agents (e.g., detergents), it often carries small coal particles and CMs embedded in coal such as Cu, Pb, and Al.[146]

Figure S18 and **Table S26** show the characteristics of coal washing wastewater.[143,145,147–152] around 1.25 m^3 of wastewater is generated to process every ton of the coal, and the major reported background ions were SO_4, Cl, and Ca with concentrations reaching a few hundred mg/L. The major CMs included Mg, Zn, Al, Cu, and Mn with concentrations on the order of mg/L; other CMs were found at lower concentrations. The pH ranged from 0.81 to 7.93. The TDS ranged from 137 to 20,250 mg/L. The TOC ranged from 0.82 to 4.06 mg/L.

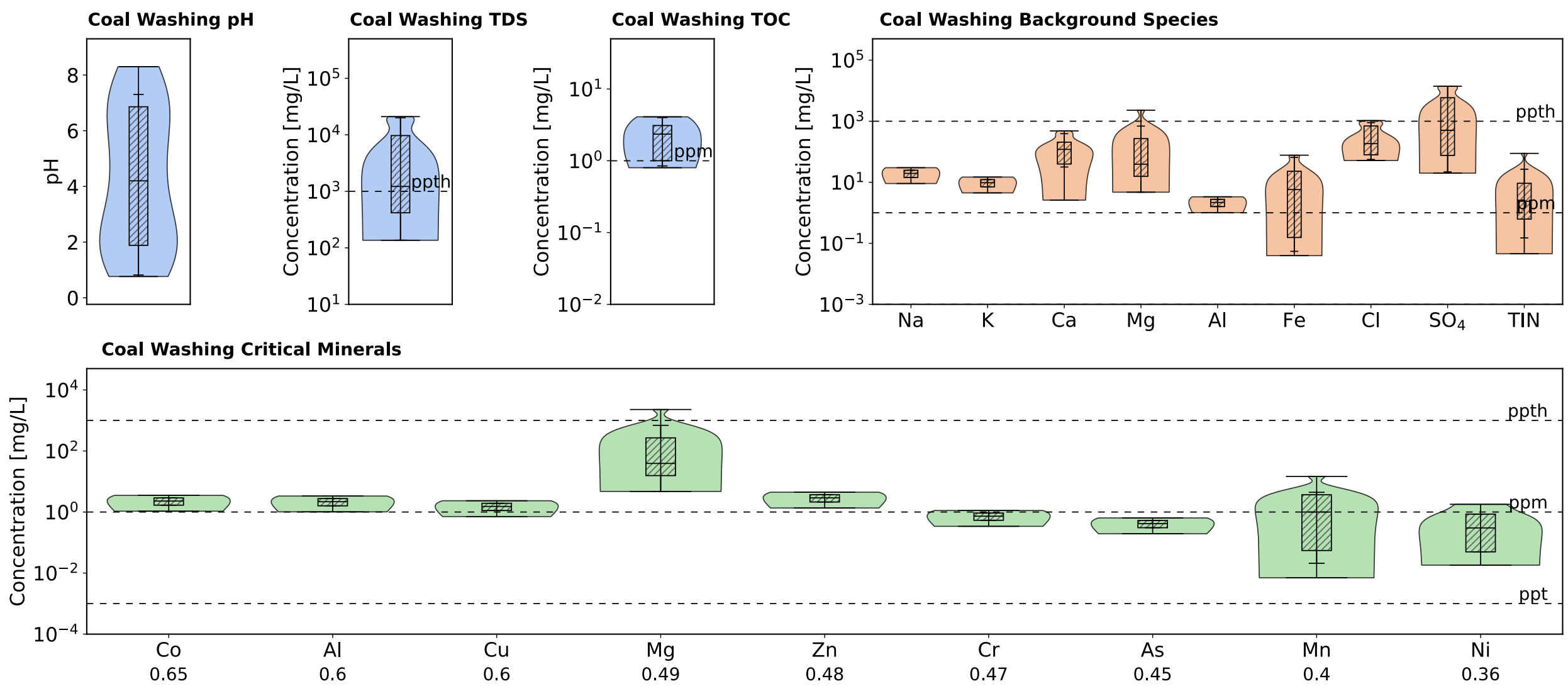

Figure S18. Coal washing wastewater background species and critical minerals concentration distribution.

Table S26. Coal washing wastewater characteristics. Values in parentheses represent the median concentrations. The range represents the 5th to the 95th percentile.

Coal Washing					
Flow [m^3/ton-coal processed]		pH		TDS [mg/L]	TOC [mg/L]
1.25		0.81–7.93 (4.20)		137–20,250 (1,220)	0.82–4.06 (2.35)
**Wastewater Constituents**					
**Background Species**	**Concentration [mg/L]**	**Median Mineral Proportion in TDS [%]**	**Critical Minerals**	**Concentration [mg/L]**	**Criticality Rating**
SO_4	21–13,925 (502)	54	Co	0–3.14 (0.53)	0.65
Cl	55–932 (184)	20	Al	1.13–3.22 (2.18)	0.6
Ca	24–413 (119)	13	Cu	0.79–2.26 (1.52)	0.6
Mg	4.73–1,567 (39)	4.23	Mg	4.73–1,567 (39)	0.49
Na	10–29 (20)	2.11	Zn	1.51–4.30 (2.91)	0.48
K	5.00–14 (9.64)	1.04	Cr	0.38–1.08 (0.73)	0.47
Fe	0.05–69 (5.76)	0.62	As	0.22–0.62 (0.42)	0.45
Al	1.13–3.22 (2.18)	0.24	Mn	0.01–10 (1.00)	0.4
TIN	2.00×10^{-3}–24 (0.17)	0.02	Ni	0.02–1.60 (0.30)	0.36

S7.11 Data sources

The metal mines included in our data sources are Cu, Zn, Pb, Au, Fe, Al, Hg, Au, Ag, Mo, and W. Most of our data sources were from the U.S., with limited data from other countries including Ireland, Spain, Brazil, Chile, and China. We report characteristics of wastewater measured directly at the source (i.e., prior to any treatment or control), and we include all background species and CMs that were reported in the original data, including those below the detection limit.

Data on wastewater characteristics from mining activities presented in **S7.3–S7.7** including excavation, milling and flotation, leaching, smelting and refining, and waste gas treatment, were primarily obtained from technical reports published by the U.S. EPA and from peer-reviewed literature.[5–7,102,114–116,153,154] These reports are dedicated to identifying major water pollution problems associated with mining industry activities and providing supporting information for establishing wastewater discharge guidelines. The earliest available report dates to 1973 and the most recent to 2011, with most data collected between the 1970s and 1990s. The journal articles were published between 1988 and 2022.

Data on tailings pond overflow wastewater (**S7.8**) were primarily obtained from the Discharge Monitoring Report Database (DMR) database[29], peer-reviewed literature,[140,155,156] and

the aforementioned EPA reports.[116,157] We interpreted the major wastewater discharge reported in the DMR as tailings pond overflow, since waste streams from upstream ore processing activities are usually directed to the tailings pond, making its overflow the primary source of wastewater discharge. The DMR database was dated 2024. The journal articles were dated from 2005 to 2022.

Data on abandoned mine drainage wastewater (**S7.9**) was primarily obtained from the NEWTS coal mine drainage dataset[158], peer-reviewed literature,[135,136,159,160] and the aforementioned reports.[30,116] After comparing the wastewater characteristics of coal mine drainage and metal mine drainage, no significant differences were observed; therefore, we did not distinguish between these two sources. The NEWTS dataset was dated 2015. The journal articles were dated from 2007 to 2022.

Data on coal washing wastewater (**S7.10**) was primarily obtained from peer-reviewed literature published between 2006 and 2025.[143,145,147–152] These data were collected from coal washing process, coal preparation plant, and coal washery plants.

S7.12 Data limitations

Age of the available data. Most of our U.S. data sources date to the late 20th century; therefore, discrepancies may exist compared with current wastewater characteristics if different mining practices are used. Comparing the industrial activity descriptions between the earliest referenced report in 1973[157] and the most recent report in 2024[161], the water recycling rate increased from around 22% to as high as 80%. The metal extraction and recovery efficiency had also improved due to the development of hydrometallurgy.[162] For example, the widespread adoption of electrolytic refining for Cu, high-pressure leaching for nickel [163] and high temperature leaching for platinum[164] have achieved recovery efficiencies over 99%, much higher than conventional methods that yielded only around 80% recovery.[165]

Incomplete reporting of dissolved species. Many CMs and background species were not reported in the source data, either because they were not of interest (for example, not relevant to monitoring regulatory compliance) and/or difficult to measure due to the limitations of detection technology. Therefore, we caution readers that the absence of a particular CM in our analysis may not necessarily mean it is absent from that wastewater. In some cases, it is possible to infer the presence of CMs in a mining wastewater based on the elemental composition of the metal and coal deposits (although we have not attempted to do so here). For example, some Cu sulfide ores in Nigeria contain CMs such as Ni, Ti, and Nd, which are present at levels on the order of 0.01 wt%.[166] Traces of these minerals should be present in the wastewater after Cu recovery, but were not reported.

Minerals from suspended solids (SS). Data presented in our study only includes dissolved species. However, wastewater from mining also contains a significant number of SS that may also carry CMs, particularly in flotation and tailings pond wastewater.[154] Recovering CMs from suspended solids likely requires pyrometallurgy or hydrometallurgy, following procedures similar to current mining practices[155]; therefore, we do not include SS in our discussions. However, it is worth noting that SS in tailings ponds may eventually dissolve due to changes in water chemistry over time, resulting in time-dependent concentration increases of CMs and environmental species in the pond.[156]

Section S8. Manufacturing

Global manufacturing output has risen steadily since the 1970s, as evidenced by the average annual growth rate of 3.8% between 1997 and 2022, contributing 13.8–17.5% of the global GDP, driven by industrial globalization and advances in electronics, automotive, and consumer goods production.[167] Such growth has been accompanied by increasing wastewater generation, arising from processing water, cleaning operations, solvent and electrolyte use, and chemical reagents. Here, we compiled the available data from five industries known to generate wastewater with CMs and has relatively large quantities—1) semiconductor manufacturing, 2) leather tanning, 3) metal plating and finishing, 4) battery manufacturing, and 5) lithium-ion battery (LIB) recycling.

S8.1 Semiconductor manufacturing wastewater

Semiconductor fabrication involves processing silicon wafers through oxidation, photolithography, etching, deposition, metallization, chemical mechanical polishing/planarization (CMP), and packaging, as illustrated in **Figure S18**. Wastewater generated during fabrication comprises the ultrapure water used for rinsing between processing steps and the chemical solutions used for cleaning and etching (**Figure S18,** numbers in circles**)**.

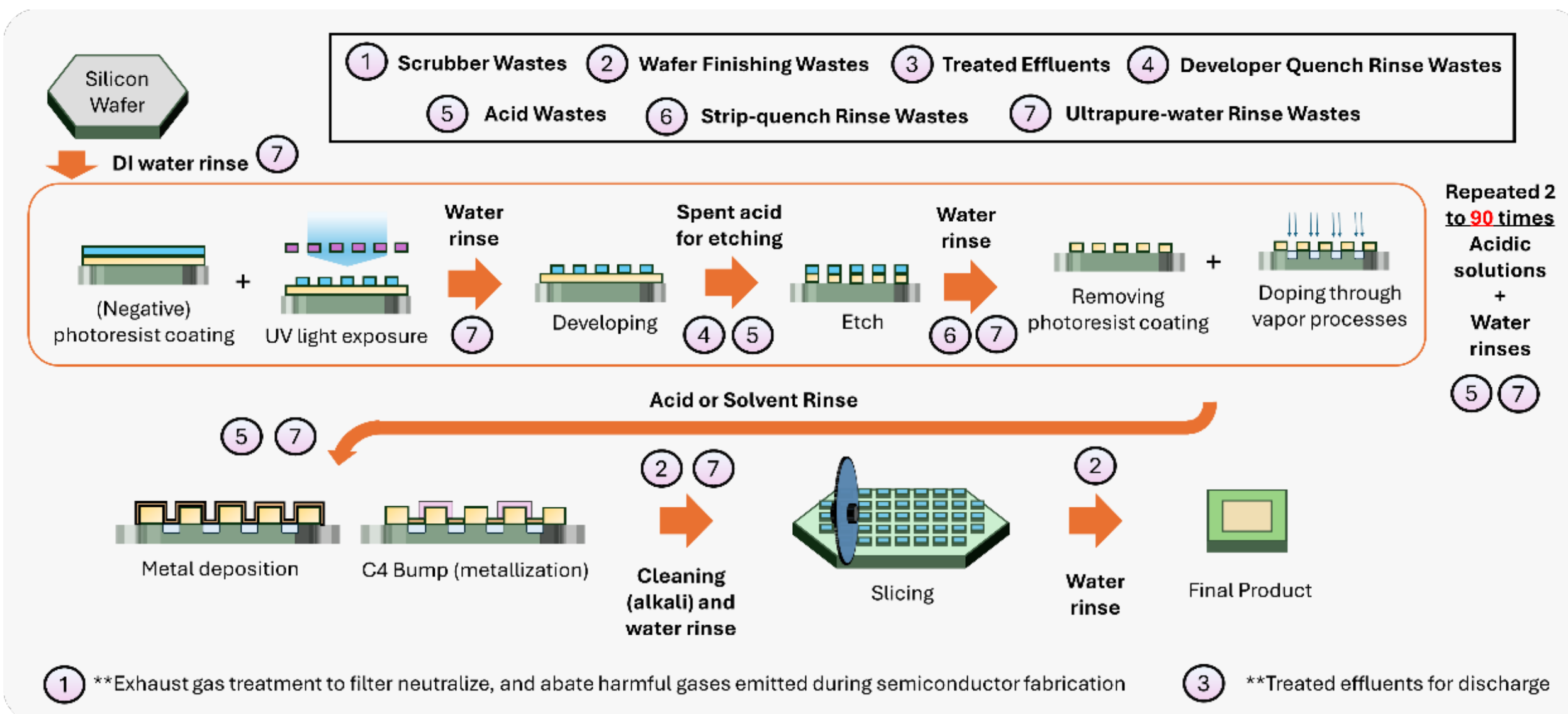

Figure S19. Flow chart of semiconductor manufacturing steps.

Table S27 and **Figure S20** show the characteristics of semiconductor wastewater. An average chip manufacturing plant consumes 18.6–37.8 thousand m^3 of water daily.[48,168] The pH ranges from 1.41 to 9.43. The TDS ranges from 0.68 to 837 mg/L, and the TOC ranges from 3.75 to 322 mg/L. The major background species are K, Ca, Mg, F, Si, and TIN, with concentrations from tens to hundreds of mg/L. A variety of CMs are found, with F, Si, and Mg reaching a few mg/L; other CMs are mostly less than 1 mg/L.

The variation of pH originates from processes involving the use of acidic and alkaline chemicals. Acidic chemicals such as hydrofluoric, hydrochloric, and sulfuric acids are used during etching, while alkaline chemicals such as hydroxides and carbonates are used during cleaning and crystal growth processes. The primary contributors to TDS were Ca, Mg, F, and SO_4, accounting for around 70% of the TDS. Ca and Mg originate from integrated chemical treatments: calcium hydroxide ($Ca(OH)_2$) is used as a coagulant to remove hydrofluoric acid by forming CaF, while

Mg-based additives are used for ammonium removal through struvite precipitation.[169,170] Common cleaning agents include hydrochloric and sulfuric acids contribute to the presence of SO_4 and Cl, although Cl was not reported from collected date. Additionally, although not reported in our data sources, it is reasonable to anticipate high NH_3/NH_4^+ in wastewater due to the use of NH_4OH during device fabrication.[169,171] The TOC comes from the metalorganic compounds used during chemical vapor deposition (CVD).[172] The large number of CMs originates from the use of diverse chemicals during manufacturing. For example, the high F concentration can be attributed to the use of hydrofluoric acid and ammonium bifluoride as cleaning and etching agents;[173,174] metallization of integrated circuits introduces Al, Cu, and Ti; conventional generation of printed circuit boards often use metals such as Cu, Ni, and Sn[169].

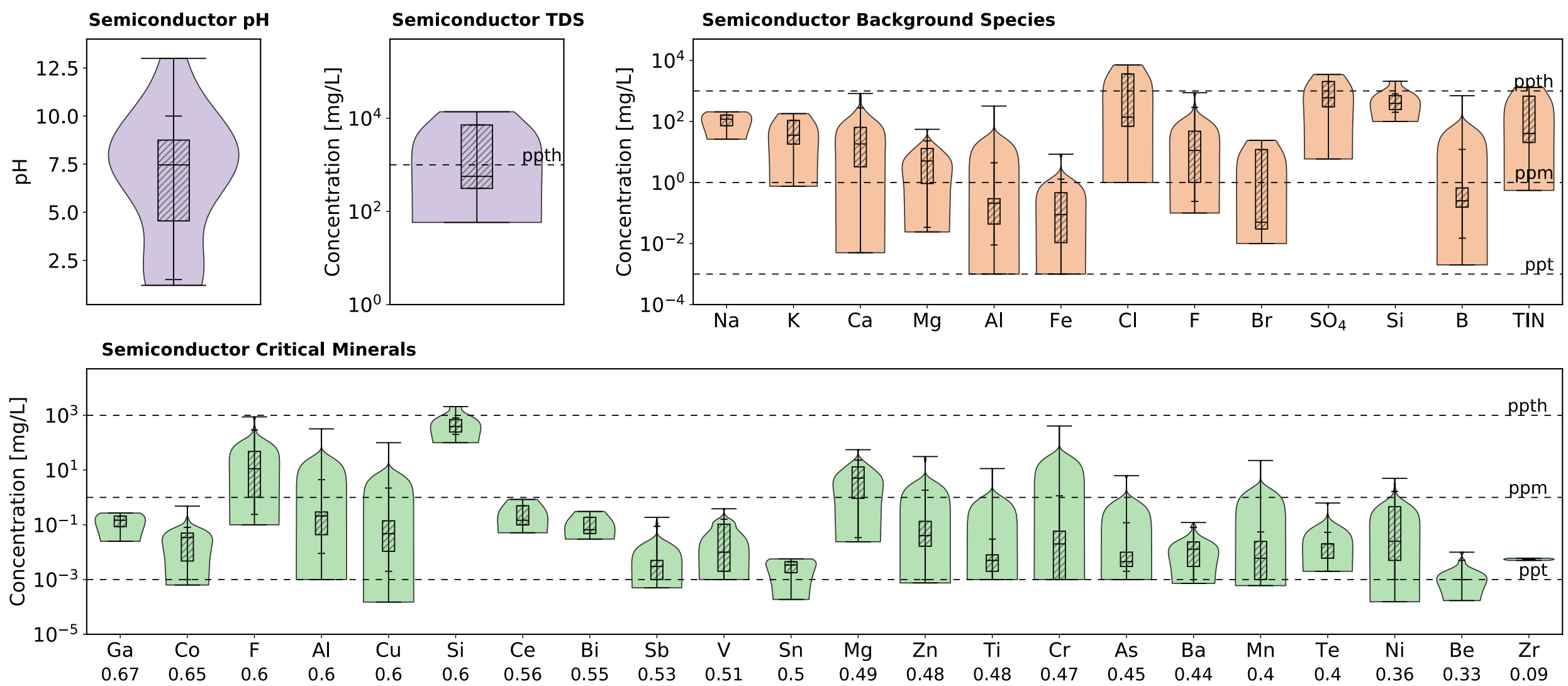

Figure S20. Semiconductor manufacturing wastewater pH, TDS, TOC, background species, and critical minerals concentration distribution. Note that sulfate is missing from the background species because only one data point was found in references.

Table S27. Semiconductor manufacturing wastewater characteristics. Values in parentheses represent the median concentrations. The presented data range is from the 5th to the 95th percentile**.**

Semiconductor Manufacturing			
**Flow [Mm^3/day/plant]**	**pH**	**TDS [mg/L]**	**TOC [mg/L]**
0.02–0.04 (0.03)	1.41–11 (7.46)	109–12,477 (566)	247–289 (268)

Wastewater Constituents					
**Background Species**	**Concentration [mg/L]**	**Median Mineral Proportion in TDS [%]**	**Critical Minerals**	**Concentration [mg/L]**	**Criticality Rating**
SO_4	65–3,183 (599)	45	Ga	0.04–0.26 (0.15)	0.67
Si	125–1,758 (390)	30	Co	1.00×10^{-3}–0.12 (0.03)	0.65
Cl	15–6,395 (138)	10	Cu	1.90×10^{-3}–2.35 (0.05)	0.6
Na	35–198 (119)	9.00	Al	3.40×10^{-3}–5.38 (0.21)	0.6
K	4.24–166 (36)	2.69	F	0.22–302 (11)	0.6
Ca	5.00×10^{-3}–420 (18)	1.39	Pt	3.00×10^{-3}–0.05 (0.01)	0.6
F	0.22–302 (11)	0.87	Ce	0.06–0.78 (0.15)	0.56
Mg	0.03–24 (5.08)	0.38	Bi	0.03–0.28 (0.07)	0.55
TIN	0.14–31 (4.37)	0.33	Sb	8.80×10^{-4}–0.09 (3.00×10^{-3})	0.53
B	9.60×10^{-3}–56 (0.25)	0.02	V	1.00×10^{-3}–0.17 (0.01)	0.51
Al	3.40×10^{-3}–5.38 (0.21)	0.02	Sn	2.28×10^{-3}–0.26 (0.03)	0.5
Fe	1.00×10^{-3}–1.82 (0.09)	6.69×10^{-3}	Mg	0.03–24 (5.08)	0.49
Br	0.01–22 (0.05)	3.78×10^{-3}	Zn	1.00×10^{-3}–8.90 (0.04)	0.48
			Ti	1.00×10^{-3}–0.03 (5.00×10^{-3})	0.48
			Cr	1.00×10^{-3}–7.40 (0.02)	0.47
			As	1.00×10^{-3}–0.12 (4.50×10^{-3})	0.45
			Ba	1.00×10^{-3}–0.09 (0.01)	0.44
			Te	2.00×10^{-3}–0.13 (0.02)	0.4
			Mn	1.00×10^{-3}–0.09 (6.00×10^{-3})	0.4
			Ni	1.00×10^{-3}–1.76 (0.03)	0.36
			Be	8.74×10^{-4}–7.25×10^{-3} (1.00×10^{-3})	0.33
			Zr	5.05×10^{-3}–5.95×10^{-3} (5.50×10^{-3})	0.09

We also observed differences in CM proportions across fabrication steps. As shown in **Figure S21**, CM in the semiconductor fabrication wastewater is dominated by Al, Fe, Cu, and Zn. Al (17.36%) and Cu (5.66%) primarily originate from metallization and interconnect fabrication in integrated circuits (ICs)[169,175–177], Fe (17.33) present as materials associated with yttrium-iron-garnet substrate as well as impurities removed during wafer processing[178–180]. Zn (8.30) is associated with sulfide-based coating used in photocatalytic applications.[179,181] During wafer finishing, wastewater arises from grinding, lapping, and CMP processes and contain nanoparticles (Al_2O_3, SiO_2, and CeO_2),[176,182], oxidizing agents ($CuSO_4$, $Fe(NO_3)_3$),[176] and trace metals (Pt, Sb, and Zn).[179] Acid waste Cr is abundant in due to the use of chromium oxide (CrO_3) for etching[179,183], and Ni is commonly used in metallization and metal surface finishing[179]. Beyond metallization, process specific treatment contributes additional chemical and metal discharges in rinse wastewater. For instance, local wet-scrubber systems treat volatile metal particulates and organic-compound emissions from chemical vapor deposition (CVD) and etching vapors, converting them into a liquid waste stream enriched in F, Cl, and organic compounds.[184,185] Acid rinses used in post-etch cleaning increased the solubility of metal hydroxides such as $Al(OH)_3$ and $Fe(OH)_2$.[177] Quench rinses remove etchants such as hydrochloric acid along with etched metallic impurities including Fe, Cu,[180,186] Al, Zn, and Ni.[186] Collectively, these operations produce wastewater containing a wide array of chemicals including heavy metals, solvents, acids, and bases.

While inorganic solids from sludge wastes such as SiO_2, CaF_2, and CeO_2 are typically managed separately from aqueous wastes through dedicated physical (flotation, magnetic separation) and chemical (leaching, precipitation, solvent extraction) processes, aqueous waste treatment in the semiconductor industry is well developed.[182,187,188] For example, TSMC's in-house waste management reclaim air and exhaust scrubber wastewater for ultrapure water production, thus enabled the recycling of 679,000 m^3 of backwash water in 2024.[188] In addition, solid sludges including CaF_2 and SiO_2 are outsourced externally for purification and resource reutilization for other industries.[187,188]

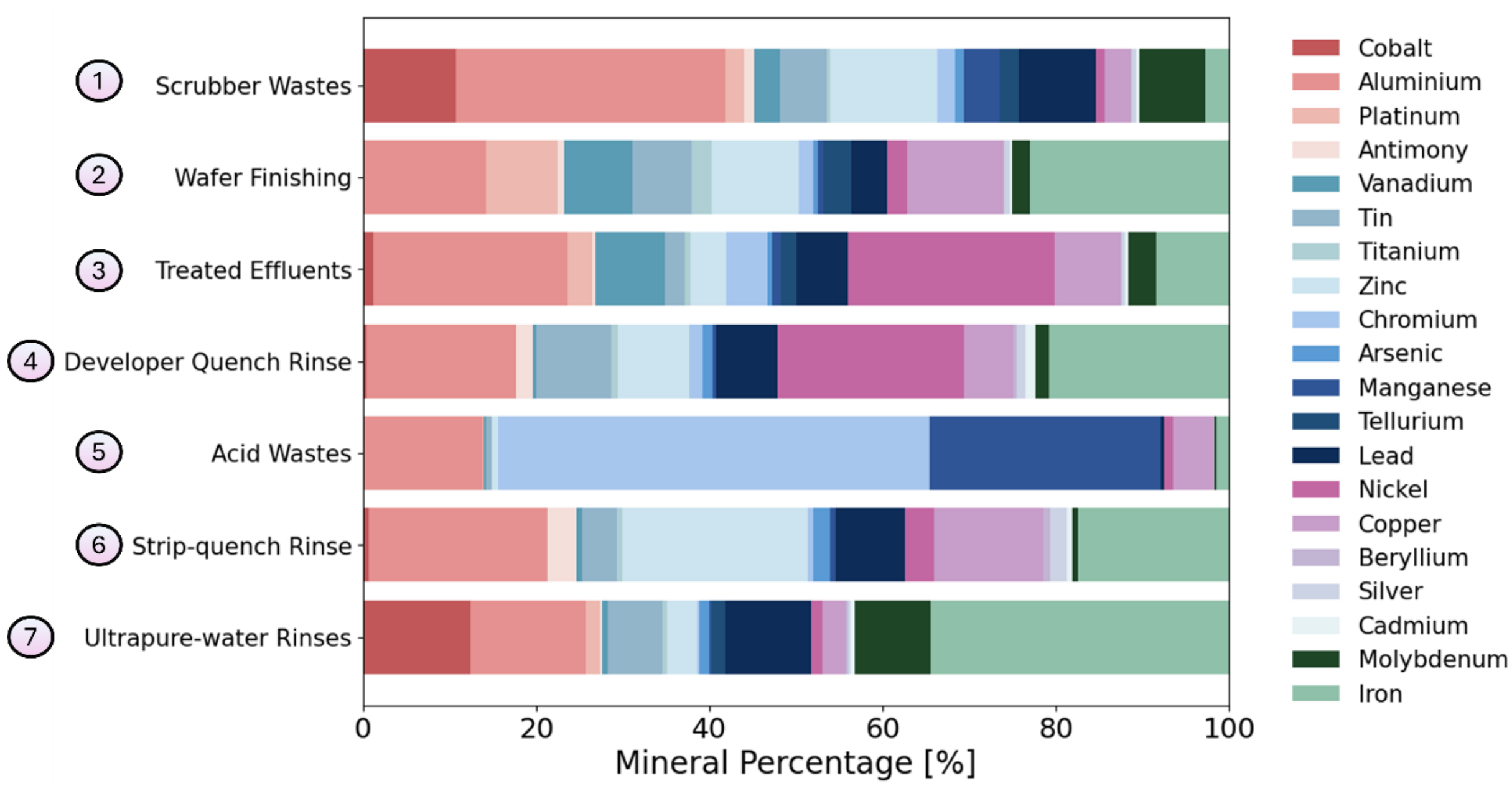

Figure S21. Critical mineral distribution in wastewater from specific semiconductor fabrication steps. Data used in this figure was obtained from EPA report "Electrical And Electronic Components Point Source Category Phase I".[179]

S8.2 Leather tanning wastewater

Leather tanning converts raw hide (also referred to pelts or skin) into finished leather goods through a series of chemical and mechanical treatments. Wastewater generated from tanning industries is derived from three main processes: (1) pre-tanning, (2) tanning, and (3) post tanning. In general, pre-tanning and post-tanning processes account for the largest share of water consumption in leather tanning.[189–191] Within this stage, soaking and (de)liming contribute most of the dissolved solids.[189,190,192] Pre-tanning includes the preparation of raw hides for tanning (e.g., removing hair from hides) by soaking, liming, unhairing, bating, and degreasing. Tanning improves the durability and flexibility of the animal hide through chemical treatments. Post-tanning improves the overall physical properties and appearance of leather. Altogether, these three processes generate wastewater with high levels of sodium chloride, sodium sulfide, colored dyes, toxic metals, and suspended solids.[189,190,192–194] As of 2023, four countries account for nearly half of worldwide cattle hide production: China (17%), United States (13%), Brazil (11%), and India (7%).[195]

Table S28 and **Figure S22** show the typical volume and characteristics of tanning wastewater. The pH ranges from 3.47 to 12. The TDS ranges from 821 to 28,267 mg/L. The TOC ranges from 62 to 3,101 mg/L. Major background species include Na, K, Cl, SO_4, and TIN, with concentrations reaching a few to tens of thousands of mg/L. The concentration of nitrogen compounds is high (2.3–31,000 mg/L) and is dominated by dissolved ammonia. Most CMs have concentrations around tens of mg/L; for Mg, Al, and Cr, the concentration can be as high as hundreds of mg/L.

The variation of pH can be attributed to the chemical used during different tanning activities. For instance, sodium sulfide (Na_2S) and lime (CaO) are used in pre-tanning resulting in a pH as high as 12. On the other hand, strong acids like sulfuric acid are used during and post-tanning, resulting in a low pH around 3–4.[189,190,196] The high TOC is from the protein and fat of the hide and organic dyes used for color development.[189,197,198] Background species, including Ca, Mg, and Na, are used to pretreat the effluent.[199] The high Cr is attributed to the prevalence of chrome tanning method that uses chromium sulfate, dichromate, and sodium dichromate.[189] Among the four top hide-producing countries, chrome processing is predominantly used in China and India;[194] in particular, approximately 80% of tanneries in India employed chrome processes in 2022.[200] Other CMs including Cu, As, and Zn are often adopted in dye fixation to impart colors to textiles, resulting in high concentration of CMs in the wastewater.[190,192,196,201,202]

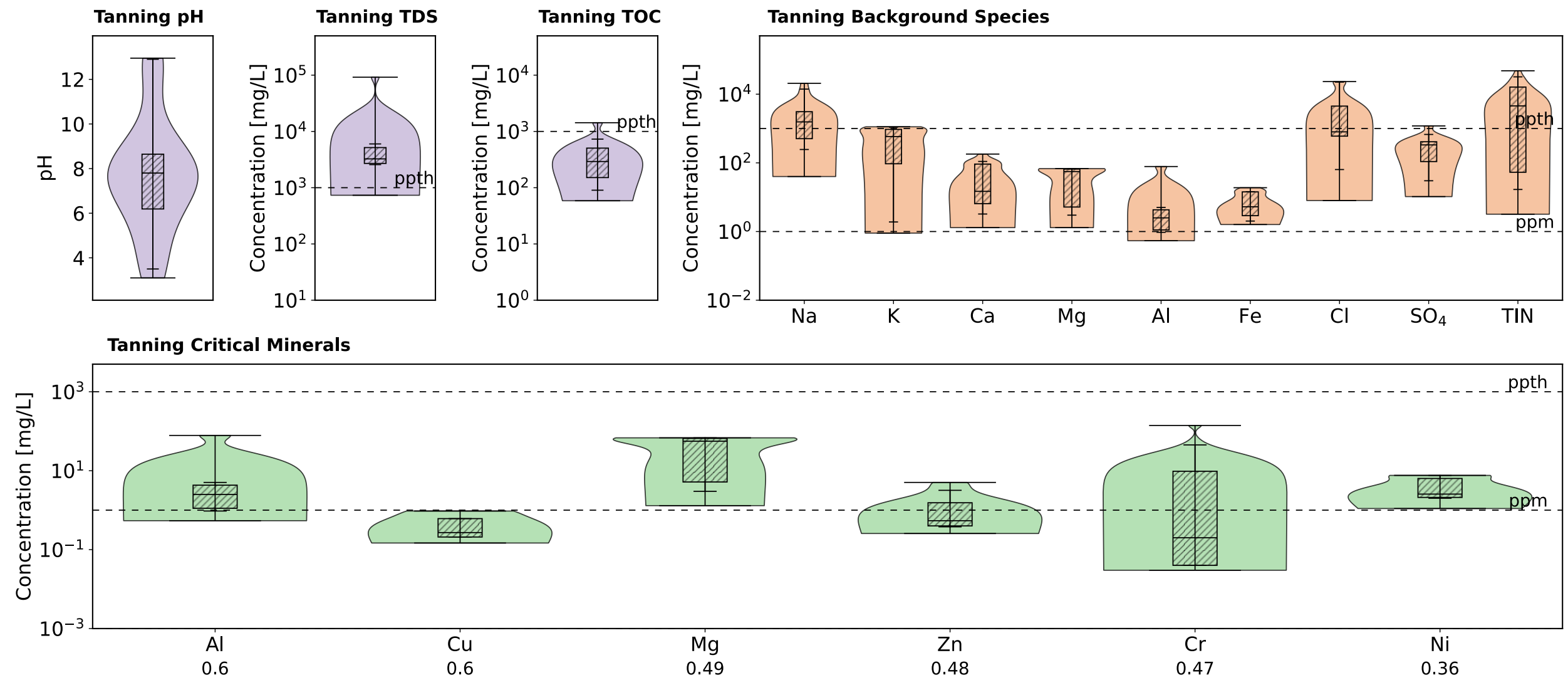

Figure S22. Tanning wastewater pH, TDS, TOC, background species, and critical minerals concentration distribution.

Table S28. Leather tanning wastewater characteristics. Values in parentheses represent the median concentrations. The presented data range is from the 5th to the 95th percentile.

Leather Tanning					
**Flow [m^3/ton-raw hide]**		**pH**		**TDS [mg/L]**	**TOC [mg/L]**
15–50 (32)		3.45–13 (7.80)		1,836–40,400 (3,252)	81–940 (292)
**Wastewater Constituents**					
**Background Species**	**Concentration [mg/L]**	**Median Mineral Proportion in TDS [%]**	**Critical Minerals**	**Concentration [mg/L]**	**Criticality Rating**
Na	184–16,057 (1,560)	42	Cu	0.16–0.88 (0.27)	0.6
Cl	64–22,500 (800)	22	Al	0.66–56 (2.50)	0.6
K	1.35–1,110 (584)	16	Mg	1.90–68 (56)	0.49
SO_4	21–906 (333)	9.03	Zn	0.30–4.30 (0.54)	0.48
Mg	1.90–68 (56)	1.51	Cr	0.04–64 (0.20)	0.47
TIN	4.05–1,468 (29)	0.79	Ni	1.33–7.59 (2.55)	0.36
Ca	2.66–131 (15)	0.40			
Fe	1.85–16 (5.26)	0.14			
Al	0.66–56 (2.50)	0.07			

S8.3 Metal plating and finishing wastewater

Metal plating and finishing contributes to a variety of products in the automotive, electronics, marine, and aerospace sectors by ensuring electrical conductivity, enhancing appearance, and/or improving corrosion resistance. For example, Zn and Cr electroplating protect steel parts from corrosion, while Ni coatings add chemical resistance and increase surface hardness. The diverse range of coating compositions and application methods results in the presence of a wide variety of metals including Fe, Cu, Cr, Ni, and Zn in wastewater associated with these operations.[203]

Table S29 and **Figure S23** show the typical characteristics of metal plating and finishing wastewater. The pH ranges from 1.18 to 7.85. The TDS ranges from 18-1,266 mg/L. The TOC ranges from 42 to 279 mg/L. Major background species are Na, Al, Fe, and SO_4. Almost all reported CMs have concentrations from tens to hundreds of mg/L.

The low pH originates from acid cleaning agents, most commonly sulfuric acid, along with hydrochloric and phosphoric acids.[204] These acid also contribute to the background species including SO_4, Cl, and PO_4. Note that Cl and PO_4 are not shown in the **Table S29** and **Figure S23** because Cl is not reported in our references, and PO_4 is not included in our list of background species. Chemicals used during surface finishing operations contribute both to the high CM concentration and the background species. For example, Cr plating use chromic acid or potassium dichromate ($KCrO_4$);[205] Zn and Al plating use acidic (e.g., $ZnSO_4$, $AlCl_3/Al_2(SO_4)_3$) or alkaline (e.g., $ZnCl_2$, NH_4Cl) electrolytes;[203,204,206] Cu plating uses cyanide-based alkaline or acid sulfate baths; Ni plating uses chemicals including Watts-type ($NiSO_4$-$NiCl_2$-$B(OH)_3$), sulfamate-based ($Ni(NH_2SO_3)_2$-$NiCl_2$-$B(OH)_3$), and other reducing agents (e.g., sodium hypophosphite).[204,206] Treatment steps prior to wastewater discharge, such as using calcium-based reagents (e.g., CaO, $Ca(OH)_2$, $CaCl_2$, and $CaSO_4$) for metal precipitation and neutralization, also introduce background species.[204]

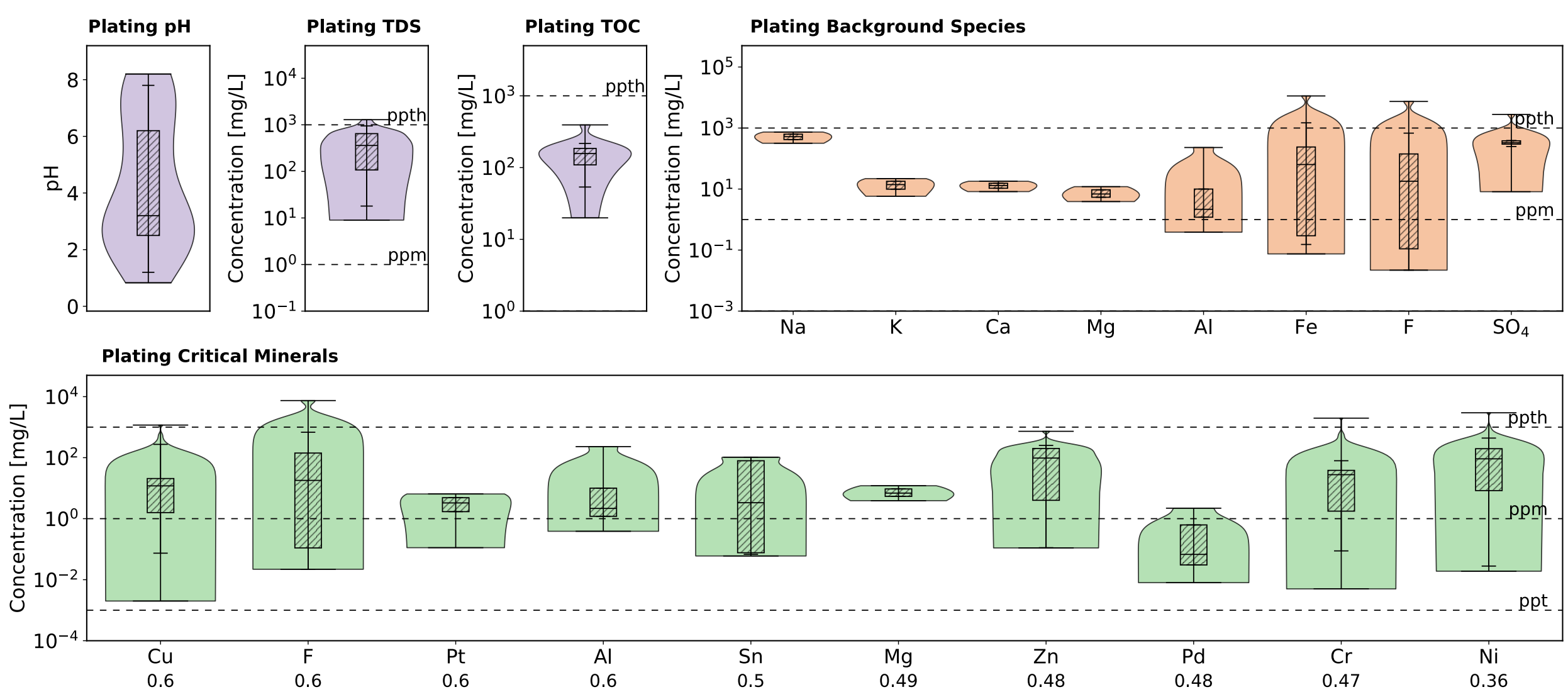

Figure S23. Plating wastewater pH, TDS, TOC, background species, and critical minerals concentration distribution.

Table S29. Metal plating and finishing wastewater characteristics. Values in parentheses represent the median concentrations. The presented data range is from the 5th to the 95th percentile**.**

Metal Plating and Finishing					
Flow [Mm^3/year]		pH		TDS [mg/L]	TOC [mg/L]
4.39–33 (19)		1.16–7.88 (3.20)		17–969 (360)	44–270 (157)
**Wastewater Constituents**					
**Background Species**	**Concentration [mg/L]**	**Median Mineral Proportion in TDS [%]**	**Critical Minerals**	**Concentration [mg/L]**	**Criticality Rating**
Na	337–717 (521)	38	Cu	0.06–378 (12)	0.6
SO_4	140–1,473 (331)	24	F	0.02–4,732 (18)	0.6
F	0.02–4,732 (18)	1.30	Al	0.55–186 (2.17)	0.6
K	6.63–21 (14)	1.01	Pt	0.43–6.14 (3.28)	0.6
Ca	8.73–18 (13)	0.94	Sn	0.06–103 (3.34)	0.5
Mg	4.19–11 (6.89)	0.50	Mg	4.19–11 (6.89)	0.49
Al	0.55–186 (2.17)	0.16	Zn	0.11–299 (97)	0.48
Fe	0.04–6,374 (0.41)	0.03	Pd	0.01–1.89 (0.07)	0.48
			Cr	0.09–504 (28)	0.47
			Ni	0.03–557 (92)	0.36

S8.4 Battery manufacturing wastewater

The battery manufacturing industry has evolved with advances in electrochemical energy storage, spanning a wide range of electrochemical technologies from legacy Pb-acid to state-of-the-art Li-ion batteries. In 2024, the Pb-acid battery industry and production in the U.S. accounts for 86% of national Pb consumption, largely driven by the demand for automotive and industrial standby power.[12] Beyond Pb acid batteries, manufacturing of several legacy battery chemistries also generates wastewater containing CMs including Zn, Cd, and Co.[8] As categorized in the U.S. EPA, these legacy batteries are defined by their electrode chemistries which determines the metal constituents released during electrode fabrication, electrolyte preparation, and equipment cleaning. Representative examples include cadmium-based batteries such as Ag-cadmium, Ni-cadmium, and Hg-cadmium batteries, while Zn-based batteries are comprised of AgO, Zn and Ni-Zn electrodes. Alkaline manganese cells incorporate $Ni(OH)_2$, HgO, MnO_2, and Ag_2O, depending on the cell architecture and formulation.

Figure S24 and **Table S30** describe the characteristics of battery manufacturing wastewater. The pH ranges from 2.03 to 12. The TDS ranges from 2.05–480 mg/L. The background

species reported were Al, Fe and SO_4, all around 1 mg/L level. Major CMs, all reported at or above 1 mg/L concentration, were Co, Al, Cu, Sb, Zn, Cr, and Mn. As and Li were reported at lower levels.

The wide pH range reflects the use of alkaline and acid electrolytes, as well as acid used for sulfuric acid treatments to condition Pb electrodes.[8] For example, cadmium-based, zinc-based and alkaline-manganese battery uses alkaline electrolytes (e.g., potassium and lithium hydroxide), contributing to the high pH, whereas lead battery use acid electrolytes (e.g., H_2SO_4 and NH_4SCN).[8–10] Other processes, such as cell washing and cleaning incorporates acetic acids and chromic acids, as well as sulfuric acid used in Pb-acid battery to form $PbSO_4$ layers on Pb electrodes, also contribute to the low pH.[8] The presence of the major background species are originated from the dissolution of electrodes and current collector materials, and the sulfuric acid-containing electrolytes. Moreover, Fe is often used as a coagulant to pre-treat wastewater prior to discharge.[204] CMs such as Sb, As, Cd, Cu, Se, and Sn can leach from battery electrodes during manufacturing and recycling processes, as these materials are commonly incorporated as grid hardening agents and other functional additives.[207]

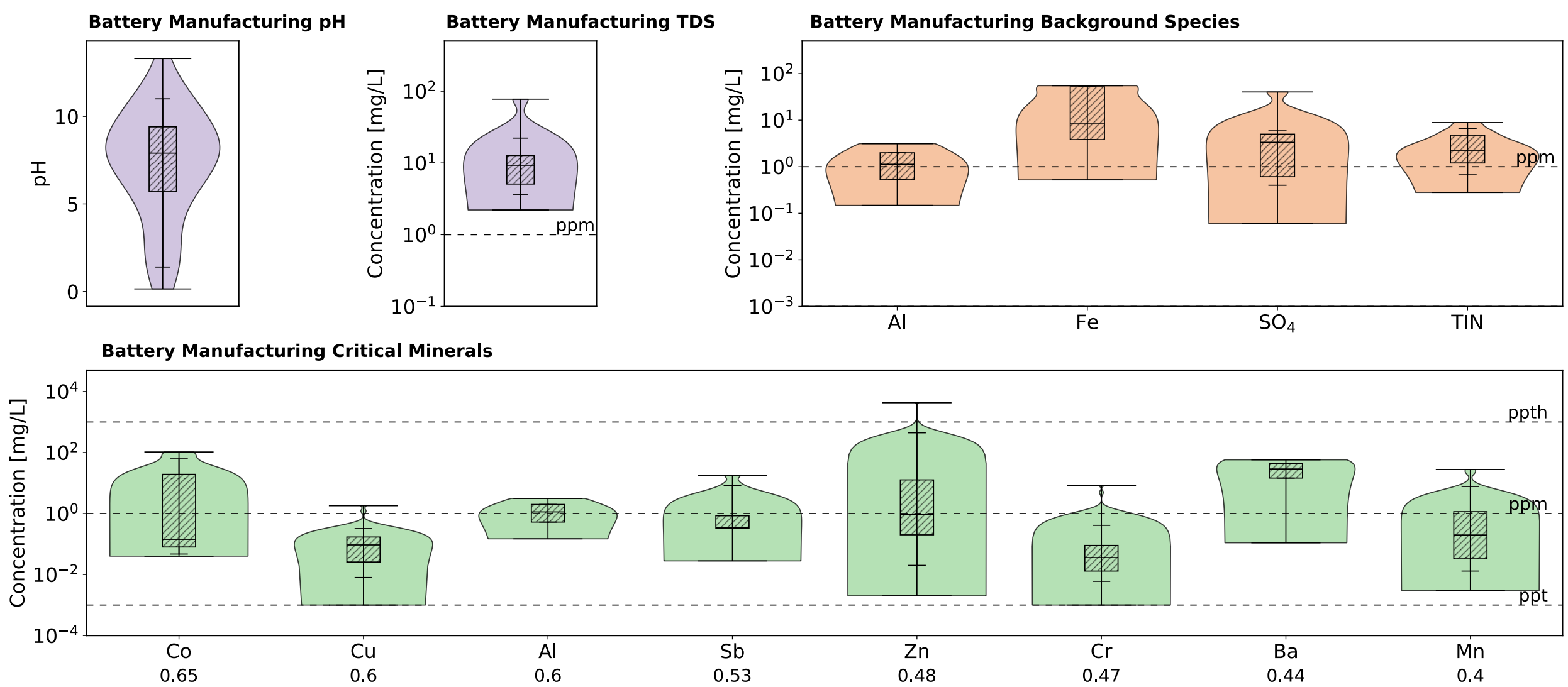

Figure S24. Battery manufacturing wastewater pH, TDS, background species, and critical minerals concentration distribution.

Table S30. Battery manufacturing wastewater characteristics. Values in parentheses represent the median concentrations. The presented data range is from the 5th to the 95th percentile.

Battery Manufacturing					
Flow [Mm^3/year]		pH		TDS [mg/L]	TOC [mg/L]
4.39–139 (30)		1.40–11 (7.90)		2.79–55 (9.28)	1,350 (1,350)
**Wastewater Constituents**					
**Background Species**	**Concentration [mg/L]**	**Median Mineral Proportion in TDS [%]**	**Critical Minerals**	**Concentration [mg/L]**	**Criticality Rating**
Fe	1.18–54 (8.30)	49	Co	0.04–89 (0.14)	0.65
SO_4	0.20–39 (3.50)	21	Cu	6.40×10^{-3}–0.69 (0.10)	0.6
TIN	0.30–8.46 (2.26)	13	Al	0.03–2.83 (0.65)	0.6
Al	0.03–2.83 (0.65)	3.82	Sb	0.19–13 (0.39)	0.53
			Zn	0.01–477 (0.76)	0.48
			Cr	5.70×10^{-3}–1.44 (0.04)	0.47
			As	2.80×10^{-3}–0.29 (0.04)	0.45
			Mn	9.20×10^{-3}–21 (0.20)	0.4
			Ni	2.00×10^{-3}–14 (0.08)	0.36

S8.5 Lithium battery (LIB) recycling wastewater

Since the 1990s, the growing use of rechargeable lithium-ion battery (LIB) has spurred increasing interest in spent batteries recycling. It typically involves first sorting and mechanical crushing, followed by hydrometallurgical,[208,209] pyrometallurgical,[208] direct recycling (i.e., direct re-use of active components), floatation,[210] or integrated pyrometallurgy with subsequent hydrometallurgical treatment of the resulting slag. Wastewater generated during these operations contains dissolved metals, residual electrolyte salts, and organic compounds.[211] As of 2025, the global LIB recycling capacity is approximately 1.6 Mt per ton of raw battery waste every year. This capacity is dominated China, which contribute more than 1.1 Mt/year, followed by Europe, U.S. and India with the capacities of 200,000 tons/year, 144,000 tons/year and 89,900 tons/year respectively.[212] Among the recycled LIBs, electric vehicle related waste was estimated to be 500,000 tons in 2019 and is projected to increase to about 8 Mt by 2040.[213]

Figure S25 and **Table S31** describe the typical volume and characteristics of LIB recycling wastewater.[214–216] The pH vary by the recycling methods; pH of direct recycling wastewater ranges from 7.1 to 7.4, and hydrometallurgical processes have slightly more acidic wastewater around 6.6. TOC was mainly reported from direct recycling effluents that ranges from 300 to 502 mg/L[215]. The TDS ranges from 1,011 to 133,233 mg/L and depends on the recycling processes; wastewater from hydrometallurgy have TDS above 10,000 mg/L, whereas from direct recycling the TDS is only a few hundred to a thousand mg/L. This difference arises because hydrometallurgy leverages chemical leaching for recovery, while direct recycling preserves material purity with simpler processes.[217,218]. Major background species are Na and SO_4 with concentration around several thousand mg/L. Major CMs are Cu, Mg, Li, Ni, with typically above 1 mg/L; other CMs are before 1 mg/L level

The relatively higher pH in direct recycling wastewater is largely attributed to the use of NaOH during washing and hydrolysis to precipitate metals from electrolyte.[219] The low pH in hydrometallurgy wastewater is due to the use of strong acids such as H_2SO_4 or HNO_3 to leach spent electrodes.[214,219–221] Organic electrolyte components such as ethylene carbonate (EC), dimethyl carbonate (DC), and diethyl carbonate (DMC) contribute to the TOC. The wastewater composition depends on the cell components, including active material coated on the metallic current collector (typically composed of either an Al or Cu foil), organic separator, and steel or plastic casings.[209,215,222] Other major contributors include spent electrodes and electrolyte salts, such as Li_2CO_3, Li_2SO_4, NaF, LiF, and $LiPF_6$.[223,224] Elements such as Mn, Co, Li, and Cu originate from cathodic materials, commonly nickel-manganese-cobalt oxide cathode.[225]

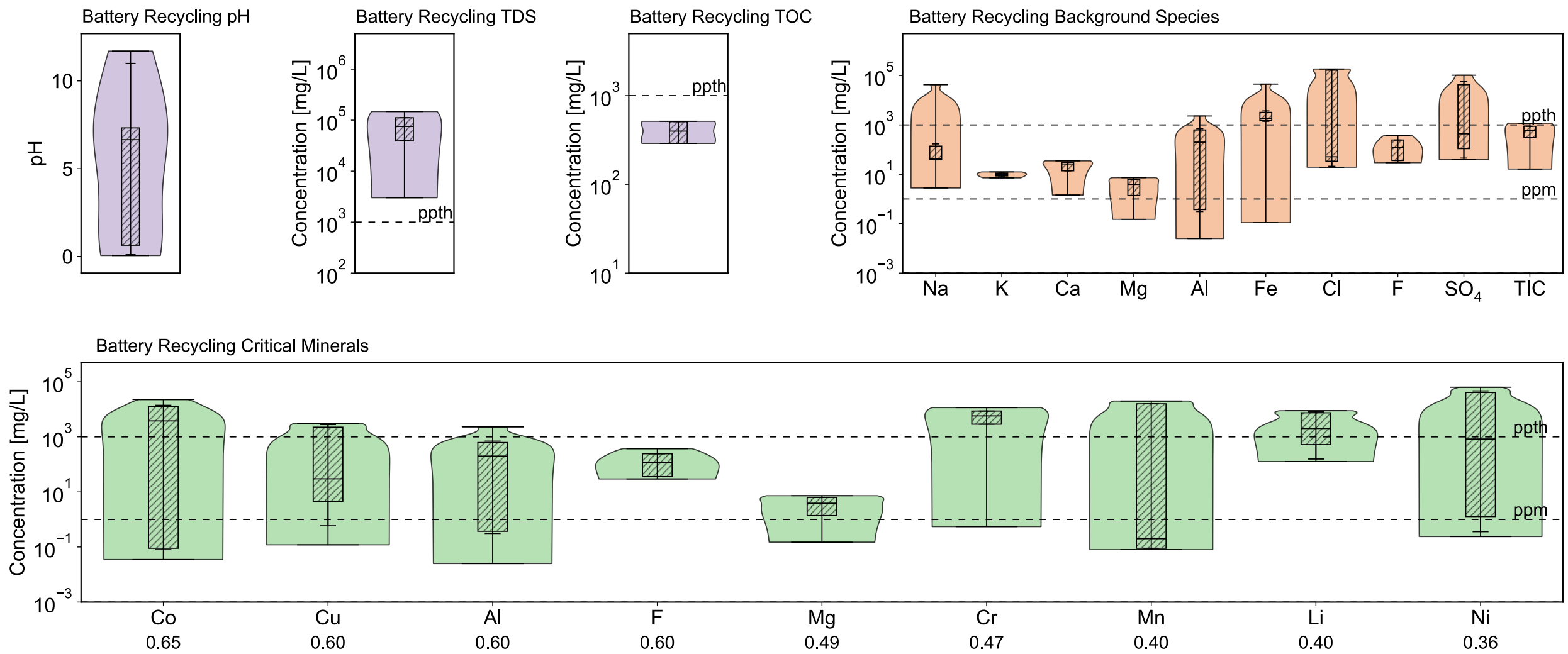

Figure S25. Lithium-ion battery recycling wastewater pH, TDS, background species, and critical minerals concentration distribution.

Table S31. Lithium-ion battery recycling wastewater characteristics. Values in parentheses represent the median concentrations. The presented data range is from the 5th to the 95th percentile.

Lithium Battery Recycling					
Flow [m^3/ton-recycled]		pH		TDS [mg/L]	TOC [mg/L]
675		0.07–11 (6.65)		10,242–140,457 (75,350)	289–513 (398)
**Wastewater Constituents**					
**Background Species**	**Concentration [mg/L]**	**Median Mineral Proportion in TDS [%]**	**Critical Minerals**	**Concentration [mg/L]**	**Criticality Rating**
SO_4	40–87,614 (803)	32	Co	0.04–0.12 (0.08)	0.65
F	140–451 (296)	12	Cu	0.17–27 (0.59)	0.6
Na	8.46–36,108 (105)	4.20	Al	0.05–0.54 (0.31)	0.6
Cl	47–51 (49)	1.95	F	140–451 (296)	0.6
Ca	3.92–34 (26)	1.05	Mg	0.73–7.17 (6.00)	0.49
Mg	0.73–7.17 (6.00)	0.24	Li	132–2,073 (1,038)	0.4
Al	0.05–0.54 (0.31)	0.01	Mn	0.08–0.19 (0.09)	0.4
			Ni	0.26–444 (0.48)	0.36

S8.6 Data sources

Semiconductor manufacturing wastewater characteristics were primarily obtained from technical reports published by the U.S. EPA in 1983[179] and 2022[226].

Metal plating/finishing wastewater characteristics were obtained from wastewater discharge reports from the U.S., Turkey,[227,228] China,[205] India,[229] and Hong Kong[230] spanning from 1979 to 2024. These reports describe wastewater characteristics from both large-scale plating/finishing facilities and product-specific manufacturing processes.

Tanning wastewater characteristics were obtained from studies describing facilities in the U.S., Ethiopia,[231] Ghana,[189] India,[192,232,233] Bangladesh,[234,235] Pakistan,[236–238] and Iran[239]. The dataset includes both chrome and vegetable leather tanning processes, along with textile finishing operations which together represent the major sources of tanning wastewater.

Battery manufacturing wastewater characteristics were obtained from technical reports published by the U.S. EPA in 1976 [8] and a 1993 peer-reviewed article[240]. These reports focused on the manufacturing of then-dominant battery technologies such as Pb-acid, cadmium, Leclanché, and Zn-carbon cells rather than contemporary LIB production. Wastewater data on current LIB production and recycling remains scarce due to the still-emerging state of the industry, proprietary industrial processes and the limited public reporting of discharge compositions.

LIB recycling wastewater data were primarily obtained from technical reports and peer-reviewed articles from the U.S.[210,216], Norway[241], Germany[210,215], Korea[214], China[242], and Italy[221] spanning from 2010 to 2026. The reported volume and characteristics of wastewater is a combination of industrial-scale and pilot-scale studies that are representative of modern-day LIB electrode compositions. Our data sources include: the industrial hydrometallurgical wastewater data from Norway, Korea, and Italy[214,221,241]; direct recycling wastewater data from the U.S., China, and Germany[215,216,242]; and black mass flotation wastewater from Germany[210].

We report the wastewater characteristics measured directly at the point of discharge, including all background species and CMs listed in the original data. Reported wastewater characteristics for semiconductor and battery manufacturing reflect the integrated in-plant recovery and treatment methods before final discharge.

S8.7 Data limitations

Representativeness of industries: Manufacturing activities are highly process and product driven. Hence, the wastewater characteristics are expected to vary to an even greater extent than those of other industries depending on the specific operations, chemistry, and location of a facility. However, data are highly limited for most manufacturing sectors in our data search. As a result, in reality, specific facilities may have wastewater characteristics that fall outside the ranges we report here.

We also have not further segment the data based on the specific manufacturing steps within each activity. For example, for semiconductor manufacturing activity, data are from sources that generalize across multiple fabrication steps such as etching and rising, rather than reflecting process-specific discharges. In practice, wastewater chemistry depends strongly on etching chemistry and process integration. Similarly, our battery manufacturing wastewater dataset includes sourced from Pb-acid, cadmium, Leclanché, and Zn-carbon batteries; while each generated wastewater with different characteristics, our dataset did not report them separately.

Section S9. Fossil Fuel Power Generation

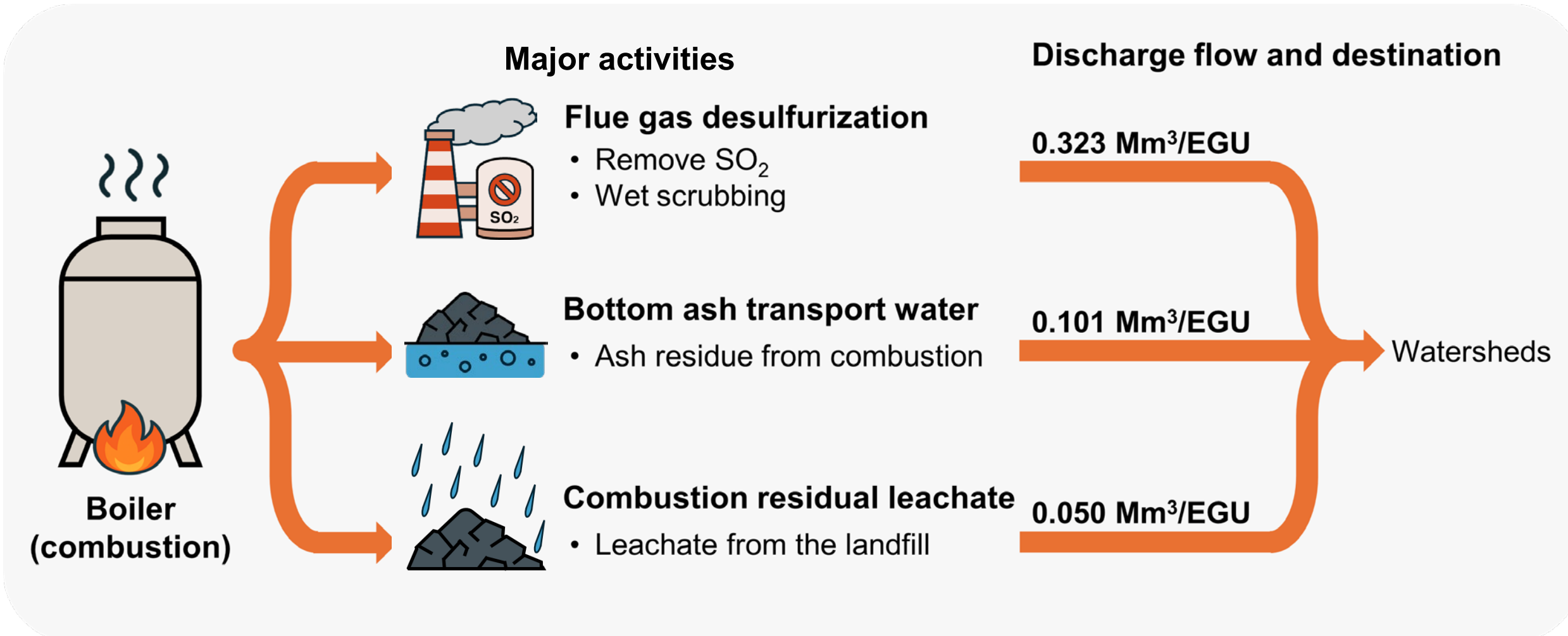

Figure S26. Major activities and flow rates associated with fossil fuel power generation.

S9.1 Industry description

Fossil fuels, including coal, oil, and natural gas, accounted for 63.1% of the world's total electricity supply in 2019 (an estimated 26,936 terawatt-hours; TWh) due to their high energy density and reliability.[243–245] Fossil fuel power plants convert the chemical energy of fossil fuels into thermal energy by combustion, which raises the temperature of a working fluid (often liquid water) in a boiler, creating high pressure steam. This steam subsequently rotates the steam turbine connected to an electric generator, consequently producing electricity.[246] Coal was the leading fuel source, generating 36.7% of global electricity, followed by natural gas (23.6%).[245] In the U.S., fossil fuels accounted for 60% of the 4,178 TWh of electricity generated in 2023. Natural gas was the leading fuel source (43.1%) followed by coal (16.2%).[247]

S9.2 Overview of fossil fuel wastewater streams

Management of the waste generated by combustion is the primary source of wastewater generation by this industry. Major waste handling processes, include flue gas desulfurization (FGD), ash handling and landfill leachate (**Figure S26**). EPA's 2024 effluent limit guidelines recognized four distinct types of wastewater: FGD wastewater, bottom ash transport water, combustion residual leachate (CRL) wastewater, and legacy water.[52,248] We adopted this categorization in our analysis; however, we excluded legacy wastewater since it only represents residual mixtures of wastewater streams produced before tightened 2015 or 2020 rules were applied. Note that cooling water is not included in our study because it is primarily sourced from freshwater, does not come into contact with process materials, and is typically discharged directly to watershed without additional treatments (known as "once through cooling").[249] The annual wastewater volume from each stream is normalized by energy generating unit (EGU), where one EGU corresponds to one major piece of generating equipment (e.g., a boiler or turbine).[52]

S9.3 Flue gas desulfurization (FGD) wastewater

Sulfur dioxide (SO_2) gas is a significant by-product of fossil fuel combustion.[250] Fossil fuel power plants leverage FGD systems to remove SO_2 to meet emission limits.[251,252] FGD systems employ adsorbents or scrubbers to capture SO_2, and are categorized into wet, semi-dry, and dry systems

based on the physical state of the sorbents. Wet systems exhibit higher removal efficiency (≥ 90%) compared to other processes (50-90%) and also generate the largest volume of wastewater.[253] Recent efforts to achieve zero liquid discharge are shifting the industry towards dry or semi-dry systems.[254,255] In the U.S, 200 out of 336 power plants employ dry or semi-dry systems, while the remining 136 power plants operate wet FGD as of 2022.[254] Despite their smaller numbers, however, wet FGD systems remain the dominant source of wastewater generation by fossil fuel power plants.

Table S32 and **Figure S27** summarize the typical volume and characteristics of FGD wastewater.[52,256,257] Each EGU generated approximately 0.32 Mm^3 of FGD wastewater. The pH ranges from 5.97 to 7.23. The TDS ranges from 8,100 to 35,000 mg/L. The major background species were Na, Ca, Mg, Cl, and SO_4, with concentrations exceeding thousands of mg/L. The major CMs were Al, F, Si, Mg, Ti, and Mn, with concentrations tens to thousands of mg/L; other reported CMs were typically below 1 mg/L.

The variation of water quality in FGD wastewater is determined by multiple factors such as the type of coal used, choice of sorbents and additives, operational materials, and upstream air pollution systems.[258] For instance, wet scrubbers utilize materials such as limestone ($CaCO_3$) to make slurry with fresh water or seawater for SO_2 capture. This process generates wastewater that contains flue gas byproducts, scrubber residues, and other associated constituents,[259,260] resulting in high concentration of Ca, Mg, Cl, and SO_4.

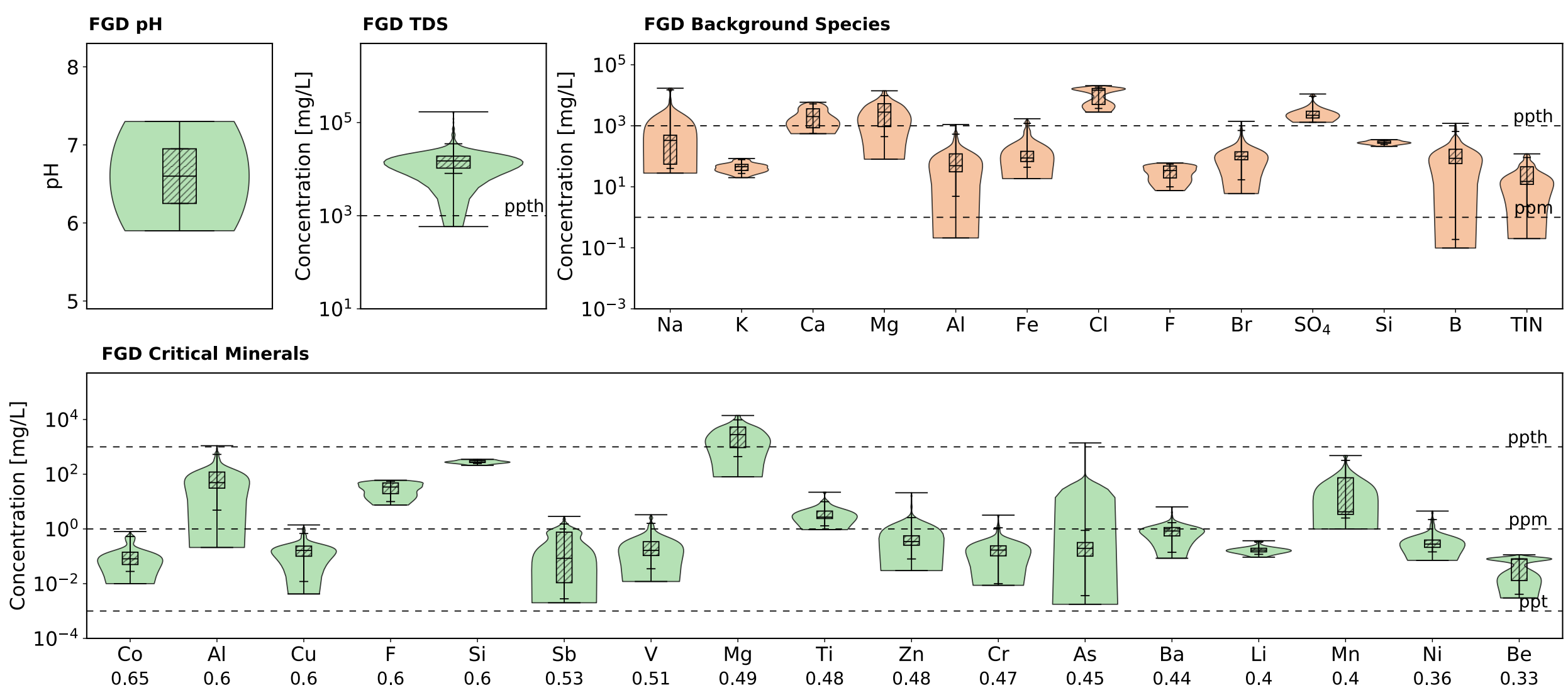

Figure S27. Flue gas desulfurization wastewater pH, TDS, background species, and critical minerals concentration distribution.

Table S32. FGD wastewater characteristics. Values in parentheses represent the median concentrations. The presented data range is from the 5th to the 95th percentile**.**

Flue Gas Desulfurization					
Flow [Mm^3/year/EGU]		pH		TDS [mg/L]	TOC [mg/L]
0.32		5.97–7.23 (6.60)		8,100–35,000 (15,000)	N/A
**Wastewater Constituents**					
**Background Species**	**Concentration [mg/L]**	**Median Mineral Proportion in TDS [%]**	**Critical Minerals**	**Concentration [mg/L]**	**Criticality Rating**
Cl	3,700–18,500 (14,600)	71	Co	0.02–0.53 (0.08)	0.65
Mg	440–9,700 (2,800)	14	Al	0–491 (40)	0.6
SO_4	1,300–9,300 (2,200)	11	Cu	0–0.68 (0.15)	0.6
Na	40–15,000 (330)	1.60	F	9.55–56 (34)	0.6
Si	231–345 (280)	1.35	Sb	0–1.47 (0.04)	0.53
Br	17–700 (100)	0.48	V	0–1.53 (0.14)	0.51
B	0.19–672 (86)	0.42	Mg	440–9,700 (2,800)	0.49
Fe	0–1,135 (75)	0.36	Zn	0.07–5.05 (0.35)	0.48
K	27–78 (46)	0.22	Ti	0–7.50 (1.30)	0.48
Al	0–491 (40)	0.19	Cr	0–1.10 (0.16)	0.47
F	9.55–56 (34)	0.16	As	0–0.79 (0.17)	0.45
TIC	18–61 (31)	0.15	Ba	0.14–1.77 (0.84)	0.44
Ca	13–4,600 (27)	0.13	Mn	2.48–334 (4.23)	0.4
TIN	0–92 (15)	0.07	Li	0.09–0.34 (0.17)	0.4
			Ni	0.14–2.20 (0.28)	0.36
			Be	0–0.08 (0.02)	0.33

S9.4 Bottom ash transport water

Bottom ash is produced from the combustion of fossil fuel, mostly from coal and oil, and accumulates at the bottom of the boiler. Wet–sluicing systems are used to transport this ash from the boiler to a waste ash pond, producing bottom ash transport wastewater.

Figure S28 and **Table S33** summarize the typical volume and characteristics of bottom ash transport water.[52,261] Each EGU generates approximately 0.10 Mm^3. The pH is neutral to slightly alkaline, ranging from 5.81 to 8.73. The TDS ranges from 129 to 540 mg/L. The major background species are Ca and SO_4, with concentrations reaching hundreds of mg/L. The major CMs are Al, Si, and Mg, with concentrations from a few to tens of mg/L. Other CM concentrations were on the order of 0.01 mg/L.

The major constituents are suspended solids including ash residues (i.e., fine particulate matter) with trace dissolved metals from combustion.[248] Most of the reported background ions, including Si, Al, Fe, Ca, and Mg, are major constituents of coal ash while trace metals such as Cr and As originate from the parent coal but become concentrated in the low-volume ash.[262]

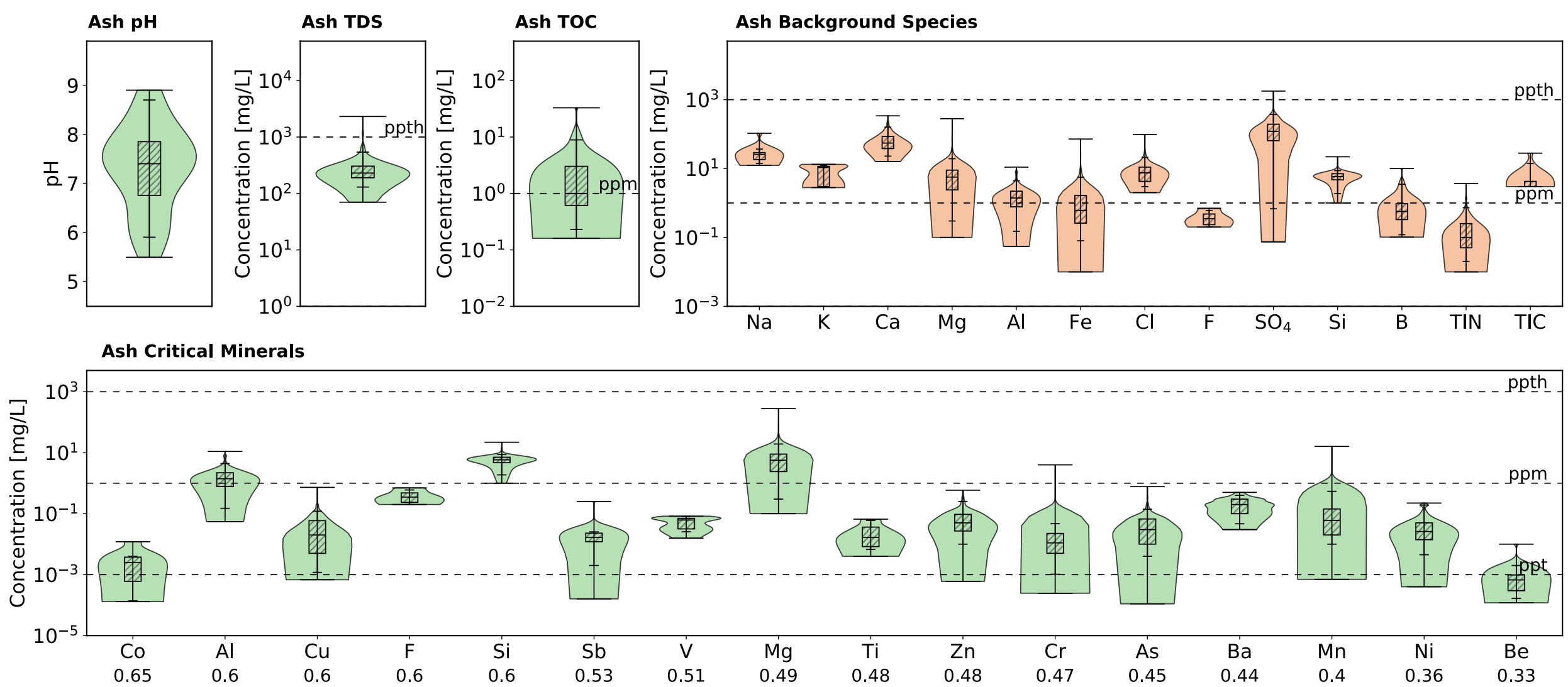

Figure S28. Bottom as transport wastewater pH, TDS, TOC, background species, and critical minerals concentration distribution.

Table S33. Bottom ash transport water characteristics. Values in parentheses represent the median concentrations. The presented data range is from the 5th to the 95th percentile.

Ash Transport					
Flow [Mm^3/year/EGU]		pH		TDS [mg/L]	TOC [mg/L]
0.10		5.81–8.73 (7.40)		129–540 (230)	0–10 (1.00)
**Wastewater Constituents**					
**Background Species**	**Concentration [mg/L]**	**Median Mineral Proportion in TDS [%]**	**Critical Minerals**	**Concentration [mg/L]**	**Criticality Rating**
SO_4	0.45–371 (120)	38	Al	0.08–4.45 (1.40)	0.6
TIC	4.80–230 (84)	26	Cu	0–0.11 (7.50×10^{-3})	0.6
Ca	23–160 (57)	18	F	0.07–0.66 (0.31)	0.6
Na	13–68 (26)	8.08	Sb	0–0.03 (0.01)	0.53
K	2.85–13 (11)	3.35	V	0–0.08 (0.04)	0.51
Cl	3.00–21 (7.00)	2.19	Mg	0.30–19 (5.65)	0.49
Si	1.85–8.71 (5.85)	1.83	Zn	0–0.24 (0.04)	0.48
Mg	0.30–19 (5.65)	1.77	Ti	0–0.06 (6.00×10^{-3})	0.48
Al	0.08–4.45 (1.40)	0.44	Cr	0–0.03 (3.00×10^{-3})	0.47
Fe	0.01–5.36 (0.57)	0.18	As	0–0.13 (0.02)	0.45
B	0.11–4.02 (0.55)	0.17	Ba	0–0.40 (0.10)	0.44
F	0.07–0.66 (0.31)	0.10	Mn	0–0.45 (0.04)	0.4
TIN	0–5.05 (0.20)	0.06	Ni	0–0.12 (5.67×10^{-3})	0.36

S9.5 Combustion residual leachate

Solid combustion byproducts generated from fossil fuel combustion are collected and disposed of in landfills. These include residual solids derived from the wastewater treatment processes above, specifically dewatered adsorbents utilized in FGD and bottom ash treatment.[263] The other byproducts are from chemicals used for environmental controls such as hydrated lime, trona, or powdered activated carbon.[263] CRL is created when liquids such as groundwater or rainwater pass through landfills and dissolve and transport the waste.

Figure S29 and **Table S34** show the typical volume and characteristics of CRL produced in the U.S.[264,265] Around 0.05 Mm^3 wastewater is generated per EGU. The pH values vary from 6.06 to 9.95 while TDS concentrations lie between 27 and 9,204 mg/L. The major background species are Na, Ca, Mg, Cl, and SO_4, with concentrations from a few hundred to a few thousand mg/L. The major CM is Mg, with concentration exceeding 10 mg/L; other CM concentrations are on the order of 0.01 mg/L.

CRL constituents vary depending on the type of fossil fuels combusted. For example, CRL from bituminous coal combustion generally contains higher lithium and manganese, whereas subbituminous coal combustion residual leachate has higher Hg and V.[266] Additionally, CRL contains similar minerals to FGD wastewater, but with lower concentrations and smaller volumes.[52]

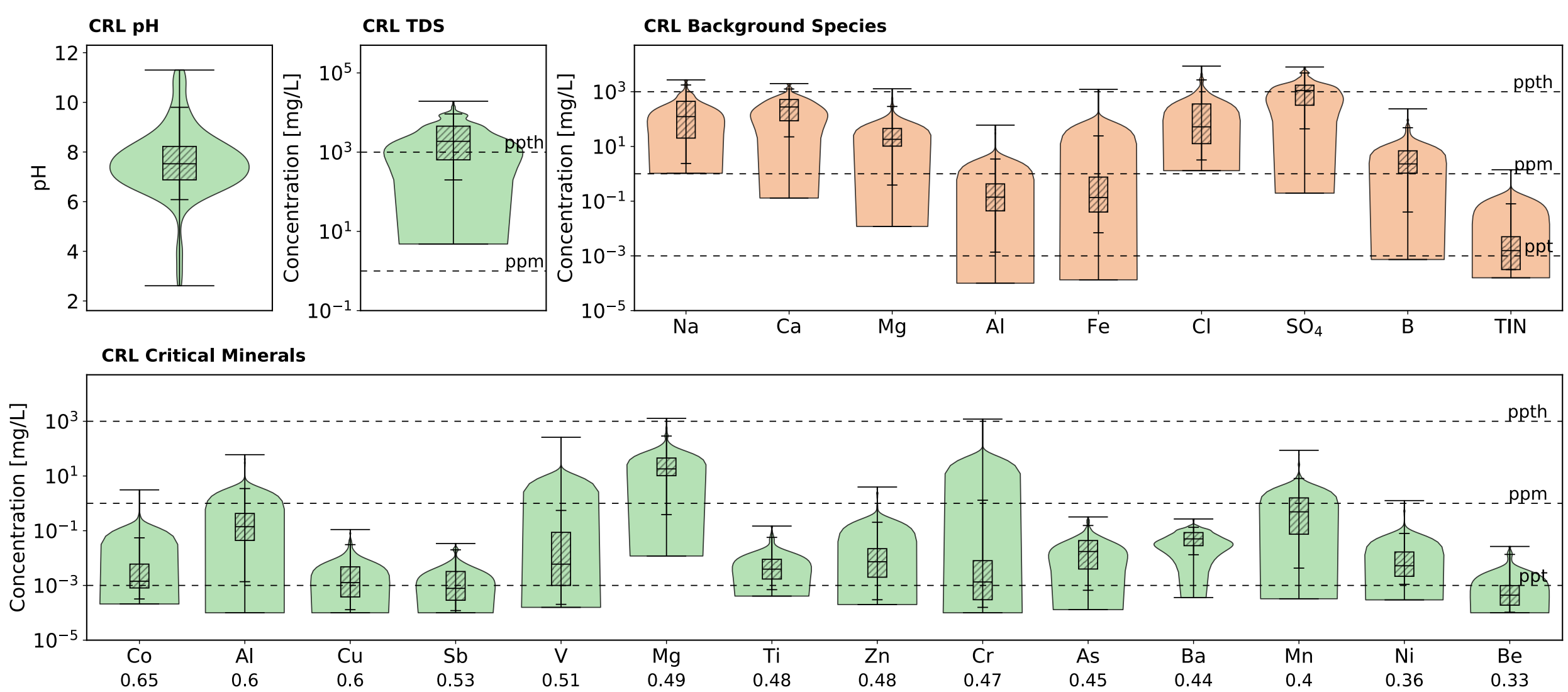

Figure S29. Combustion residual leachate pH, TDS, background species, and critical minerals concentration distribution.

Table S34. Combustion residual leachate characteristics. Values in parentheses represent the median concentrations. The presented data range is from the 5th to the 95th percentile.

Combustion Residual Leachate					
Flow [Mm^3/year/EGU]		pH		TDS [mg/L]	TOC [mg/L]
0.05		6.06–9.95 (7.53)		27–9,204 (1,853)	N/A
**Wastewater Constituents**					
**Background Species**	**Concentration [mg/L]**	**Median Mineral Proportion in TDS [%]**	**Critical Minerals**	**Concentration [mg/L]**	**Criticality Rating**
SO_4	2.16–4,892 (1,095)	70	Co	0–0.04 (9.60×10^{-4})	0.65
Ca	4.31–1,265 (266)	17	Al	0–3.11 (0.10)	0.6
Na	2.14–1,766 (120)	7.72	Cu	0–0.03 (5.15×10^{-4})	0.6
Cl	2.88–2,791 (52)	3.35	Sb	0–8.91×10^{-3} (1.59×10^{-4})	0.53
Mg	0.04–294 (18)	1.15	V	0–0.45 (8.15×10^{-4})	0.51
B	6.90×10^{-3}–48 (2.26)	0.15	Mg	0.04–294 (18)	0.49
Al	0–3.11 (0.10)	6.63×10^{-3}	Ti	0–0.04 (2.04×10^{-4})	0.48
Fe	0–20 (0.10)	6.35×10^{-3}	Zn	0–0.10 (2.84×10^{-3})	0.48
			Cr	0–0.37 (1.70×10^{-4})	0.47
			As	0–0.13 (0.01)	0.45
			Ba	1.75×10^{-4}–0.13 (0.05)	0.44
			Mn	0–8.07 (0.35)	0.4
			Ni	0–0.08 (3.71×10^{-3})	0.36

S9.6 Data sources

All our data sources represent power plants in the U.S. We report characteristics of wastewater measured directly at the source (i.e., prior to any treatment or control), and we include all background species and CMs that were reported in the original data, including those below the detection limit (which we treat as a concentration of zero, for statistical purposes). Some data were only reported as ranges rather than discrete values. In such cases, the reported minimum and maximum values were added to the overall dataset, and we report the overall minimum and maximum range, since calculating percentile from these extreme values would be misleading.

FGD wastewater data was obtained from the NEWTS EPA FGD Effluent Database (dated 2022)[256] and the power plant monitoring data of Eastern Research Group reported in literature

(dated 2016).[255,257] As noted above, only wastewater before treatment is included in analysis, which was achieved by utilizing solely "FGD Purge" data from NEWTS database. The median mineral proportions in TDS were calculated using only data from the NEWTS EPA FGD Effluent Database, because data in the Eastern Research Group database was not sufficiently detailed to make this calculation.

Bottom ash transport wastewater data was obtained from the NEWTS EPA Ash Database[261] by querying only "bottom ash effluent" and "combined ash effluent." We report the 5% and 95% quantile as well as the median value. The NEWTS database was dated 2023 and the bottom ash data were collected in specific time periods of 1973–1976 and 1995–2014.

Data on CRL was obtained from the NEWTS EPA Leachate Database (dated 2022) [267] and combustion product information (CPinfo) database from the Electrical Power Research Institute (EPRI, dated 2021).[264,265] This data includes the wastewater from both combustion residual landfills and impoundments. Because CPinfo only provided the minimum and maximum values, we report the minimum and maximum values for all data rather than the 5% and 95% quantile.

S9.7 Data limitations

Geographic limitation. Data included in our analysis only represents power plants in the U.S., because we were unable to find a comprehensive or reliable source of wastewater characteristics data for power plants elsewhere. According to the global database of power plants,[268] as of 2016, 30,084 power plants were operational around the world, of which 8,644 (28.7%) were located in the U.S. Therefore, our data represent a significant fraction of active power plants globally, but may not fully reflect variations in wastewater characteristics due to local coal sources, for example.

Did not account for the transition of treatment technology. In an effort to achieve net-zero emissions, fossil fuel power plants are gradually adopting zero liquid discharge systems, [52] resulting in a transition from wet to dry FGD systems.[254] Similarly, emerging policies may encourage a similar technical shift for bottom ash transport water treatment as well.[269] Thus, both the volume of wastewater per EGU and its characteristics may change in coming years. Our analysis makes no attempt to predict such changes; rather we offer a "snapshot" of current practices.

Limitations in types of fossil fuel plants. Most of the wastewater constituent data analyzed in this study are from coal-fired power plants, along with some datasets from facilities where the fuel source was not clearly identified. Although our analysis is not exclusively based on coal-fired power plants, it is largely limited to coal-fired power plants which have sufficiently detailed data for analysis. Currently, coal is the most dominant fossil fuel for electricity generation globally, while natural gas predominates in the U.S.

Section S10. Geothermal Power Generation

S10.1 Industry description

Geothermal power generation is the process of converting hydrothermal resources (hot geofluid), such as hot water and steam, into electricity. Typically, geofluids are extracted from their reservoirs and routed through turbines or heat exchangers to generate electricity, then discharged after losing their heat. Like hydrocarbons, geofluids reservoirs are located deep underground, and many are trapped in porous rocks. Production wells are drilled to access them, while injection wells are used to dispose of spent fluids and potentially regenerate the reservoir. As of 2022, there are 673 geothermal power units in operation globally, which generates approximately 97 billion kWh of electricity.[270] The U.S. and Indonesia are the top two contributors, each accounting for around 18% (17 billion kWh) of the world's total geothermal energy generation. Recent advances in geothermal technologies (e.g., enhanced geothermal systems) and the initiative to co-produce geofluids with hydrocarbon[55] have encouraged the expansion of existing plants[271,272] and construction of new plants[273,274] around the world.

S10.2 Overview of geothermal wastewater streams

Wastewater generated from geothermal power is primarily drilling wastewater and spent geofluids (also categorized as "PW")[88]. This closely resembles wastewater generation in the oil and gas industry due to geological similarities between hydrothermal and hydrocarbon reservoirs, as well as the shared extraction technologies such as drilling and hydraulic stimulation.[275,276] Due to data limitation, for drilling wastewater properties we refer to the oil and gas industry. The properties of the geothermal PW are discussed below.

Figure S30 and **Table S35** show the geothermal PW constituents. For 1 kWh energy produced, 3.13-1126 kg of PW is generated (See SI for calculations).[56,276] The pH ranges from 5.16 to 8.90. The TDS ranges from 57 to 4,439 mg/L. The major environmental species are Na and Cl, with concentration reaching a thousand mg/L. The TOC is not reported, The major CMs are F, Al, Mg, and Li, with concentrations range from a few to hundred mg/L; others CMs are found to be below 1 mg/L.

The flow rate of PW depends on geofluid enthalpy and the geothermal-to-electricity conversion efficiency.[56] Low geofluid enthalpy and conversion efficiency lead to a high PW flow rate. The water constituents strongly resemble oil and gas PW due to the geological similarity between the hydrothermal and hydrocarbon reservoirs, although the concentrations of all minerals are lower.

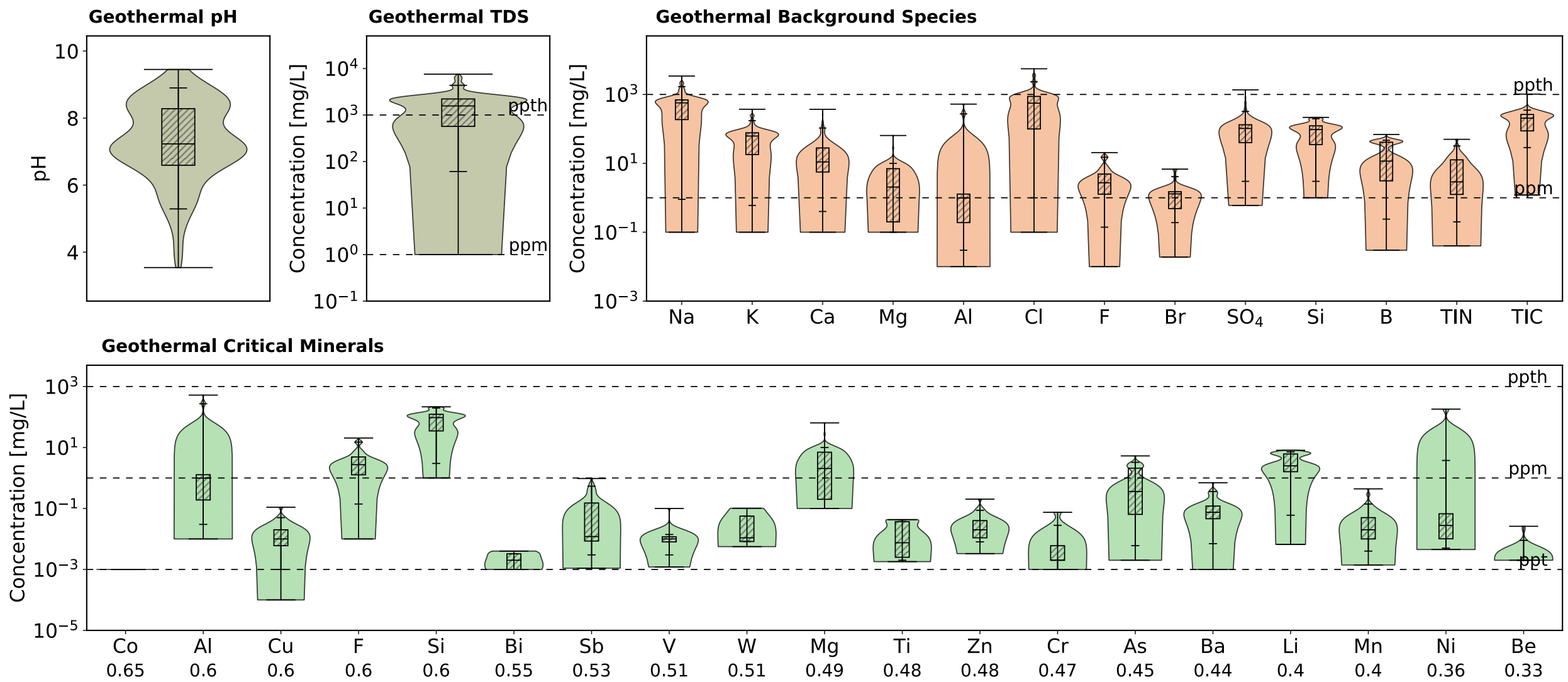

Figure S30. Geothermal produced water pH, TDS, background species, and critical minerals concentration distribution.

Table S35. Produced water from geothermal power generation. Values in parentheses represent the median concentrations. The range represents the 5th to the 95th percentile.

Geothermal					
Flow [kg/kwh]		pH		TDS [mg/L]	TOC [mg/L]
3.13–1,126 (15)		5.16–8.90 (7.23)		57–4,439 (1,561)	N/A
**Wastewater Constituents**					
**Background Species**	**Concentration [mg/L]**	**Median Mineral Proportion in TDS [%]**	**Critical Minerals**	**Concentration [mg/L]**	**Criticality Rating**
Na	0.84–1,689 (571)	38	Co	1.00×10^{-3} (1.00×10^{-3})	0.65
Cl	0.97–2,340 (558)	37	Cu	1.00×10^{-3}–0.05 (0.01)	0.6
SO_4	2.92–332 (103)	6.83	Al	0.03–275 (0.99)	0.6
Si	3.00–197 (96)	6.34	F	0.14–15 (2.73)	0.6
TIC	0.15–313 (80)	5.31	Bi	1.00×10^{-3}–3.85×10^{-3} (2.00×10^{-3})	0.55
K	0.53–174 (62)	4.11	Sb	3.00×10^{-3}–0.62 (0.01)	0.53
B	0.23–47 (12)	0.77	W	6.13×10^{-3}–0.09 (0.01)	0.51
Ca	0.40–108 (11)	0.73	V	3.00×10^{-3}–0.02 (0.01)	0.51
F	0.14–15 (2.73)	0.18	Mg	0.10–11 (2.05)	0.49
Mg	0.10–11 (2.05)	0.14	Ti	1.88×10^{-3}–0.04 (7.50×10^{-3})	0.48
TIN	0.02–19 (1.57)	0.10	Zn	7.20×10^{-3}–0.10 (0.02)	0.48
Br	0.16–5.03 (1.30)	0.09	Cr	1.00×10^{-3}–0.05 (2.00×10^{-3})	0.47
Al	0.03–275 (0.99)	0.07	As	6.00×10^{-3}–3.26 (0.36)	0.45
			Ba	6.50×10^{-3}–0.36 (0.07)	0.44
			Mn	3.40×10^{-3}–0.22 (0.02)	0.4
			Li	0.06–7.20 (2.50)	0.4
			Ni	4.75×10^{-3}–93 (0.03)	0.36
			Be	2.00×10^{-3}–0.02 (2.00×10^{-3})	0.33

S10.3 Data sources

Most of our data sources were from the U.S., with limited data from other countries including Switzerland and New Zealand. We report characteristics of wastewater measured directly at the source (i.e., prior to any treatment or control), and we include all environmental species and CMs that were reported in the original data, including those below the detection limit.

Data on wastewater characteristics were obtained from the USGS database[88], dated 2023. Data on wastewater generation were obtained from journal articles[56] and technical reports from ANL[276], date 2009.

S10.4 Data limitations

Because geofluids reservoirs closely resemble hydrocarbon reservoirs in terms of geological formation, and because the geothermal wastewater data used in our study come from the same source as oil and gas produced water (the USGS National Produced Waters Geochemical Database), we anticipate similar data limitations for these two industries. Therefore, we refer readers to our discussion of data limitations for oil and gas extraction **(S6.7)**.

Section S11. Gasification

S11.1 Industry description

Gasification is a thermochemical process that converts carbon-based feedstocks, such as coal, biomass, or municipal waste, into a synthetic gas (syngas) composed mainly of hydrogen (H_2) and carbon monoxide (CO).[277] The syngas can be subsequently converted to methane or other liquid fuels.[278] In a typical gasification process, feedstocks are pretreated to have desired gasification properties, then sent into a chamber (gasifier) with controlled temperature, pressure, oxygen content, and steam, resulting in incomplete combustion. Syngas is then produced from the gasifier, along with byproducts including waste gases (e.g., acid gases such as hydrogen sulfide and hydrogen chloride), char, and ash. Purification of syngas is done by passing the raw gas through a series of gas scrubbers and water condensers.[277,279] Coal has historically been the primary gasification feedstock, although new plants that use waste or biomass-based feedstocks that contribute to lower carbon emissions are being developed worldwide.[280,281] As of 2019, there are 675 gasification sites in operation or under construction worldwide, more than 70% are located in China, and around 4% are located in the U.S.[282]

S11.2 Overview of gasification wastewater streams

Wastewater generated from gasification is primarily composed of process water, which includes condensed steam water from combusting the feedstock and water used to purify and cool the syngas. The water flow rate, as well as the water characteristic, is highly dependent on the feedstock properties and the gasifier type. We estimate that the worldwide gasification wastewater generation is around 95.13 Mm^3 per year.[59–61]

Figure S31 and **Table S36** show the characteristics of the coal gasification of wastewater. For 1 Mm^3 of gas produced, 3,280 to 6,900 m^3 of wastewater is being generated.[59,60] The pH of gasification wastewater ranges from 6 to 10. The TDS ranges from 9.35 to 26,750 mg/L. The TOC is high, ranging from 16 to 2,500 mg/L. The major environmental species are Na, Al, SO_4, and TIN, with concentration of a few thousands of mg/L. The wastewater can have a high concentration of nitrogen compounds (0–11,295 mg/L) and is dominated by dissolved ammonia produced during syngas generation. The major CMs are Al, Mg, and Ni, with concentrations reaching tens of mg/L; other CMs have concentrations below 1 mg/L.

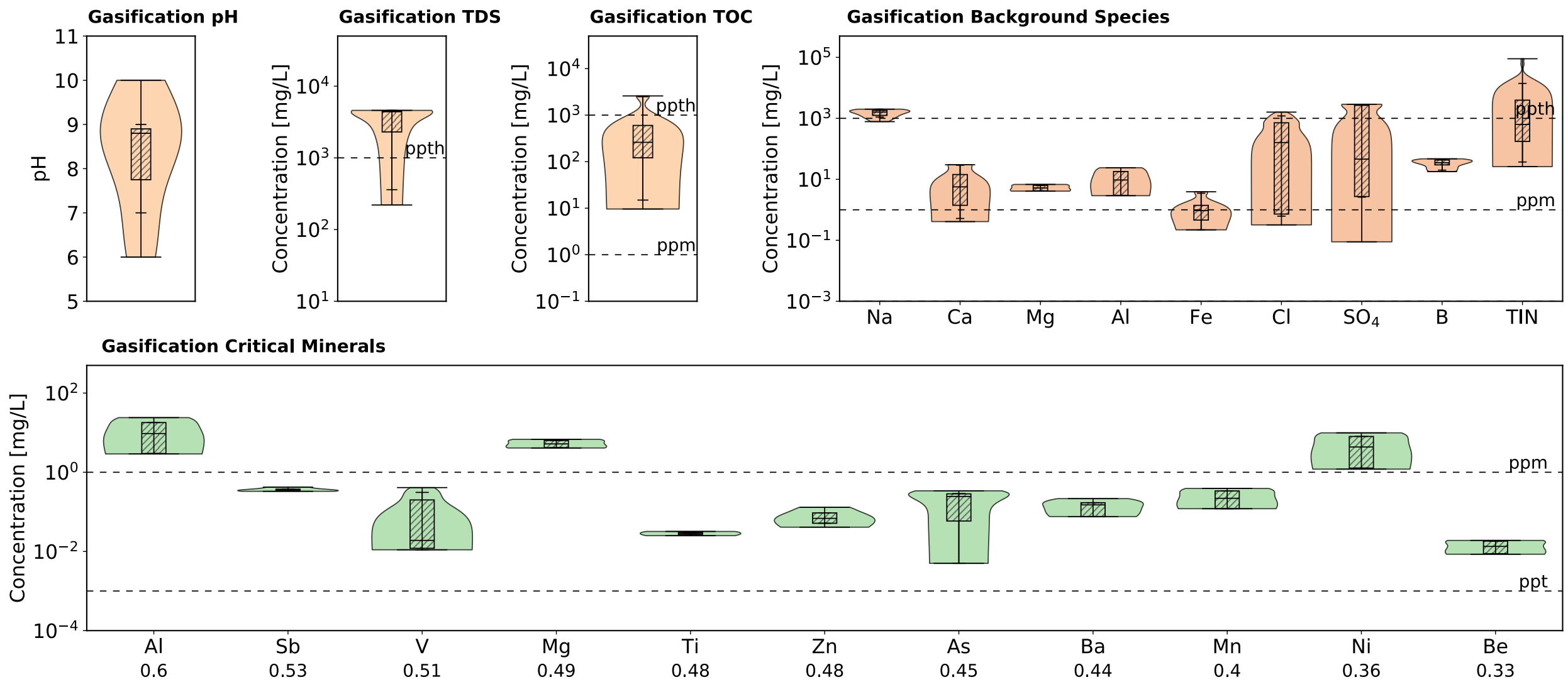

Figure S31. Gasification wastewater pH, TDS, TOC, background species, and critical minerals concentration distribution.

Table S36. Gasification wastewater quality. Values in parentheses represent the median concentrations. The presented data range is from the 5th to the 95th percentile.

Gasification					
Flow [m^3/Mm^3-gas]		pH		TDS [mg/L]	TOC [mg/L]
3,280–6,900 (5,329)		6.30–9.70 (8.80)		0–4,600 (4,000)	14–2,515 (260)
**Wastewater Constituents**					
**Background Species**	**Concentration [mg/L]**	**Median Mineral Proportion in TDS [%]**	**Critical Minerals**	**Concentration [mg/L]**	**Criticality Rating**
SO_4	1,093–22,400 (2,900)	54	Al	2.92–22 (16)	0.6
Na	1,020–10,750 (1,750)	32	Cu	4.46×10^{-3}–7.75×10^{-3} (7.30×10^{-3})	0.6
Cl	170–1,505 (700)	13	Sb	0.33–0.41 (0.35)	0.53
Al	2.92–22 (16)	0.30	V	0.01–0.36 (0.02)	0.51
Ca	0.47–29 (10)	0.19	Mg	3.30–6.80 (4.00)	0.49
Mg	3.30–6.80 (4.00)	0.07	Ti	0.03 (0.03)	0.48
TIN	0.11–60,000 (2.10)	0.04	Zn	0.04–0.12 (0.07)	0.48
			Cr	0.02–0.08 (0.02)	0.47
			As	0.15–0.32 (0.20)	0.45
			Mn	0.04–0.38 (0.12)	0.4
			Ni	0.03–9.03 (0.62)	0.36
			Be	8.55×10^{-3}–0.02 (0.01)	0.33

S11.3 Data sources

Most of our data sources were from the U.S. and China. We report characteristics of wastewater measured directly at the source (i.e., prior to any treatment or control), and we include all background species and CMs that were reported in the original data, including those below the detection limit.

Data on wastewater characteristics were obtained from the NEWTS EPA Gasification Dataset and Case Studies[283] and journal articles[60,284,285] , dated from 2006 to 2023. Data on wastewater generation were obtained from a journal article[60] and a technical report from NETL[59], dated 2022 to 2023.

S11.4 Data limitations

Limitations in types of gasification plants. A variety of feedstocks can be used for gasification, such as coal, biomass, and municipal solid waste. However, our data only include coal-based gasification. Depending on the feedstock, differences in wastewater composition are anticipated. For example, higher TIN is expected for biomass-based gasification wastewater[286], whereas higher metal content is expected for municipal waste-based[287], according to their representative elemental compositions.

Section S12. Treated Wastewater volume and CM content

The primary analysis target of this study was wastewater before any treatment. However, some wastewater sources may subject to primary treatment before discharge to local watersheds or centralized treatment facilities. If the discharged wastewater has high volume and CM concentration, it could also be a valuable target for critical mineral recovery. Therefore, in this section, we analyzed the industrial wastewater discharge data from the 2024 U.S. EPA's Discharge Monitoring Report (DMR) database, which include the volume and concentration of CMs. The DMR database tracks wastewater discharged from 59 industries; we selected only the 9 industries that are relevant to our study. The selected industries are listed in **Table S37**, and the volume and CM concentration are listed in **Table S38**–**Table S46**. For each industry, multiple sub-processes (represented by SIC codes) are included. For simple demonstration, when more than five sub-processes are present, only the five largest contributors to critical mineral discharge in wastewater are listed in the table.

Table S37. Summary of wastewater discharge data from selected industries. 40 CFR is title 40 of the U.S. code of federal regulations.

40 CFR	Industry	Wastewater discharge [Mm^3 / yr]	Proportion of total discharged wastewater [%]	CMs discharge [ton/yr]	# of reported facilities
423	Steam electric power generating	43,625.6	41.67	8,630.3	1,189
433	Metal finishing	2,576.1	2.44	736.2	3,340
419	Petroleum refining	1,875.9	1.78	402.1	1,323
434	Coal mining	631.3	0.61	3,226.5	2,786
440	Ore mining and dressing	444.2	0.42	770.4	191
435	Oil and gas extraction	335.3	0.32	968.5	1,380
425	Leather training and finishing	0.6	0.005	1.0	5
461	Battery manufacturing	0.5	0.004	12.0	13
469	Electrical and electronic components	0.4	0.004	9.3	18

S12.1 Oil and gas extraction wastewater discharge volume and CM content

The characteristics of wastewater from **oil and gas extraction**—corresponding to **Section S6** except **S6.5**—is shown in **Table S38**. Oil and gas extraction industries discharge around 335.3 Mm^3 of wastewater every year, carrying 968.5 tons of CMs every year.

Table S38. Average wastewater discharge rate (million m^3/year) and total CM discharge rate (kg/year) from oil and gas extraction. The facilities are categorized based on the Standard Industrial Classification (SIC) Code (listed in square brackets). The CMs for energy are color coded based on the DOE's criticality rating, with red as "critical" and yellow as "near critical".[104] All data were acquired from 2024 DMR database.[29] The "Total" column represents the total wastewater discharge volume and the mass of each mineral from the sub-processes listed in the table. The contribution of the listed sub-process to the total across all sub-processes is indicated in parentheses.

Total Wastewater and Metal Discharge From Oil And Gas Extraction							
		Crude Petroleum And Natural Gas [1311]	Oil And Gas Field Services, Nec [1389]	Gas Production And/or Distribution [4925]	Oil And Gas Exploration Services [1382]	Drilling Oil And Gas Wells [1381]	Total
Avg. Flow [Mm^3/yr]		324.1	3.4	1.3	6.5	0.0	335.3(100%)
Supply Risk Rating	Mineral	Discharge [kg/yr]					
0.6	Aluminum	6.6	58.4	7.8	0.0	0.0	72.9(100%)
0.49	Magnesium	967154.7	0.0	0.0	0.0	0.0	967154.7(100%)
0.48	Zinc	714.6	25.9	14.0	0.0	0.0	754.5(100%)
0.47	Chromium	4.9	2.8	0.0	0.0	0.0	7.6(100%)
0.45	Arsenic	342.3	0.0	0.0	0.0	0.0	342.3(100%)
0.4	Manganese	99.0	0.6	0.0	0.0	0.0	99.6(100%)
0.36	Nickel	12.1	3.0	0.0	0.0	0.0	15.1(100%)
0.34	Copper	58.1	4.1	1.4	0.0	0.0	63.5(100%)
0.33	Beryllium	0.0	2.0	0.0	0.0	0.0	2.0(100%)
Total Discharge [ton/yr]		968.4	0.1	0.0	0.0	0.0	968.5(100%)

S12.2 Petroleum refining wastewater discharge volume and CM content

The characteristics of wastewater from **petroleum refining**—corresponding to **S6.5 Petroleum refining**—is shown in **Table S39**. Petroleum refining industries discharge around 1875.9 Mm^3 of wastewater every year, carrying 402.1 tons of CMs every year.

Table S39. Average wastewater discharge rate (million m^3/year) and total CM discharge rate (kg/year) from petroleum refining. The facilities are categorized based on the Standard Industrial Classification (SIC) Code (listed in square brackets). The CMs for energy are color coded based on the DOE's criticality rating, with red as "critical" and yellow as "near critical".[104] All data were acquired from 2024 DMR database.[29] The "Total" column represents the total wastewater discharge volume and the mass of each mineral from the sub-processes listed in the table. The contribution of the listed sub-process to the total across all sub-processes is indicated in parentheses.

Total Wastewater and Metal Discharge From Petroleum Refining							
		Petroleum Refining [2911]	Petroleum And Coal Products, Nec [2999]	Lubricating Oils And Greases [2992]	Petroleum Bulk Stations And Terminals [5171]	Crude Petroleum Pipelines [4612]	Total
Avg. Flow [Mm^3/yr]		438.3	17.0	5.2	1411.4	4.1	1875.9(100%)
Supply Risk Rating	Mineral	Discharge [kg/yr]					
0.6	Uranium-238	1.4	0.0	0.0	0.0	0.0	1.4(100%)
0.6	Aluminum	86639.2	548.1	73.6	39.0	8.4	87308.3(100%)
0.53	Antimony	25.0	0.0	0.0	4.5	0.0	29.5(100%)
0.51	Vanadium	306.7	0.0	0.0	0.0	0.0	306.7(100%)
0.49	Magnesium	284198.1	0.0	0.0	0.0	0.0	284198.1(100%)
0.48	Zinc	17004.6	32.4	632.5	284.5	61.5	18015.5(100%)
0.47	Chromium	853.7	0.1	0.0	51.3	0.0	905.1(100%)
0.45	Arsenic	1079.6	0.0	0.0	8.7	0.0	1088.3(100%)
0.4	Lithium	11.2	0.0	0.0	0.0	0.0	11.2(100%)
0.4	Manganese	6255.0	187.6	0.0	0.0	0.0	6442.6(100%)
0.36	Nickel	1893.9	0.0	0.1	5.1	0.0	1899.0(100%)
0.34	Copper	1786.5	35.5	0.3	35.9	1.6	1859.8(100%)
0.33	Beryllium	2.3	0.0	0.0	0.0	0.0	2.3(100%)
Total Discharge [ton/yr]		400.1	0.8	0.7	0.4	0.1	402.1(100%)

S12.3 Ore mining and dressing wastewater discharge volume and CM content

The characteristics of wastewater from **ore mining and dressing**—corresponding to **Section S7 Metal Ore and Coal Mining**—is shown in **Table S40**. Ore mining and dressing industries discharge around 444.2 Mm^3 of wastewater every year, carrying 770.4 tons of CMs every year.

Table S40. Average wastewater discharge rate (million m^3/year) and total CM discharge rate (kg/year) from ore mining and dressing. The facilities are categorized based on the Standard Industrial Classification (SIC) Code (listed in square brackets). The CMs for energy are color coded based on the DOE's criticality rating, with red as "critical" and yellow as "near critical".[104] All data were acquired from 2024 DMR database.[29] The "Total" column represents the total wastewater discharge volume and the mass of each mineral from the sub-processes listed in the table. The contribution of the listed sub-process to the total across all sub-processes is indicated in parentheses.

Total Wastewater and Metal Discharge From Ore Mining And Dressing							
		Gold Ores [1041]	Uranium-radium-vanadium Ores [1094]	Iron Ores [1011]	Lead And Zinc Ores [1031]	Metal Ores, Nec [1099]	Total
Avg. Flow [Mm^3/yr]		39.8	1.4	237.0	93.7	10.6	444.2(86%)
Supply Risk Rating	Mineral	Discharge [kg/yr]					
0.65	Cobalt	0.0	0.0	0.0	160.9	0.0	162.6(99%)
0.6	Uranium-238	133.7	766.3	0.0	0.0	0.0	904.9(99%)
0.6	Aluminum	1790.6	0.0	6959.3	2347.6	3003.4	14139.4(100%)
0.53	Antimony	102.6	0.1	0.0	56.0	0.3	160.6(99%)
0.51	Vanadium	0.0	100939.4	0.0	0.0	0.0	100939.4(100%)
0.49	Magnesium	491377.6	0.0	76963.7	0.0	0.0	568341.3(100%)
0.48	Zinc	306.7	1.1	1098.5	72493.3	17.9	74802.9(99%)
0.47	Chromium	0.6	0.0	0.0	0.0	0.0	2.3(24%)
0.45	Arsenic	513.4	1.1	122.7	23.4	0.0	893.2(74%)
0.4	Manganese	2582.5	131.1	0.0	626.9	216.4	5795.7(61%)
0.36	Nickel	62.4	0.0	947.5	759.7	5.4	2060.8(86%)
0.34	Copper	50.5	0.8	611.1	949.3	2.2	2092.0(77%)
0.33	Beryllium	0.0	0.0	121.6	0.0	0.0	121.6(100%)
Total Discharge [ton/yr]		496.9	101.8	86.8	77.4	3.2	770.4(99%)
Only the first 5 out of 9 SIC codes are presented							

S12.4 Coal mining wastewater discharge volume and CM content

The characteristics of wastewater from **coal mining** is shown in **Table S41**. Coal mining industries discharge around 631.3 Mm^3 of wastewater every year, carrying 3226.5 tons of CMs every year.

Table S41. Average wastewater discharge rate (million m^3/year) and total CM discharge rate (kg/year) from coal mining. The facilities are categorized based on the Standard Industrial Classification (SIC) Code (listed in square brackets). The CMs for energy are color coded based on the DOE's criticality rating, with red as "critical" and yellow as "near critical".[104] All data were acquired from 2024 DMR database.[29] The "Total" column represents the total wastewater discharge volume and the mass of each mineral from the sub-processes listed in the table. The contribution of the listed sub-process to the total across all sub-processes is indicated in parentheses.

Total Wastewater and Metal Discharge From Coal Mining					
		Bituminous Coal And Lignite-surface Mining [1221]	Bituminous Coal-underground Mining [1222]	Anthracite Mining [1231]	Total
Avg. Flow [Mm^3/yr]		595.0	32.2	4.2	631.3(100%)
Supply Risk Rating	Mineral	Discharge [kg/yr]			
0.6	Aluminum	462490.5	4972.5	815.4	468278.4(100%)
0.53	Antimony	19.9	0.0	0.0	19.9(100%)
0.49	Magnesium	2232628.4	6413.4	0.0	2239041.8(100%)
0.48	Zinc	16525.7	205.4	0.0	16731.1(100%)
0.47	Chromium	17.1	6.5	0.0	23.6(100%)
0.45	Arsenic	157.2	6.5	0.0	163.6(100%)
0.4	Manganese	372675.2	31160.0	8551.0	412386.2(100%)
0.36	Nickel	87838.1	408.4	0.0	88246.5(100%)
0.34	Copper	1564.2	51.1	0.0	1615.3(100%)
0.33	Beryllium	10.3	0.0	0.0	10.3(100%)
Total Discharge [ton/yr]		3173.9	43.2	9.4	3226.5(100%)

S12.5 Semiconductor manufacturing wastewater discharge volume and CM content

The characteristics of wastewater from **electric and electronic components**—corresponding to **S8.1 Semiconductor manufacturing wastewater**—is shown in **Table S42**. Electric and electronic components industries discharge around 0.4 Mm^3 of wastewater every year, carrying 9.3 tons of CMs every year.

Table S42. Average wastewater discharge rate (million m^3/year) and total CM discharge rate (kg/year) from electric and electronic components. The facilities are categorized based on the Standard Industrial Classification (SIC) Code (listed in square brackets). The CMs for energy are color coded based on the DOE's criticality rating, with red as "critical" and yellow as "near critical".[104] All data were acquired from 2024 DMR database.[29] The "Total" column represents the total wastewater discharge volume and the mass of each mineral from the sub-processes listed in the table. The contribution of the listed sub-process to the total across all sub-processes is indicated in parentheses.

Total Wastewater and Metal Discharge From Electrical And Electronic Components				
		Semiconductors And Related Devices [3674]	Electron Tubes [3671]	Total
Avg. Flow [Mm^3/yr]		0.4	0.0	0.4(100%)
Supply Risk Rating	Mineral	Discharge [kg/yr]		
0.6	Aluminum	57.6	0.0	57.6(100%)
0.49	Magnesium	9220.5	0.0	9220.5(100%)
0.48	Zinc	2.4	0.0	2.4(100%)
0.4	Manganese	4.4	0.0	4.4(100%)
0.34	Copper	0.4	0.0	0.4(100%)
Total Discharge [ton/yr]		9.3	0.0	9.3(100%)

S12.6 Leather tanning and finishing wastewater discharge volume and CM content

The characteristics of wastewater from **leather tanning and finishing**—corresponding to **S8.2**—is shown in **Table S43**. Leather tanning and finishing industries discharge around 0.6 Mm^3 of wastewater every year, carrying 1.0 tons of CMs every year.

Table S43. Average wastewater discharge rate (million m^3/year) and total CM discharge rate (kg/year) from leather training and finishing. The facilities are categorized based on the Standard Industrial Classification (SIC) Code (listed in square brackets). The CMs for energy are color coded based on the DOE's criticality rating, with red as "critical" and yellow as "near critical".[104] All data were acquired from 2024 DMR database.[29] The "Total" column represents the total wastewater discharge volume and the mass of each mineral from the sub-processes listed in the table. The contribution of the listed sub-process to the total across all sub-processes is indicated in parentheses.

Total Wastewater and Metal Discharge From Leather Tanning And Finishing			
		Leather Tanning And Finishing [3111]	Total
Avg. Flow [Mm^3/yr]		0.6	0.6(100%)
Supply Risk Rating	Mineral	Discharge [kg/yr]	
0.6	Aluminum	1015.4	1015.4(100%)
0.48	Zinc	24.6	24.6(100%)
0.34	Copper	4.8	4.8(100%)
Total Discharge [ton/yr]		1.0	1.0(100%)

S12.7 Metal plating wastewater discharge volume and CM content

The characteristics of wastewater from **metal finishing**—corresponding to **S8.3 Metal plating and finishing**—is shown in **Table S44**. Metal finishing industries discharge around 2576.1 Mm^3 of wastewater every year, carrying 736.2 tons of CMs every year.

Table S44. Average wastewater discharge rate (million m^3/year) and total CM discharge rate (kg/year) from metal finishing. The facilities are categorized based on the Standard Industrial Classification (SIC) Code (listed in square brackets). The CMs for energy are color coded based on the DOE's criticality rating, with red as "critical" and yellow as "near critical".[104] All data were acquired from 2024 DMR database.[29] The "Total" column represents the total wastewater discharge volume and the mass of each mineral from the sub-processes listed in the table. The contribution of the listed sub-process to the total across all sub-processes is indicated in parentheses.

Total Wastewater and Metal Discharge From Metal Finishing							
		Aircraft Engines And Engine Parts [3724]	Plating And Polishing [3471]	Shipbuilding And Repairing [3731]	Photographic Equipment And Supplies [3861]	Metal Coating And Allied Services [3479]	Total
Avg. Flow [Mm^3/yr]		14.2	9.7	140.7	5.9	3.3	2576.1(7%)
Supply Risk Rating	Mineral	Discharge [kg/yr]					
0.65	Cobalt	0.0	15.1	0.1	5.7	0.0	54.0(39%)
0.6	Uranium-238	0.0	0.0	0.0	0.0	0.0	183.9(0%)
0.6	Aluminum	76222.7	687.3	159332.5	4407.1	2262.0	285042.1(85%)
0.53	Antimony	0.0	0.0	0.0	82.6	0.0	85.0(97%)
0.51	Tungsten	0.0	782.6	0.0	0.0	0.0	782.6(100%)
0.5	Tin	0.0	0.3	0.0	0.0	0.0	0.3(100%)
0.49	Magnesium	131487.9	545.2	0.0	48996.3	0.0	188175.6(96%)
0.48	Zinc	639.4	108528.8	12405.7	758.8	14816.6	146163.1(94%)
0.48	Titanium	0.0	0.0	0.0	0.0	0.0	86.5(0%)
0.47	Chromium	256.0	7577.3	0.4	67.5	49.1	8086.5(98%)
0.45	Arsenic	0.0	2.2	1077.2	0.9	0.0	1128.7(96%)
0.4	Lithium	0.0	0.0	0.0	0.0	0.0	338.0(0%)
0.4	Manganese	1.1	9.6	1.6	0.0	1913.9	3395.8(57%)
0.36	Nickel	274.2	8265.3	1309.2	107.0	52.9	10725.8(93%)
0.34	Copper	49.8	79404.8	11105.6	86.1	160.1	91914.6(99%)
Total Discharge [ton/yr]		208.9	205.8	185.2	54.5	19.3	736.2(92%)
Only the first 5 out of 161 SIC codes are presented							

S12.8 Battery manufacturing wastewater discharge volume and CM content

The characteristics of wastewater from **battery manufacturing**—corresponding to **S8.4 Battery manufacturing wastewater**—is shown in **Table S45**. Battery manufacturing industries discharge around 0.5 Mm^3 of wastewater every year, carrying 12.0 tons of CMs every year.

Table S45. Average wastewater discharge rate (million m^3/year) and total CM discharge rate (kg/year) from battery manufacturing. The facilities are categorized based on the Standard Industrial Classification (SIC) Code (listed in square brackets). The CMs for energy are color coded based on the DOE's criticality rating, with red as "critical" and yellow as "near critical".[104] All data were acquired from 2024 DMR database.[29] The "Total" column represents the total wastewater discharge volume and the mass of each mineral from the sub-processes listed in the table. The contribution of the listed sub-process to the total across all sub-processes is indicated in parentheses.

Total Wastewater and Metal Discharge From Battery Manufacturing				
		Primary Batteries, Dry And Wet [3692]	Storage Batteries [3691]	Total
Avg. Flow [Mm^3/yr]		0.1	0.4	0.5(100%)
Supply Risk Rating	Mineral	Discharge [kg/yr]		
0.53	Antimony	1.1	0.0	1.1(100%)
0.48	Zinc	9.8	0.0	9.8(100%)
0.45	Arsenic	0.3	0.0	0.3(100%)
0.36	Nickel	0.2	0.0	0.2(100%)
0.34	Copper	0.2	0.4	0.6(100%)
Total Discharge [kg/yr]		11.7	0.4	12.0(100%)

S12.9 Steam electric power generation wastewater discharge volume and CM content

The characteristics of wastewater from **steam electric power generating**—corresponding to Section S9 Fossil Fuel Power —is shown in **Table S46**. Steam electric power generating industries discharge around 43625.6 Mm^3 of wastewater every year, carrying 8630.3 tons of CMs every year.

Table S46. Average wastewater discharge rate (million m^3/year) and total CM discharge rate (kg/year) from steam electric power generating. The facilities are categorized based on the Standard Industrial Classification (SIC) Code (listed in square brackets). The CMs for energy are color coded based on the DOE's criticality rating, with red as "critical" and yellow as "near critical".[104] All data were acquired from 2024 DMR database.[29] The "Total" column represents the total wastewater discharge volume and the mass of each mineral from the sub-processes listed in the table. The contribution of the listed sub-process to the total across all sub-processes is indicated in parentheses.

Total Wastewater and Metal Discharge From Steam Electric Power Generating						
		Electric Services [4911]	Steam And Air-conditioning Supply [4961]	Combination Utilities, Nec [4939]	Electric And Other Services Combined [4931]	Total
Avg. Flow [Mm^3/yr]		43457.9	93.3	4.6	69.9	43625.6(100%)
Supply Risk Rating	Mineral	Discharge [kg/yr]				
0.65	Cobalt	6.5	0.0	0.0	0.0	6.5(100%)
0.6	Uranium-238	20.7	0.0	0.0	0.0	20.7(100%)
0.6	Aluminum	2836370.2	1229583.7	27.1	1647.4	4067628.4(100%)
0.53	Antimony	44913.3	0.0	0.0	0.1	44913.4(100%)
0.51	Vanadium	70.9	0.0	0.0	0.0	70.9(100%)
0.49	Magnesium	3130453.7	406365.9	0.0	0.0	3536819.7(100%)
0.48	Zinc	325791.6	110.6	38.6	141.2	326082.0(100%)
0.48	Titanium	140.7	0.0	0.0	0.0	140.7(100%)
0.47	Chromium	9266.2	0.0	0.0	8.4	9274.5(100%)
0.45	Arsenic	13573.6	616.0	1.2	15.4	14206.3(100%)
0.4	Lithium	395.1	0.0	0.0	0.0	395.1(100%)
0.4	Manganese	130035.2	0.0	9.5	67.7	130112.4(100%)
0.36	Nickel	37381.8	0.0	0.0	1.5	37383.2(100%)
0.34	Copper	313074.5	1818.2	148170.1	41.9	463104.6(100%)
0.33	Beryllium	162.3	0.0	0.0	0.0	162.3(100%)
Total Discharge [ton/yr]		6841.7	1638.5	148.2	1.9	8630.3(100%)

References

(1) Dubber, D.; Gray, N. F. Replacement of Chemical Oxygen Demand (COD) with Total Organic Carbon (TOC) for Monitoring Wastewater Treatment Performance to Minimize Disposal of Toxic Analytical Waste. *J Environ Sci Health A Tox Hazard Subst Environ Eng* **2010**, *45* (12), 1595–1600. https://doi.org/10.1080/10934529.2010.506116.

(2) Charlton, S. R.; Parkhurst, D. L. Modules Based on the Geochemical Model PHREEQC for Use in Scripting and Programming Languages. *Computers & Geosciences* **2011**, *37* (10), 1653–1663. https://doi.org/10.1016/j.cageo.2011.02.005.

(3) Parkhurst, D. L.; Appelo, C. a. J. Description of Input and Examples for PHREEQC Version 3 — A Computer Program for Speciation, Batch-Reaction, One-Dimensional Transport, and Inverse Geochemical Calculations. In *Modeling Techniques, Book 6*; 2013; pp 1–678.

(4) Anne E. Gorsuch; Frederic A. Eidsness, Jr.; Stephen Schatzow; Jeffery Denit; B. Matthew Jarrett. *Development Document for Final Effluent Limitations Guidelines and New Source Performance Standards for the Ore Mining and Dressing Point Source Category*; 0-381-085/4488; U.S. Environmental Protection Agency, 1982.

(5) Gerald A. Isaacs; W. Devitt., T.; Hal M. Drake; Douglas J. Morell; Jeffrey A. Smith. *Environmental Assessment of the Domestic Primary Copper, Lead and Zinc Industries. VOLUME I*; Margaret J. Stasikowski, Series Ed.; 68-02–1321; U.S. Environmental Protection Agency, 1976.

(6) Hallowell, J. B. *Water-Pollution Control in the Primary Nonferrous-Metals Industry*; Washington : For sale by the Supt. of Docs., U.S. Govt. Print. Off., 1973., 1973.

(7) J.s. Greber; E.A. Pfetzing; R.S. Amick; R.O. Toftner. *Assessment of Environmental Impact of the Mineral Mining Industry*; 68-03–2479; U.S. Environmental Protection Agency, 1979.

(8) Ruckelshaus, W. D.; Denit, J. D.; Hall, E. P.; Belefski, M. L. Development Document for Effluent Limitations Guidelines and Standards for Battery Manufacturing. *U.S. Environmental Protection Agency* **1984**.

(9) McLarnon, F. R.; Cairns, E. J. The Secondary Alkaline Zinc Electrode. *J. Electrochem. Soc.* **1991**, *138* (2), 645. https://doi.org/10.1149/1.2085653.

(10) Xie, W.; Zhu, K.; Yang, H.; Yang, W. Advancements in Achieving High Reversibility of Zinc Anode for Alkaline Zinc-Based Batteries. *Advanced Materials* **2024**, *36* (5), 2306154. https://doi.org/10.1002/adma.202306154.

(11) Kingsbury, R. pyEQL: A Python Interface for Water Chemistry. *JOSS* **2024**, *9* (95), 6295. https://doi.org/10.21105/joss.06295.

(12) *Mineral Commodity Summaries 2025*; 2025. https://doi.org/10.3133/mcs2025.

(13) *Samarium Price | Critical and Strategic Metals Hub*. Critical and Strategic Metals Hub. https://criticalstrategicmetals.com/minerals/samarium/price/ (accessed 2026-05-25).

(14) *Uranium Marketing Annual Report - U.S. Energy Information Administration (EIA)*. https://www.eia.gov//uranium/marketing/index.php (accessed 2026-05-25).

(15) *Praseodymium Price - Daily Update, Forecast, Historical Data*. https://earthrarest.com/praseodymium/price/ (accessed 2026-05-25).

(16) ICF Consulting. *Overview of Exploration and Production Waste Volumes and Waste Management Practices in the United States. American Petroleum Institute*; The American Petroleum Institute, 2000.

(17) C.E. Clark; J.A. Veil. *Produced Water Volumes and Management Practices in the United States*; DE-AC02-06CH11357; Argonne National Lab: Argonne National Lab, 2009.

(18) Scanlon, B. R.; Reedy, R. C.; Nicot, J.-P. Comparison of Water Use for Hydraulic Fracturing for Unconventional Oil and Gas versus Conventional Oil. *Environ. Sci. Technol.* **2014**, *48* (20), 12386–12393. https://doi.org/10.1021/es502506v.

(19) AECOM, Inc. *Petroleum Refining Water / Wastewater Use and Management*; Operations Good Practice Series; IPIECA: London SE1 8NL, United Kingdom, 2010.

(20) International Energy Agency. World Energy Statistics and Balances, 2025. https://www.iea.org/data-and-statistics/data-product/world-energy-statistics-and-balances (accessed 2025-10-31).

(21) *Frequently Asked Questions (FAQs) - U.S. Energy Information Administration (EIA)*. https://www.eia.gov/tools/faqs/faq.php (accessed 2025-02-26).

(22) *U.S. Energy Information Administration - EIA - Independent Statistics and Analysis*. https://www.eia.gov/totalenergy/data/browser/?tbl=T05.02#/?f=A&start=1949&end=2024&charted=14-8-17 (accessed 2025-02-25).

(23) 闫星周. *China's major shale oil base achieves annual output of over 1m tons*. https://www.chinadaily.com.cn/a/202411/28/WS6747d568a310f1265a1d00a5.html (accessed 2025-10-31).

(24) *China extracts commercially viable natural gas from deeper shale formations - U.S. Energy Information Administration (EIA)*. https://www.eia.gov/todayinenergy/detail.php?id=63284 (accessed 2025-10-31).

(25) China National Petroleum Corporation. *China National Petroleum Corporation 2023 Report.Pdf*; China National Petroleum Corporation, 2023. https://www.cnpc.com.cn/en/2023ar/2023Annual.shtml.

(26) SINOPEX COPR 2024 Annual Report and Accounts. *SINOPEX COPR 2024 Annual Report and Accounts*; SINOPEX COPR. https://www.annualreports.com/Company/china-petroleum-chemical-corporation.

(27) Russia's Oil Drilling Slips From Record Amid OPEC+ Output Curbs. *Bloomberg.com*. August 2, 2024. https://www.bloomberg.com/news/articles/2024-08-02/russia-s-oil-drilling-slips-from-record-amid-opec-output-curbs (accessed 2025-10-31).

(28) Shi, J.; Xu, C.; Han, Y.; Han, H. Case Study on Wastewater Treatment Technology of Coal Chemical Industry in China. *Critical Reviews in Environmental Science and Technology* **2021**, *51* (10), 1003–1044. https://doi.org/10.1080/10643389.2020.1742530.

(29) *Water Pollution Search | ECHO | US EPA*. https://echo.epa.gov/trends/loading-tool/water-pollution-search (accessed 2024-11-14).

(30) U.S. Environmental Protechtion Agency. *Technical Document: Acid Mine Drainage Prediction*; U.S. Environmental Protection Agency: Washington, DC 20460.

(31) Gray, N. F. Acid Mine Drainage Composition and the Implications for Its Impact on Lotic Systems. *Water Research* **1998**, *32* (7), 2122–2134. https://doi.org/10.1016/S0043-1354(97)00449-1.

(32) *Rapid Expansion of Electrowinning Nickel Production Capacity Casts a Huge Influence on the Nickel Market | SMM*. https://news.metal.com/newscontent/102107093/rapid-expansion-of-electrowinning-nickel-production-capacity-casts-a-huge-influence-on-the-nickel-market (accessed 2025-10-02).

(33) icsg. *Copper Factbook*. International Copper Study Group. https://icsg.org/copper-factbook/ (accessed 2025-10-02).

(34) Huang, Y.; Zhang, Z.; Cao, Y.; Han, G.; Peng, W.; Zhu, X.; Zhang, T.; Dou, Z. Overview of Cobalt Resources and Comprehensive Analysis of Cobalt Recovery from Zinc Plant Purification Residue- a Review. *Hydrometallurgy* **2020**, *193*, 105327. https://doi.org/10.1016/j.hydromet.2020.105327.

(35) Kim, J.; Kim, R.; Han, K. N. Advances in Hydrometallurgical Gold Recovery through Cementation, Adsorption, Ion Exchange and Solvent Extraction. *Minerals* **2024**, *14* (6), 607. https://doi.org/10.3390/min14060607.

(36) Hall–Héroult Process. *Wikipedia*; 2025.

(37) Can Sener, S. E.; Thomas, V. M.; Hogan, D. E.; Maier, R. M.; Carbajales-Dale, M.; Barton, M. D.; Karanfil, T.; Crittenden, J. C.; Amy, G. L. Recovery of Critical Metals from Aqueous Sources. *ACS Sustainable Chem. Eng.* **2021**, *9* (35), 11616–11634. https://doi.org/10.1021/acssuschemeng.1c03005.

(38) Carmen. *The five largest iron ore mines in operation in US*. Mining Technology. https://www.mining-technology.com/marketdata/five-largest-iron-ore-mines-the-us/ (accessed 2025-10-02).

(39) Carmen. *The five largest gold mines in operation in US*. Mining Technology. https://www.mining-technology.com/marketdata/five-largest-gold-mines-the-us/ (accessed 2025-10-02).

(40) Carmen. *The five largest lead mines in operation in US*. Mining Technology. https://www.mining-technology.com/marketdata/five-largest-lead-mines-the-us/ (accessed 2025-10-02).

(41) Carmen. *The five largest zinc mines in operation in US*. Mining Technology. https://www.mining-technology.com/marketdata/five-largest-zinc-mines-the-us/ (accessed 2025-10-02).

(42) *US defence department awards $20.6m for nickel prospecting*. https://www.mining-technology.com/news/us-defence-department-nickel-prospecting/ (accessed 2025-10-02).

(43) *Number of active mines by sector and year, 1983 - 2023 | NIOSH | CDC*. https://wwwn.cdc.gov/niosh-mining/MMWC/Mine (accessed 2025-10-02).

(44) International Energy Agency. *Coal Mid-Year Update 2025*; International Energy Agency, 2025.

(45) Survey, U. S. G. *Mineral Commodity Summaries 2024*; 2024; U.S. Geological Survey, 2024. https://doi.org/10.3133/mcs2024.

(46) Maus, V.; Giljum, S.; Gutschlhofer, J.; da Silva, D. M.; Probst, M.; Gass, S. L. B.; Luckeneder, S.; Lieber, M.; McCallum, I. A Global-Scale Data Set of Mining Areas. *Sci Data* **2020**, *7* (1), 289. https://doi.org/10.1038/s41597-020-00624-w.

(47) Avital, R. *Water Sustainability in the Semiconductor Industry*. IDE Tech. https://ide-tech.com/en/blog/why-water-sustainability-is-vital-for-the-semiconductor-industry/ (accessed 2026-01-12).

(48) *Semiconductor manufacturing and big tech's water challenge*. World Economic Forum. https://www.weforum.org/stories/2024/07/the-water-challenge-for-semiconductor-manufacturing-and-big-tech-what-needs-to-be-done/.

(49) Hu, J.; Xiao, Z.; Zhou, R.; Deng, W.; Wang, M.; Ma, S. Ecological Utilization of Leather Tannery Waste with Circular Economy Model. *Journal of Cleaner Production* **2011**, *19* (2), 221–228. https://doi.org/10.1016/j.jclepro.2010.09.018.

(50) *Information on Metal Plating | Demographics*. American Plating Company. https://www.americanplatingcompany.com/information-on-metal-plating-demographics/ (accessed 2026-01-12).

(51) Iturrondobeitia, M.; Vallejo, C.; Berroci, M.; Akizu-Gardoki, O.; Minguez, R.; Lizundia, E. Environmental Impact Assessment of LiNi1/3Mn1/3Co1/3O2 Hydrometallurgical Cathode Recycling from Spent Lithium-Ion Batteries. *ACS Sustainable Chem. Eng.* **2022**, *10* (30), 9798–9810. https://doi.org/10.1021/acssuschemeng.2c01496.

(52) *Technical Development Document for Final Supplemental Effluent Limitations Guidelines and Standards for the Steam Electric Power Generating Point Source Category*; EPA-821-R-24-004; U.S. Environmental Protection Agency, 2024.

(53) *Carbon Dioxide Mitigation Program*; Washington State, 2025; Vol. 463-85–110. https://apps.leg.wa.gov/wac/default.aspx?cite=463-85-110.

(54) *Global coal power capacity by major country 2025*. Statista. https://www.statista.com/statistics/530569/installed-capacity-of-coal-power-plants-in-selected-countries/ (accessed 2025-10-02).

(55) INTERNATIONAL ENERGY AGENCY. *The Future of Geothermal Energy*; 2024. https://www.iea.org/reports/the-future-of-geothermal-energy.

(56) Zarrouk, S. J.; Moon, H. Efficiency of Geothermal Power Plants: A Worldwide Review. *Geothermics* **2014**, *51*, 142–153. https://doi.org/10.1016/j.geothermics.2013.11.001.

(57) Ghanashyam Neupane; Daniel S Wendt. Assessment of Mineral Resources in Geothermal Brines in the US. In *42nd Workshop on Geothermal Reservoir Engineering*; Stanford University, Stanford, Californiz.

(58) GeoEnergy, T.; Cariaga, C. *ThinkGeoEnergy's Top 10 Geothermal Countries 2024 – Power*. https://www.thinkgeoenergy.com/thinkgeoenergys-top-10-geothermal-countries-2024-power/ (accessed 2025-10-31).

(59) *2.4. Gasification Process Aqueous Effluents/Wastewater*. netl.doe.gov. https://www.netl.doe.gov/research/Coal/energy-systems/gasification/gasifipedia/aqueous-effluents-wastewater (accessed 2025-03-11).

(60) Botao Zhang; Guifeng Chen; Wanwang Peng. Review on Treatment Technologies of Coal Gasification Wastewater. *Clean Coal Technology* **2023**, *29* (10), 185–198. https://doi.org/10.13226/j.issn.1006-6772.23040601.

(61) *China increased both natural gas imports and domestic production in 2021 - U.S. Energy Information Administration (EIA)*. https://www.eia.gov/todayinenergy/detail.php?id=52139 (accessed 2025-03-13).

(62) *Syngas Market Size, Share, Growth & Trends Report, 2030*. https://www.grandviewresearch.com/industry-analysis/syngas-market-report (accessed 2025-12-01).

(63) Bret-Rouzaut, N., Favennec, Jean-Pierre. Oil and Gas Exploration and Production - Reserves, Costs, Contracts (3rd Edition Revised and Updated); Editions Technip.

(64) News, O. & G. I. *Z-44 Chayvo Well*. Oil & Gas IQ. https://www.oilandgasiq.com/drilling-and-development/articles/z-44-chayvo-well-the-deepest-oil-extraction (accessed 2025-02-19).

(65) U.S. Energy Information Administration. *U.S. Oil and Natural Gas Wells by Production Rate*; U.S. Energy Information Administration: Washington, DC 20585, 2024. https://www.eia.gov/petroleum/wells/.

(66) International Energy Agency (IEA). *World Energy Statistics and Balances*. https://www.iea.org/data-and-statistics/data-product/world-energy-statistics-and-balances.

(67) U.S. Energy Information Administration. *Short-Term Energy Outlook*; U.S. Energy Information Administration, 2025.

(68) US EPA, O. *Class II Oil and Gas Related Injection Wells*. https://www.epa.gov/uic/class-ii-oil-and-gas-related-injection-wells (accessed 2025-02-19).

(69) U.S. Environmental Protection Agency. *Management of Exploration, Development and Production Wastes: Factors Informing a Decision on the Need for Regulatory Action*; U.S. Environmental Protection Agency, 2019.

(70) Spears & Associates, Inc. *Profile of the Oilfield Chemicals Market*; Spears & Associates, Inc.: Tulsa, OK, 2016. https://static1.squarespace.com/static/592512f4ff7c504ec5e3c1f0/t/5a826b80085229c5ead133c1/1518496642269/Oilfield+Chemicals_February+2016.pdf.

(71) *Average Depth of Crude Oil and Natural Gas Wells*. https://www.eia.gov/dnav/ng/ng_enr_welldep_s1_a.htm (accessed 2025-02-19).

(72) *Nearly all new U.S. crude oil and natural gas wells are horizontal or directional - U.S. Energy Information Administration (EIA)*. https://www.eia.gov/todayinenergy/detail.php?id=52138 (accessed 2025-02-19).

(73) Shih, C.-J.; Lin, S.; Strano, M. S.; Blankschtein, D. Understanding the Stabilization of Liquid-Phase-Exfoliated Graphene in Polar Solvents: Molecular Dynamics Simulations and

Kinetic Theory of Colloid Aggregation. *Journal of the American Chemical Society* **2010**, *132* (41), 14638–14648. https://doi.org/10.1021/ja1064284.

(74) Thacker, J.; Carlton, D.; Hildenbrand, Z.; Kadjo, A.; Schug, K. Chemical Analysis of Wastewater from Unconventional Drilling Operations. *Water* **2015**, *7* (4), 1568–1579. https://doi.org/10.3390/w7041568.

(75) Guo, L. J.; Li, J.; Hu, H. Y.; Liu, H. N.; Lu, J. Decontamination of Drilling Fluid Waste from Coalbed Methane Well. *AMR* **2012**, *581–582*, 962–968. https://doi.org/10.4028/www.scientific.net/AMR.581-582.962.

(76) Gamal, H.; Elkatatny, S.; Basfar, S.; Al-Majed, A. Effect of pH on Rheological and Filtration Properties of Water-Based Drilling Fluid Based on Bentonite. *Sustainability* **2019**, *11* (23), 6714. https://doi.org/10.3390/su11236714.

(77) Healy, R. W.; Alley, W. M.; Engle, M. A.; McMahon, P. B.; Bales, J. D. *The Water-Energy Nexus: An Earth Science Perspective*; Circular; Report 1407; Reston, VA, 2015; p 122. https://doi.org/10.3133/cir1407.

(78) Alberta Energy Regulator. *Water and Oil: An Overview of the Use of Water for Enhanced Oil Recovery in Alberta*; Alberta Energy Regulator, 2014.

(79) U.S. Energy Information Administration. *Annual Energy Outlook 2023*; U.S. Energy Information Administration, 2023. https://www.eia.gov/outlooks/aeo/pdf/AEO2023_Narrative.pdf.

(80) Argonne National Laboratory. *Produced Water Volumes and Mangement Practives in the United States*; DE-AC02-06CH11357; U.S. Department of Energy, 2009.

(81) All Consulting. *U.S. Produced Water Volumes and Management Practices in 2021*; 2022. https://www.gwpc.org/wp-content/uploads/2021/09/2021_Produced_Water_Volumes.pdf.

(82) Huishu Li. PRODUCED WATER QUALITY CHARACTERIZATION AND PREDICTION FOR WATTENBERG FIELD. Master Thesis, Colorado State University, Colorado State University Fort Collins, Colorado, 2013.

(83) Ferrer, I.; Thurman, E. M. Chemical Constituents and Analytical Approaches for Hydraulic Fracturing Waters. *Trends in Environmental Analytical Chemistry* **2015**, *5*, 18–25. https://doi.org/10.1016/j.teac.2015.01.003.

(84) U.S. Geological Survey; U.S. Department of the Interior. *Water Produced with Coal-Bed Methane*; U.S. Geological Survey, 2000.

(85) Zolfaghari, A.; Gehman, J.; Alessi, D. S. Cost Analysis of Wastewater Production from Conventional and Unconventional Oil and Gas Wells. *Fuel* **2022**, *323*, 124222. https://doi.org/10.1016/j.fuel.2022.124222.

(86) Al-Hameedi, A. T.; Alkinani, H. H.; Dunn-Norman, S.; Trevino, H. A.; Al-Alwani, M. A. Conventional and Eco-Friendly Hydraulic Fracturing Fluid Additives: A Review. In *SPE Hydraulic Fracturing Technology Conference and Exhibition*; SPE: The Woodlands, Texas, USA, 2020; p D021S006R003. https://doi.org/10.2118/199750-MS.

(87) U.S. Energy Information Administration. *Drilling Productivity Report*; U.S. Energy Information Administration, 2024. https://www.eia.gov/petroleum/drilling/.

(88) Madalyn S Blondes; Katherine J Knierim; Mary R. Croke; Philip A Freeman; Colin Doolan; Amanda S Herzberg; Jenna L Shelton. U.S. Geological Survey National Produced Waters Geochemical Database (Ver. 3.0, December 2023). https://doi.org/10.5066/P9DSRCZJ.

(89) Aitani, A. M. Oil Refining and Products. In *Encyclopedia of Energy*; Elsevier, 2004; pp 715–729. https://doi.org/10.1016/B0-12-176480-X/00259-X.

(90) U.S. Environmental Protechtion Agency. *Detailed Study of the Petroleum Refining Category-2019 Report*; U.S. Environmental Protection Agency, 2019.

(91) Guo, H.; Qin, Q.; Hu, M.; Chang, J.-S.; Lee, D.-J. Treatment of Refinery Wastewater: Current Status and Prospects. *Journal of Environmental Chemical Engineering* **2024**, *12* (2), 112508. https://doi.org/10.1016/j.jece.2024.112508.

(92) *Number of oil refineries 2024*. Statista. https://www.statista.com/statistics/1445314/number-of-oil-refineries-worldwide/ (accessed 2025-05-28).

(93) *U.S. Number and Capacity of Petroleum Refineries*. https://www.eia.gov/dnav/pet/pet_pnp_cap1_dcu_nus_a.htm (accessed 2025-05-28).

(94) Lawan, M. S.; Kumar, R.; Rashid, J.; Barakat, M. A. E.-F. Recent Advancements in the Treatment of Petroleum Refinery Wastewater. *Water* **2023**, *15* (20), 3676. https://doi.org/10.3390/w15203676.

(95) Shih, J.-S.; Saiers, J. E.; Anisfeld, S. C.; Chu, Z.; Muehlenbachs, L. A.; Olmstead, S. M. Characterization and Analysis of Liquid Waste from Marcellus Shale Gas Development. *Environ. Sci. Technol.* **2015**, *49* (16), 9557–9565. https://doi.org/10.1021/acs.est.5b01780.

(96) U.S. Geological Survey; U.S. Department of the Interior. *Estimates of Water Use Associated with Continuous Oil and Gas Development in the Permian Basin, Texas and New Mexico, 2010–19, with Comparisons to the Williston Basin, North Dakota and Montana*; U.S. Geological Survey, 2021.

(97) Louis Ethelbert Otts Jr. *Water Requirements of the Petroleum Refining Industry*; Water Supply Paper 1330-G; 1964. https://doi.org/10.3133/wsp1330G.

(98) Ho, S.; Cm, I.; Cn, S. Bioremoval of Heavy Metals from Effluent of Port Harcourt Refinery Using Pluerotus Ostreatus. *J Pet Environ Biotechnol* **2017**, *08* (02). https://doi.org/10.4172/2157-7463.1000324.

(99) Igbagara, P. W.; Ntekim, J. Determination of Heavy Metals Concentration in Waste Water of Warri Refining and Petrochemicals Company (WRPC). *TJST* **2021**, *2* (1), 29–37. https://doi.org/10.47524/tjst.21.5.

(100) Ghezali, K.; Bentahar, N.; Barsan, N.; Nedeff, V.; Moșneguțu, E. Potential of Canna Indica in Vertical Flow Constructed Wetlands for Heavy Metals and Nitrogen Removal from Algiers Refinery Wastewater. *Sustainability* **2022**, *14* (8), 4394. https://doi.org/10.3390/su14084394.

(101) Caenn, R.; Darley, H. C. H.; Gray, G. R. Composition and Properties of Drilling and Completion Fluids (6th Edition); Elsevier.

(102) Richard T. Coleman; Robert F. Klausmeier; Wayne C. Micheletti; J. David Colley; Nancy P. Meserole; Klaus Schwitzgebel. *Sources and Treatment of Wastewater in the Nonferrous*

Metals Industry; EPA-600/2-80-074; U.S. Environmental Protection Agency: CINCINNATI, OHIO 45268, 1980.

(103) U.S. Environmental Protection Agency. *Abandoned Mine Site Characterization and Cleanup Handbook*; U.S. Environmental Protection Agency.

(104) Diana J. Bauer; Ruby T. Nguyen; Braeton J. Smith. *Critical Materials Assessment*; Govermental DOE/EE-2756; U.S. Department of Energy, 2023.

(105) Joseph . Krisanda. *2017 Minerals Yearbook Statistical Summary*; U.S. Geological Survey, 2024.

(106) *Number of active mines by sector and year, 1983 - 2022 | NIOSH | CDC*. https://wwwn.cdc.gov/NIOSH-Mining/MMWC/Mine?StartYear=1983&EndYear=2022&SelectedMineType=&SelectedCommodity= (accessed 2024-11-13).

(107) *Montana Supreme Court Rules in Favor of Major Copper Mine*. US News & World Report. //www.usnews.com/news/best-states/montana/articles/2024-02-26/montana-supreme-court-rules-in-favor-of-major-copper-mine (accessed 2025-02-14).

(108) Livak, L. *New Mine Opens in Nevada*. National Mining Association. https://nma.org/2024/07/23/new-mine-opens-in-nevada-2/ (accessed 2025-02-14).

(109) Shaw, A. *Three uranium mines open in the US*. Mining Technology. https://www.mining-technology.com/news/three-new-uranium-mines-open-in-us/ (accessed 2025-02-14).

(110) Roy Neulicht; Jeff Shular. *Coal Cleaning Final Report*; Emission Factor Documentation for AP-42 Section 11.10; 68-D2-0159; U.S. Environmental Protechtion Agency: Research Triangle Park, NC 27711, 1995.

(111)*Global Coal MIne Tracker*. Global Energy Monitor.

(112) U.S. Energy Information Administration. *Annual Coal Report 2023*; U.S. Energy Information Administration: U.S. Department of Energy Washington, DC 20585, 2024; p 85.

(113) *Statistical Review of World Energy*; Energy institute, 2024.

(114) US EPA, O. *Ore Mining and Dressing Effluent Guidelines Documents*. https://www.epa.gov/eg/ore-mining-and-dressing-effluent-guidelines-documents (accessed 2024-11-12).

(115) *Technical Resource Document: Extraction and Beneficiation of Ores and Minerals. Volume 3 Iron*; 530-R-94–013; U.S. Environmental Protection Agency, 1994.

(116) Anne E. Gorsuch; Stephen Schatzow; Jeffery Denit; B. Matthew Jarrett. *Development Document for Final Effluent Limitations Guidelines and New Source Performance Standards for the Ore Mining and Dressing Point Source Category*; 1986.

(117) Adamovic, D.; Ishiyama, D.; Kawaraya, H.; Ogawa, Y.; Stevanovic, Z. Geochemical Characteristics and Estimation of Groundwater Pollution in Catchment Areas of Timok and Pek Rivers, Eastern Serbia: Determination of Early-Stage Groundwater Pollution in Mining Areas. *Groundwater for Sustainable Development* **2022**, *16*, 100719. https://doi.org/10.1016/j.gsd.2021.100719.

(118) Zeng, K.; Qin, W.; Jiao, F.; He, M.; Kong, L. Treatment of Mine Drainage Generated by Lead-Zinc Concentration Plant. *J. Cent. South Univ.* **2014**, *21* (4), 1453–1460. https://doi.org/10.1007/s11771-014-2085-2.

(119) Leja, J. *Surface Chemistry of Froth Flotation*; Plenum Publishing Corp.,New York, NY: United States, 1 AD.

(120) Samaniego, J.; Tanchuling, M. a. N. Treatment of Small Scale Gold Mining Wastewater Using Pilot-Scale Sedimentation and Cocopeat Filter Bed System. *Global Journal of Environmental Science and Management* **2019**, *5* (4), 461–470. https://doi.org/10.22034/GJESM.2019.04.06.

(121) Mu, Y.; Peng, Y.; Lauten, R. A. The Depression of Pyrite in Selective Flotation by Different Reagent Systems – A Literature Review. *Minerals Engineering* **2016**, *96–97*, 143–156. https://doi.org/10.1016/j.mineng.2016.06.018.

(122) Pawliszak, P.; Bradshaw-Hajek, B. H.; Skinner, W.; Beattie, D. A.; Krasowska, M. Frothers in Flotation: A Review of Performance and Function in the Context of Chemical Classification. *Minerals Engineering* **2024**, *207*, 108567. https://doi.org/10.1016/j.mineng.2023.108567.

(123) Foroutan, A.; Abbas Zadeh Haji Abadi, M.; Kianinia, Y.; Ghadiri, M. Critical Importance of pH and Collector Type on the Flotation of Sphalerite and Galena from a Low-Grade Lead–Zinc Ore. *Sci Rep* **2021**, *11* (1), 3103. https://doi.org/10.1038/s41598-021-82759-3.

(124) Kong, L.; Lv, J.; Li, J.; Ni, L.; Qin, L. The Effects of pH Regulators on the Flotation Separation of Sphalerite and Dolomite and Their Interaction Mechanism. *Applied Surface Science* **2025**, *680*, 161450. https://doi.org/10.1016/j.apsusc.2024.161450.

(125) Wan, S.; Bi, H.; Zhou, Y.; Xie, X.; Su, S.; Yin, K.; Sun, L. Graphene Oxide as High-Performance Dielectric Materials for Capacitive Pressure Sensors. *Carbon* **2017**, *114*, 209–216. https://doi.org/10.1016/j.carbon.2016.12.023.

(126) Hocking, M. B. 13 - Ore Enrichment and Smelting of Copper. In *Handbook of Chemical Technology and Pollution Control (Third Edition)*; Hocking, M. B., Ed.; Academic Press: San Diego, 2005; pp 391–420. https://doi.org/10.1016/B978-012088796-5/50016-8.

(127) Rezende Moreira, V.; Abner Rocha Lebron, Y.; Gontijo, D.; Cristina Santos Amaral, M. Membrane Distillation and Dispersive Solvent Extraction in a Closed-Loop Process for Water, Sulfuric Acid and Copper Recycling from Gold Mining Wastewater. *Chemical Engineering Journal* **2022**, *435*, 133874. https://doi.org/10.1016/j.cej.2021.133874.

(128) Batnasan, A.; Haga, K.; Huang, H.-H.; Shibayama, A. High-Pressure Oxidative Leaching and Iodide Leaching Followed by Selective Precipitation for Recovery of Base and Precious Metals from Waste Printed Circuit Boards Ash. *Metals* **2019**, *9*, 363. https://doi.org/10.3390/met9030363.

(129) Habashi, F. Bayer's Process for Alumina Production--a Historical Perspective. *Cahiers d'Histoire de l'Aluminium* **1994**, *13*.

(130) Rees, K. L.; Van Deventer, J. S. J. The Role of Metal-Cyanide Species in Leaching Gold from a Copper Concentrate. *Minerals Engineering* **1999**, *12* (8), 877–892. https://doi.org/10.1016/S0892-6875(99)00075-8.

(131) U.S. Environmental Protechtion Agency. *Water Pollution Control in the Primary Nonferrous Metals Industry Vol.II Aluminum, Mercury, Gold, Silver, Molybdenum, and Tungsten*; EPA-R2-73-247a; U.S. Environmental Protection Agency: Washington, DC 20460, 1973.

(132) Kleinmann, R. *Prediction of Water Quality at Surface Coal Mines*; The National Mine Land Reclamation Center, 2019.

(133) Pondja Jr., E. A.; Persson, K. M.; Matsinhe, N. P. A Survey of Experience Gained from the Treatment of Coal Mine Wastewater. *JWARP* **2014**, *06* (18), 1646–1658. https://doi.org/10.4236/jwarp.2014.618148.

(134) Akcil, A.; Koldas, S. Acid Mine Drainage (AMD): Causes, Treatment and Case Studies. *Journal of Cleaner Production* **2006**, *14* (12–13), 1139–1145. https://doi.org/10.1016/j.jclepro.2004.09.006.

(135) Rodríguez-Galán, M.; Baena-Moreno, F. M.; Vázquez, S.; Arroyo-Torralvo, F.; Vilches, L. F.; Zhang, Z. Remediation of Acid Mine Drainage. *Environ Chem Lett* **2019**, *17* (4), 1529–1538. https://doi.org/10.1007/s10311-019-00894-w.

(136) Navarro, M. C.; Pérez-Sirvent, C.; Martínez-Sánchez, M. J.; Vidal, J.; Tovar, P. J.; Bech, J. Abandoned Mine Sites as a Source of Contamination by Heavy Metals: A Case Study in a Semi-Arid Zone. *Journal of Geochemical Exploration* **2008**, *96* (2–3), 183–193. https://doi.org/10.1016/j.gexplo.2007.04.011.

(137) *Coal Mining Detailed Study*; EPA-821-R-08-012; U.S. Environmental Protection Agency, 2008.

(138) Li, Q.; Zhang, W. Process Development for Recovering Critical Elements from Acid Mine Drainage. *Resources, Conservation and Recycling* **2022**, *180*, 106214. https://doi.org/10.1016/j.resconrec.2022.106214.

(139) Nordstrom, D. K.; Alpers, C. N. The Geochemistry of Acid Mine Waters. In *The environmental geochemistry of mineral deposits: Part A: Processes, methods and health issues*; Geoffrey S. Plumlee, M.J. Logsdon, Eds.; Society of Economic Geologists, Series Ed.; Society of Economic Geologists, 1999; pp 133–160.

(140) Stumbea, D.; Chicoș, M. M.; Nica, V. Effects of Waste Deposit Geometry on the Mineralogical and Geochemical Composition of Mine Tailings. *Journal of Hazardous Materials* **2019**, *368*, 496–505. https://doi.org/10.1016/j.jhazmat.2019.01.071.

(141) Baker, B. J.; Banfield, J. F. Microbial Communities in Acid Mine Drainage. *FEMS Microbiology Ecology* **2003**, *44* (2), 139–152. https://doi.org/10.1016/S0168-6496(03)00028-X.

(142) Coal Mining Effluent Guidelines. https://www.epa.gov/eg/coal-mining-effluent-guidelines (accessed 2025-04-11).

(143) Maiti, D.; Ansari, I.; Rather, M. A.; Deepa, A. Comprehensive Review on Wastewater Discharged from the Coal-Related Industries – Characteristics and Treatment Strategies. *Water Science and Technology* **2019**, *79* (11), 2023–2035. https://doi.org/10.2166/wst.2019.195.

(144) Das, B.; Prakash, S.; Biswal, S. K.; Reddy, P. S. R. Settling Characteristics of Coal Washery Tailings Using Synthetic Polyelectrolytes with Fine Magnetite. **2006**, *106*.

(145) Noble, A.; Luttrell, G. H. A Review of State-of-the-Art Processing Operations in Coal Preparation. *International Journal of Mining Science and Technology* **2015**, *25* (4), 511–521. https://doi.org/10.1016/j.ijmst.2015.05.001.

(146) Jinisha, R.; Jerlin Regin, J. Bioremediation of Coal Washery Effluent Using Dual-Chambered Microbial Fuel Cell for Effective Pollutant Removal and Sustainable Power Generation. *Desalination and Water Treatment* **2025**, *323*, 101347. https://doi.org/10.1016/j.dwt.2025.101347.

(147) Shen, L.; Zhang, J.; Lai, W.; Li, M.; Huo, B. Microstructure and Mechanical Behaviors of Coal Gangue - Coal Slime Water Backfill Cementitious Materials. *Journal of Materials Research and Technology* **2022**, *20*, 3772–3783. https://doi.org/10.1016/j.jmrt.2022.08.089.

(148) Nnaji, P. C.; Okolo, B. I.; Menkiti, M. C. Nephelometric Performance Evaluation of Oxidized Starch in the Treatment of Coal Washery Effluent. *NR* **2014**, *05* (03), 79–89. https://doi.org/10.4236/nr.2014.53009.

(149) Menkiti, M. C.; Onukwuli, O. D. Impact of pH Variation on Coag-Flocculation Behaviour of Chitin Derived Coag-Flocculant in Coal Washery Effluent Medium. *JMMCE* **2011**, *10* (15), 1391–1407. https://doi.org/10.4236/jmmce.2011.1015109.

(150) Vaseem, H.; Singh, V. K.; Singh, M. P. Heavy Metal Pollution Due to Coal Washery Effluent and Its Decontamination Using a Macrofungus, Pleurotus Ostreatus. *Ecotoxicology and Environmental Safety* **2017**, *145*, 42–49. https://doi.org/10.1016/j.ecoenv.2017.07.001.

(151) Thiruvenkatachari, R.; Younes, M.; Su, S. Coal Mine Site Investigation of Wastewater Quality in Australia. *Desalination and Water Treatment* **2011**, *32* (1–3), 357–364. https://doi.org/10.5004/dwt.2011.2722.

(152) Sabah, E.; Cengiz, I. An Evaluation Procedure for Flocculation of Coal Preparation Plant Tailings. *Water Research* **2004**, *38* (6), 1542–1549. https://doi.org/10.1016/j.watres.2003.12.017.

(153) Jeffers, T. H.; Gritton, K. S.; Bennett, P. G.; Seidel, D. C. Recovery of Cobalt from Spent Copper Leach Solution Using Continuous Ion Exchange. *Nuclear and Chemical Waste Management* **1988**, *8* (1), 37–44. https://doi.org/10.1016/0191-815X(88)90006-X.

(154) Barros, K. S.; Vielmo, V. S.; Moreno, B. G.; Riveros, G.; Cifuentes, G.; Bernardes, A. M. Chemical Composition Data of the Main Stages of Copper Production from Sulfide Minerals in Chile: A Review to Assist Circular Economy Studies. *Minerals* **2022**, *12* (2), 250. https://doi.org/10.3390/min12020250.

(155) Sarker, S. K.; Haque, N.; Bhuiyan, M.; Bruckard, W.; Pramanik, B. K. Recovery of Strategically Important Critical Minerals from Mine Tailings. *Journal of Environmental Chemical Engineering* **2022**, *10* (3), 107622. https://doi.org/10.1016/j.jece.2022.107622.

(156) García, C.; Ballester, A.; González, F.; Blázquez, M. L. Pyrite Behaviour in a Tailings Pond. *Hydrometallurgy* **2005**, *76* (1–2), 25–36. https://doi.org/10.1016/j.hydromet.2004.07.010.

(157) U.S. Environmental Protechtion Agency. *Water Pollution Control in the Primary Nonferrous Metals Industry Vol.I Copper, Zinc, and Lead Industries*; EPA-R2-73-247a; U.S.

Environmental Protection Agency: Washington, DC 20460, 1973. https://nepis.epa.gov/Exe/ZyPURL.cgi?Dockey=2000WPVM.TXT.

(158) Siefert, N.; BELARBI, Z.; Fritz, A.; Wenzlick, M. NEWTS Coal Mine Drainage Dataset from Cravotta Brady (2015), 2023. https://doi.org/10.18141/1964003.

(159) Gunesegeran, K.; Kamal, M. R.; Man, H. C.; Wayayok, A.; Haider, S. A. Acid Mine Drainage and Heavy Metals Contamination of Abandoned and Active Mine Site at Old Repas Dam in Bentong, Pahang, Malaysia. *IOP Conf. Ser.: Earth Environ. Sci.* **2021**, *646* (1), 012047. https://doi.org/10.1088/1755-1315/646/1/012047.

(160) Chen, A.; Lin, C.; Lu, W.; Wu, Y.; Ma, Y.; Li, J.; Zhu, L. Well Water Contaminated by Acidic Mine Water from the Dabaoshan Mine, South China: Chemistry and Toxicity. *Chemosphere* **2007**, *70* (2), 248–255. https://doi.org/10.1016/j.chemosphere.2007.06.041.

(161) Newmont Corporation. *Realizing Our Potential 2024 Sustainability Report*; Newmont Corporation: 6900 E. Layton Avenue, Suite 700 Denver, Colorado 80237 USA, 2024.

(162) *Innovations: How Hydrometallurgy and the SX/EW Process Made Copper the "Green" Metal*. https://www.copper.org/publications/newsletters/innovations/2001/08/hydrometallurgy.html (accessed 2025-08-20).

(163) *Nickel industry - Part 3 - Processing nickel laterites, high pressure acid leaching*. https://nickelinstitute.org/en/blog/2025/march/nickel-industry-part-3-processing-nickel-laterites-high-pressure-acid-leaching (accessed 2025-08-20).

(164) Ding, Y.; Zheng, H.; Li, J.; Zhang, S.; Liu, B.; Ekberg, C.; Jian, Z. Recovery of Platinum from Spent Petroleum Catalysts: Optimization Using Response Surface Methodology. *Metals* **2019**, *9* (3), 354. https://doi.org/10.3390/met9030354.

(165) Petersen, J. Heap Leaching as a Key Technology for Recovery of Values from Low-Grade Ores – A Brief Overview. *Hydrometallurgy* **2016**, *165*, 206–212. https://doi.org/10.1016/j.hydromet.2015.09.001.

(166) Taofeek, A.; Amos, I. A. Physicochemical Characterization of Copper Ore from Akiri, Awe LGA, Nasarawa State. *SWJ* **2023**, *18* (3), 371–374. https://doi.org/10.4314/swj.v18i3.7.

(167) Thomas, D. *Annual Report on the U.S. Manufacturing Economy : 2024*; NIST AMS 600-16; National Institute of Standards and Technology (U.S.): Gaithersburg, MD, 2024; p NIST AMS 600-16. https://doi.org/10.6028/NIST.AMS.600-16.

(168) Meninger, A. *Water Sustainability in the Semiconductor Industry | IDE Tech*. IDE Tech. https://ide-tech.com/en/blog/why-water-sustainability-is-vital-for-the-semiconductor-industry/.

(169) Sim, J.; Lee, J.; Rho, H.; Park, K.-D.; Choi, Y.; Kim, D.; Kim, H.; Woo, Y. C. A Review of Semiconductor Wastewater Treatment Processes: Current Status, Challenges, and Future Trends. *Journal of Cleaner Production* **2023**, *429*, 139570. https://doi.org/10.1016/j.jclepro.2023.139570.

(170) Noman, E. A.; Ali Al-Gheethi, A.; Al-Sahari, M.; Yashni, G.; Mohamed, R. M. S. R.; Soon, C. F.; Nguyen, H.-H. T.; Vo, D.-V. N. An Insight into Microelectronics Industry Wastewater

Treatment, Current Challenges, and Future Perspectives: A Critical Review. *Appl Water Sci* **2024**, *14* (4), 64. https://doi.org/10.1007/s13201-024-02104-7.

(171) Lin, S. H.; Yang, C. R. Chemical and Physical Treatments of Chemical Mechanical Polishing Wastewater from Semiconductor Fabrication. *Journal of Hazardous Materials* **2004**, *108* (1), 103–109. https://doi.org/10.1016/j.jhazmat.2004.01.014.

(172) Manasevit, H. M. The Use of Metalorganics in the Preparation of Semiconductor Materials: Growth on Insulating Substrates. *Journal of Crystal Growth* **1972**, *13–14*, 306–314. https://doi.org/10.1016/0022-0248(72)90175-3.

(173) Nojiri, K. *Dry Etching Technology for Semiconductors*; Springer International Publishing: Cham, 2015. https://doi.org/10.1007/978-3-319-10295-5.

(174) Yota, J.; Burrows, V. A. Surface Chemistry of GaAs Treated with Buffered HF and NH4F Solutions: Slow Reactions of Process Residuals. *J. Vac. Sci. Technol. A* **1992**, *10* (4), 837–842. https://doi.org/10.1116/1.577681.

(175) Sandhu, S.; Zumeit, A.; Tian, Z.; Vinciguerra, V.; Dahiya, R. Semiconductor Manufacturing Wastewater Challenges and the Potential Solutions via Printed Electronics. *iScience* **2025**, *28* (10). https://doi.org/10.1016/j.isci.2025.113576.

(176) Tsai, J.-C.; Kumar, M.; Chen, S.-Y.; Lin, J.-G. Nano-Bubble Flotation Technology with Coagulation Process for the Cost-Effective Treatment of Chemical Mechanical Polishing Wastewater. *Separation and Purification Technology* **2007**, *58* (1), 61–67. https://doi.org/10.1016/j.seppur.2007.07.022.

(177) Proceedings of the Fifth International Symposium on Cleaning Technology in Semiconductor Device Manufacturing. In *Cleaning technology in semiconductor device manufacturing 1989 - 2007*; Ruzyllo, J., Ed.; ECS: Pennington, NJ, 2007.

(178) Istratov, A. A.; Hieslmair, H.; Weber, E. R. Iron Contamination in Silicon Technology. *Appl Phys A* **2000**, *70* (5), 489–534. https://doi.org/10.1007/s003390051074.

(179) Ruckelshaus, W. D.; Chief, G. E. S.; Kinch, R.; Pepson, D. Electrical And Electronic Components Point Source Category Phase I. *U.S. Environmental Protection Agency* **1983**.

(180) Kniffin, M. L.; Beerling, T. E.; Helms, C. R. The Effectiveness of Aqueous Cleaning for the Removal of Evaporated Iron and Copper from Silicon Surfaces. *J. Electrochem. Soc.* **1992**, *139* (4), 1195. https://doi.org/10.1149/1.2069365.

(181) Thennarasu, G.; Rajendran, S.; Kalairaj, A.; Rathore, H. S.; Panda, R. C.; Senthilvelan, T. A Comprehensive Review on the Application of Semiconductor Nanometal Oxides Photocatalyst for the Treatment of Wastewater. *Clean Techn Environ Policy* **2025**, *27* (2), 495–516. https://doi.org/10.1007/s10098-024-02960-6.

(182) Chang, B.-C.; Ho, H.-J.; Iizuka, A. Total Resource Circulation in Chemical Mechanical Polishing Wastewater Treatment from Semiconductor Manufacturing Industry: A Review. *Journal of Water Process Engineering* **2025**, *72*, 107619. https://doi.org/10.1016/j.jwpe.2025.107619.

(183) Kanwal, A.; Ilic, B. R.; Ray, C. H.; Siebein, K.; Liddle, J. A. Chromium Oxide – A Novel Sacrificial Layer Material for MEMS/NEMS and Micro/Nanofluidic Device Fabrication. *Micro and Nano Engineering* **2022**, *16*, 100145. https://doi.org/10.1016/j.mne.2022.100145.

(184) Eng, C. Y.; Yan, D.; Withanage, N.; Liang, Q.; Zhou, Y. Wastewater Treatment and Recycle from a Semiconductor Industry: A Demo-Plant Study. *Water Practice and Technology* **2019**, *14* (2), 371–379. https://doi.org/10.2166/wpt.2019.020.

(185) Sturgill, J. A.; Swartzbaugh, J. T.; Randall, P. M. Pollution Prevention in the Semiconductor Industry through Recovery and Recycling of Gallium and Arsenic from GaAs Polishing Wastes. *Clean Products and Processes* **2000**, *2* (1), 18–27. https://doi.org/10.1007/s100980050047.

(186) Norga, G. J.; Kimerling, L. C. Metal Removal from Silicon Surfaces in Wet Chemical Systems. *J. Electron. Mater.* **1995**, *24* (4), 397–404. https://doi.org/10.1007/BF02659705.

(187) *2023 TSMC Sustainability Report*; 2023.

(188) *2024 TSMC Sustainability Report*; 2024.

(189) Appiah-Brempong, M.; Essandoh, H. M. K.; Asiedu, N. Y.; Dadzie, S. K.; Momade, F. W. Y. Artisanal Tannery Wastewater: Quantity and Characteristics. *Heliyon* **2021**, *8* (1), e08680. https://doi.org/10.1016/j.heliyon.2021.e08680.

(190) Chowdhury, M.; Mostafa, M. G.; Biswas, T. K.; Mandal, A.; Saha, A. K. Characterization of the Effluents from Leather Processing Industries. *Environ. Process.* **2015**, *2* (1), 173–187. https://doi.org/10.1007/s40710-015-0065-7.

(191) Hansen, É.; de Aquim, P. M.; Gutterres, M. Environmental Assessment of Water, Chemicals and Effluents in Leather Post-Tanning Process: A Review. *Environmental Impact Assessment Review* **2021**, *89*, 106597. https://doi.org/10.1016/j.eiar.2021.106597.

(192) Bhardwaj, A.; Kumar, S.; Singh, D. Tannery Effluent Treatment and Its Environmental Impact: A Review of Current Practices and Emerging Technologies. *Water Quality Research Journal* **2023**, *58* (2), 128–152. https://doi.org/10.2166/wqrj.2023.002.

(193) Wang, X.; Jiang, J.; Gao, W. Reviewing Textile Wastewater Produced by Industries: Characteristics, Environmental Impacts, and Treatment Strategies. *Water Science and Technology* **2022**, *85* (7), 2076–2096. https://doi.org/10.2166/wst.2022.088.

(194) Yu, Y.; Lin, Y.; Zeng, Y.; Wang, Y.; Zhang, W.; Zhou, J.; Shi, B. Life Cycle Assessment for Chrome Tanning, Chrome-Free Metal Tanning, and Metal-Free Tanning Systems. *ACS Sustainable Chem. Eng.* **2021**, *9* (19), 6720–6731. https://doi.org/10.1021/acssuschemeng.1c00753.

(195) *Materials Market Report 2024*. Textile Exchange. https://textileexchange.org/knowledge-center/reports/materials-market-report-2024/.

(196) Muralidharan, V.; Palanivel, S.; Balaraman, M. Turning Problem into Possibility: A Comprehensive Review on Leather Solid Waste Intra-Valorization Attempts for Leather Processing. *Journal of Cleaner Production* **2022**, *367*, 133021. https://doi.org/10.1016/j.jclepro.2022.133021.

(197) Taghipour, M.; Jalali, M. Influence of Organic Acids on Kinetic Release of Chromium in Soil Contaminated with Leather Factory Waste in the Presence of Some Adsorbents. *Chemosphere* **2016**, *155*, 395–404. https://doi.org/10.1016/j.chemosphere.2016.04.063.

(198) Meriç, S.; Nicola, E. D.; Iaccarino, M.; Gallo, M.; Gennaro, A. D.; Morrone, G.; Warnau, M.; Belgiorno, V.; Pagano, G. Toxicity of Leather Tanning Wastewater Effluents in Sea Urchin Early Development and in Marine Microalgae. *Chemosphere* **2005**, *61* (2), 208–217. https://doi.org/10.1016/j.chemosphere.2005.02.037.

(199) Nur-E-Alam, Md.; Mia, Md. A. S.; Ahmad, F.; Rahman, Md. M. An Overview of Chromium Removal Techniques from Tannery Effluent. *Appl Water Sci* **2020**, *10* (9), 205. https://doi.org/10.1007/s13201-020-01286-0.

(200) Arti; Mehra, R. Analysis of Heavy Metals and Toxicity Level in the Tannery Effluent and the Environs. *Environ Monit Assess* **2023**, *195* (5), 554. https://doi.org/10.1007/s10661-023-11154-4.

(201) Jaffari, Z. H.; Hong, J.; Park, K. Y. A Systematic Review of Innovations in Tannery Solid Waste Treatment: A Viable Solution for the Circular Economy. *Science of The Total Environment* **2024**, *948*, 174848. https://doi.org/10.1016/j.scitotenv.2024.174848.

(202) Ma, H.; Zhou, J.; Hua, L.; Cheng, F.; Zhou, L.; Qiao, X. Chromium Recovery from Tannery Sludge by Bioleaching and Its Reuse in Tanning Process. *Journal of Cleaner Production* **2017**, *142*, 2752–2760. https://doi.org/10.1016/j.jclepro.2016.10.193.

(203) Guo, S.; Wang, H.; Liu, X.; Zhang, Z.; Liu, Y. Approaches for the Treatment and Resource Utilization of Electroplating Sludge. *Materials* **2024**, *17* (7), 1707. https://doi.org/10.3390/ma17071707.

(204) Costle, D. M.; Davis, S.; Schaffer, R.; Hall, E.; Hanson, J. B. ELECTROPLATING POINT SOURCE CATEGORY. *United States Environmental Protection Agency (EPA)* **1979**.

(205) Fu, D.; Kurniawan, T. A.; Avtar, R.; Xu, P.; Othman, M. H. D. Recovering Heavy Metals from Electroplating Wastewater and Their Conversion into Zn2Cr-Layered Double Hydroxide (LDH) for Pyrophosphate Removal from Industrial Wastewater. *Chemosphere* **2021**, *271*, 129861. https://doi.org/10.1016/j.chemosphere.2021.129861.

(206) Parkinson, R. PLATING AND ELECTROFORMING-ESSENTIAL INDUSTRIES FOR TODAY AND THE FUTURE, 2021.

(207) Matlock, M. M.; Howerton, B. S.; Atwood, D. A. Chemical Precipitation of Lead from Lead Battery Recycling Plant Wastewater. *Ind. Eng. Chem. Res.* **2002**, *41* (6), 1579–1582. https://doi.org/10.1021/ie010800y.

(208) Brückner, L.; Frank, J.; Elwert, T. Industrial Recycling of Lithium-Ion Batteries—A Critical Review of Metallurgical Process Routes. *Metals* **2020**, *10* (8), 1107. https://doi.org/10.3390/met10081107.

(209) Zhang, X.; Zhu, M. Recycling Spent Lithium-Ion Battery Cathode: An Overview. *Green Chem.* **2024**, *26* (13), 7656–7717. https://doi.org/10.1039/D4GC01781A.

(210) Salces, A. M.; Kelly, N.; Streblow, G. J.; Temel, E. T.; Rudolph, M.; Chagnes, A.; Vanderbruggen, A. A Contribution to Understanding Ion-Exchange Mechanisms for Lithium Recovery from Industrial Effluents of Lithium-Ion Battery Recycling Operations. *Journal of Environmental Chemical Engineering* **2024**, *12* (3), 112951. https://doi.org/10.1016/j.jece.2024.112951.

(211) Tembo, P. M.; Dyer, C.; Subramanian, V. Lithium-Ion Battery Recycling—a Review of the Material Supply and Policy Infrastructure. *NPG Asia Mater* **2024**, *16* (1), 43. https://doi.org/10.1038/s41427-024-00562-8.

(212) *Lithium-ion Battery Recycling: Market and Innovation Trends for a Green Future*. https://www.cas.org/resources/cas-insights/lithium-ion-battery-recycling-trends.

(213) Baum, Z. J.; Bird, R. E.; Yu, X.; Ma, J. Lithium-Ion Battery Recycling─Overview of Techniques and Trends. *ACS Energy Lett.* **2022**, *7* (2), 712–719. https://doi.org/10.1021/acsenergylett.1c02602.

(214) Kim, S.; Kim, J.; Kim, S.; Lee, J.; Yoon, J. Electrochemical Lithium Recovery and Organic Pollutant Removal from Industrial Wastewater of a Battery Recycling Plant. *Environ. Sci.: Water Res. Technol.* **2018**, *4* (2), 175–182. https://doi.org/10.1039/C7EW00454K.

(215) Wagner-Wenz, R.; Teppala, D. T.; Necke, T.; Brückner, F.; Fabian, A.; Horn, D.; Woth, J.; Zimmermann, J.; Balke-Grünewald, B.; Weidenkaff, A.; Ionescu, E. Removal and Recovery of Phosphorus and Fluorine in Process Water from Water Based Direct Physical Lithium-Ion Battery Recycling. *Water Research* **2025**, *268*, 122476. https://doi.org/10.1016/j.watres.2024.122476.

(216) Hyder, A. H. M. G.; Nuraeni, B.; Gallagher, S. M.; Wasilk, M.; Macholz, J. D.; Lipson, A. L.; Spangenberger, J. Lithium Recovery and Conversion from Wastewater Produced by Recycling of Li-Ion Batteries via Two-Stage Electrodialysis. *ACS Sustainable Chem. Eng.* **2025**, *13* (12), 4651–4660. https://doi.org/10.1021/acssuschemeng.4c07708.

(217) Fang, D.; Wu, F.; Zhang, X.; Zhang, X.; Ye, Y.; Chen, R.; Li, L. Direct Upcycling of Degraded LiCoO2 Towards High-Performance Cathodes via a Multifunctional Organic Lithium Salt. *Angewandte Chemie International Edition* **2025**, *64* (17), e202502176. https://doi.org/10.1002/anie.202502176.

(218) Du, K.; Ang, E. H.; Wu, X.; Liu, Y. Progresses in Sustainable Recycling Technology of Spent Lithium-Ion Batteries. *ENERGY & ENVIRONMENTAL MATERIALS* **2022**, *5* (4), 1012–1036. https://doi.org/10.1002/eem2.12271.

(219) Velázquez-Martínez, O.; Valio, J.; Santasalo-Aarnio, A.; Reuter, M.; Serna-Guerrero, R. A Critical Review of Lithium-Ion Battery Recycling Processes from a Circular Economy Perspective. *Batteries* **2019**, *5* (4), 68. https://doi.org/10.3390/batteries5040068.

(220) Barbosa de Mattos, D. F.; Duda, S.; Petranikova, M. Recycling of Lithium Iron Phosphate (LiFePO4) Batteries from the End Product Quality Perspective. *Batteries* **2025**, *11* (1), 33. https://doi.org/10.3390/batteries11010033.

(221) Carena, E.; Morina, R.; Brugnetti, G.; Pianta, N.; Binetti, S. O.; Ferrara, C. The Recycling of Lithium-Ion Batteries LiCoO2 Cathode: Balancing Sustainability and Efficiency. *J. Chem. Educ.* **2024**, *101* (5), 2124–2133. https://doi.org/10.1021/acs.jchemed.4c00204.

(222) Rey, I.; Vallejo, C.; Santiago, G.; Iturrondobeitia, M.; Lizundia, E. Environmental Impacts of Graphite Recycling from Spent Lithium-Ion Batteries Based on Life Cycle Assessment. *ACS Sustainable Chem. Eng.* **2021**, *9* (43), 14488–14501. https://doi.org/10.1021/acssuschemeng.1c04938.

(223) Heiskanen, S. K.; Kim, J.; Lucht, B. L. Generation and Evolution of the Solid Electrolyte Interphase of Lithium-Ion Batteries. *Joule* **2019**, *3* (10), 2322–2333. https://doi.org/10.1016/j.joule.2019.08.018.

(224) Nie, M.; Abraham, D. P.; Chen, Y.; Bose, A.; Lucht, B. L. Silicon Solid Electrolyte Interphase (SEI) of Lithium Ion Battery Characterized by Microscopy and Spectroscopy. *J. Phys. Chem. C* **2013**, *117* (26), 13403–13412. https://doi.org/10.1021/jp404155y.

(225) Jegan Roy, J.; Srinivasan, M.; Cao, B. Bioleaching as an Eco-Friendly Approach for Metal Recovery from Spent NMC-Based Lithium-Ion Batteries at a High Pulp Density. *ACS Sustainable Chem. Eng.* **2021**, *9* (8), 3060–3069. https://doi.org/10.1021/acssuschemeng.0c06573.

(226) *40 CFR Part 469 -- Electrical and Electronic Components Point Source Category*. U.S. Environmental Protection Agency. https://www.ecfr.gov/current/title-40/part-469.

(227) Karakaş, İ.; Kızıl, S. A Critical Study on the Treatability of Metal Plating Industry Wastewater and Real Scale Adaptation. *TUJE* **2024**, *8* (3), 427–435. https://doi.org/10.31127/tuje.1406437.

(228) Akbal, F.; Camcı, S. Treatment of Metal Plating Wastewater by Electrocoagulation. *Environmental Progress & Sustainable Energy* **2012**, *31* (3), 340–350. https://doi.org/10.1002/ep.10546.

(229) Singh, V.; Ram, C. Physico-Chemical Characterization of Electroplating Industrial Effluents of Chandigarh and Haryana Region. *J Civil Environ Eng* **2016**, *6* (4). https://doi.org/10.4172/2165-784X.1000237.

(230) Tsang, KL; Chiu, H.; Lee, RML. Treatment of Electroplating Wastes. *J. Inst. Water Pollut. Control* **1987**, *86(1)*, 12–19.

(231) Alemu, A.; Gabbiye, N.; Lemma, B. Evaluation of Tannery Wastewater Treatment by Integrating Vesicular Basalt With Local Plant Species in a Constructed Wetland System. *Front. Environ. Sci.* **2021**, *9*. https://doi.org/10.3389/fenvs.2021.721014.

(232) Purkait, M. K.; Kumar, V. D.; Maity, D. Treatment of Leather Plant Effluent Using NF Followed by RO and Permeate Flux Prediction Using Artificial Neural Network. *Chemical Engineering Journal* **2009**, *151* (1), 275–285. https://doi.org/10.1016/j.cej.2009.03.023.

(233) Ubale, M. A.; Salkar, V. D. Experimental Study on Electrocoagulation of Textile Wastewater by Continuous Horizontal Flow through Aluminum Baffles. *Korean J. Chem. Eng.* **2017**, *34* (4), 1044–1050. https://doi.org/10.1007/s11814-016-0351-8.

(234) Alam, M. S.; Han, B.; Al-Mizan, null; Pichtel, J. Assessment of Soil and Groundwater Contamination at a Former Tannery District in Dhaka, Bangladesh. *Environ Geochem Health* **2020**, *42* (7), 1905–1920. https://doi.org/10.1007/s10653-019-00457-6.

(235) Haque, K. Guide for Assessment of Effluent Treatment Plants in EMP/EIA Reports for Textile Industries, 2008.

(236) Shehzadi, M.; Afzal, M.; Khan, M. U.; Islam, E.; Mobin, A.; Anwar, S.; Khan, Q. M. Enhanced Degradation of Textile Effluent in Constructed Wetland System Using *Typha Domingensis* and Textile Effluent-Degrading Endophytic Bacteria. *Water Research* **2014**, *58*, 152–159. https://doi.org/10.1016/j.watres.2014.03.064.

(237) Hussain, Z.; Arslan, M.; Malik, M. H.; Mohsin, M.; Iqbal, S.; Afzal, M. Integrated Perspectives on the Use of Bacterial Endophytes in Horizontal Flow Constructed Wetlands for the Treatment of Liquid Textile Effluent: Phytoremediation Advances in the Field. *Journal of Environmental Management* **2018**, *224*, 387–395. https://doi.org/10.1016/j.jenvman.2018.07.057.

(238) Tara, N.; Arslan, M.; Hussain, Z.; Iqbal, M.; Khan, Q. M.; Afzal, M. On-Site Performance of Floating Treatment Wetland Macrocosms Augmented with Dye-Degrading Bacteria for the Remediation of Textile Industry Wastewater. *Journal of Cleaner Production* **2019**, *217*, 541–548. https://doi.org/10.1016/j.jclepro.2019.01.258.

(239) Hussein, F. H. Chemical Properties of Treated Textile Dyeing Wastewater. *Asian J. Chem.* **2013**, *25* (16), 9393–9400. https://doi.org/10.14233/ajchem.2013.15909A.

(240) Macchi, G.; Pagano, M.; Santori, M.; Tiravanti, G. Battery Industry Wastewater: Pb Removal and Produced Sludge. *Water Research* **1993**, *27* (10), 1511–1518. https://doi.org/10.1016/0043-1354(93)90095-Y.

(241) Saleem, U.; Buvik, V.; Bandyopadhyay, S.; Knuutila, H. K. Selective Extraction of Lithium from Acidic Chloride Leachates of Spent Batteries. *Sci Rep* **2026**, *16* (1), 14984. https://doi.org/10.1038/s41598-026-43332-y.

(242) Wu, S.; Zhu, H.; Wu, Y.; Li, S.; Zhang, G.; Miao, Z. Resourceful Treatment of Battery Recycling Wastewater Containing H2SO4 and NiSO4 by Diffusion Dialysis and Electrodialysis. *Membranes* **2023**, *13* (6), 570. https://doi.org/10.3390/membranes13060570.

(243) Electric Power Sector Basics. https://www.epa.gov/power-sector/electric-power-sector-basics (accessed 2025-06-11).

(244) Cleveland, C. Fuel Energy Density: What Is It and Why Is It Important, 2025. https://visualizingenergy.org/fuel-energy-density-what-is-it-and-why-is-it-important/ (accessed 2025-06-11).

(245) International Energy Agency. *Key World Energy Statistics 2021*; Statistics report; International Energy Agency, 2021.

(246) Steam Electric Power Generating Effluent Guidelines. https://www.epa.gov/eg/steam-electric-power-generating-effluent-guidelines (accessed 2025-03-11).

(247) What Is U.S. Electricity Generation by Energy Source? https://www.eia.gov/tools/faqs/faq.php?id=427&t=3 (accessed 2025-03-21).

(248) *Supplemental Effluent Limitations Guidelines and Standards for the Steam Electric Power Generating Point Source Category*; EPA–HQ–OW–2009–0819; U.S. Environmental Protection Agency, 2024.

(249) Cheryl A. Dieter; Molly A. Maupin; Rodney R. Caldwell; Melissa A. Harris; Tamara I. Ivahnenko; John K. Lovelace; Nancy L. Barber; Kristin S. Linsey. *Estimated Use of Water in the United States in 2015: U.S. Geological Survey Circular 1441*; Circular 1441; 2017–1131; U.S. Department of the Interior, U.S. Geological Survey: U.S. Geological Survey, Reston, Virginia: https://doi.org/10.3133/cir1441.

(250) Sulfur Dioxide Basics. https://www.epa.gov/so2-pollution/sulfur-dioxide-basics (accessed 2025-06-11).

(251) Power Plant Emissions of Sulfur Dioxide and Nitrogen Oxides Continue to Decline in 2012. February 27, 2013. https://www.eia.gov/todayinenergy/detail.php?id=10151 (accessed 2025-03-31).

(252) Power Plants and Neighboring Communities. https://www.epa.gov/power-sector/power-plants-and-neighboring-communities#:~:text=Burning%20fossil%20fuels%20at%20power%20plants%20creates,dioxide%20(CO2)%2C%20mercury%20(Hg)%2C%20and%20other%20pollutants.&text=Over%20the%20past%20two%20decades%2C%20the%20power,but%20important%20health%20and%20environmental%20concerns%20remain. (accessed 2025-03-31).

(253) Air Pollution Control Technology Fact Sheet, 2003. https://www3.epa.gov/ttncatc1/dir1/ffdg.pdf (accessed 2025-03-31).

(254) Form EIA-923 Detailed Data with Previous Form Data (EIA-906/920).

(255) Gingerich, D. B.; Grol, E.; Mauter, M. S. Fundamental Challenges and Engineering Opportunities in Flue Gas Desulfurization Wastewater Treatment at Coal Fired Power Plants. *Environ. Sci.: Water Res. Technol.* **2018**, *4* (7), 909–925. https://doi.org/10.1039/C8EW00264A.

(256) Wenzlick, M.; Siefert, N.; BELARBI, Z. NEWTS EPA FGD Effluent Database, 2022. https://doi.org/10.18141/1890169.

(257) Gingerich, D. B.; Mauter, M. S. Flue Gas Desulfurization Wastewater Composition and Implications for Regulatory and Treatment Train Design. *Environ. Sci. Technol.* **2020**, *54* (7), 3783–3792. https://doi.org/10.1021/acs.est.9b07433.

(258) *Treatment Technology Summary for Critical Pollutants of Concern in Power Plant Wastewaters*; 1012549; ELECTRIC POWER RESEARCH INSTITUTE, 2007.

(259) Pandey, R. A.; Biswas, R.; Chakrabarti, T.; Devotta, S. Flue Gas Desulfurization: Physicochemical and Biotechnological Approaches. *Critical Reviews in Environmental Science and Technology* **2005**, *35* (6), 571–622. https://doi.org/10.1080/10643380500326374.

(260) Poullikkas, A. Review of Design, Operating, and Financial Considerations in Flue Gas Desulfurization Systems. *Energy Technology & Policy* **2015**, *2* (1), 92–103. https://doi.org/10.1080/23317000.2015.1064794.

(261) Siefert, N.; BELARBI, Z.; Wenzlick, M.; Fritz, A. NEWTS EPA Ash Dataset, 2023. https://doi.org/10.18141/1909006.

(262) Amrika, D.; Allan, K.; Michael, D. Trace Elements in Coal Ash, 2015. https://pubs.usgs.gov/fs/2015/3037/pdf/fs2015-3037.pdf (accessed 2025-10-31).

(263) *Coal Combustion Product Landfill Terminology and Water Management Fundamentals*; The Electric Power Research Institute (EPRI), 2021.

(264) Able, C.; Rellergert, D.; Mazzoni, V.; Grol, E. Assessment of Combustion Residual Leachate Volume, Composition, and Treatment Costs. *Journal of Hazardous Materials* **2023**, *457*, 131731. https://doi.org/10.1016/j.jhazmat.2023.131731.

(265) Able, C.; Fritz, A.; Grol, E. Assessment of Combustion Residual Leachate: Local Treatment Needs and Critical Mineral Recovery. *Resources, Conservation and Recycling* **2024**, *205*, 107535. https://doi.org/10.1016/j.resconrec.2024.107535.

(266) Able, C.; Rellergert, D.; Mazzoni, V.; Grol, E. Assessment of Combustion Residual Leachate Volume, Composition, and Treatment Costs. *Journal of Hazardous Materials* **2023**, *457*, 131731. https://doi.org/10.1016/j.jhazmat.2023.131731.

(267) Siefert, N.; BELARBI, Z.; Wenzlick, M.; Fritz, A. NEWTS EPA Leachate Dataset, 2023. https://doi.org/10.18141/1909007.

(268) Byers, L.; Friedrich, J.; Hennig, R.; Kressig, A.; Li, X.; Valeri, L. M.; McCormick, C. A Global Database of Power Plants. **2018**.

(269) Weakle, D. *Zero Liquid Discharge: Bottom Ash Transport Water*; White paper; Gai consultants, 2016.

(270) Gutiérrez-Negrín, L. C. A. Evolution of Worldwide Geothermal Power 2020–2023. *Geothermal Energy* **2024**, *12* (1), 14. https://doi.org/10.1186/s40517-024-00290-w.

(271) *Geothermal Energy: Utah*. Bureau of Land Management. https://www.blm.gov/programs/energy-and-minerals/renewable-energy/geothermal-energy/regional-information/utah (accessed 2025-04-24).

(272) *Loan Agreement Signed for Expansion of Muara Laboh Geothermal Power Project in Indonesia*. Sumitomo Corporation in East Asia. http://www.sumitomocorp.com/en/easia//news/release/2025/group/18900 (accessed 2025-04-24).

(273) Bauer, S. Trump Administration's Policies Support Rapid Growth of Geothermal Power in the United States with 1.2 GW Planned by End of Term. **2025**.

(274) SINOPEC. *Sinopec's First Geothermal Power Production Pilot Project Connects to Grid*. https://www.prnewswire.com/news-releases/sinopecs-first-geothermal-power-production-pilot-project-connects-to-grid-302297203.html (accessed 2025-04-24).

(275) *Geothermal Energy from Oil and Gas Demonstrated Engineering*. Energy.gov. https://www.energy.gov/eere/geothermal/geothermal-energy-oil-and-gas-demonstrated-engineering (accessed 2025-04-24).

(276) Clark, C. E.; Harto, C. B.; Sullivan, J. L.; Wang, M. Q. *Water Use in the Development and Operation of Geothermal Power Plants.*; ANL/EVS/R-10/5, 1013997; 2010; p ANL/EVS/R-10/5, 1013997. https://doi.org/10.2172/1013997.

(277) Higman, C.; van der Burgt, M. Gasification (2nd Edition); Elsevier.

(278) Jensen, J. H.; Poulsen, J. M.; Andersen, N. U. *From Coal to Clean Energy*; Topsoe A/S, 2011.

(279) Lestander, T. A.; Weiland, F.; Grimm, A.; Rudolfsson, M.; Wiinikka, H. Gasification of Pure and Mixed Feedstock Components: Effect on Syngas Composition and Gasification Efficiency. *Journal of Cleaner Production* **2022**, *369*, 133330. https://doi.org/10.1016/j.jclepro.2022.133330.

(280) AG, A. *Klabin successfully starts up new gasification plant supplied by ANDRITZ*. https://www.andritz.com/newsroom-en/pulp-paper/2022-11-10-klabin-wsa-group (accessed 2025-03-11).

(281) Coca, N. *A US company is building a massive coal gasification plant in Indonesia*. Energy Monitor. https://www.energymonitor.ai/sectors/industry/a-us-company-is-quietly-building-a-massive-coal-to-gas-plant-in-indonesia (accessed 2025-03-11).

(282) David L. Denton. Commercial-Scale Coal Gasification: Lessons Learned and R&D Needs. Gasification Workshop Presentations, 2022. https://www.energy.gov/eere/bioenergy/gasification-workshop-presentations.

(283) Siefert, N.; Fritz, A.; BELARBI, Z.; Wenzlick, M. NEWTS EPA Gasification Dataset and Case Studies, 2023. https://doi.org/10.18141/1974171.

(284) Wang, W.; Han, H.; Yuan, M.; Li, H.; Fang, F.; Wang, K. Treatment of Coal Gasification Wastewater by a Two-Continuous UASB System with Step-Feed for COD and Phenols Removal. *Bioresource Technology* **2011**, *102* (9), 5454–5460. https://doi.org/10.1016/j.biortech.2010.10.019.

(285) Zhang, W.; Ma, J.; Yang, S.; Zhang, T.; Li, Y. Pretreatment of Coal Gasification Wastewater by Acidification Demulsion. *Chinese Journal of Chemical Engineering* **2006**, *14* (3), 398–401. https://doi.org/10.1016/S1004-9541(06)60090-6.

(286) Vassilev, S. V.; Baxter, D.; Andersen, L. K.; Vassileva, C. G. An Overview of the Chemical Composition of Biomass. *Fuel* **2010**, *89* (5), 913–933. https://doi.org/10.1016/j.fuel.2009.10.022.

(287) US EPA, O. *Guide to the Facts and Figures Report about Materials, Waste and Recycling*. https://www.epa.gov/facts-and-figures-about-materials-waste-and-recycling/guide-facts-and-figures-report-about (accessed 2025-12-01).
